# Alterations in Tissue Metabolite Profiles with Amifostine-Prophylaxed Mice Exposed to Gamma Radiation

**DOI:** 10.3390/metabo10050211

**Published:** 2020-05-21

**Authors:** Amrita K. Cheema, Yaoxiang Li, Michael Girgis, Meth Jayatilake, Oluseyi O. Fatanmi, Stephen Y. Wise, Thomas M. Seed, Vijay K. Singh

**Affiliations:** 1 Department of Oncology, Lombardi Comprehensive Cancer Center, Georgetown University Medical Center, Washington, DC 20057, USA; akc27@georgetown.edu (A.K.C.); yl814@georgetown.edu (Y.L.); mg1773@georgetown.edu (M.G.); mmj61@georgetown.edu (M.J.); 2 Department of Biochemistry, Molecular and Cellular Biology, Georgetown University Medical Center, Washington, DC 20007, USA; 3 Division of Radioprotectants, Department of Pharmacology and Molecular Therapeutics, F. Edward Hébert School of Medicine, Uniformed Services University of the Health Sciences, Bethesda, MD 20814, USA; oluseyi.fatanmi@usuhs.edu (O.O.F.); stephen.wise.ctr@usuhs.edu (S.Y.W.); 4 Armed Forces Radiobiology Research Institute, Uniformed Services University of the Health Sciences, Bethesda, MD 20814, USA; 5 Tech Micro Services, Bethesda, MD 20814, USA; tmseed@verizon.net

**Keywords:** acute radiation syndrome, amifostine, biomarker, gamma radiation, lipidomes, metabolites, mice, tissue

## Abstract

Acute exposure to high-dose ionizing irradiation has the potential to severely injure the hematopoietic system and its capacity to produce vital blood cells that innately serve to ward off infections and excessive bleeding. Developing a medical radiation countermeasure that can protect individuals from the damaging effects of irradiation remains a significant, unmet need and an area of great public health interest and concern. Despite significant advancements in the field of radiation countermeasure development to find a nontoxic and effective prophylactic agent for acute radiation syndrome, no such drug has yet been approved by the Food and Drug Administration. This study focuses on examining the metabolic corrections elicited by amifostine, a potent radioprotector, on tissues of vital body organs, such as the heart, spleen, and kidney. Our findings indicate that prophylaxis with this drug offers significant protection against potentially lethal radiation injury, in part, by correction of radiation-induced metabolic pathway perturbations.

## 1. Introduction

Unwanted exposures to ionizing radiation from either intentional use of radiological/nuclear devices or unintentional radiological accidents can lead to serious, sometimes life-threatening injuries [1]. Acute radiation syndrome (ARS) manifests after acute exposures to whole-body or partial-body radiation at doses above 1 Gray (Gy) delivered at relatively high dose rates of ~0.05 Gy/h or higher. Clinical manifestations of ARS are often categorized into three distinct subsyndromes, each with assigned ranges of eliciting radiation doses, namely, the hematopoietic subsyndrome (H-ARS, 1–6 Gy), the gastrointestinal subsyndrome (GI-ARS, >6 Gy), and the neurovascular subsyndrome (>10 Gy) [2]. The neurovascular subsyndrome is characterized clinically by vascular accidents that are often systemic in nature and by the occurrence of multiorgan failure. Fatal outcomes appear to be inevitable. This subsyndrome, when manifested, is exceedingly difficult to manage clinically and, as such, is often considered untreatable.

Medical radiation countermeasures have been divided into three groups depending on the timing of drug administration relative to the irradiation event which include radioprotectors, radiomitigators, and radiation therapeutics [3]. Radioprotectors are prophylactic agents administered before irradiation and carry the expectation that they will protect individuals that are subsequently irradiated. Unfortunately, to date, no radioprotector for either H-ARS or GI-ARS has been approved by the US Food and Drug Administration (FDA) [4]. The latter situation is in spite of the rather large number of possible radioprotective candidates that have been identified, synthesized, and evaluated over the last six decades. Nevertheless, this national research effort yielded the successful identification of at least one major group of chemical agents with strong radioprotective attributes, namely, the phosphoroaminothioates [3]. Within this group, an agent originally designated WR-2721 and now commonly referred to as amifostine has been shown to have exceptional radioprotective ability when tested in preclinical animal models [3,5]. Amifostine has been investigated extensively by many scientists at various research institutions and was generally found to have unparalleled efficacy, but with questionable safety profiles [3,6,7,8,9,10]. Initial investigations in animal models demonstrated that amifostine was capable of protecting animals against high doses of lethal radiation [3]. In addition, amifostine is known as a systemic cytoprotective agent [11]. Preclinical studies demonstrated that amifostine is a powerful, systemically effective radiation countermeasure capable of protecting normal tissues against radiation injury [12,13]. Unfortunately, despite all positive attributes listed above, amifostine was found to be toxic to humans with serious side effects when administered at higher doses needed to provide radioprotection. Amifostine is a hypotensive eliciting agent, leading to both lower and upper gastrointestinal disturbances and to performance decrement [14,15,16,17,18]. Due to its significant adverse side effects, amifostine did not get FDA approval for use as a radioprotective agent for ARS [19,20]. Investigators at various institutions have worked diligently to reduce its toxic side effects without losing its radioprotective efficacy through multiple, rather novel approaches. At this point in time, however, the outcome from such a combined effort has not been encouraging but may prove useful in the future [3]. Nevertheless, amifostine has been approved by the FDA for limited indications:

(a) to decrease the xerostomia in malignant patients receiving radiotherapy after surgery for head and neck cancer and
(b) to reduce the renal toxicity of repeated use of cisplatin in ovarian cancer patients [21,22].

Several studies have been conducted to elucidate the differences between high and low doses of amifostine [3,11,23,24]. In addition, serious efforts have been made by a large number of investigators to combine amifostine with other promising radiation countermeasures in order to decrease its side effects during the treatment of H-ARS [3,11,23].

It has previously been reported that treatment with amifostine at a dose of 200 mg/kg led to a high survival benefit in irradiated mice, while a lower dose of 50 mg/kg resulted in limited benefit [25]. These results demonstrated that the use of amifostine elicits metabolic shifts that would later offer benefits in terms of recovery from potentially lethal radiation injuries. Amifostine prophylaxis resulted in the correction of specific metabolic processes dysregulated by radiation exposure in bone marrow, lung, and jejunum of mice in a dose-dependent manner [25]. Bone marrow exhibited strong responses to radiation exposure and was associated with the protective effects of amifostine, while the lung and jejunum showed limited changes in response to amifostine treatment.

Herein, we extended the scope of the previous study to examine the radioprotective effects of amifostine in the heart, spleen, and kidney tissue in mice exposed to a lethal dose of total-body cobalt-60 (^60^Co) radiation. Furthermore, we examined both the transient and dose-dependent differences in metabolic profiles following administration of amifostine. Our results suggest that administration of amifostine prior to irradiation leads to metabolic alterations in tissue profiles that help to mitigate, or perhaps even to correct, radiation-induced alterations in biochemical pathways. In addition, we observed that the heart was most responsive to alleviation by amifostine of metabolically based radiation injury while the spleen showed modest changes followed by the kidney, which was the least responsive organ.

## 2. Results

### 2.1. Exposure to Gamma-Radiation-Induced Robust Changes in Tissue Metabolic Profiles

**Figure 1:**
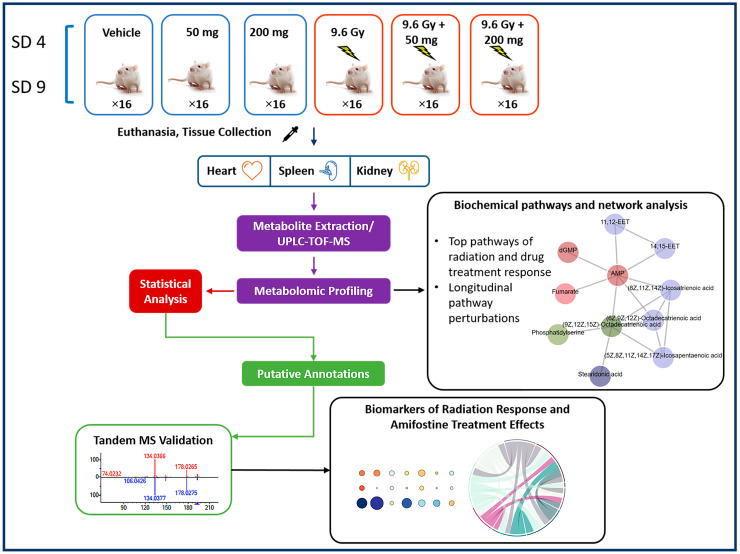
Experimental and analytical design of the study. Heart, spleen, and kidney samples were collected from irradiated and/or amifostine-treated mice at day (SD) 4 or 9 and prepared for untargeted LC-MS metabolomic profiling. Pathway and network analyses were performed by Mummichog 2. Putative annotation was done by an in-house CEU Mass Mediator RESTful API service, which has the capability to search Kegg, HMDB, LipidMaps, Metlin, PubChem and utilizing R packages “cmmr”. MS/MS validation was done by the TandemQuery tool (Li et al., unpublished) and NIST 2017 MS/MS spectra database.

**Figure 2:**
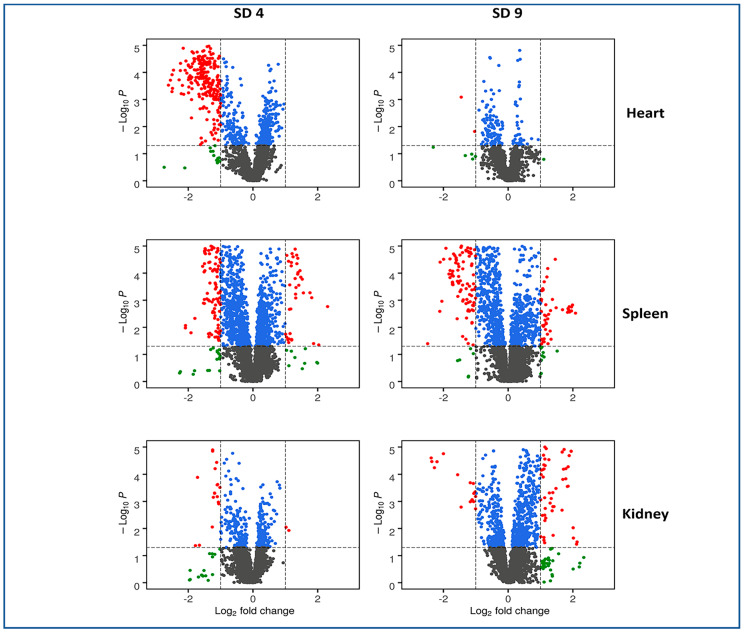
Volcano plots displaying dysregulated metabolites for heart, spleen, and kidney tissues at 4 and 9 days post-irradiation. In each plot, black dots represent metabolites that were not changed significantly, green dots represent metabolites with a significant fold change (<0.5 or >2), blue dots for metabolites with a significant *p* value (<0.05), and the red dots are used to annotate metabolites with a significant fold change as well as *p* value.

We utilized liquid chromatography–mass spectrometry (LC-MS)-based untargeted metabolomic approaches to investigate metabolic profiles and changes associated with exposure to ionizing radiation in heart, spleen, and kidney tissues (Figure 1). Preprocessing of LC-MS data yielded 5780 and 5064 numbers of features in the electrospray positive and negative modes, respectively. Radiation-induced dysregulation in metabolic profiles was visualized as volcano plots for all three tissue types at four and nine days post-irradiation (Figure 2). We found that the heart tissue showed robust radiation-induced significant dysregulation of metabolites four days post-irradiation. Furthermore, spleen tissue showed modest changes in metabolic profiles at both time points following irradiation while the kidney remained relatively recalcitrant with minimal changes in metabolic profiles at SD 4 and modest dysregulation at SD 9. The latter finding is consistent with previous reports that the kidney is a late-responding organ to radiation effects [26].

Statistical tests for early-stage (SD 4) and late-stage (SD 9) radiation effects (versus vehicle, Supplementary Table S2) and for high (200 mg) doses and low (50 mg) doses of amifostine at early and late time points were performed to determine drug effects on select metabolites (versus vehicle, Supplementary Table S4). Radiation-induced metabolic changes across all tissue types and metabolic corrections due to amifostine prophylaxis are listed in Supplementary Table S5. Specifically, at the early time point (SD 4), 19 out of 24 radiation-dysregulated metabolites in the heart were less altered (i.e., protected) by amifostine prophylaxis. Similarly, 25 out of 30 radiation-affected metabolites in the spleen were less altered/protected by amifostine, while 4 out of 7 metabolites that were altered by irradiation were less affected/protected by amifostine in mouse kidney tissue. However, the outcome at the later time point (SD 9) appeared different; in this case, 14 out of 24, 16 out of 30, and 4 out of 7 radiation-dysregulated metabolites were less affected/protected by amifostine prophylaxis in the heart, spleen, and kidney, respectively. A comprehensive list of validated metabolites with collision-induced dissociation (CID) fragmentation information is included in Supplementary Table S1. We observed changes in several classes of lipids including phosphatidylcholines, phosphatidylserines, phosphatidylinositols, and phosphatidylethanolamines [25,27]. We also observed changes in tissue levels of inflammatory mediators such as thromboxane A2 in the kidney tissue and hydroxyprostaglandin E1 in the heart tissue, amino acids such as l-glutamic acid in heart and spleen tissues, and l-aspartic acid in both the spleen and kidneys, as well as a multitude of fatty acids such as arachidonic acid and eicosapentaenoic acid (EPA) that play a vital role in bio-signaling pathways. Supplementary Table S2 includes a full list of assessed metabolites across all sampled tissues with their statistical *p* values, false discovery rate (FDR)-adjusted *p* value, and the corresponding fold change comparing the irradiated group of mice to the vehicle-treated group at SD 4 and SD 9.

**Figure 3:**
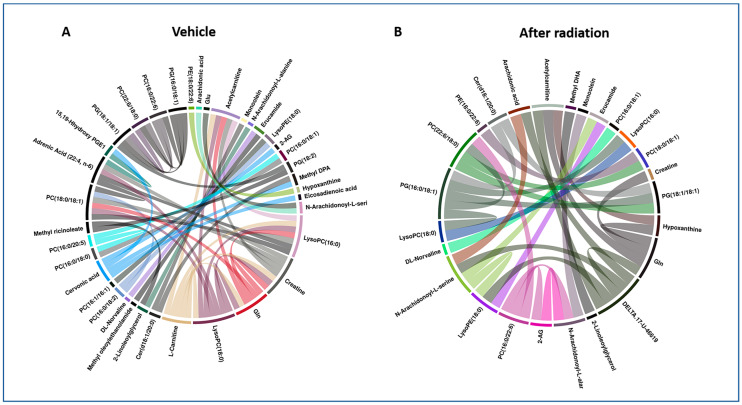
Circos plot showing correlations for annotated metabolites before (**A**) and after (**B**) radiation exposure at SD 4 for heart tissue. This figure illustrates the impact of ionizing radiation on dysregulation of metabolic profiles. Each band in the plot represents a statistical measure of the strength of a monotonic relationship between paired metabolites. Spearman correlation coefficient was set to a minimum of 0.5 and the *p* value < 0.01.

To further understand the nature and network of metabolic perturbations, we used circos plots to visualize these correlations (Figure 3, Supplementary Figures S2 and S3). These plots were constructed based on the peak intensities of dysregulated metabolites that were unambiguously identified using tandem mass spectrometry (MS), and they represent a statistical measure of the strength of a monotonic relationship between paired metabolites. The Spearman correlation coefficient was set to a minimum of 0.5 and the *p* value was less than 0.01. For heart tissue, creatinine, LysoPC (16:0), LysoPC (18:0), l-Carnitine, and cervonic acid were the most correlated metabolites among all tandem MS validated metabolites before radiation (Figure 3A). Some of the correlations, however, disappeared (l-Carnitine and cervonic acid) in the post-radiation circos plot (Figure 3B), perhaps suggesting that irradiation had disrupted these metabolic networks. For some metabolites, including creatinine, LysoPC (16:0), and LysoPC (18:0), correlations were preserved but decreased in significance. For the spleen, mannose 6-phosphate, omega-3 arachidonic acid, and PS (18:0/18:2) were the most correlated metabolites before radiation (Supplementary Figure S2A). Interestingly, the correlation between mannose 6-phosphate and omega-3 arachidonic acid was not disrupted by irradiation (Supplementary Figure S2B). For kidney tissue, PC (18:0/18:1), PC (18:0/22:6), EPA, and cervonic acid (Supplementary Figure S3A) were the most correlated metabolites among all tandem MS validated metabolites before radiation. Taken together, these analyses help understand the biological correlations between metabolites that offer novel insights into the mode of action of amifostine. All the metabolite correlations were significantly decreased post-irradiation (Supplementary Figure S3B).

In addition, we performed untargeted metabolomics pathway analysis using the Mummichog analysis Python package (Supplementary Table S3), which emphasized significant *p* value-related changes in numerous pathways such as the fatty acid biosynthesis, activation, and metabolism for select tissues (e.g., heart tissue). Similar analyses of spleen and kidney tissue metabolites revealed concurrent perturbations of several amino acid metabolic pathways including: valine, leucine, and isoleucine degradation and glycine, serine, alanine, and threonine metabolism.

### 2.2. Amifostine (50 and 200 mg/kg) Does Not Stimulate Major Metabolic Changes in Mice

Amifostine was shown to be nontoxic and effective in the treatment of xerostomia in patients receiving post-operative radiation regimens when administered at lower doses (50 mg/kg). Nevertheless, higher doses of amifostine (200 mg/kg) led to a number of adverse side effects in these clinical studies [28,29]. There is little understanding of the basic processes by which amifostine elicits these corrective metabolic changes within various types of tissues. Hence, we designed this study in an effort to understand the dose-dependent metabolic perturbations at 4 and 9 days post-irradiation in three distinct tissues of the body (heart, spleen, and kidney) with the overall goal of providing insights into drug toxicity at higher doses. We intended to examine the changes in the metabolic profiles generated by high and low doses of amifostine within these different tissue types. Further, the corrective effects of amifostine on the tissue metabolic profiles at SD 4 and SD 9 after irradiation were assembled and analyzed in detail. A comprehensive list of all identified metabolites that changed significantly (as defined by *p* values) for each tissue type at the tested dose strengths and at the specified time points is displayed in Supplementary Table S4A–E. While there were a few significant changes in a select number of metabolites for both drug doses at SD 4, after adjusting the FDR for multiple hypothesis testing, the results did not show any significant differences. More metabolites were identified to be *p* value significant for both doses, but the FDR adjustment resulted in no significant difference for both doses of the drug. The only exception to the observed trend was PC (P-18:0/20:4), which was significantly reduced after administering the higher drug dose at SD 9. Taken together, these results suggest that the drug did not induce deleterious metabolic perturbations in mice at both doses tested in this study.

### 2.3. Administration of Amifostine Partially Corrects Metabolic Perturbations Caused by Ionizing Radiation in Mice Heart, Spleen, and Kidney Tissues

**Figure 4:**
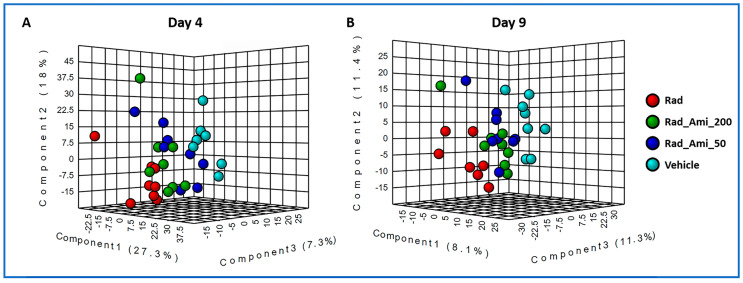
A three-dimensional PLS-DA plot showing separation for study groups vehicle only, radiation only, amifostine 50 mg + radiation, and amifostine 200 mg + radiation based on metabolic profiles of heart at day 4 (**A**) and day 9 (**B**) post-irradiation, negative mode.

**Figure 5:**
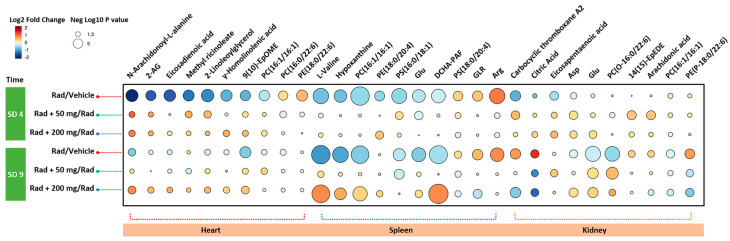
Raindrop plot demonstrating amifostine protective effects on a representative subset of the validated metabolites after radiation exposure in heart, spleen, and kidney tissues after four (SD 4) and nine (SD 9) days post-irradiation.

Next, we asked if treatment with amifostine prior to irradiation would help alleviate radiation-induced perturbations of the metabolic profiles of the sampled tissues of interest (i.e., the heart, spleen, and kidney). We used partial least squares–discriminant analysis (PLS-DA) to examine changes in metabolic profiles in the sampled tissues. Specifically and based on metabolic profiles at day 4 and at day 9, we compared the control group of mice (receiving vehicle only) with those that received either radiation, amifostine 50 mg/kg + radiation, and/or amifostine 200 mg/kg + radiation (Figure 4). We performed these comparative analyses for each tissue type at four and nine days post-irradiation. As annotated in Supplementary Table S5, the heart tissue as well as the spleen tissue showed the most corrective responses at both doses of amifostine. On the other hand, the kidney tissue demonstrated lower levels of recovery compared to the other two tissue types as evidenced by the significant raw *p* value but not the FDR-adjusted *p* value. Figure 5 displays a raindrop plot demonstrating amifostine’s corrective effects on a representative subset of the validated metabolites after radiation exposure in heart, spleen, and kidney tissues at SD 4 and SD 9 for both tested doses. The suppressed levels of several metabolites appeared to be recovered in a drug-dose-dependent fashion within heart tissue as a result of amifostine prophylaxis (e.g., N-arachidonoyl-l-alanine, 2-AG, and eicosadienoic acid were lowered by ionizing radiation, but increased following amifostine administration).

We also performed Mummichog pathway analysis that indicated minimal metabolic pathway changes in the heart tissue at both time points at the lower dose of the drug. However, with the higher dose of amifostine and at SD 9, some pathways changed demonstratively by way of the synthesis, activation, and biotransformation of fatty acids. At SD 4, spleen tissue showed alterations in the pathways pertaining to amino acid metabolism as well as purine and pyrimidine metabolism. The kidney tissue demonstrated few changes in amino acid metabolism pathways, glycophospholipid metabolism, and linoleate metabolism pathways at SD 4 when using the lower dose of the drug. Surprisingly, the kidney tissue at the higher dose of amifostine showed more pronounced changes in amino acid metabolism as well as in the purine/pyrimidine metabolism pathways at SD 4, as compared to SD 9. Supplementary Table S6 shows all pathway analyses for the designated tissue types for both doses at SD 4 and SD 9.

## 3. Discussion

Since its FDA approval in 1995, amifostine (Ethyol^®^) has been successfully used to reduce the nephrotoxic effects caused by repeated treatment with cisplatin for advanced ovarian cancer and to mitigate xerostomia in some patients undergoing radiotherapy procedures for cancers of the head and neck [3,22,30]. Several serious, drug-dose-dependent, adverse side effects have been reported in humans [3,31,32,33]. A study by Pandit et al. examined the metabolic rate by gas exchange in six healthy subjects and concluded that amifostine protects normal cells from toxic effects of chemotherapy by reducing metabolic rate [34]. In another study, Koukourakis et al. used human and mouse models to study the radioprotective effects of amifostine using calorimetric canopy and found reduction in oxygen consumption rates in cancer patients receiving amifostine [35].

In order to better understand amifostine’s radioprotective and toxic properties at various levels of administered drug doses, we used a lower dose of 50 mg/kg and compared it with a higher dose of 200 mg/kg while applying a global metabolomic tissue profiling approach. This was done in order to better understand the irradiation-elicited responses of normal tissues at the metabolic level, as well as the potential corrective actions (on various metabolic networks) of the radioprotective drug. We examined a total of 288 tissue samples of heart (N = 96), spleen (N = 96), and kidney (N = 96) obtained from mice that were either irradiated with 9.6 Gy γ-radiation or sham irradiated. Mice were administered amifostine 50 mg/kg (N = 16), 200 mg/kg, or were treated with vehicle (saline). Tissues were harvested on day 4 or day 9 post-irradiation or post-drug administration. We selected the day 4 post-irradiation time point as it is comparable to the symptom-free latent phase of humans following the prodromal phase. Day 4 post-irradiation for mice is equivalent to the illness phase presenting clinical symptoms.

The results presented served to reconfirm the ‘survival benefit’ of administering amifostine prophylactically to lethally irradiated animals and that the degree of this ‘benefit’ was drug-dose-dependent. In terms of the primary metabolic analyses made here in this study, differential responses of three different types of tissues (heart, spleen, and kidney) were noted following acute irradiation. We observed changes in tissue profiles across several classes of lipids including phosphatidylcholines, phosphatidylserines, phosphatidylinositols, and phosphatidylethanolamines [25,27]. We also observed changes in tissue levels of inflammatory mediators such as thromboxane A2 in the kidney tissue, hydroxyprostaglandin E1 in the heart tissue, various amino acids (l-glutamic acid and l-aspartic acid) in both spleen and kidneys, and a multitude of fatty acids that play vital roles in bio-signaling pathways (e.g., arachidonic acid and EPA). These metabolites have important functional and regulatory roles in biological systems and can help explain the initiation and progression of acute and long-term tissue injury occurring after exposure to ionizing radiation. Supplementary Table S2 includes a full list of metabolites across all tested tissues with their statistical *p* values, FDR-adjusted *p* value, and the corresponding fold change comparing the irradiated group of mice to the vehicle-treated group at SD 4 and SD 9.

Of the three tissues sampled, heart tissue was the most susceptible to metabolic alterations early (SD 4) following acute irradiation, as indicated by the change in a number of affected metabolites (Figure 2B and Supplementary Table S2). By contrast, these radiation-altering effects appeared to be minimized at the latter time point (SD 9). These affected metabolites included DHA, adrenic acid, γ-homolinolenic acid, 2-arachidonoylglycerol, 2-linoleoylglycerol, and N-arachidonoyl-l-alanine. It has been reported that oxidative stress caused by ionizing radiation results in a reduction in the levels of key fatty acids [36]. Additionally, the spleen showed an increase in proinflammatory metabolites including ω-3 arachidonic acid and arachidonic acid which was more prominently elevated at SD 9 post-irradiation. Several amino acids were found to be lowered especially at SD 9 including valine and glutamic and aspartic acids. Meanwhile, l-arginine was elevated at both time points. Interestingly, elevation in l-arginine has been shown to protect hematopoietic progenitors [37]. Furthermore, the level of oxidized glutathione (GSSG) was lowered significantly at SD 9 which could be due to reduced splenic volume, along with increased rates of apoptosis [38]. By contrast, kidney tissues showed rather minor metabolic alterations in response to irradiation and minimal alleviation with amifostine administration at both time points tested. However, phenaceturic acid and PS (18:0/0:0) were found to be significantly elevated at SD 9 based on the corrected *p* value. Furthermore, we sought to explain the noted correlations via circos plots between identified metabolites within the irradiated and vehicle-treated groups at SD 4 (Figure 3 and Supplementary Figures S2 and S3). For these correlations, the Spearman correlation coefficient was set to a minimum of 0.5 and the *p* value was less than 0.01. For example, in panel A, there was a clear correlation between arachidonic acid and N-arachidonoyl-l-serine as well as glutamic acid. This was clearly documented previously in the literature [39]. Another example includes the correlation between glutamine and creatinine seen in panel B.

We further examined the overall countering effects of amifostine at both drug doses on the radiation-altered metabolic profiles. In this regard, we found that levels of several metabolites recovered within the heart tissue following amifostine administration, and the extent of those noted drug-elicited responses appeared drug-dose-dependent. Tissue levels of N-arachidonoyl-l-alanine, 2-AG, and eicosadienoic acid were all reduced by ionizing radiation. However, following graded drug dosing with amifostine, normal tissue levels of these metabolites were noted.

Surprisingly, the tissue levels of l-valine, glutamic acid, and hypoxanthine were lowered by the exposure to ionizing radiation. However, this lowering of metabolite levels was mitigated by amifostine prophylaxis. On the other hand, some metabolites like PS (18:0/20:4) and l-arginine that were initially shown to be elevated following ionizing irradiation were corrected by amifostine in a dose-dependent way. Another key inflammatory mediator that was significantly elevated after radiation exposure, but was corrected by amifostine, was carbocyclic thromboxane A2 [40,41]. Kidney tissues showed minimal signs of metabolic dysregulation. Interestingly, we observed that the levels of citric acid, EPA, and arachidonic acid were all reduced after radiation exposure at SD 4 but elevated at SD 9. In both cases, pretreatment with amifostine helped restore normal tissue levels of these metabolites. In summary, these changes suggest perhaps dysregulated mitochondrial functions in response to radiation that seems to be corrected at least in part by treatment with amifostine.

## 4. Materials and Methods

### 4.1. Mice

Six- to eight-week-old male CD2F1 mice were procured from Envigo, Indianapolis, IN, USA and housed in a controlled-environment vivarium accredited by the Association for Assessment and Accreditation of Laboratory Animal Care International. Mice were quarantined and a representative sample was examined for bacterial infections and the assured absence of *Pseudomonas aeruginosa* [25]. All animal procedures discussed for this study were conducted based on protocol number P-2017-08-009 approved by the Institutional Animal Care and Use Committee. The study was performed in accordance with the Guide for the Care and Use of Laboratory Animals, the Institute of Laboratory Animal Resources, National Research Council, United States National Academy of Sciences [42].

### 4.2. Experimental Design

A total of six groups were used in this study and in each group there were 16 mice, a number that provided for high statistical power for metabolomics data [25]. Two different doses of amifostine, 50 and 200 mg/kg, were used with and without irradiation. There were two control groups: vehicle (saline) without irradiation and the vehicle (saline) with irradiation. Animals were administered amifostine 30 min (± 10 min) before irradiation. There were four animals in each cage and each was identified with 1, 2, 3, or 4 bands marked on the tail. Tissue samples for metabolomic/lipidomic analysis were collected from serially sacrificed mice on days 4 and 9 post-irradiation (eight animals each on day 4 and day 9). Parallel to this study, there were three additional groups of irradiated animals used for survival analyses: one vehicle control, one pretreated with 50 mg/kg amifostine, and one with 200 mg/kg amifostine. The treatment schedule is illustrated in the study design (Figure 1).

### 4.3. Drug Administration to Mice

Pharmaceutical-grade amifostine (Ethyol^®^) was procured for use from Cumberland Pharmaceuticals (Nashville, TN, USA) as sterile lyophilized powder vials of 500 mg. Such vials of amifostine were reconstituted with normal saline before use and administered subcutaneously in 0.1 mL volume at the nape of the neck of mice. Subcutaneous/intramuscular route of administration is the most suitable for field use of any radiation countermeasure. Control group animals not receiving amifostine received equal volume of normal saline.

### 4.4. Radiation Exposure

Radiation dosimetry was based on the alanine/electron paramagnetic resonance (EPR) system [43]. Mice were exposed acutely to whole-body 9.6 Gy of ^60^Co γ-radiation at a dose rate of 0.6 Gy/min. The delivered dose of irradiation equated to LD_90/30_ exposures; the LD_50/30_ dose for the CD2F1 strain of male mice is estimated at 8.6 Gy [25]. Following irradiation, the exposed (or sham exposed) mice were returned to their respective cages and subsequently monitored until samples were collected.

### 4.5. Collection of Tissue Samples

Mice tissue samples were collected on days 4 and 9 post-irradiation. Animals were anesthetized using isoflurane (1–5%, Abbott Laboratories, Chicago, IL, USA) and then euthanized. From each euthanized mouse, the heart, kidneys, and spleen were collected and immediately frozen in liquid nitrogen and stored at −80 °C until used.

### 4.6. Tissue Metabolomic Profile Analysis Utilizing UPLC-QTOF Mass Spectrometry

Tissue samples (5 mg each) were homogenized on ice in a volume of 150 μL of extracting solution mixture comprising 35% water, 25% methanol, and 40% isopropanol containing the internal standards (debrisoquine and 4-nitrobenzoic acid) and extracted as described previously [25]. The quality control (QC) sample for each tissue type consisted of a pooled aliquot of each respective tissue type, thus representing all metabolites in each matrix. The column was conditioned using the pooled QC sample and was injected every 10 samples to observe mass accuracy, shifts in retention time, and variations in signal intensities to monitor reproducibility and data quality [44]. The overlap of QC sample chromatograms (base peak intensity) shows minimal shifts in retention time and consistency in peak intensities throughout the acquisition (detailed in Supplementary Figure S1).

### 4.7. Statistical Analysis of Metabolomics Data

For analysis of untargeted metabolomics data, raw MS data files were converted to NetCDF format using the MassLynx Software (Waters Corporation, USA). NetCDF files were then processed using an in-house implementation of the XCMS (Scripps Institute, La Jolla, CA, USA) R package for peak detection and retention time correction. Initially, the ion peaks were filtered and detected using the matched filter algorithm. The peak detection algorithm allows data to be binned into parts with predefined widths and mass; it is then compared to known peaks of similar distributions. The Ordered Bijective Interpolated Warping (OBI-Warp) algorithm was applied for retention time correction [45]. All parameters for the matched filter and OBI-Warp algorithm were optimized by IPO (Isotopologue Parameter Optimization) R package [46]. During the preprocessing procedure, the features (with a combination of retention time and mass-to-charge ratio) in both positive and negative ionization modes were initially normalized to the internal standards and then by probabilistic quotient normalization (PQN) to eliminate the concentration variance [47]. PQN was originally used for 1H-NMR data normalization and then proved to be robust and effective in UPLC-QTOF MS data as well [48]. Database search for putative metabolite identifications was performed using an in-house CEU Mass Mediator (CMM), which searched KEGG [49], HMDB [50], LIPID MAPS [51], METLIN [52], and PubChem [53]. All normalized features were log-transformed and Pareto-scaled. The total number of samples was 96 in this study; the level of differential expression for each metabolite was calculated using an unpaired t-test, comparing vehicle versus amifostine 50 mg/kg (side effect of drug), vehicle versus amifostine 200 mg/kg (side effect of drug), vehicle and radiation (effect of radiation), radiation vs. radiation + amifostine 50 mg/kg or 200 mg/kg (effect of drug), constrained by FDR-adjusted *p* value < 0.05. The metabolic pathways analysis to assess effects of irradiation and amifostine administration was done by Mummichog v2.0, a Python package specifically designed for untargeted metabolomics [54,55,56,57].

## 5. Conclusions

While administration of amifostine alone did not result in major metabolic perturbations in mice at either of the drug doses tested, pretreatment with amifostine at the higher, survival-sparing dose of 200 mg/kg did appear to trigger a significant correction of certain metabolic responses within select tissues of acutely irradiated animals. Heart tissue appeared particularly responsive, while the spleen was only modestly responsive and the kidney even less so. By contrast, treatment with the lower dose of amifostine (50 mg/kg) appeared only mitigative, but not entirely corrective in terms of the radiation-induced dysfunctions of multiple metabolic pathways. Finally, noted corrective/recovery processes within the different tissues appeared to differ, with recovery within heart tissue appearing faster than in spleen or kidney tissue.

## Supplementary Materials

The following are available online at https://www.mdpi.com/2218-1989/10/5/211/s1, Supplementary Table S1, Supplementary Table S2, Supplementary Table S3, Supplementary Table S4, Supplementary Table S5, Supplementary Table S6, Supplementary Figure S1 (Panels A-C), Supplementary Figure S2 (Panels A-B), Supplementary Figure S3 (Panels A-B).
