# Supplementary material for: Alterations in Tissue Metabolite Profiles with Amifostine-Prophylaxed Mice Exposed to Gamma Radiation
Source: Metabolites. 2020 May 21;10(5):211. doi: 10.3390/metabo10050211 (PMC7281564; doi:10.3390/metabo10050211)
Supplement: Supplementary file 1 [file metabolites-10-00211-s001.pdf]

# Supplementary Figure 1

A

| Name | Initial<br>POS | <br>INPUT | <br>PPM error |
|---|---|---|---|
| Acetaminophen | 152.0712 | 152.0713 | 0.65 |
| Sulfaguanidine | 215.0603 | 215.0607 | 1.85 |
| Sulfadimethoxine | 311.0814 | 311.0815 | 0.32 |
| Val-Tyr-Val | 380.2185 | 380.2183 | -0.52 |
| Terfenadine | 472.3216 | 472.3212 | -0.84 |
| Leu-Enkephalin | 556.2771 | 556.2778 | 1.25 |

| Name | Final<br>POS | <br>INPUT | <br>PPM error |
|---|---|---|---|
| Acetaminophen | 152.0712 | 152.0713 | 0.65 |
| Sulfaguanidine | 215.0603 | 215.0608 | 2.32 |
| Sulfadimethoxine | 311.0814 | 311.0809 | -1.6 |
| Val-Tyr-Val | 380.2185 | 380.2185 | 0 |
| Terfenadine | 472.3216 | 472.321 | -1.27 |
| Leu-Enkephalin | 556.2771 | 556.2766 | -0.89 |

B

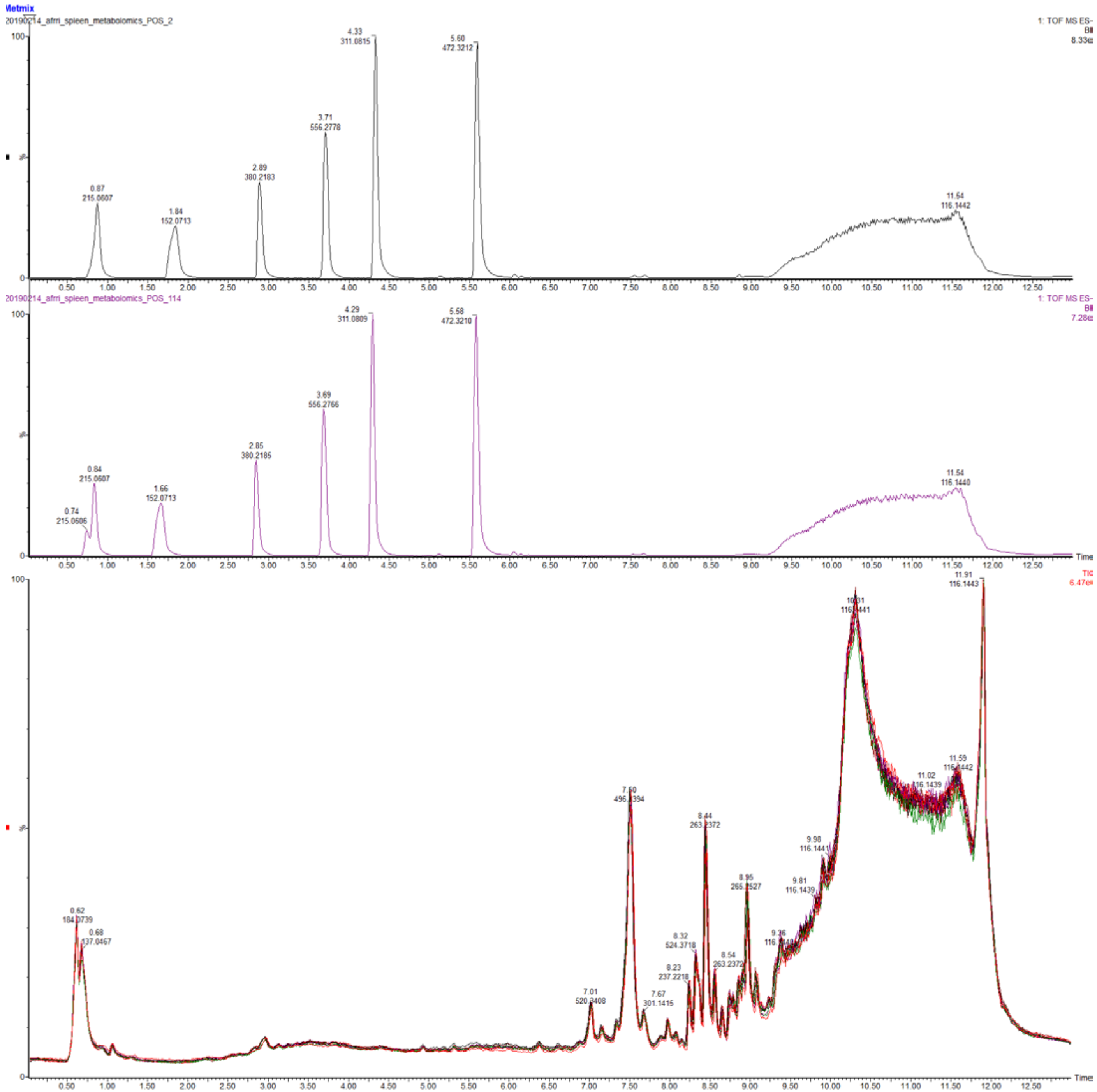

**Supplementary Figure 1: A)** Mass accuracy checks were performed at the beginning and end of the batch using a mix of 6 standard compounds. The ppm mass accuracy checks for these 6 compounds were all within 5 ppm for both positive and negative ionization modes. **B)** Chromatograms of the mass accuracy checks at the beginning (top) and the end (bottom) of a batch. **C)** Overlay of all pooled QC samples throughout a batch show minimum shifts in retention times and intensities.

Supplementary Figure 2

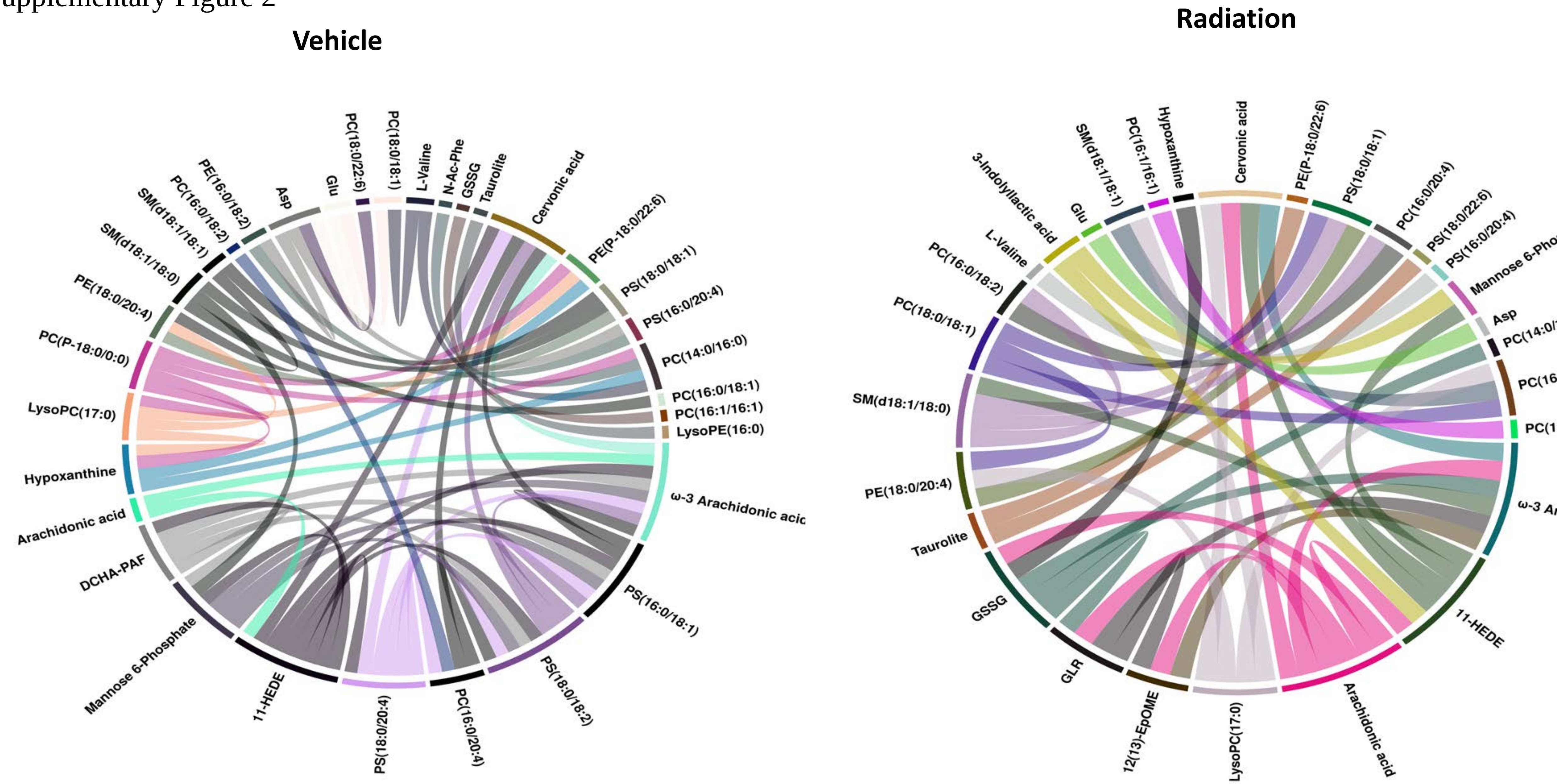

**Supplementary Figure 2: Circos plot for identified metabolites and their correlations before (Panel A) and after (Panel B) radiation expose at SD4 in spleen tissue.** This figure illustrates the dysregulating impact of ionizing radiation on the metabolic profiles. Each band in the plot represents a statistical measure of the strength of a monotonic relationship between paired metabolites. Spearman correlation coefficient was set to a minimum of 0.5 and the p value $< 0.01$.

Supplementary Figure 3

Vehicle

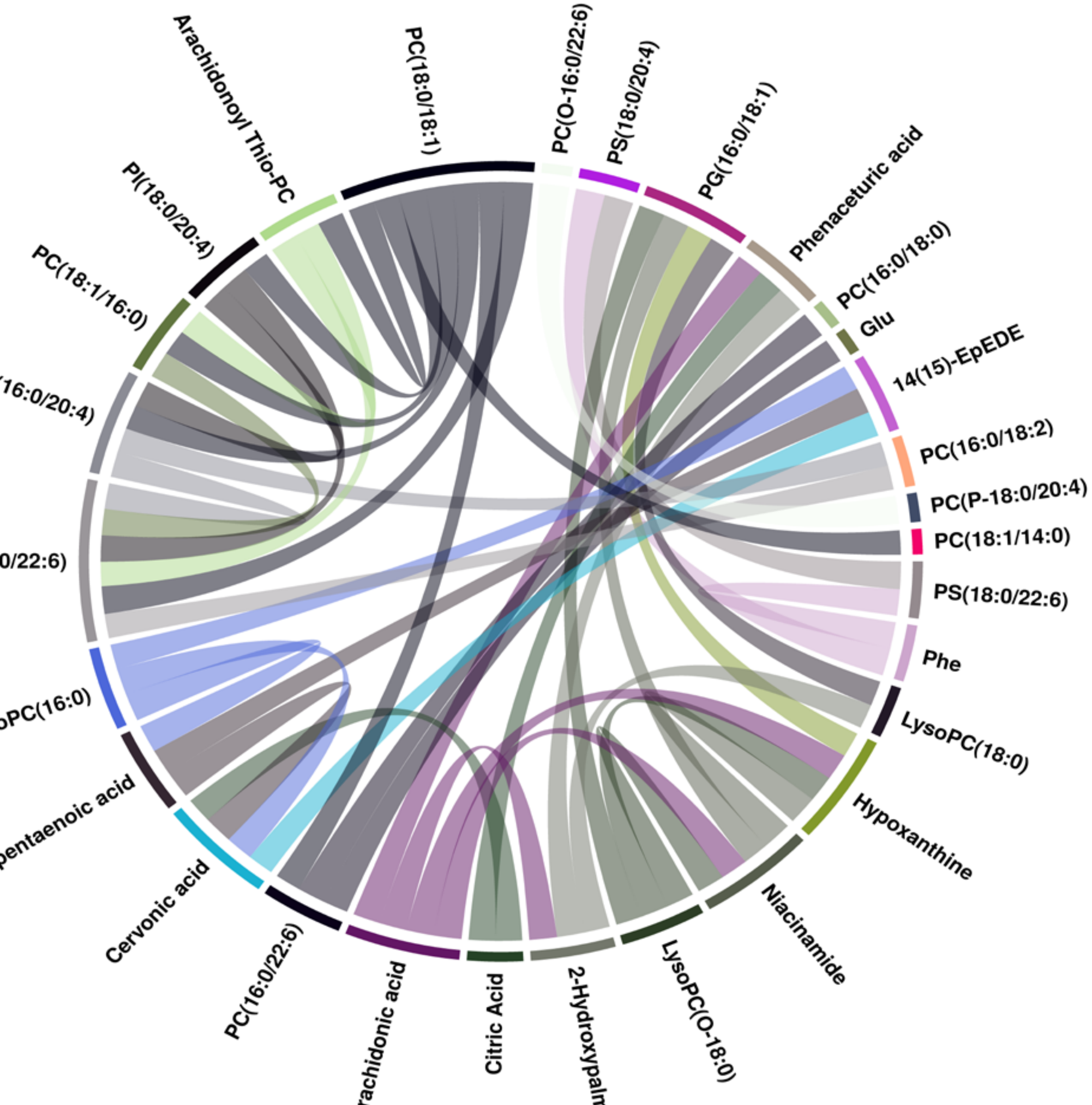


Radiation

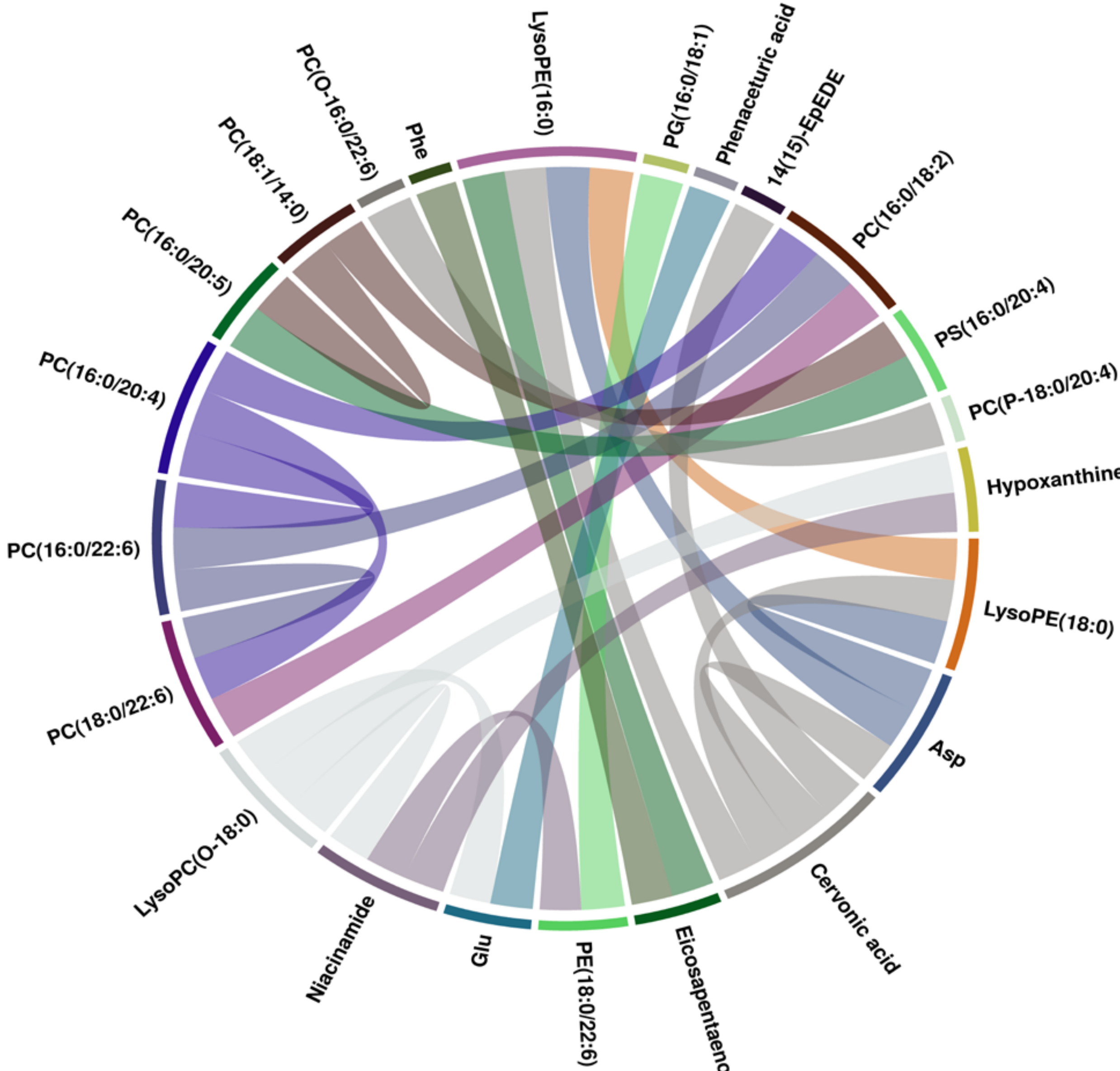

**Supplementary Figure 3: Circos plot for identified metabolites and their correlations before (Panel A) and after (Panel B) radiation expose at SD4 for kidney tissue.** This figure illustrates the dysregulating impact of ionizing radiation on the metabolic profiles. Each band in the plot represents a statistical measure of the strength of a monotonic relationship between paired metabolites. Spearman correlation coefficient was set to a minimum of 0.5 and the p value $< 0.01$.

## Supplementary Table 1. Table of Tandem MS Validated Metabolites

| Name | Ionization Mode | Matrix | precursor mz | rt | Synonyms | Adduct | Level | CID | Class | |
|---|---|---|---|---|---|---|---|---|---|---|
| N-(1-Oxo-5Z,8Z,11Z,14Z-eicosatetraenyl)-L-alanine | Positive | Heart | 375.2612 | 7.63 | N-Arachidonoyl-L-alanine | [M+H]+ | 1 | 375.26, 287.26, 269.22, 245.22, 213.16, 203.17, 185.13, 171.11, 147.11, 131.1, 119.08, 105.06, 95.08, 81.06 | N-Acyl Amino Acids | |
| 1-Glyceryl oleate | Positive | Heart | 357.2772 | 8.9 | Monoolein | [M+H]+ | 1 | 357.29, 339.29, 265.25, 177.16, 149.13, 135.11, 121.1, 109.1, 95.08, 81.07, 69.07 | Monoglycerides | |
| DL-2-Aminovaleric acid | Positive | Heart | 118.0874 | 0.43 | DL-Norvaline | [M+H]+ | 1 | 118.08, 72.08 | Amino Acids | |
| L-Glutamine | Positive | Heart | 147.0776 | 0.44 | Gln | [M+H]+ | 1 | 147.06, 130.06, 84.04 | Amino Acids | |
| cis-5,8,11,14-Eicosatetraenoic acid | Positive | Heart | 305.2484 | 8.02 | Arachidonic acid | [M+H]+ | 1 | 305.24, 287.23, 269.22, 245.22, 221.15, 177.16, 147.11, 121.1, 95.08 | Fatty Acids And Derivatives | |
| (3R)-3-Hydroxy-4-(trimethylammonio)butanoate | Positive | Heart | 162.1136 | 0.41 | L-Carnitine | [M+H]+ | 1 | 162.11, 103.03, 85.08, 60.08 | Amino Acids | |
| (alpha-Methylguanido)acetic acid | Positive | Heart | 132.0778 | 0.42 | Creatine | [M+H]+ | 1 | 132.07, 114.06, 90.05 | Amino Acidds | |
| L-Glutamic acid | Positive | Heart | 148.0049 | 0.43 | Glu | [M+H]+ | 1 | 148.00, 130.05, 102.05, 84.04 | Amino Acids | |
| 1-Stearoyl-2-hydroxy-sn-glycero-3-phosphocholine | Positive | Heart | 524.3713 | 7.46 | LysoPC(18:0) | [M+H]+ | 1 | 524.37, 306.36, 341.3, 184.07, 104.1 | Phosphatidylchlines | |
| Docosahexaenoic acid methyl ester | Positive | Heart | 342.203 | 9.11 | Methyl DHA | [M+2H]+ | 1 | 345.2787,344.2668,343.2641,311.2384,301.2101,161.1316,145.1016,133.1013,131.0855,121.1017,119.0858,107.0856,105.07,95.0859,93.07 | Fatty Acids And Derivatives | |
| Adenosine 5'-monophosphate | Positive | Heart | 348.0715 | 0.45 | 5'-Adenylic acid | [M+H]+ | 2 | 348.07, 137.04, 136.06119.03, 97.02 | Nucleoside Monophosphate | |
| 1-Palmitoyl-sn-glycero-3-phosphocholine | Positive | Heart | 991.6712 | 6.71 | LysoPC(16:0) | [2M+H]+ | 2 | 991.67, 590.32, 496.34, 184.07, 104.08 | Lysophosphatidylcholine | |
| 1-Stearoyl-2-hydroxy-sn-glycero-3-phosphoethanolamine | Positive | Heart | 482.3248 | 7.43 | LysoPE(18:0) | [M+H]+ | 2 | 482.32, 464.31, 421.27, 341.3, 310.31, | Lysophosphatidylethanolamine | |
| N-(Eicosanoyl)sphingosine | Positive | Heart | 575.5029 | 10.8 | Cer(d18:1/20:0) | [M+H-H2O]+ | 2 | 575.5, 282.22, 264.27, 262.25, 252.26, | Ceramides | |
| N-[(1R)-2-Hydroxy-1-methylethyl-9Z-octadecenamide | Positive | Heart | 323.2528 | 8.15 | Methyl oleoylethanolamide | [M+H-H2O]+ | 2 | 323.25, 263.23, 245.22, 191.17, 177.2, 163.2, 149.132, 135.17, 121.05, 109.1, 97.1, | N-Acyl Amino Acids | |
| cis-4,7,10,13,16,19-Docosahexaenoic acid | Positive | Heart | 329.2477 | 7.48 | Cervonic acid | [N+H]+ | 2 | 329.24, 293.22, 269.22, 247.16, 215.17, 201.16, 175.14, 159.11, 145.1, 133.1, 119.08, 107.08, 96.08, 81.06 | Fatty Acids And Derivatives | |
| Docosapentaenoic acidmethyl ester | Positive | Heart | 344.2587 | 8.67 | Methyl DPA | [M+2H]+ | 2 | 344.25, 311.23, 293.232, 287.23, 283.21, 273.19, 269.22, 255.17, 261.18, 241.16, 215.16, 201.16, 175.14, 161.13, 133.1, 93.07, 67.05 | Fatty Acids And Derivatives | |
| 8Z,14Z-Eicosadienoic acid | Positive | Heart | 291.2665 | 8.27 | Eicosadienoic acid | [M+H-H2O]+ | 2 | 291.26, 147.11, 123.11, 109.1, 95.08, 81.07, 67.05 | Fatty Acids And Derivatives | |
| Ricinoleic acid methyl ester | Positive | Heart | 313.2739 | 8.01 | Methyl ricinoleate | [M+H]+ | 2 | 313.27, 295.26, 263.23, 245.22 | Fatty Acids And Derivatives | |
| cis-8,11,14-Eicosatrienoic acid | Positive | Heart | 307.2588 | 8.32 | γ-Homolinolenic acid | [M+H]+ | 2 | 307.25, 289.25, 209.14, 173.13, 109.1, 96.08, 81.07 | Fatty Acids And Derivatives | |
| 2-Arachidonoylglycerol | Positive | Heart | 379.2825 | 8.14 | 2-AG | [M+H]+ | 2 | 379.28, 305.24, 287.23, 269.22, 245.22, 203.17 | Monoglycerides | |
| 1-Palmitoyl-2-hydroxy-sn-glycero-3-phosphoethanolamine | Positive | Heart | 454.3896 | 6.9 | LysoPE(16:0) | [M+H]+ | 2 | 454.38, 313.27, 155.02, 85.02 | Lysophosphatidylethanolamine | |
| cis-7,10,13,16-Docosatetraenoic acid | Positive | Heart | 333.2791 | 8.5 | Adrenic Acid (22:4, n-6) | [M+H]+ | 2 | 33.27, 249.18, 205.19, 135.11, 121.1 | Fatty Acids And Derivatives | |
| 13-Docosenamide, (Z)- | Positive | Heart | 338.1829 | 8.61 | Erucamide | [M+H]+ | 2 | 338.18, 303.3, 163.14, 135.1, 121.1, 109.1, 95.08, 81.07 | Fatty Acids And Derivatives | |
| 5-Heptenoic acid, 7-[(1R,4S,5S,6R)-6-[(1E,,3S,5Z)-3-hydroxy-1,5-octadien-1-yl]-2-oxabicyclo[2.2.1]hept-5-yl]-, (5Z)- | Positive | Heart | 331.2637 | 8.11 | DELTA.17-U-46619 | [M+H-H2O]+ | 2 | 331.26, 239.14, 135.11, 121.1, 109.1, 95.08, 81.08 | Prostaglandins And Derivatives | |
| 1-Hexadecanoyl-2-octadecadienoyl-sn-glycero-3-phosphocholine | Positive | Heart | 758.5699 | 9.87 | PC(16:0/18:2) | [M+H]+ | 2 | 758.56, 184.07, 86.09 | Phosphatidylcholine | |
| 1,2-Dipalmitoleoyl-sn-glycero-3-phosphocholine | Positive | Heart | 730.5382 | 9.74 | PC(16:1/16:1) | [M+H]+ | 2 | 730.53, 184.07, 86.09 | Phosphatidylcholine | |
| 1-Palmitoyl-2-oleoyl-phosphatidylglycerol | Positive | Heart | 771.5146 | 11.04 | PG(16:0/18:1) | [M+Na]+ | 2 | 771.52, 577.2, 195.08, 67.05 | Phosphatidylglycerol | |
| 1,2-Dilinoleoyl-sn-glycero-3-phospho-(1'-rac-glycerol) | Positive | Heart | 793.5039 | 11.59 | PG(18:2) | [M+Na]+ | 2 | 793.5, 195.08 | Diglycerides | |
| 1,2-Dioleoyl-sn-glycero-3-phospho-rac-1-glycerol | Positive | Heart | 797.5311 | 11.06 | PG(18:1/18:1) | [M+Na]+ | 2 | 797.53, 195.00 | Diglycerides | |
| N-[1-Oxo-5Z,8Z,11Z,14Z-eicosatetraenyl]-L-serine | Positive | Heart | 287.2357 | 8.02 | N-Arachidonoyl-L-serine | [M+H-C3H7O3N]+ | 2 | 287.23, 269.22, 203.17, 171.11, 148.97, 131.04, 119.08 | N-Acyl Amino Acids | |
| 1-Octadecanoyl-2-octadecenoyl-sn-glycero-3-phosphocholine | Positive | Heart | 810.5073 | 9.69 | PC(18:0/18:1) | [M+Na]+ | 2 | 810.5, 184.07 | Phosphatidylchlines | |
| Arachidonoylthiophosphorylcholine | Positive | Heart | 784.5823 | 9.86 | Arachidonoyl Thio-PC | [M+H]+ | 2 | 784.56, 184.07 | Thiophospholipids | |
| 2-Docosahexaenoyl-1-stearoyl-sn-glycero-3-phosphoethanolamine | Positive | Heart | 792.5703 | 9.89 | PE(18:0/22:6) | [M+H]+ | 2 | 792.57, 651.53 | Phosphatidylethanolamine | |
| 6H-Purin-6-one, 1,7-dihydro- | Positive | Heart | 137.047 | 0.47 | Hypoxanthine | [M+H]+ | 2 | 137.04, 119.03, 110.03, 82.03 | Purine Derivative | Might indicate DNA damage |
| 7α,12α-Dihydroxycholest-4-en-3-one | Positive | Heart | 400.2676 | 7.69 | 7α,12α-Dihydroxycholest-4-en-3-one | [M+H-H2O]+ | 3 | 400.28, 269.22, 227.17, 185.12, 159.11, 131.07, 93.06 | 7Alpha-Hydroxy Steroid | |
| 9(10)-Epoxy-12Z-octadecenoic acid | Positive | Heart | 279.2321 | 7.69 | 9(10)-EpOME | [M+H-H2O]+ | 3 | 279.23, 173.13, 149.13, 135.16, 123.18, 104.1 | Fatty Acids And Derivatives | |
| 15(R),19(R)-Hydroxyprostaglandin E1 | Positive | Heart | 353.2668 | 8.12 | 15,19-Hhydroxy PGE1 | [M+H-H2O]+ | 3 | 353.26, 263.23, 245.22, 121.1, 95.08, 81.08 | Prostaglandins And Derivatives | |
| 1-Palmitoyl-2-stearoyl-sn-glycero-3-phosphocholine | Positive | Heart | 762.5126 | 9.67 | PC(16:0/18:0) | [M+H]+ | 3 | 762.51, 184.07 | Phosphatidylchlines | |
| 9Z,12Z-Octadecadienoic acid, 2-glyceryl ester⍰ | Positive | Heart | 355.2837 | 7.63 | 2-Linoleoylglycerol | [M+H]+ | 1 | 355.28, 337.27, 263.23, 245.22, 189.16, 175.14, 149.13, 135.11, 121.1, 95.08, 81.06 | Monoglycerides | |
| 1-Propanaminium, 2-(acetyloxy)-3-carboxy-N,N,N-trimethyl-, inner salt | Positive | Heart | 204.1241 | 0.44 | Acetylcarnitine | [M+H]+ | 1 | 204.13, 145.04, 85.02 | Miscellaneous | |
| Palmitoyleicosapentaenoyl phosphatidylcholine | Positive | Heart | 780.5537 | 9.7 | PC(16:0/20:5) | [M+H]+ | 2 | 780.55, 184.07 | Phosphatidylcholine | |
| 2-Oleoyl-1-palmitoyl-sn-glycero-3-phosphocholine | Positive | Heart | 760.5854 | 9.96 | PC(16:0/18:1) | [M+H]+ | 2 | 760.58, 184.07 | Phosphatidylcholine | |
| 1-Docosahexaenoyl-2-stearoyl-sn-glycero-3-phosphocholine | Positive | Heart | 834.6007 | 9.89 | PC(22:6/18:0) | [M+H]+ | 2 | 834.6, 184.07 | Phosphatidylcholine | |
| 1-Palmitoyl-2-docosahexaenoyl-sn-glycero-3-phosphocholine | Positive | Heart | 806.5694 | 9.78 | PC(16:0/22:6) | [M+H]+ | 2 | 806.56, 184.07 | Phosphatidylcholine | |
| 1-Stearoyl-2-hydroxy-sn-glycero-3-phosphoethanolamine | Negative | Kidney | 480.309 | 8.31 | LysoPE(18:0) | [M-H]- | 2 | 480.3, 283.26, 196.03, 140.01, 78.95 | Phosphatidylethanolamine And Derivatives | |
| L-Glutamic acid | Negative | Kidney | 146.045 | 0.62 | Glu | [M-H]- | 1 | 146.04, 128.03, 102.05, 85.02 | Amino Acids And Derivatives | |
| 2-Hydroxypalmitic acid | Negative | Kidney | 271.2269 | 8.59 | 2-Hydroxypalmitic acid | [M-H]- | 1 | 271.2273, 225.2225 | Fatty Acids And Derivatives | |
| 1,2,3-Propanetricarboxylic acid, 2-hydroxy- | Negative | Kidney | 191.0187 | 0.7 | Citric Acid | [M-H]- | 1 | 191.01, 129.01, 111.09, 87.01, 85.02 | Miscellaneous | |
| 1-Palmitoyl-2-arachidonoyl-sn-glycero-3-phosphocholine | Negative | Kidney | 826.5608 | 10.2 | PC(16:0/20:4) | [M+HCO2]- | 1 | 826.5570, 766.5362, 480.3084, 303.2321, 255.2321 | Phosphatidylcholine And Derivatives | |
| 1-Palmitoyl-2-linoleoyl-sn-glycero-3-phosphocholine | Negative | Kidney | 802.5607 | 10.25 | PC(16:0/18:2) | [M-H]- | 2 | 802.56, 742.53, 480.3, 391.22, 279.23, 255.23 | Phosphatidylcholine And Derivatives | |
| Carbocyclic thromboxane A2 | Negative | Kidney | 329.2477 | 9.03 | Carbocyclic thromboxane A2 | [M-H-H2O]- | 1 | 329.2470, 285.2580, 231.2046 | Miscellaneous | |
| cis-4,7,10,13,16,19-Docosahexaenoic acid | Negative | Kidney | 327.232 | 8.65 | Cervonic acid | [M-H]- | 2 | 327.23, 309.2, 283.24, 249.18, 229.19, 191.18, 177.16 | Fatty Acids And Derivatives | |
| 1-Palmitoyl-2-docosahexaenoyl-sn-glycero-3-phosphocholine | Negative | Kidney | 850.5611 | 10.15 | PC(16:0/22:6) | [M-H]- | 2 | 850.56, 790.54, 480.3, 327.23, 283.24 | Phosphatidylcholine And Derivatives | |
| 1-Stearoyl-2-arachidonoyl-sn-glycero-3-phospho-(1'-myo-inositol) | Negative | Kidney | 886.6084 | 10.37 | PI(18:0/20:4) | [M-2H]- | 2 | 886.6, 419.25, 283.26 | Phosphatidylinositol And Derivatives | |
| 1-Oleoyl-2-palmitoyl-sn-glycero-3-phosphocholine | Negative | Kidney | 804.5756 | 10.35 | PC(18:1/16:0) | [M+H]+ | 2 | 804.57, 744.55, 281.24, 255.23 | Phosphatidylcholine And Derivatives | |
| 1-Palmitoyl-2-hydroxy-sn-glycero-3-phosphoethanolamine | Negative | Kidney | 452.2776 | 7.48 | LysoPE(16:0) | [M-H]- | 2 | 452.27, 255.23, 196.04 | Phosphatidylethanolamine And Derivatives | |
| 8,11-Tridecadienoic acid, 13-(3-pentyl-2-oxiranyl)-, (8Z,11Z)- | Negative | Kidney | 303.2321 | 8.79 | 14(15)-EpEDE | [M-H-H2O]- | 1 | 303.2325, 285.2220, 259.2429, 231.2116, 191.1077, 177.0919, 163.0764 | Fatty Acids And Derivatives | |
| 1-Stearoyl-2-docosahexaenoyl-sn-glycero-3-phosphocholine | Negative | Kidney | 878.5919 | 10.26 | PC(18:0/22:6) | [M+HCO2]- | 1 | 878.5917, 818.5676, 508.3403, 327.2319, 283.2632 | Phosphatidylcholine And Derivatives | |
| 1-Palmitoyl-2-arachidonoyl-sn-glycero-3-phosphoserine | Negative | Kidney | 781.4888 | 11.91 | PS(16:0/20:4) | [M-2H]- | 2 | 781.48, 605.48, 439.22, 391.22, 303.23, 255.23 | Phosphatidylserine And Derivatives | |
| 1-Stearoyl-2-hydroxy-sn-glycero-3-phosphocholine | Negative | Kidney | 568.2675 | 7.42 | LysoPC(18:0) | [M+Formic-H]- | 2 | 568.26, 508.33, 419.25, 283.26, 224.06, 152.99 | Phosphatidylcholine And Derivatives | |
| 2-Docosahexaenoyl-1-stearoyl-sn-glycero-3-phosphoserine | Negative | Kidney | 834.5288 | 11.91 | PS(18:0/22:6) | [M-H]- | 2 | 834.52, 747.49, 403.22, 419.25, 283.26, 152.99 | Phosphatidylserine And Derivatives | |
| Glycine, N-(2-phenylacetyl)- | Negative | Kidney | 192.0654 | 3.15 | Phenaceturic acid | [M-H]- | 1 | 192.06, 74.02 | Amino Acids And Derivatives | |
| 1-Palmitoyl-2-oleoyl-phosphatidylglycerol | Negative | Kidney | 747.5197 | 10.26 | PG(16:0/18:1) | [M-H]- | 2 | 747.51, 281.24, 152.99 | Phosphatidylglycerol And Derivatives | |
| 1-Palmitoyl-2-stearoyl-sn-glycero-3-phosphocholine | Negative | Kidney | 806.5851 | 10.39 | PC(16:0/18:0) | [M+HCO2]- | 1 | 806.5887, 746.5676, 480.3088, 283.2633, 255.2320 | Phosphatidylcholine And Derivatives | |
| L-Aspartic acid | Negative | Kidney | 132.0292 | 0.62 | Asp | [M-H]- | 2 | 132.02, 115, 88.04, 71.01 | Amino Acids And Derivatives | |
| 2-Docosahexaenoyl-1-stearoyl-sn-glycero-3-phosphoethanolamine | Negative | Kidney | 790.5385 | 10.3 | PE(18:0/22:6) | [M-H]- | 2 | 790.53, 480.3, 327.23, 283.26 | Phosphatidylethanolamine And Derivatives | |
| 1-Stearoyl-2-arachidonoyl-sn-glycero-3-phosphoserine | Negative | Kidney | 810.5296 | 11.92 | PS(18:0/20:4) | [M-H]- | 2 | 810.52, 723.49, 419.25, 283.26, 152.99 | Phosphatidylserine And Derivatives | |
| 1-Octadecanoyl-sn-glycero-3-phosphoserine | Negative | Kidney | 524.2986 | 12.01 | PS(18:0/0:0) | [M-H]- | 2 | 524.29, 437.26, 152.99 | Phosphatidylserine And Derivatives | |
| 1-(1Z-octadecenyl)-2-docosahexaenoyl-sn-glycero-3-phosphoethanolamine | Negative | Kidney | 464.3138 | 8.52 | PE(P-18:0/22:6) | [M-H]- | 2 | 464.3165, 267.2704, 196.0388, 140.0125, 78.9594 | Phosphoethanolamine And Derivatives | |
| cis-5,8,11,14,17-Eicosapentaenoic acid | Positive | Kidney | 303.2307 | 8.38 | Eicosapentaenoic acid | [M+H]+ | 2 | 303.23, 163.14, 147.17, 121.1, 96.07 | Fatty Acids And Derivatives | |
| 2-Oleoyl-1-palmitoyl-sn-glycero-3-phosphocholine | Positive | Kidney | 782.5704 | 10.25 | PC(18:0/18:1) | [M+H]+ | 2 | 782.57, 184.07, 86.09 | Phosphatidylcholine And Derivatives | |
| DL-Phenylalanine | Positive | Kidney | 166.0862 | 1.11 | Phe | [M+H]+ | 1 | 166.08, 120.08, 103.05 | Amino Acids And Derivatives | |
| 6H-Purin-6-one, 1,7-dihydro- | Positive | Kidney | 137.0462 | 0.72 | Hypoxanthine | [M+H]+ | 1 | 137.27, 137.04, 119.03, 110.03, 82.01 | Miscellaneous | |
| Niacinamide | Positive | Kidney | 123.0415 | 0.69 | Niacinamide | [M+H]+ | 1 | 123.04, 96.04, 80.04 | Miscellaneous | |
| 1-Palmitoyl-sn-glycero-3-phosphocholine | Positive | Kidney | 518.3202 | 7.49 | LysoPC(16:0) | [M+H]+ | 2 | 518.32, 459.24, 313.27, 146.98 | Phosphatidylcholine And Derivatives | |
| cis-5,8,11,14-Eicosatetraenoic acid | Positive | Kidney | 305.2473 | 8.78 | Arachidonic acid | [M+H]+ | 1 | 305.24, 287.23, 269.22, 245.22, 147.11, 121.1, 93.07 | Fatty Acids And Derivatives | |
| 1-O-Octadecyl-sn-glyceryl-3-phosphorylcholine | Positive | Kidney | 510.3542 | 7.88 | LysoPC(O-18:0) | [M+H]+ | 2 | 510.35, 184.07, 125, 104.1, 86.09 | Phosphatidylcholine And Derivatives | |
| Palmitoyleicosapentaenoyl phosphatidylcholine | Positive | Kidney | 780.5565 | 10.15 | PC(16:0/20:5) | [M+H]+ | 2 | 780.56, 184.07 | Phosphatidylcholine And Derivatives | |
| Arachidonoylthiophosphorylcholine | Positive | Kidney | 784.5822 | 10.28 | Arachidonoyl Thio-PC | [M+H]+ | 2 | 784.58, 184.07 | Phosphatidylcholine And Derivatives | |
| 1,2-Dipalmitoleoyl-sn-glycero-3-phosphocholine | Positive | Kidney | 730.545 | 10.19 | PC(16:1/16:1) | [M+H]+ | 2 | 730.54, 184.07 | Phosphatidylcholine And Derivatives | |
| 1-(1Z-Octadecenyl)-2-(5Z,8Z,11Z,14Z-eicosatetraenoyl)-sn-glycero-3-phosphocholine | Positive | Kidney | 794.5986 | 10.27 | PC(P-18:0/20:4) | [M+H]+ | 2 | 794.58, 184.07 | Phosphatidylcholine And Derivatives | |
| 1-O-Hexadecyl-2-O-(4Z,7Z,10Z,13Z,16Z,19Z-docosahexaenoyl)-sn-glyceryl-3-phosphorylcholine | Positive | Kidney | 792.59 | 10.26 | PC(O-16:0/22:6) | [M+H]+ | 2 | 792.59, 184.07 | Phosphatidylcholine And Derivatives | |
| 1-Oleoyl-2-myristoyl-sn-glycero-3-phosphocholine | Positive | Kidney | 732.5564 | 10.31 | PC(18:1/14:0) | [M+H]+ | 2 | 732.55, 184.07 | Phosphatidylcholine And Derivatives | |
| N-(Octadecanoyl)sphing-4-enine-1-phosphocholine | Positive | Kidney | 731.5898 | 10.4 | Sphingomyelin | [M+H]+ | 2 | 731.58, 184.07 | Sphingomyelin | |
| 12(13)-Epoxy-9Z-octadecenoic acid | Negative | Spleen | 295.227 | 7.22 | 12(13)-EpOME | [M-H]- | 2 | 195.22, 277.21, 195.13, 183.01, 171.1 | Fatty Acis And Derivative | |
| L-Aspartic acid | Negative | Spleen | 132.0293 | 0.62 | Asp | [M-H]- | 2 | 132.03, 115, 88.04, 71.01 | Amino Acid And Derivatives | |
| cis-4,7,10,13,16,19-Docosahexaenoic acid | Negative | Spleen | 327.2322 | 8.65 | Cervonic acid | [M-H]- | 2 | 327.23, 249.17, 229.19, 177.16 | Fatty Acis And Derivative | |
| L-Glutamic acid | Negative | Spleen | 146.0449 | 0.62 | Glu | [M-H]- | 2 | 146.04, 128.04, 102.06 | Amino Acid And Derivatives | |
| D-Mannose 6-phosphate | Negative | Spleen | 259.0216 | 0.66 | Mannose 6-Phosphate | [M-H]- | 2 | 259.02, 168.99, 96.96, 78.95 | Miscellaneous | |
| 1-Palmitoyl-2-linoleoyl-sn-glycero-3-phosphocholine | Negative | Spleen | 802.5608 | 10.24 | PC(16:0/18:2) | [M+Formic]- | 2 | 802.56, 742.53, 480.3, 391.22, 279.23, 255.23 | Phosphatidylcholine And Derivatives | |
| 1-Palmitoyl-2-arachidonoyl-sn-glycero-3-phosphocholine | Negative | Spleen | 826.5609 | 10.2 | PC(16:0/20:4) | [M-H]- | 3 | 826.56, 766.53 | Phosphatidylcholine And Derivative | |
| 2-Linoleoyl-1-palmitoyl-sn-glycero-3-phosphoethanolamine | Negative | Spleen | 714.5079 | 10.31 | PE(16:0/18:2) | [M-H]- | 2 | 714.5, 452.27, 279.23, 255.23 | Phosphatidylethanolamine And Derivatives | |
| 2-Arachidonoyl-1-palmitoyl-sn-glycero-3-phosphoethanolamine | Negative | Spleen | 738.5085 | 10.27 | PE(16:0/20:4) | [M-H]- | 2 | 738.5, 500.27, 452.27, 303.23, 255.23 | Phosphatidylethanolamine And Derivatives | |
| 2-Arachidonoyl-1-stearoyl-sn-glycero-3-phosphoethanolamine | Negative | Spleen | 520.2662 | 7.46 | PE(18:0/20:4) | [M-H]- | 2 | 766.53, 480.3, 303.23 | Phosphatidylethanolamine And Derivatives | |
| 1-(1Z-Octadecenyl)-2-(4Z,7Z,10Z,13Z,16Z,19Z-docosahexaenoyl)-sn-glycero-3-phosphoethanolamine | Negative | Spleen | 464.312 | 7.87 | PE(P-18:0/22:6) | [M-H]- | 2 | 464.31, 267.27, 196.03, 140.01, 78.95 | Phosphatidylethanolamine And Derivatives | |
| 2-Oleoyl-1-palmitoyl-sn-glycero-3-phosphoserine | Negative | Spleen | 760.5127 | 11.91 | PS(16:0/18:1) | [M-H]- | 2 | 760.5, 673.47, 391.22, 152.99 | Phosphatidylserine And Derivative | |
| 1-Palmitoyl-2-arachidonoyl-sn-glycero-3-phosphoserine | Negative | Spleen | 782.4981 | 11.91 | PS(16:0/20:4) | [M-H]- | 2 | 782.49, 695.46, 391.22, 255.23 | Phosphatidylserine And Derivative | |
| 2-Oleoyl-1-stearoyl-sn-glycero-3-phosphoserine | Negative | Spleen | 788.5428 | 11.9 | PS(18:0/18:1) | [M-H]- | 2 | 788.54, 701.51, 419.25, 283.26 | Phosphatidylserine And Derivative | |
| 1-Stearoyl-2-linoleoyl-sn-glycero-3-phospho-L-serine | Negative | Spleen | 786.5289 | 11.91 | PS(18:0/18:2) | [M-H]- | 2 | 786.52, 699.49, 419.25, 283.26, 152.99, 78.95 | Phosphatidylserine And Derivative | |
| 1-Stearoyl-2-arachidonoyl-sn-glycero-3-phosphoserine | Negative | Spleen | 810.5295 | 11.91 | PS(18:0/20:4) | [M-H]- | 2 | 810.52, 723.49, 419.25, 283.26, 152.99, 78.95 | Phosphatidylserine And Derivative | |
| 2-Docosahexaenoyl-1-stearoyl-sn-glycero-3-phosphoserine | Negative | Spleen | 834.6139 | 10.43 | PS(18:0/22:6) | [M-H]- | 2 | 834.61, 147.49, 419.25, 283.26, 152.99, 78.95 | Phosphatidylserine And Derivative | |
| 8Z,11Z,14Z,17Z-Eicosatetraenoic acid | Negative | Spleen | 303.2321 | 8.8 | ω-3 Arachidonic acid | [M-H]- | 2 | 303.23, 285.22, 259.24, 205.19 | Fatty Acis And Derivative | |
| 11-Hydroxy-12E,14Z-eicosadienoic acid | Positive | Spleen | 307.2567 | 9.03 | 11-HEDE | [M+H]+ | 1 | 307.26, 289.24, 209.17, 149.12, 137.13, 123.11, 109.1, 95.08, 81.07, 67.05 | Fatty Acis And Derivative | |
| DL-Indole-3-lactic acid | Positive | Spleen | 206.0677 | 0.64 | 3-Indolyllactic acid | [M+H]+ | 2 | 206.06, 188.07, 146.06, 118.06 | Miscellaneous | |
| cis-5,8,11,14-Eicosatetraenoic acid | Positive | Spleen | 305.2477 | 8.8 | Arachidonic acid | [M+H]+ | 1 | 305.24, 287.23, 269.22, 259.2, 245.22, 221.15, 147.11, 121.1, | Fatty Acis And Derivative | |
| Arginine | Positive | Spleen | 175.0747 | 0.63 | Arg | [M+H]+ | 3 | 175.11, 116.07, 70.06 | Amino Acids And Derivatives | |
| Docosahexaenoyl PAF C-16 | Positive | Spleen | 792.5738 | 10.28 | DCHA-PAF | [M+H]+ | 2 | 792.57, 184.07 | Phosphatidylcholine And Derivative | |
| Gly-Leu-Arg | Positive | Spleen | 173.0806 | 0.85 | GLR | [M+2H]2+ | 2 | 173.08, 158.08, 116.06, 112.04, 86.1, 70.06, 60.05 | Short Peptides | |
| Glutathione, oxidized | Positive | Spleen | 613.1586 | 0.69 | GSSG | [M+H]+ | 1 | 613.15, 638.13, 484.11, 409.08, 355.07, 288.06, 231.04, 177.03, 130.04 | Miscellaneous | |
| 6H-Purin-6-one, 1,7-dihydro- | Positive | Spleen | 137.0466 | 0.7 | Hypoxanthine | [M+H]+ | 2 | 137.04, 119.03, 110.03, 82.04 | Miscellaneous | |
| (S)-2-Amino-3-methylbutanoic acid | Positive | Spleen | 118.0867 | 0.67 | L-Valine | [M+H]+ | 2 | 118.08, 72.08 | Amino Acids And Derivatives | |

| | | | | | | | | | |
|---|---|---|---|---|---|---|---|---|---|
| 1-Heptadecanoyl-sn-glycero-3-phosphocholine | Positive | Spleen | 510.355 | 7.9 | LysoPC(17:0) | [M+H]+ | 2 | 510.35, 184.07, 124.99, 104.1, 88.09 | Phosphatidylcholine And Derivative |
| 1-Behenoyl-2-hydroxy-sn-glycero-3-phosphocholine | Positive | Spleen | 580.3577 | 9.64 | LysoPC(22:0) | [M+H]+ | 2 | 580.35, 184.07, 104.07 | Phosphatidylcholine And Derivative |
| 1-Palmitoyl-2-hydroxy-sn-glycero-3-phosphoethanolamine | Positive | Spleen | 476.2791 | 7.46 | LysoPE(16:0) | [M+H]+ | 2 | 476.27, 433.23 | Phosphatidylethanolamine And Derivatives |
| N-Acetyl-L-phenylalanine | Positive | Spleen | 165.0544 | 0.71 | N-Ac-Phe | [M+-Ac]+ | 2 | 165.08, 149.05, 120.08, 103.05, 93.06, 77.03 | Amino Acids And Derivatives |
| 1-Myristoyl-2-palmitoyl-sn-glycero-3-phosphocholine | Positive | Spleen | 706.5434 | 10.3 | PC(14:0/16:0) | [M+H]+ | 2 | 706.54, 184.07, 86.09 | Phosphatidylcholine And Derivative |
| 2-Oleoyl-1-palmitoyl-sn-glycero-3-phosphocholine | Positive | Spleen | 760.5845 | 10.36 | PC(16:0/18:1) | [M+H]+ | 2 | 760.58, 184.07 | Phosphatidylcholine And Derivative |
| 1,2-Dipalmitoleoyl-sn-glycero-3-phosphocholine | Positive | Spleen | 730.544 | 10.2 | PC(16:1/16:1) | [M+H]+ | 2 | 730.54, 184.07 | Phosphatidylcholine |
| 1-Octadecanoyl-2-octadecenoyl-sn-glycero-3-phosphocholine | Positive | Spleen | 810.6004 | 10.33 | PC(18:0/18:1) | [M+H]+ | 2 | 810.58, 751.52, 184.07, 146.98 | Phosphatidylcholine And Derivative |
| 1-Docosahexaenoyl-2-stearoyl-sn-glycero-3-phosphocholine | Positive | Spleen | 834.5996 | 10.25 | PC(18:0/22:6) | [M+H]+ | 2 | 834.599, 184.07 | Phosphatidylcholine And Derivative |
| 1-Oleoyl-2-myristoyl-sn-glycero-3-phosphocholine | Positive | Spleen | 732.5571 | 10.3 | PC(18:1/14:0) | [M+H]+ | 2 | 732.55, 184.07 | Phosphatidylcholine And Derivative |
| 1-(1Z-Octadecenyl)-sn-glycero-3-phosphocholine | Positive | Spleen | 508.372 | 7.89 | PC(P-18:0/0:0) | [M+H]+ | 2 | 508.37, 184.07, 125, 104.1, 86.09 | Phosphatidylcholine And Derivative |
| N-Oleoyl-D-erythro-sphingosylphosphorylcholine | Positive | Spleen | 729.5727 | 10.38 | SM(d18:1/18:1) | [M+H]+ | 2 | 729.59, 184.07 | Sphingomyelin |
| N-(Octadecanoyl)sphing-4-enine-1-phosphocholine | Positive | Spleen | 731.5945 | 10.43 | SM(d18:1/18:0) | [M+H]+ | 2 | 731.6, 184.07 | Sphingomyelins |
| Tauroursodeoxycholic acid | Positive | Spleen | 482.324 | 8.33 | Taurolite | [M+H-H2O]+ | 2 | 482.32, 464.31 | Bile Acid Conjugates |

**Supplementary Table 2. Radiation effects Metabolites**

**Radiation effect at SD 4 and SD 9 for validated metabolites across all tissues**

| | | | SD 4 | | | | | SD 9 | | | | |
|---|---|---|---|---|---|---|---|---|---|---|---|---|
| | | | Rad/Vehicle | | | | | Rad/Vehicle | | | | |
| **Name** | **Matrix** | **Ionization Mode** | ***p-value*** | ***FDR*** [a] | ***Fold Change*** | | ***log2(FC)*** | ***p-value*** | ***FDR*** | ***Fold Change*** | | ***log2(FC)*** |
| PC(16:0/18:2) | Heart | Positive | 2.97E-04 | 5.20E-03 | 0.78077 | ↓ | -0.35704 | NS | NS | 1.0147 | ↑ | 0.020996 |
| PC(16:0/20:5) | Heart | Positive | 1.17E-02 | NS | 0.86089 | ↓ | -0.2161 | NS | NS | 0.84106 | ↓ | -0.24972 |
| Arachidonoyl Thio-PC | Heart | Positive | NS | NS | 0.92349 | ↓ | -0.11484 | 4.12E-03 | NS | 1.2167 | ↑ | 0.28302 |
| PC(16:1/16:1) | Heart | Positive | 1.34E-03 | 1.46E-02 | 0.75197 | ↓ | -0.41125 | NS | NS | 0.89575 | ↓ | -0.15884 |
| PE(18:0/22:6) | Heart | Positive | 5.45E-04 | 7.79E-03 | 1.4382 | ↑ | 0.52426 | NS | NS | 1.0307 | ↑ | 0.043632 |
| PC(16:0/18:0) | Heart | Positive | 4.71E-03 | 4.02E-02 | 1.4114 | ↑ | 0.4971 | NS | NS | 1.0014 | ↑ | 0.0019827 |
| Cervonic acid | Heart | Positive | 7.94E-05 | 2.58E-03 | 0.32755 | ↓ | -1.6102 | NS | NS | 0.93464 | ↓ | -0.097513 |
| Methyl oleoylethanolamide | Heart | Positive | 3.89E-07 | 5.44E-04 | 0.30528 | ↓ | -1.7118 | 2.15E-04 | NS | 0.59437 | ↓ | -0.75057 |
| DELTA.17-U-46619 | Heart | Positive | 3.67E-02 | NS | 0.71799 | ↓ | -0.47797 | NS | NS | 0.93877 | ↓ | -0.091156 |
| PC(22:6/18:0) | Heart | Positive | 2.57E-03 | 2.45E-02 | 1.3541 | ↑ | 0.43735 | NS | NS | 0.93759 | ↓ | -0.092976 |
| Acetylcarnitine | Heart | Positive | NS | NS | 1.3238 | ↑ | 0.40464 | 3.51E-02 | NS | 0.7238 | ↓ | -0.46634 |
| PC(16:0/22:6) | Heart | Positive | 5.41E-03 | 4.51E-02 | 1.1827 | ↑ | 0.2421 | NS | NS | 0.93659 | ↓ | -0.094508 |
| Adrenic Acid (22:4, n-6) | Heart | Positive | 2.32E-02 | NS | 0.56321 | ↓ | -0.82825 | NS | NS | 0.92045 | ↓ | -0.11959 |
| γ-Homolinolenic acid | Heart | Positive | 3.87E-04 | 6.27E-03 | 0.52484 | ↓ | -0.93004 | NS | NS | 0.86201 | ↓ | -0.21423 |
| DL-Norvaline | Heart | Positive | 2.02E-02 | NS | 0.68375 | ↓ | -0.54846 | 5.63E-03 | NS | 0.71433 | ↓ | -0.48533 |
| 2-AG | Heart | Positive | 1.85E-03 | 1.89E-02 | 0.34981 | ↓ | -1.5154 | NS | NS | 0.87715 | ↓ | -0.18911 |
| Eicosadienoic acid | Heart | Positive | 2.47E-04 | 4.61E-03 | 0.36406 | ↓ | -1.4577 | NS | NS | 0.81349 | ↓ | -0.2978 |
| LysoPE(16:0) | Heart | Positive | 5.53E-03 | 4.53E-02 | 0.432 | ↓ | -1.2109 | NS | NS | 0.68951 | ↓ | -0.53636 |
| Gln | Heart | Positive | 2.52E-02 | NS | 1.5181 | ↑ | 0.60222 | NS | NS | 0.88803 | ↓ | -0.17132 |
| 7α,12α-Dihydroxycholest-4-en-3-one | Heart | Positive | 8.37E-05 | 2.60E-03 | 0.23422 | ↓ | -2.0941 | NS | NS | 0.86757 | ↓ | -0.20495 |
| Methyl ricinoleate | Heart | Positive | 5.91E-04 | 8.20E-03 | 0.39619 | ↓ | -1.3357 | NS | NS | 1.1194 | ↑ | 0.16277 |
| Methyl DPA | Heart | Positive | 2.11E-03 | 2.09E-02 | 0.37005 | ↓ | -1.4342 | NS | NS | 0.9106 | ↓ | -0.13512 |
| Erucamide | Heart | Positive | 1.56E-02 | NS | 0.65836 | ↓ | -0.60306 | NS | NS | 0.98585 | ↓ | -0.020563 |
| 9(10)-EpOME | Heart | Positive | 1.70E-03 | 1.76E-02 | 0.52731 | ↓ | -0.92328 | 4.89E-04 | NS | 0.61706 | ↓ | -0.69653 |
| N-Arachidonoyl-L-alanine | Heart | Positive | 1.07E-04 | 3.02E-03 | 0.24252 | ↓ | -2.0438 | 3.18E-02 | NS | 0.632 | ↓ | -0.66201 |
| 2-Linoleoylglycerol | Heart | Positive | 5.98E-05 | 2.28E-03 | 0.44597 | ↓ | -1.165 | NS | NS | 0.79351 | ↓ | -0.33369 |
| PC(16:0/20:4) | Spleen | Negative | 1.68E-04 | 1.74E-03 | 1.2985 | ↑ | 0.3769 | 1.70E-03 | 1.08E-02 | 1.2121 | ↑ | 0.27752 |
| PS(16:0/18:1) | Spleen | Negative | 2.01E-06 | 4.94E-05 | 0.67875 | ↓ | -0.55905 | 1.86E-04 | 1.83E-03 | 0.74331 | ↓ | -0.42797 |
| PC(16:0/18:2) | Spleen | Negative | 3.29E-03 | 1.85E-02 | 1.2725 | ↑ | 0.34772 | 2.52E-03 | 1.47E-02 | 1.2789 | ↑ | 0.35487 |
| PS(18:0/20:4) | Spleen | Negative | 5.27E-03 | 2.63E-02 | 1.1937 | ↑ | 0.25539 | NS | NS | 1.1608 | ↑ | 0.21508 |
| PE(16:0/18:2) | Spleen | Negative | 2.01E-05 | 3.18E-04 | 0.61356 | ↓ | -0.70473 | 4.74E-02 | NS | 0.82721 | ↓ | -0.27368 |
| Cervonic acid | Spleen | Negative | NS | NS | 0.94012 | ↓ | -0.08908 | 1.41E-02 | NS | 1.7704 | ↑ | 0.82409 |
| PE(18:0/20:4) | Spleen | Negative | 3.71E-03 | 2.00E-02 | 0.64323 | ↓ | -0.6366 | NS | NS | 0.99501 | ↓ | -0.0072137 |
| ω-3 Arachidonic acid | Spleen | Negative | NS | NS | 1.3311 | ↑ | 0.41259 | 1.18E-02 | 4.93E-02 | 1.8112 | ↑ | 0.85694 |
| PS(18:0/18:2) | Spleen | Negative | 2.07E-05 | 3.26E-04 | 0.74614 | ↓ | -0.42248 | NS | NS | 0.87768 | ↓ | -0.18823 |
| PE(P-18:0/22:6) | Spleen | Negative | NS | NS | 0.83069 | ↓ | -0.26762 | 5.76E-03 | 2.78E-02 | 1.6116 | ↑ | 0.6885 |
| PS(16:0/20:4) | Spleen | Negative | 8.89E-05 | 1.06E-03 | 0.75224 | ↓ | -0.41073 | 3.92E-05 | 5.03E-04 | 0.67253 | ↓ | -0.57232 |
| Glu | Spleen | Negative | 8.42E-05 | 1.02E-03 | 0.74132 | ↓ | -0.43183 | 1.27E-05 | 2.15E-04 | 0.63469 | ↓ | -0.65587 |
| PS(18:0/22:6) | Spleen | Negative | NS | NS | 1.081 | ↑ | 0.11232 | 4.67E-02 | NS | 1.1478 | ↑ | 0.19883 |

| | | | | | | | | | | | | |
|---|---|---|---|---|---|---|---|---|---|---|---|---|
| PE(16:0/20:4) | Spleen | Negative | 2.70E-05 | 4.01E-04 | 0.72991 | ↓ | -0.4542 | 2.22E-04 | 2.12E-03 | 0.79107 | ↓ | -0.33812 |
| Asp | Spleen | Negative | 2.72E-02 | NS | 0.83364 | ↓ | -0.26251 | 1.38E-05 | 2.25E-04 | 0.60256 | ↓ | -0.73083 |
| PE(P-18:0/22:6) | Spleen | Negative | 7.08E-05 | 9.06E-04 | 0.4727 | ↓ | -1.081 | NS | NS | 0.72367 | ↓ | -0.4666 |
| Mannose 6-Phosphate | Spleen | Negative | 1.26E-02 | NS | 0.77305 | ↓ | -0.37137 | 1.67E-02 | NS | 0.78248 | ↓ | -0.35387 |
| GLR | Spleen | Positive | 2.70E-03 | 1.90E-02 | 1.3355 | ↑ | 0.41738 | 1.68E-03 | 1.31E-02 | 1.4569 | ↑ | 0.54293 |
| PC(18:0/22:6) | Spleen | Positive | NS | NS | 1.0089 | ↑ | 0.012813 | 2.04E-02 | NS | 0.88795 | ↓ | -0.17145 |
| Hypoxanthine | Spleen | Positive | 1.15E-04 | 1.88E-03 | 0.58217 | ↓ | -0.7805 | 6.34E-07 | 1.62E-05 | 0.50178 | ↓ | -0.99489 |
| PC(16:1/16:1) | Spleen | Positive | 1.75E-09 | 6.75E-07 | 0.63929 | ↓ | -0.64545 | 1.12E-08 | 6.46E-07 | 0.62086 | ↓ | -0.68767 |
| LysoPC(17:0) | Spleen | Positive | 4.28E-02 | NS | 0.75044 | ↓ | -0.4142 | NS | NS | 0.99466 | ↓ | -0.0077273 |
| N-Ac-Phe | Spleen | Positive | 5.69E-03 | 3.18E-02 | 1.3291 | ↑ | 0.41041 | NS | NS | 1.1312 | ↑ | 0.17791 |
| LysoPE(16:0) | Spleen | Positive | 6.75E-04 | 7.46E-03 | 0.68664 | ↓ | -0.54237 | NS | NS | 1.1791 | ↑ | 0.23769 |
| PC(14:0/16:0) | Spleen | Positive | 7.57E-05 | 1.34E-03 | 0.62695 | ↓ | -0.67359 | 1.06E-04 | 1.23E-03 | 0.7469 | ↓ | -0.42102 |
| 11-HEDE | Spleen | Positive | NS | NS | 1.3256 | ↑ | 0.40661 | 1.01E-02 | NS | 1.8189 | ↑ | 0.86304 |
| PC(18:0/18:1)□ | Spleen | Positive | 1.57E-02 | NS | 1.152 | ↑ | 0.20418 | NS | NS | 0.91465 | ↓ | -0.12871 |
| L-Valine | Spleen | Positive | 9.29E-07 | 4.93E-05 | 0.58216 | ↓ | -0.7805 | 1.64E-09 | 1.21E-07 | 0.47058 | ↓ | -1.0875 |
| Arachidonic acid | Spleen | Positive | NS | NS | 1.4092 | ↑ | 0.49487 | 3.81E-03 | 2.59E-02 | 1.7899 | ↑ | 0.83984 |
| Taurolite | Spleen | Positive | 3.84E-03 | 2.42E-02 | 0.63292 | ↓ | -0.6599 | NS | NS | 1.3122 | ↑ | 0.39194 |
| DCHA-PAF | Spleen | Positive | 1.48E-05 | 3.95E-04 | 0.79423 | ↓ | -0.33237 | 7.03E-09 | 4.51E-07 | 0.71489 | ↓ | -0.48421 |
| LysoPC(22:0) | Spleen | Positive | NS | NS | 0.92673 | ↓ | -0.10978 | NS | NS | 1.0466 | ↑ | 0.065654 |
| PC(18:1/14:0) | Spleen | Positive | 2.46E-03 | 1.81E-02 | 0.85126 | ↓ | -0.23232 | 5.38E-06 | 9.40E-05 | 0.77067 | ↓ | -0.37581 |
| SM(d18:1/18:1) | Spleen | Positive | 4.78E-05 | 9.38E-04 | 1.3619 | ↑ | 0.44565 | 2.18E-05 | 3.18E-04 | 1.32 | ↑ | 0.40051 |
| GSSG | Spleen | Positive | 2.38E-02 | NS | 0.60964 | ↓ | -0.71397 | 1.59E-03 | 1.26E-02 | 0.47616 | ↓ | -1.0705 |
| SM(d18:1/18:0) | Spleen | Positive | NS | NS | 1.154 | ↑ | 0.20658 | 3.72E-02 | NS | 1.1948 | ↑ | 0.25678 |
| PC(P-18:0/0:0) | Spleen | Positive | 2.54E-06 | 1.04E-04 | 0.37062 | ↓ | -1.432 | 1.33E-04 | 1.46E-03 | 0.34645 | ↓ | -1.5293 |
| Arg | Spleen | Positive | 1.01E-06 | 5.10E-05 | 1.8313 | ↑ | 0.87288 | 2.29E-05 | 3.26E-04 | 1.7485 | ↑ | 0.80608 |
| 3-Indolyllactic acid | Spleen | Positive | 1.44E-05 | 3.89E-04 | 0.61113 | ↓ | -0.71044 | 1.90E-06 | 3.88E-05 | 0.63671 | ↓ | -0.6513 |
| Glu | Kidney | Negative | NS | NS | 0.97058 | ↓ | -0.043084 | 1.03E-06 | 1.65E-04 | 0.80399 | ↓ | -0.31476 |
| 2-Hydroxypalmitic acid | Kidney | Negative | 2.75E-02 | NS | 0.78839 | ↓ | -0.34301 | NS | NS | 1.0588 | ↑ | 0.082458 |
| Citric Acid | Kidney | Negative | NS | NS | 0.57417 | ↓ | -0.80044 | 1.78E-02 | NS | 2.6207 | ↑ | 1.3899 |
| Cervonic acid | Kidney | Negative | NS | NS | 0.93407 | ↓ | -0.098398 | 1.32E-02 | NS | 1.354 | ↑ | 0.43726 |
| LysoPE(16:0) | Kidney | Negative | NS | NS | 0.90602 | ↓ | -0.14239 | 3.08E-04 | 6.50E-03 | 1.4798 | ↑ | 0.56538 |
| PE(P-18:0/22:6) | Kidney | Negative | NS | NS | 1.0761 | ↑ | 0.10579 | 3.66E-03 | 3.45E-02 | 1.6061 | ↑ | 0.68357 |
| PS(18:0/22:6) | Kidney | Negative | 3.42E-02 | NS | 0.87227 | ↓ | -0.19716 | NS | NS | 0.91973 | ↓ | -0.12071 |
| Phenaceturic acid | Kidney | Negative | 3.56E-02 | NS | 1.6147 | ↑ | 0.69123 | 9.40E-03 | NS | 4.0261 | ↑ | 2.0094 |
| Asp | Kidney | Negative | NS | NS | 0.92302 | ↓ | -0.11557 | 2.19E-02 | NS | 0.8859 | ↓ | -0.17478 |
| Carbocyclic thromboxane A2 | Kidney | Negative | 1.99E-03 | NS | 0.55164 | ↓ | -0.8582 | 1.86E-03 | 2.16E-02 | 1.6133 | ↑ | 0.69002 |
| PS(18:0/0:0) | Kidney | Negative | NS | NS | 1.1147 | ↑ | 0.15663 | 6.99E-07 | 1.49E-04 | 3.1595 | ↑ | 1.6597 |
| LysoPC(18:0) | Kidney | Negative | 2.04E-02 | NS | 0.74492 | ↓ | -0.42485 | NS | NS | 0.95544 | ↓ | -0.065766 |
| PC(18:0/18:1) | Kidney | Positive | 1.83E-02 | NS | 1.1309 | ↑ | 0.17746 | 1.09E-03 | 1.38E-02 | 1.2546 | ↑ | 0.32719 |
| Eicosapentaenoic acid | Kidney | Positive | 1.31E-02 | NS | 0.69474 | ↓ | -0.52546 | NS | NS | 0.9571 | ↓ | -0.063263 |
| PC(16:1/16:1) | Kidney | Positive | NS | NS | 1.0702 | ↑ | 0.097882 | 1.84E-02 | NS | 0.86814 | ↓ | -0.204 |
| PC(P-18:0/20:4) | Kidney | Positive | NS | NS | 0.96158 | ↓ | -0.056528 | 2.01E-06 | 1.86E-04 | 0.62983 | ↓ | -0.66697 |
| Arachidonoyl Thio-PC | Kidney | Positive | NS | NS | 1.0747 | ↑ | 0.10387 | 1.14E-02 | NS | 1.1696 | ↑ | 0.22597 |
| PC(O-16:0/22:6) | Kidney | Positive | NS | NS | 0.98209 | ↓ | -0.026068 | 2.24E-06 | 1.86E-04 | 0.64166 | ↓ | -0.64012 |
| LysoPC(O-18:0) | Kidney | Positive | NS | NS | 0.68534 | ↓ | -0.54512 | 1.81E-02 | NS | 1.4744 | ↑ | 0.56009 |

| | | | | | | | | | | | | |
|---|---|---|---|---|---|---|---|---|---|---|---|---|
| LysoPC(16:0) | Kidney | Positive | NS | NS | 0.94716 | ↓ | -0.078316 | 1.96E-03 | 1.99E-02 | 1.2897 | ↑ | 0.36706 |

*Note*. All metabolite names are validated through tandem MS.

[a] Numbers are FDR adjusted *P* values. NS = not significant (FDR, $P > 0.05$).

Supplementary Table 3. Radiation effects Pathways

**Radiation dysregulation pathways**

**Table 3A**

| **Heart** | **SD 4** | | **SD 9** | |
|---|---|---|---|---|
| **Pathway** | ***overlap size*** | ***p-value*** | ***overlap size*** | ***p-value*** |
| De novo fatty acid biosynthesis | 17(23) | 8.40E-05 | - | - |
| Fatty acid activation | 11(15) | 6.72E-04 | 3(15) | 3.45E-02 |
| Arachidonic acid metabolism | 7(9) | 3.61E-03 | - | - |
| Leukotriene metabolism | 7(9) | 3.61E-03 | 3(9) | 6.89E-03 |
| Omega-3 fatty acid metabolism | - | - | 2(4) | 8.65E-03 |
| Fatty Acid Metabolism | 6(8) | 8.82E-03 | - | - |
| Limonene and pinene degradation | 7(10) | 8.91E-03 | - | - |
| Bile acid biosynthesis | - | - | 4(17) | 9.41E-03 |
| Linoleate metabolism | - | - | 3(12) | 1.66E-02 |
| Saturated fatty acids beta-oxidation | 4(5) | 1.75E-02 | - | - |
| Xenobiotics metabolism | 4(5) | 1.75E-02 | - | - |

*Note*. Pathway analysis result with positive mode and negative mode combined.

[a] Numbers in parenthesis indicates the pathway size.

**Table 3B**

| **Spleen** | **SD 4** | | **SD 9** | |
|---|---|---|---|---|
| **Pathway** | ***overlap size*** | ***p-value*** | ***overlap size*** | ***p-value*** |
| Tyrosine metabolism | 12(16) | 8.40E-05 | - | - |
| Pyrimidine metabolism | 3(3) | 9.24E-03 | 14(16) | 5.04E-04 |
| Glycine, serine, alanine and threonine metabolism | 9(14) | 3.32E-02 | 12(14) | 1.51E-03 |
| Glycerophospholipid metabolism | - | - | 15(19) | 2.02E-03 |
| Fructose and mannose metabolism | 6(6) | 2.27E-03 | 5(6) | 4.92E-02 |
| Beta-Alanine metabolism | 6(6) | 2.27E-03 | 6(6) | 7.06E-03 |
| Glycolysis and Gluconeogenesis | 4(4) | 2.35E-03 | 7(7) | 3.36E-03 |
| Alanine and Aspartate Metabolism | 6(7) | 8.91E-03 | 7(7) | 3.36E-03 |
| Vitamin B9 (folate) metabolism | - | - | 7(7) | 3.36E-03 |
| Aspartate and asparagine metabolism | 5(7) | 1.36E-02 | 11(13) | 3.70E-03 |
| Urea cycle/amino group metabolism | 5(6) | 4.20E-03 | 8(10) | 2.12E-02 |
| Vitamin B3 (nicotinate and nicotinamide) metabolism | - | - | 6(6) | 7.06E-03 |
| Pentose phosphate pathway | - | - | 8(9) | 7.48E-03 |
| Valine, leucine and isoleucine degradation | 3(3) | 9.24E-03 | 3(3) | 1.62E-02 |
| Aminosugars metabolism | 3(3) | 9.24E-03 | 3(3) | 1.62E-02 |
| Lysine metabolism | 3(3) | 9.24E-03 | 3(3) | 1.62E-02 |
| Tryptophan metabolism | 7(9) | 1.18E-02 | 7(9) | 3.66E-02 |
| TCA cycle | 4(4) | 1.31E-02 | 4(4) | 3.00E-02 |
| Carbon fixation | - | - | 5(5) | 1.43E-02 |
| Glycosphingolipid biosynthesis - globoseries | - | - | 5(5) | 1.43E-02 |
| Fatty acid activation | - | - | 5(7) | 2.21E-02 |
| Methionine and cysteine metabolism | 4(6) | 3.71E-02 | 9(12) | 2.69E-02 |
| Butanoate metabolism | 2(2) | 3.68E-02 | 6(7) | 2.71E-02 |

| | | | | |
|---|---|---|---|---|
| Ascorbate (Vitamin C) and Aldarate Metabolism | - | - | 6(7) | 2.71E-02 |
| Glutamate metabolism | 3(4) | 3.94E-02 | 6(7) | 2.71E-02 |
| Galactose metabolism | - | - | 6(7) | 2.71E-02 |
| Glycosphingolipid biosynthesis - neolactoseries | - | - | 4(4) | 3.00E-02 |
| Keratan sulfate biosynthesis | - | - | 4(4) | 3.00E-02 |
| O-Glycan biosynthesis | - | - | 4(4) | 3.00E-02 |
| Propanoate metabolism | - | - | 4(4) | 3.00E-02 |
| Glycosphingolipid biosynthesis - lactoseries | - | - | 4(4) | 3.00E-02 |
| Blood Group Biosynthesis | - | - | 4(4) | 3.00E-02 |
| Sialic acid metabolism | - | - | 7(9) | 3.66E-02 |
| N-Glycan biosynthesis | 2(2) | 3.68E-02 | 7(9) | 3.66E-02 |
| Arginine and Proline Metabolism | 2(2) | 3.68E-02 | - | - |
| Glutathione Metabolism | - | - | 5(6) | 4.92E-02 |

*Note*. Pathway analysis result with positive mode and negative mode combined.

[a] Numbers in parenthesis indicates the pathway size.

**Table 3C**

| **Kidney** | **SD 4** | | **SD 9** | |
|---|---|---|---|---|
| **Pathway** | ***overlap size*** | ***p-value*** | ***overlap size*** | ***p-value*** |
| Alanine and Aspartate Metabolism | 6(7) | 1.68E-04 | 5(7) | 3.42E-02 |
| Ascorbate (Vitamin C) and Aldarate Metabolism | - | - | 8(8) | 4.20E-04 |
| Aspartate and asparagine metabolism | 8(15) | 1.34E-03 | - | - |
| Glycolysis and Gluconeogenesis | 5(7) | 1.68E-03 | 6(7) | 6.05E-03 |
| De novo fatty acid biosynthesis | 4(11) | 2.52E-03 | - | - |
| Vitamin A (retinol) metabolism | 3(9) | 1.09E-02 | 5(9) | 2.77E-03 |
| Purine metabolism | - | - | 10(14) | 3.53E-03 |
| Vitamin B1 (thiamin) metabolism | 3(3) | 3.53E-03 | - | - |
| Omega-3 fatty acid metabolism | 3(3) | 3.53E-03 | - | - |
| Pyruvate Metabolism | - | - | 5(5) | 3.70E-03 |
| Methionine and cysteine metabolism | - | - | 8(11) | 7.73E-03 |
| Aminosugars metabolism | 5(9) | 8.23E-03 | - | - |
| Arginine and Proline Metabolism | 5(9) | 8.23E-03 | 6(9) | 3.33E-02 |
| Heparan sulfate degradation | - | - | 4(4) | 9.83E-03 |
| Histidine metabolism | 3(4) | 1.34E-02 | - | - |
| Butanoate metabolism | 5(10) | 1.34E-02 | - | - |
| Beta-Alanine metabolism | - | - | 5(6) | 1.40E-02 |
| Hexose phosphorylation | 2(2) | 2.13E-02 | - | - |
| Glycerophospholipid metabolism | 7(19) | 2.57E-02 | - | - |
| Sialic acid metabolism | 4(8) | 2.59E-02 | - | - |
| Glycine, serine, alanine and threonine metabolism | - | - | 8(13) | 2.66E-02 |
| Chondroitin sulfate degradation | - | - | 3(3) | 2.67E-02 |
| Fructose and mannose metabolism | 2(3) | 4.68E-02 | 3(3) | 2.67E-02 |
| Glyoxylate and Dicarboxylate Metabolism | - | - | 3(3) | 2.67E-02 |
| Glutathione Metabolism | 2(3) | 4.68E-02 | 3(3) | 2.67E-02 |
| Vitamin B9 (folate) metabolism | 3(5) | 2.72E-02 | 1(1) | 4.00E-02 |

| | | | | |
|---|---|---|---|---|
| Glutamate metabolism | - | - | 4(5) | 3.34E-02 |
| Leukotriene metabolism | - | - | 2(4) | 3.88E-02 |
| Tyrosine metabolism | - | - | 12(23) | 3.91E-02 |
| Vitamin K metabolism | - | - | 1(1) | 4.00E-02 |
| Lysine metabolism | 3(6) | 4.34E-02 | - | - |
| N-Glycan biosynthesis | 3(6) | 4.34E-02 | - | - |
| Propanoate metabolism | 3(6) | 4.34E-02 | - | - |
| Alkaloid biosynthesis II | 2(3) | 4.68E-02 | - | - |
| Tryptophan metabolism | 4(10) | 4.89E-02 | - | - |

*Note.* Pathway analysis result with positive mode and negative mode combined.

[a] Numbers in parenthesis indicates the pathway size.

**SD 4**

Heart

| pathway | overlap size | pathway size | p-value | overlap Empirical Compounds | overlap features | overlap features name |
|---|---|---|---|---|---|---|
| De novo fatty acid biosynthesis | 17 | 23 | 8.40E-05 | E613,E933,E898,E82,E227,E687,E926,E826,E119,E379,E810,E261,E455,E490,E373,E203,E438 | C06427/C06426,C00249,C01530,C00219,C01595,C06429,C06427/C06426,C00712,C00249,C06429,C03242,C00712,C00219,C01530,C03242,C00249,C00219 | acid$(5Z,8Z,11Z,14Z)-Icosatetraenoic acid; Arachidonate; Arachidonic acid; cis-5,8,11,14-Eicosatetraenoic acid$Linoleate; Linoleic acid; (9Z,12Z)-Octadecadienoic acid; 9-cis,12-cis-Octadecadienoate; 9-cis,12-cis-Octadecadienoic acid$(4Z,7Z,10Z,13Z,16Z,19Z)-Docosahexaenoic acid; 4,7,10,13,16,19-Docosahexaenoic acid; Docosahexaen |
| Fatty acid activation | 11 | 15 | 6.72E-04 | E613,E933,E898,E227,E926,E119,E810,E490,E848,E373,E203 | C06427/C06426,C00249,C01530,C01595,C06427/C06426,C00249,C03242,C01530,C06423,C03242,C00249 | tearic acid$Linoleate; Linoleic acid; (9Z,12Z)-Octadecadienoic acid; 9-cis,12-cis-Octadecadienoate; 9-cis,12-cis-Octadecadienoic acid$(9Z,12Z,15Z)-Octadecatrienoic acid; alpha-Linolenic acid; 9,12,15-Octadecatrienoic acid; Linolenate; alpha-Linolenate/(6Z,9Z,12Z)-Octadecatrienoic acid; 6,9,12-Octadecatrienoic acid; gamma-Linolenic |
| Arachidonic acid metabolism | 7 | 9 | 0.003613142 | E916,E613,E293,E82,E926,E455,E438 | leuktrE4,C06427/C06426,C00127,C00219,C06427/C06426,C00219,C00219 | ulfide; GSSG; Oxiglutatione; Oxidized glutathione$(5Z,8Z,11Z,14Z)-Icosatetraenoic acid; Arachidonate; Arachidonic acid; cis-5,8,11,14-Eicosatetraenoic acid$(9Z,12Z,15Z)-Octadecatrienoic acid; alpha-Linolenic acid; 9,12,15-Octadecatrienoic acid; Linolenate; alpha-Linolenate/(6Z,9Z,12Z)-Octadecatrienoic acid; 6,9,12-Octadecatrienoic |
| Leukotriene metabolism | 7 | 9 | 0.003613142 | E82,E191,E438,E559,E455,E228,E374 | C00219,CE5947,C00219,CE5946,C00219,CE5926/CE5930/CE2056/CE1447/CE5534/CE5533,CE5946 | -Eicosatetraenoic acid$20-COOH-10,11-dihydro-LTB4$(5Z,8Z,11Z,14Z)-Icosatetraenoic acid; Arachidonate; Arachidonic acid; cis-5,8,11,14-Eicosatetraenoic acid$10,11-dihydro-20-trihydroxy-leukotriene B4$(5Z,8Z,11Z,14Z)-Icosatetraenoic acid; Arachidonate; Arachidonic acid; cis-5,8,11,14-Eicosatetraenoic acid$//20-dihydroxyleukotri |
| Omega-3 fatty acid metabolism | - | - | - | - | - | - |
| Fatty Acid Metabolism | 6 | 8 | 0.008822788 | E933,E898,E227,E119,E490,E203 | C00249,C01530,C01595,C00249,C01530,C00249 | nitate; Cetylic acid$Octadecanoic acid; Stearate; Stearic acid$Linoleate; Linoleic acid; (9Z,12Z)-Octadecadienoic acid; 9-cis,12-cis-Octadecadienoate; 9-cis,12-cis-Octadecadienoic acid$Hexadecanoic acid; Hexadecanoate; Hexadecylic acid; Palmitic acid; Palmitate; Cetylic acid$Octadecanoic acid; Stearate; Stearic acid$Hexadecanoic aci |
| Limonene and pinene degradation | 7 | 10 | 0.008906815 | E649,E255,E950,E3,E463,E389,E146 | C11409/C02452/C00964/C02759,C06099/C06078/C00521,C11409/C02452/C00964/C02759,C11409/C02452/C00964/C02759,C06306,C02576,C06099/C06306/C06078/C00521 | onene; (+)-(4R)-Limonene//(-)-Limonene; (-)-(S)-Limonene; (-)-(4S)-Limonene$(+)-trans-Carveol; (4S,6R)-trans-Carveol/Perillyl alcohol; (-)-Perillyl alcohol; p-Mentha-1,8-dien-7-ol; (-)-Perillylalcohol/(-)-trans-Carveol/$(+)-trans-Carveol; (4S,6R)-trans-Carveol/Perillyl alcohol; (-)-Perillyl alcohol; p-Mentha-1,8-dien-7-ol; (-)-Perillylal |
| Bile acid biosynthesis | - | - | - | - | - | - |
| Linoleate metabolism | - | - | - | - | - | - |
| Saturated fatty acids beta-oxidation | 4 | 5 | 0.017477523 | E801,E119,E933,E203 | C02990,C00249,C00249,C00249 | L-Palmitoylcarnitine$Hexadecanoic acid; Hexadecanoate; Hexadecylic acid; Palmitic acid; Palmitate; Cetylic acid$Hexadecanoic acid; Hexadecanoate; Hexadecylic acid; Palmitic acid; Palmitate; Cetylic acid$Hexadecanoic acid; Hexadecanoate; Hexadecylic acid; Palmitic acid; Palmitate; Cetylic acid |
| Xenobiotics metabolism | 4 | 5 | 0.017477523 | E119,E203,E933,E769 | C00249,C00249,C00249,C00829 | Hexadecanoic acid; Hexadecanoate; Hexadecylic acid; Palmitic acid; Palmitate; Cetylic acid$Hexadecanoic acid; Hexadecanoate; Hexadecylic acid; Palmitic acid; Palmitate; Cetylic acid$Hexadecanoic acid; Hexadecanoate; Hexadecylic acid; Palmitic acid; Palmitate; Cetylic acid$Naphthalene |

**SD 4**

Spleen

| pathway | overlap size | pathway size | p-value | overlap Empirical Compounds | overlap features | overlap features name |
|---|---|---|---|---|---|---|
| Tyrosine metabolism | 12 | 16 | 8.40266E-05 | E420,E408,E563,E335,E201,E163,E35,E584,E461,E30,E483,E360 | [illegible] | [illegible] |
| Pyrimidine metabolism | 3 | 3 | 0.009242921 | E335,E569,E127 | [illegible] | [illegible] |
| Glycine, serine, alanine and threonine me | 9 | 14 | 0.033190488 | E18,E686,E141,E366,E501,E303,E395,E58,E547 | [illegible] | [illegible] |
| Glycerophospholipid metabolism | - | - | - | - | - | - |
| Fructose and mannose metabolism | 6 | 6 | 0.002268717 | E46,E310,E361,E118,E488,E722 | [illegible] | [illegible] |
| Beta-Alanine metabolism | 6 | 6 | 0.002268717 | E18,E686,E341,E75,E547,E58 | [illegible] | [illegible] |
| Glycolysis and Gluconeogenesis | 4 | 4 | 0.002352743 | E569,E127,E178,E511 | [illegible] | [illegible] |
| Alanine and Aspartate Metabolism | 6 | 7 | 0.008906815 | E121,E341,E592,E303,E58,E547 | [illegible] | [illegible] |
| Vitamin B9 (folate) metabolism | - | - | - | - | - | - |
| Aspartate and asparagine metabolism | 5 | 7 | 0.013612301 | E335,E569,E513,E159,E30 | [illegible] | [illegible] |
| Urea cycle/amino group metabolism | 5 | 6 | 0.004201328 | E335,E502,E569,E461,E563 | [illegible] | [illegible] |
| Vitamin B3 (nicotinate and nicotinamide) | - | - | - | - | - | - |
| Pentose phosphate pathway | - | - | - | - | - | - |
| Valine, leucine and isoleucine degradatio | 3 | 3 | 0.009242921 | E335,E553,E178 | [illegible] | [illegible] |
| Aminosugars metabolism | 3 | 3 | 0.009242921 | E335,E392,E127 | [illegible] | [illegible] |
| Lysine metabolism | 3 | 3 | 0.009242921 | E335,E178,E98 | C00025,C00248,C00318 | [illegible] |
| Tryptophan metabolism | 7 | 9 | 0.011763717 | E99,E72,E207,E547,E464,E722,E58 | [illegible] | [illegible] |
| TCA cycle | 4 | 4 | 0.013108142 | E342,E336,E293,E121 | [illegible] | [illegible] |
| Carbon fixation | - | - | - | - | - | - |
| Glycosphingolipid biosynthesis - globoser | - | - | - | - | - | - |
| Fatty acid activation | - | - | - | - | - | - |
| Methionine and cysteine metabolism | 4 | 6 | 0.037139736 | E335,E30,E513,E137 | [illegible] | [illegible] |
| Butanoate metabolism | 2 | 2 | 0.03680363 | E335,E553 | [illegible] | [illegible] |
| Ascorbate (Vitamin C) and Aldarate Metal | - | - | - | - | - | - |
| Glutamate metabolism | 3 | 4 | 0.039408453 | E335,E30,E569 | C00025,C00127,C00169 | [illegible] |
| Galactose metabolism | - | - | - | - | - | - |
| Glycosphingolipid biosynthesis - neolacto | - | - | - | - | - | - |
| Keratan sulfate biosynthesis | - | - | - | - | - | - |
| O-Glycan biosynthesis | - | - | - | - | - | - |
| Propanoate metabolism | - | - | - | - | - | - |
| Glycosphingolipid biosynthesis - lactoseri | - | - | - | - | - | - |
| Blood Group Biosynthesis | - | - | - | - | - | - |
| Sialic acid metabolism | - | - | - | - | - | - |
| N-Glycan biosynthesis | 2 | 2 | 0.03680363 | E392,E127 | [illegible] | [illegible] |
| Arginine and Proline Metabolism | 2 | 2 | 0.03680363 | E335,E513 | [illegible] | [illegible] |
| Glutathione Metabolism | - | - | - | - | - | - |

**SD 4**

Kidney

| pathway | overlap size | pathway size | p-value | overlap Empirical Compounds | overlap features | overlap features name |
|---|---|---|---|---|---|---|
| Alanine and Aspartate Metabolism | 6 | 7 | 1.68E-04 | E754,E306,E77,E653,E301,E689 | [illegible] | [illegible] |
| Ascorbate (Vitamin C) and Aldarate Metal | - | - | - | - | - | - |
| Aspartate and asparagine metabolism | 8 | 15 | 0.001344425 | E567,E356,E306,E425,E77,E301,E550,E220 | [illegible] | [illegible] |
| Glycolysis and Gluconeogenesis | 5 | 7 | 0.001680531 | E689,E301,E7,E114,E306 | [illegible] | [illegible] |
| De novo fatty acid biosynthesis | 4 | 11 | 0.002520797 | E171,E245,E177,E251 | [illegible] | [illegible] |
| Vitamin A (retinol) metabolism | 3 | 9 | 0.010923452 | E97,E178,E251 | [illegible] | [illegible] |
| Purine metabolism | - | - | - | - | - | - |
| Vitamin B1 (thiamin) metabolism | 3 | 3 | 0.003529115 | E363,E301,E689 | [illegible] | [illegible] |
| Omega-3 fatty acid metabolism | 3 | 3 | 0.003529115 | E42,E301,E52 | [illegible] | [illegible] |
| Pyruvate Metabolism | - | - | - | - | - | - |
| Methionine and cysteine metabolism | - | - | - | - | - | - |
| Aminosugars metabolism | 5 | 9 | 0.008234602 | E77,E618,E306,E114,E689 | [illegible] | [illegible] |
| Arginine and Proline Metabolism | 5 | 9 | 0.008234602 | E356,E77,E567,E653,E689 | [illegible] | [illegible] |
| Heparan sulfate degradation | - | - | - | - | - | - |
| Histidine metabolism | 3 | 4 | 0.013360222 | E77,E301,E689 | [illegible] | [illegible] |
| Butanoate metabolism | 5 | 10 | 0.013444248 | E425,E77,E301,E306,E689 | [illegible] | [illegible] |
| Beta-Alanine metabolism | - | - | - | - | - | - |
| Hexose phosphorylation | 2 | 2 | 0.021258718 | E7,E114 | [illegible] | [illegible] |
| Glycerophospholipid metabolism | 7 | 19 | 0.025712125 | E7,E749,E9,E306,E52,E689,E114 | [illegible] | [illegible] |
| Sialic acid metabolism | 4 | 8 | 0.025880178 | E749,E618,E7,E114 | [illegible] | [illegible] |
| Glycine, serine, alanine and threonine me | - | - | - | - | - | - |
| Chondroitin sulfate degradation | - | - | - | - | - | - |
| Fructose and mannose metabolism | 2 | 3 | 0.04680279 | E7,E114 | [illegible] | [illegible] |
| Glyoxylate and Dicarboxylate Metabolism | - | - | - | - | - | - |
| Glutathione Metabolism | 2 | 3 | 0.04680279 | E77,E567 | [illegible] | [illegible] |
| Vitamin B9 (folate) metabolism | 3 | 5 | 0.027224603 | E77,E454,E754 | [illegible] | [illegible] |
| Glutamate metabolism | - | - | - | - | - | - |
| Leukotriene metabolism | - | - | - | - | - | - |
| Tyrosine metabolism | - | - | - | - | - | - |
| Vitamin K metabolism | - | - | - | - | - | - |
| Lysine metabolism | 3 | 6 | 0.043357701 | E356,E77,E301 | [illegible] | [illegible] |
| N-Glycan biosynthesis | 3 | 6 | 0.043357701 | E391,E7,E114 | [illegible] | [illegible] |
| Propanoate metabolism | 3 | 6 | 0.043357701 | E425,E301,E689 | [illegible] | [illegible] |
| Alkaloid biosynthesis II | 2 | 3 | 0.04680279 | E393,E301 | [illegible] | [illegible] |
| Tryptophan metabolism | 4 | 10 | 0.048903453 | E77,E301,E7,E409 | [illegible] | [illegible] |

SD9

**Heart**

| pathway | overlap size | pathway size | p-value | overlap Empirical Compounds | overlap features | overlap features name |
|---|---|---|---|---|---|---|
| De novo fatty acid biosynthesis | - | - | - | - | - | - |
| Fatty acid activation | 3 | 15 | 0.0344509 | E38,E20,E948 | C06426/C06427,C06426/C06427,C00249 | [illegible] |
| Arachidonic acid metabolism | - | - | - | - | - | - |
| Leukotriene metabolism | 3 | 9 | 0.0068902 | E944,E459,E939 | CE5947,CE5533/CE5926/CE5930/CE2056/CE1447/CE5534,CE5946 | 20-COOH-10,11-dihydro-LTB4$///20-dihydroxyleukotriene B4/11-dehydrothromboxane B2/$10,11-dihydro-20-trihydroxy-leukotriene B4 |
| Omega-3 fatty acid metabolism | 2 | 4 | 0.0086547 | E38,E20 | C06426/C06427,C06426/C06427 | [illegible] |
| Fatty Acid Metabolism | - | - | - | - | - | - |
| Limonene and pinene degradation | - | - | - | - | - | - |
| Bile acid biosynthesis | 4 | 17 | 0.009411 | E486,E370,E509,E293 | C05463/C05465,CE4874/CE1277/CE1278/CE1279,C05455/C01561,C06341/C05458/C05453/C05501/C15518/C15520/CE5530,C05499/C05445 | [illegible] |
| Linoleate metabolism | 3 | 12 | 0.0165532 | E563,E38,E20 | C14762/C14825/C14826,C06426/C06427,C06426/C06427 | [illegible] |
| Saturated fatty acids beta-oxidation | - | - | - | - | - | - |
| Xenobiotics metabolism | - | - | - | - | - | - |

SD9

**Spleen**

| pathway | overlap size | pathway size | p-value | overlap Empirical Compounds | overlap features | overlap features name |
|---|---|---|---|---|---|---|
| Tyrosine metabolism | - | - | - | - | - | - |
| Pyrimidine metabolism | 14 | 16 | 0.0005042 | E169,E223,E353,E207,E274,E23,E140,E180,E689,E470,E608,E67,E168,E537 | [illegible] | [illegible] |
| Glycine, serine, alanine and threonine metabolism | 12 | 14 | 0.0015125 | E307,E207,E123,E140,E86,E252,E223,E274,E608,E672,E125,E353 | C00037,C00164/C00109/C00349/C06002/C00232,C00065/C00740,C00143,C05519/C00188,CE0737/C00546/C00511,C00217/C05938/C00025,C00020/C00362,C00099/C00213/C00133/C00041,C01005/C02532,C00256/C00186/C00184/C01013,C05145/C01026/C01205/C02356/[illegible] | [illegible] |
| Glycerophospholipid metabolism | 15 | 19 | 0.0020166 | E495,E638,E460,E397,E123,E5,E180,E23,E261,E196,E251,E117,E125,E282,E445 | [illegible] | [illegible] |
| Fructose and mannose metabolism | 5 | 6 | 0.0491555 | E180,E262,E495,E261,E638 | [illegible] | [illegible] |
| Beta-Alanine metabolism | 6 | 6 | 0.0070582 | E169,E363,E223,E608,E125,E353 | C00402/C00049,C00135,C00217/C05938/C00025,C00099/C00213/C00133/C00041,C00256/C00186/C00184/C01013,C05145/C01026/C01205/C02356/C00334/C03284 | [illegible] |
| Glycolysis and Gluconeogenesis | 7 | 7 | 0.0033611 | E495,E180,E84,E261,E274,E125,E262 | [illegible] | [illegible] |
| Alanine and Aspartate Metabolism | 7 | 7 | 0.0033611 | E307,E169,E511,E223,E274,E608,E508 | C00037,C00402/C00049,C05520/C01045/C01042,C00217/C05938/C00025,C00020/C00362,C00099/C00213/C00133/C00041,C00122 | [illegible] |
| Vitamin B9 (folate) metabolism | 7 | 7 | 0.0033611 | E307,E21,E123,E511,E140,E223,E189 | C00037,C05928,C00065/C00740,C05520/C01045/C01042,C00143,C00217/C05938/C00025,C05928 | [illegible] |
| Aspartate and asparagine metabolism | 11 | 13 | 0.0036972 | E307,E129,E169,E194,E116,E207,E223,E274,E694,E353,E653 | C00037,C06001/C01089/C03197/C00989/C05984,C00402/C00049,C04281/C04282/C01879,C02238,C00164/C00109/C00349/C06002/C00232,C00217/C05938/C00025,C00020/C00362,C03287,C05145/C01026/C01205/C02356/C00334/C03284,C00127 | [illegible] |
| Urea cycle/amino group metabolism | 8 | 10 | 0.0211747 | E307,E169,E140,E223,E608,E694,E508,E353 | C00037,C00402/C00049,C00143,C00217/C05938/C00025,C00099/C00213/C00133/C00041,C03287,C00122,C05145/C01026/C01205/C02356/C00334/C03284 | [illegible] |
| Vitamin B3 (nicotinate and nicotinamide) metabolism | 6 | 6 | 0.0070582 | E22,E256,E169,E689,E223,E274 | C10164/C00253,C01185,C00402/C00049,C01101/C03736/C00442/C00199,C00217/C05938/C00025,C00020/C00362 | [illegible] |
| Pentose phosphate pathway | 8 | 9 | 0.0074784 | E495,E180,E689,E700,E274,E315,E288,E261 | [illegible] | [illegible] |
| Valine, leucine and isoleucine degradation | 3 | 3 | 0.0162171 | E417,E384,E37 | C00025,C00248,C00431/C00183/C00719 | [illegible] |
| Aminosugars metabolism | 3 | 3 | 0.0162171 | E417,E486,E385 | C00025,C00128,C00092/C05345/C00085/C00103/C01172/C00636/C01177/C03862/C04006/C01097/C01094/C03546/C00446/C00275/CE3075 | [illegible] |
| Lysine metabolism | 3 | 3 | 0.0162171 | E417,E375,E384 | C00025,C00318,C00248 | [illegible] |
| Tryptophan metabolism | 7 | 9 | 0.0366356 | E22,E261,E223,E274,E608,E639,E350 | C10164/C00253,C00795/C00095/C00267/C00031/C00137/C00738/C00221/C00962/C00247/C01582/C00936/C00159/C02336/C00984/C00293/C00124,C00217/C05938/C00025,C00020/C00362,C00099/C00213/C00133/C00041,C02220,C03824 | [illegible] |
| TCA cycle | 4 | 4 | 0.0299975 | E191,E508,E732,E84 | C00042/C02170,C00122,C00311/C00158/C04575,C00149 | [illegible] |
| Carbon fixation | 5 | 5 | 0.0142845 | E180,E315,E169,E495,E262 | [illegible] | [illegible] |
| Glycosphingolipid biosynthesis - globoseries | 5 | 5 | 0.0142845 | E516,E168,E23,E261,E338 | C00128,C00029/C00052,C00055,C00795/C00095/C00267/C00031/C00137/C00738/C00221/C00962/C00247/C01582/C00936/C00159/C02336/C00984/C00293/C00124,C00043/C01170/C00203 | [illegible] |
| Fatty acid activation | 5 | 7 | 0.022099 | E437,E24,E566,E198,E394 | adrn,C01530,tette6,C00712/C01712,C03242 | [illegible] |
| Methionine and cysteine metabolism | 9 | 12 | 0.0268885 | E307,E207,E123,E223,E274,E395,E36,E167,E653 | C00037,C00164/C00109/C00349/C06002/C00232,C00065/C00740,C00217/C05938/C00025,C00020/C00362,C02712,C00245,C00302,C00127 | [illegible] |
| Butanoate metabolism | 6 | 7 | 0.0271406 | E191,E207,E129,E223,E274,E353 | C00042/C02170,C00164/C00109/C00349/C06002/C00232,C06001/C01089/C03197/C00989/C05984,C00217/C05938/C00025,C00020/C00362,C05145/C01026/C01205/C02356/C00334/C03284 | [illegible] |
| Ascorbate (Vitamin C) and Aldarate Metabolism | 6 | 7 | 0.0271406 | E209,E732,E132,E570,E426,E653 | C00818,C00311/C00158/C04575,C01620,C02045,C02147,C00127 | [illegible] |
| Glutamate metabolism | 6 | 7 | 0.0271406 | E191,E207,E223,E608,E353,E653 | C00042/C02170,C00164/C00109/C00349/C06002/C00232,C00217/C05938/C00025,C00099/C00213/C00133/C00041,C05145/C01026/C01205/C02356/C00334/C03284,C00127 | [illegible] |
| Galactose metabolism | 6 | 7 | 0.0271406 | E495,E638,E180,E261,E168,E262 | [illegible] | [illegible] |
| Glycosphingolipid biosynthesis - neolactoseries | 4 | 4 | 0.0299975 | E338,E168,E516,E23 | C00043/C01170/C00203,C00029/C00052,C00128,C00055 | [illegible] |
| Keratan sulfate biosynthesis | 4 | 4 | 0.0299975 | E168,E23,E516,E338 | C00029/C00052,C00055,C00128,C00043/C01170/C00203 | [illegible] |
| O-Glycan biosynthesis | 4 | 4 | 0.0299975 | E338,E168,E516,E23 | C00043/C01170/C00203,C00029/C00052,C00128,C00055 | [illegible] |
| Propanoate metabolism | 4 | 4 | 0.0299975 | E274,E125,E207,E129 | C00020/C00362,C00256/C00186/C00184/C01013,C00164/C00109/C00349/C06002/C00232,C06001/C01089/C03197/C00989/C05984 | [illegible] |
| Glycosphingolipid biosynthesis - lactoseries | 4 | 4 | 0.0299975 | E168,E23,E516,E338 | C00029/C00052,C00055,C00128,C00043/C01170/C00203 | [illegible] |
| Blood Group Biosynthesis | 4 | 4 | 0.0299975 | E168,E23,E516,E338 | C00029/C00052,C00055,C00128,C00043/C01170/C00203 | [illegible] |
| Sialic acid metabolism | 7 | 9 | 0.0366356 | E23,E460,E123,E180,E261,E251,E516 | [illegible] | [illegible] |
| N-Glycan biosynthesis | 7 | 9 | 0.0366356 | E338,E610,E23,E180,E261,E168,E516 | [illegible] | [illegible] |
| Arginine and Proline Metabolism | - | - | - | - | - | - |
| Glutathione Metabolism | 5 | 6 | 0.0491555 | E608,E653,E307,E223,E194 | C00099/C00213/C00133/C00041,C00127,C00037,C00217/C05938/C00025,C04281/C04282/C01879 | [illegible] |

SD9

**Kidney**

| pathway | overlap size | pathway size | p-value | overlap Empirical Compounds | overlap features | overlap features name |
|---|---|---|---|---|---|---|
| Alanine and Aspartate Metabolism | 5 | 7 | 0.0341988 | E483,E3,E487,E28,E54 | C00122,C00217/C05938/C00025,C00222/C00022,C00266/C00033,C00049/C00402 | [illegible] |
| Ascorbate (Vitamin C) and Aldarate Metabolism | 8 | 8 | 4.20E-04 | E212,E64,E431,E761,E330,E499,E352,E112 | C00818,C02045,glac/C00072/C02670,C00257/C00800,C00618/C06472/C00191,C01620,C00311/C00158/C04575,CE5985 | [illegible] |
| Aspartate and asparagine metabolism | - | - | - | - | - | - |
| Glycolysis and Gluconeogenesis | 6 | 7 | 0.0060499 | E487,E592,E28,E441,E17,E444 | [illegible] | [illegible] |
| De novo fatty acid biosynthesis | - | - | - | - | - | - |
| Vitamin A (retinol) metabolism | 5 | 9 | 0.0027729 | E303,E160,E157,E277,E43 | C15492,CE5014,CE5757,retinol-cis-13/retinol-cis-11/CE5590/CE1754,C00899/C00473 | [illegible] |
| Purine metabolism | 10 | 14 | 0.0035291 | E493,E36,E431,E53,E3,E487,E575,E483,E54,E670 | C00366,C00312/C00310/C00508/C00259/C00181/C00309/C00121,glac/C00072/C02670,C00385,C00217/C05938/C00025,C00222/C00022,C00294,C00122,C00049/C00402,C01762 | [illegible] |
| Vitamin B1 (thiamin) metabolism | - | - | - | - | - | - |
| Omega-3 fatty acid metabolism | - | - | - | - | - | - |
| Pyruvate Metabolism | 5 | 5 | 0.0036972 | E444,E487,E367,E17,E28 | C00149,C00222/C00022,CE0737/C00546/C00511,C00256/C00186/C00184/C01013,C00266/C00033 | [illegible] |
| Methionine and cysteine metabolism | | | | | | |
| Aminosugars metabolism | | | | | | |
| Arginine and Proline Metabolism | | | | | | |
| Heparan sulfate degradation | | | | | | |
| Histidine metabolism | | | | | | |
| Butanoate metabolism | | | | | | |
| Beta-Alanine metabolism | 5 | 6 | 0.0140324 | E3,E17,E487,E54,E628 | C00217/C05938/C00025,C00256/C00186/C00184/C01013,C00222/C00022,C00049/C00402,C05145/C01026/C01205/C02356/C00334/C03284 | [illegible] |
| Hexose phosphorylation | - | - | - | - | - | - |
| Glycerophospholipid metabolism | - | - | - | - | - | - |
| Sialic acid metabolism | - | - | - | - | - | - |
| Glycine, serine, alanine and threonine metabolism | 8 | 13 | 0.0265524 | E487,E367,E159,E628,E3,E28,E17,E240 | C00222/C00022,CE0737/C00546/C00511,C05519/C00188,C05145/C01026/C01205/C02356/C00334/C03284,C00217/C05938/C00025,C00266/C00033,C00256/C00186/C00184/C01013,C00258 | [illegible] |
| Chondroitin sulfate degradation | 3 | 3 | 0.0267204 | E330,E592,E36 | C00618/C06472/C00191,C00795/C00221/C00095/C00267/C00137/C00738/C00962/C00247/C00984/C00031/C00936/C01582/C00159/C02336/C00293/C00124,C00312/C00310/C00508/C00259/C00181/C00309/C00121 | [illegible] |
| Fructose and mannose metabolism | 3 | 3 | 0.0267204 | E441,E115,E592 | [illegible] | [illegible] |
| Glyoxylate and Dicarboxylate Metabolism | 3 | 3 | 0.0267204 | E28,E36,E240 | C00266/C00033,C00312/C00310/C00508/C00259/C00181/C00309/C00121,C00258 | [illegible] |
| Glutathione Metabolism | 3 | 3 | 0.0267204 | E3,E109,E52 | C00217/C05938/C00025,C00097,C04281/C04282/C01879 | [illegible] |
| Vitamin B9 (folate) metabolism | 1 | 1 | 0.0399966 | E130 | C00065/C00740 | [illegible] |
| Glutamate metabolism | 4 | 5 | 0.0334426 | E3,E487,E440,E628 | C00217/C05938/C00025,C00222/C00022,C00042/C02170,C05145/C01026/C01205/C02356/C00334/C03284 | [illegible] |
| Leukotriene metabolism | 2 | 4 | 0.0388203 | E132,E247 | C00219,CE4995 | [illegible] |
| Tyrosine metabolism | 12 | 23 | 0.0390723 | E163,E487,E311,E334,E331,E517,E109,E316,E431,E3,E397,E483 | C00355,C00222/C00022,C03758,C01061/C01036,CE5277,CE2176,C00097,C02505/C02558,glac/C00072/C02670,C00217/C05938/C00025,CE5536/C01586,C00122 | [illegible] |
| Vitamin K metabolism | 1 | 1 | 0.0399966 | E134 | C02059/C01628 | [illegible] |
| Lysine metabolism | - | - | - | - | - | - |
| N-Glycan biosynthesis | - | - | - | - | - | - |
| Propanoate metabolism | - | - | - | - | - | - |
| Alkaloid biosynthesis II | - | - | - | - | - | - |
| Tryptophan metabolism | - | - | - | - | - | - |

emialdehyde/(S)-Methylmalonate semial
$AMP; Adenosine 5'-monopho
te; D-myo-Inositol 1-phosphate; 1D-myo-Inositol 1-mon

l-monophosphate; Inositol 3-monophosphate; 1L-myo-Inositol 1-phosphate; L-m

emialdehyde/Succinate
butanoate; (S)-3-Amino-isobutyrate; L-

l-monophosphate; Inositol 3-monophosphate; 1L-myo-Inositol 1-phosphate; L-m

D-myo-Inositol 4-phosphate; 1D-myo-Inositol 4-phosphate; 1D-myo-In

onophosphate$beta-Alanine; 3-Ami

-Adenosine monophosphate; Adenos

)-2-Aminobutanoate; (S)-2-Aminobut
hate; myo-Inositol 3-monophosphate; Inositol 3-monophosphate; 1L-myo-Inositol

te$(S)-3-Hydroxyisobutyrate/(R)-3-Hydroxybut

ate; Inositol 3-phosphate; 1D-myo-Inositol 3-monophosphate;
-Inositol 3-monophosphate; myo-Inositol 3-monophosphate; Inositol 3-monoph

hate/alpha-D-Hexose 1-phosphate/b

obutyrate; 4-Aminobutyric acid; gamm

: acid; Glutamate$Glycolaldehyde; Hydrox

myo-Inositol 4-phosphate; D-myo-Inositol 4-phosphate; 1D-myo-Inositol 4-

ate$adrenochrome/Hippurate; Hippuric acid;

Supplementary Table 4. Metabolic Changes due to amifostine treatment (side effects)

**TABLE 4A**

**Amifostine 50mg side effect in SD 4 for validated metabolites across all tissues**

| Name | Organ/Tissue | *p-value* | *FDR* [a] | *Fold Change* | *Log2(FC)* |
|---|---|---|---|---|---|
| PC(16:0/18:2) | Heart | 5.16E-04 | *NS* | 1.2581 | ↑ 0.3313 |
| PC(16:0/20:5) | Heart | 1.46E-03 | *NS* | 1.22 | ↑ 0.28685 |
| Arachidonoyl Thio-PC | Heart | 3.06E-03 | *NS* | 1.2112 | ↑ 0.27642 |
| PC(16:0/18:1) | Heart | 9.31E-03 | *NS* | 1.2973 | ↑ 0.3755 |
| 5'-Adenylic acid | Heart | 2.01E-02 | *NS* | 1.6784 | ↑ 0.74711 |
| PC(16:1/16:1) | Heart | 3.62E-02 | *NS* | 1.3142 | ↑ 0.3942 |
| PC(16:0/20:4) | Spleen | 4.00E-02 | *NS* | 1.1496 | ↑ 0.20108 |
| LysoPE(18:0) | Kidney | 9.80E-04 | *NS* | 0.75206 | ↓ -0.41108 |
| Glu | Kidney | 3.64E-03 | *NS* | 1.1143 | ↑ 0.15609 |
| 2-Hydroxypalmitic acid | Kidney | 4.13E-03 | *NS* | 1.4594 | ↑ 0.54533 |
| Citric Acid | Kidney | 5.11E-03 | *NS* | 3.4518 | ↑ 1.7873 |
| PC(16:0/20:4) | Kidney | 8.73E-03 | *NS* | 1.5163 | ↑ 0.60052 |
| PC(16:0/18:2) | Kidney | 1.31E-02 | *NS* | 1.4881 | ↑ 0.57346 |
| Cervonic acid | Kidney | 2.44E-02 | *NS* | 0.77093 | ↓ -0.37532 |
| PC(16:0/22:6) | Kidney | 2.91E-02 | *NS* | 1.2456 | ↑ 0.3168 |
| PI(18:0/20:4) | Kidney | 3.63E-02 | *NS* | 1.8556 | ↑ 0.8919 |
| PC(18:1/16:0) | Kidney | 4.10E-02 | *NS* | 1.1882 | ↑ 0.24875 |
| LysoPE(16:0) | Kidney | 4.31E-02 | *NS* | 0.86704 | ↓ -0.20584 |
| 14(15)-EpEDE | Kidney | 4.91E-02 | *NS* | 0.78772 | ↓ -0.34424 |
| PC(18:0/22:6) | Kidney | 4.94E-02 | *NS* | 1.3458 | ↑ 0.42849 |

*Note.* All metabolite names are validated through tandem MS.

[a] Numbers are FDR adjusted *P* values. NS = not significant (FDR, *P* value > 0.05).

**TABLE 4B**

**Amifostine 200mg side effect in SD 4 for validated metabolites across all tissues**

| Name | Organ/Tissue | *p-value* | *FDR* [a] | *Fold Change* | | *Log2(FC)* |
|---|---|---|---|---|---|---|
| PC(16:0/18:1) | Heart | 5.24E-03 | *NS* | 1.2747 | ↑ | 0.35012 |
| Cer(d18:1/20:0) | Heart | 2.98E-02 | *NS* | 0.47695 | ↓ | -1.0681 |
| PG(16:0/18:1) | Heart | 2.83E-02 | *NS* | 0.51327 | ↓ | -0.96222 |
| PG(18:1/18:1) | Heart | 2.08E-02 | *NS* | 0.55572 | ↓ | -0.84757 |
| LysoPE(18:0) | Kidney | 1.03E-03 | *NS* | 0.82492 | ↓ | -0.27767 |
| Glu | Kidney | 3.28E-02 | *NS* | 1.1272 | ↑ | 0.17274 |
| PS(16:0/20:4) | Kidney | 2.18E-02 | *NS* | 0.84246 | ↓ | -0.24732 |

*Note.* All metabolite names are validated through tandem MS.

[a] Numbers are FDR adjusted *P* values. NS = not significant (FDR, *P* value > 0.05).

**TABLE 4C**

**Amifostine 50mg side effect in SD 9 for validated metabolites across all tissues**

| Name | Organ/Tissue | *p-value* | *FDR* [a] | *Fold Change* | *Log2(FC)* |
|---|---|---|---|---|---|
| PC(16:0/18:2) | Heart | 5.40E-04 | *NS* | 1.2244 | ↑ 0.29208 |
| Arachidonoyl Thio-PC | Heart | 1.41E-02 | *NS* | 1.1389 | ↑ 0.18765 |
| PC(16:1/16:1) | Heart | 2.85E-02 | *NS* | 1.2215 | ↑ 0.28864 |
| DELTA.17-U-46619 | Heart | 2.40E-03 | *NS* | 0.73629 | ↓ -0.44165 |
| γ-Homolinolenic acid | Heart | 4.51E-02 | *NS* | 0.8078 | ↓ -0.30792 |
| LysoPC(18:0) | Heart | 4.81E-02 | *NS* | 0.72867 | ↓ -0.45666 |
| Erucamide | Heart | 2.16E-02 | *NS* | 1.176 | ↑ 0.23391 |
| PC(18:0/18:1) | Heart | 3.54E-02 | *NS* | 1.9092 | ↑ 0.93298 |
| PS(18:0/18:1) | Spleen | 1.85E-02 | *NS* | 1.1983 | ↑ 0.26101 |
| PS(16:0/20:4) | Spleen | 3.53E-02 | *NS* | 0.88142 | ↓ -0.18211 |
| PE(16:0/20:4) | Spleen | 2.33E-02 | *NS* | 0.89713 | ↓ -0.15661 |
| PC(14:0/16:0) | Spleen | 3.17E-02 | *NS* | 1.1618 | ↑ 0.21639 |
| LysoPC(22:0) | Spleen | 2.43E-02 | *NS* | 1.0872 | ↑ 0.12057 |
| LysoPE(18:0) | Kidney | 3.48E-02 | *NS* | 0.85927 | ↓ -0.21882 |
| PC(18:1/16:0) | Kidney | 1.18E-02 | *NS* | 1.1101 | ↑ 0.15064 |
| 14(15)-EpEDE | Kidney | 1.74E-02 | *NS* | 0.80136 | ↓ -0.31947 |
| PS(16:0/20:4) | Kidney | 1.27E-02 | *NS* | 1.1808 | ↑ 0.23982 |
| PC(16:0/18:0) | Kidney | 4.91E-02 | *NS* | 1.1042 | ↑ 0.143 |
| PS(18:0/22:6) | Kidney | 1.34E-02 | *NS* | 1.1979 | ↑ 0.26055 |
| Asp | Kidney | 3.58E-03 | *NS* | 0.81293 | ↓ -0.2988 |
| Carbocyclic thromboxane A2 | Kidney | 2.14E-02 | *NS* | 0.73809 | ↓ -0.43814 |
| Arachidonic acid | Kidney | 1.07E-02 | *NS* | 0.78308 | ↓ -0.35276 |
| Arachidonoyl Thio-PC | Kidney | 1.40E-02 | *NS* | 1.0897 | ↑ 0.12395 |
| PC(16:0/20:5) | Kidney | 2.41E-02 | *NS* | 1.0965 | ↑ 0.13285 |
| Phe | Kidney | 2.95E-02 | *NS* | 0.68777 | ↓ -0.53999 |

*Note.* All metabolite names are validated through tandem MS.

[a] Numbers are FDR adjusted *P* values. NS = not significant (FDR, *P* value > 0.05).

**TABLE 4D**

**Amifostine 200mg side effect in SD 9 for validated metabolites across all tissues**

| Name | Organ/Tissue | *p-value* | *FDR* [a] | *Fold Change* | | *Log2(FC)* |
|---|---|---|---|---|---|---|
| PS(18:0/18:1) | Spleen | 2.44E-03 | *NS* | 1.2932 | ↑ | 0.37091 |
| PS(18:0/18:2) | Spleen | 1.56E-02 | *NS* | 1.1701 | ↑ | 0.2266 |
| PE(16:0/20:4) | Spleen | 2.42E-02 | *NS* | 0.90067 | ↓ | -0.15093 |
| PC(16:0/18:1) | Spleen | 1.94E-02 | *NS* | 0.93569 | ↓ | -0.095905 |
| N-Ac-Phe | Spleen | 5.57E-03 | *NS* | 0.74393 | ↓ | -0.42675 |
| L-Valine | Spleen | 2.30E-02 | *NS* | 0.88045 | ↓ | -0.18369 |
| LysoPC(22:0) | Spleen | 4.85E-02 | *NS* | 1.0959 | ↑ | 0.13212 |
| SM(d18:1/18:0) | Spleen | 2.46E-02 | *NS* | 1.2121 | ↑ | 0.2775 |
| Arg | Spleen | 7.72E-04 | *NS* | 0.78418 | ↓ | -0.35074 |
| Citric Acid | Kidney | 2.05E-02 | *NS* | 1.7349 | ↑ | 0.79481 |
| PC(P-18:0/20:4) | Kidney | 9.57E-05 | 3.76E-02 | 0.78413 | ↓ | -0.35084 |
| PC(18:1/14:0) | Kidney | 3.54E-02 | *NS* | 1.1501 | ↑ | 0.20177 |
| PC(O-16:0/22:6) | Kidney | 8.92E-04 | *NS* | 0.8362 | ↓ | -0.25808 |

*Note*. All metabolite names are validated through tandem MS.

[a] Numbers are FDR adjusted *P* values. NS = not significant (FDR, *P* value > 0.05).

**TABLE 4E**

**Amifostine side effect in SD4 and SD9 for validated metabolites across all tissues**

| | | | | SD4 ami_50/Vehicle | | | | SD4 ami_200/Vehicle | | | | SD9 ami_50/Vehicle | | | | SD9 ami_200/Vehicle | | | |
|---|---|---|---|---|---|---|---|---|---|---|---|---|---|---|---|---|---|---|---|
| Matrix | Mode | mz_rt | Name | p.value | FDR | Fold.Change | log2.FC | p.value | FDR | Fold.Change | log2.FC | p.value | FDR | Fold.Change | log2.FC | p.value | FDR | Fold.Change | log2.FC |
| Heart | Positive | 758.5699_9.87 | PC(16:0/18:2) | 5.16E-04 | 9.35E-01 | 1.2581 ↑ | 0.3313 | 2.47E-01 | 8.07E-01 | 1.1056 ↑ | 0.14481 | 5.40E-04 | 6.07E-01 | 1.2244 ↑ | 0.29208 | 7.07E-01 | 9.98E-01 | 0.98332 ↓ | -0.024263 |
| Heart | Positive | 780.5537_9.7 | PC(16:0/20:5) | 1.46E-03 | 9.35E-01 | 1.22 ↑ | 0.28685 | 5.93E-01 | 9.92E-01 | 1.049 ↑ | 0.069056 | 4.60E-01 | 7.62E-01 | 1.0451 ↑ | 0.063572 | 6.52E-01 | 9.97E-01 | 0.96989 ↓ | -0.0441 |
| Heart | Positive | 784.5823_9.86 | Arachidonoyl Thio-PC | 3.06E-03 | 9.35E-01 | 1.2112 ↑ | 0.27642 | 1.89E-01 | 7.50E-01 | 1.1192 ↑ | 0.16243 | 1.41E-02 | 6.07E-01 | 1.1389 ↑ | 0.18765 | 5.80E-01 | 9.97E-01 | 0.97134 ↓ | -0.041954 |
| Heart | Positive | 760.5854_9.96 | PC(16:0/18:1) | 9.31E-03 | 9.35E-01 | 1.2973 ↑ | 0.3755 | 5.24E-03 | 5.18E-01 | 1.2747 ↑ | 0.35012 | 5.79E-01 | 8.07E-01 | 1.0391 ↑ | 0.055322 | 9.44E-02 | 9.97E-01 | 0.83565 ↓ | -0.25903 |
| Heart | Positive | 348.0715_0.45 | 5'-Adenylic acid | 2.01E-02 | 9.35E-01 | 1.6784 ↑ | 0.74711 | 1.46E-01 | 6.88E-01 | 1.3899 ↑ | 0.47497 | 1.62E-01 | 6.14E-01 | 0.77276 ↓ | -0.3719 | 2.90E-01 | 9.97E-01 | 0.83727 ↓ | -0.25623 |
| Heart | Positive | 730.5382_9.74 | PC(16:1/16:1) | 3.62E-02 | 9.35E-01 | 1.3142 ↑ | 0.3942 | 2.60E-01 | 8.27E-01 | 1.1554 ↑ | 0.2084 | 2.85E-02 | 6.07E-01 | 1.2215 ↑ | 0.28864 | 2.60E-01 | 9.97E-01 | 0.94585 ↓ | -0.080312 |
| Heart | Positive | 792.5703_9.89 | PE(18:0/22:6) | 6.91E-02 | 9.35E-01 | 1.2064 ↑ | 0.27066 | 1.65E-01 | 7.22E-01 | 1.1611 ↑ | 0.21554 | 5.65E-01 | 7.98E-01 | 1.0642 ↑ | 0.089767 | 2.42E-01 | 9.97E-01 | 0.8794 ↓ | -0.1854 |
| Heart | Positive | 762.5126_9.67 | PC(16:0/18:0) | 1.07E-01 | 9.35E-01 | 1.1988 ↑ | 0.26157 | 3.03E-01 | 8.76E-01 | 1.1218 ↑ | 0.16576 | 5.29E-01 | 7.87E-01 | 0.9441 ↓ | -0.082987 | 3.24E-01 | 9.97E-01 | 0.90227 ↓ | -0.14837 |
| Heart | Positive | 329.2477_7.48 | Cervonic acid | 1.11E-01 | 9.35E-01 | 0.68047 ↓ | -0.55541 | 2.42E-01 | 8.06E-01 | 0.76975 ↓ | -0.37754 | 9.61E-01 | 9.85E-01 | 1.0436 ↑ | 0.061564 | 1.82E-01 | 9.97E-01 | 1.299 ↑ | 0.37744 |
| Heart | Positive | 323.2528_8.15 | Methyl oleoylethanolamide | 1.34E-01 | 9.35E-01 | 0.77541 ↓ | -0.36698 | 5.87E-02 | 5.51E-01 | 0.6974 ↓ | -0.51995 | 1.07E-01 | 6.07E-01 | 0.8618 ↓ | -0.21458 | 5.64E-01 | 9.97E-01 | 1.2331 ↑ | 0.30234 |
| Heart | Positive | 331.2637_8.11 | DELTA.17-U-46619 | 1.58E-01 | 9.35E-01 | 0.83772 ↓ | -0.25546 | 3.04E-01 | 8.76E-01 | 0.87018 ↓ | -0.20062 | 2.40E-03 | 6.07E-01 | 0.73629 ↓ | -0.44165 | 4.77E-01 | 9.97E-01 | 0.94134 ↓ | -0.087213 |
| Heart | Positive | 137.047_0.47 | Hypoxanthine | 1.65E-01 | 9.35E-01 | 0.91312 ↓ | -0.13113 | 6.89E-01 | 9.92E-01 | 0.97225 ↓ | -0.040602 | 8.04E-01 | 9.10E-01 | 1.0226 ↑ | 0.032244 | 5.45E-01 | 9.97E-01 | 1.0548 ↑ | 0.076954 |
| Heart | Positive | 482.3248_7.43 | LysoPE(18:0) | 1.78E-01 | 9.35E-01 | 1.2461 ↑ | 0.31739 | 1.36E-01 | 6.71E-01 | 1.3886 ↑ | 0.47366 | 1.83E-01 | 6.17E-01 | 0.79026 ↓ | -0.3396 | 6.07E-02 | 9.97E-01 | 0.69171 ↓ | -0.53177 |
| Heart | Positive | 575.5029_10.8 | Cer(d18:1/20:0) | 2.18E-01 | 9.35E-01 | 0.64473 ↓ | -0.63323 | 2.98E-02 | 5.18E-01 | 0.47695 ↓ | -1.0681 | 9.40E-01 | 9.73E-01 | 1.2587 ↑ | 0.33193 | 2.64E-01 | 9.97E-01 | 1.2511 ↑ | 0.32316 |
| Heart | Positive | 771.5146_11.04 | PG(16:0/18:1) | 2.26E-01 | 9.35E-01 | 0.692 ↓ | -0.53116 | 2.83E-02 | 5.18E-01 | 0.51327 ↓ | -0.96222 | 7.15E-01 | 8.74E-01 | 1.4849 ↑ | 0.57036 | 1.51E-01 | 9.97E-01 | 1.5267 ↑ | 0.61039 |
| Heart | Positive | 797.5311_11.06 | PG(18:1/18:1) | 2.39E-01 | 9.35E-01 | 0.75435 ↓ | -0.40669 | 2.08E-02 | 5.18E-01 | 0.55572 ↓ | -0.84757 | 5.56E-01 | 7.97E-01 | 1.5086 ↑ | 0.59319 | 3.53E-01 | 9.97E-01 | 1.3023 ↑ | 0.38109 |
| Heart | Positive | 834.6007_9.89 | PC(22:6/18:0) | 2.63E-01 | 9.35E-01 | 1.1077 ↑ | 0.14759 | 6.22E-01 | 9.92E-01 | 1.0556 ↑ | 0.078041 | 8.79E-01 | 9.40E-01 | 1.0201 ↑ | 0.028768 | 1.98E-01 | 9.97E-01 | 0.89432 ↓ | -0.16114 |
| Heart | Positive | 342.203_9.11 | Methyl DHA | 2.72E-01 | 9.35E-01 | 0.88443 ↓ | -0.17719 | 7.87E-01 | 9.92E-01 | 1.0133 ↑ | 0.019082 | 8.29E-01 | 9.18E-01 | 1.0207 ↑ | 0.029514 | 7.25E-01 | 9.98E-01 | 0.95388 ↓ | -0.068123 |
| Heart | Positive | 991.6712_6.71 | LysoPC(16:0) | 2.89E-01 | 9.35E-01 | 1.5493 ↑ | 0.63159 | 3.03E-01 | 8.76E-01 | 1.4619 ↑ | 0.54782 | 1.09E-01 | 6.07E-01 | 0.57649 ↓ | -0.79463 | 6.63E-01 | 9.98E-01 | 1.1085 ↑ | 0.1486 |
| Heart | Positive | 357.2772_8.9 | Monoolein | 3.49E-01 | 9.37E-01 | 0.62002 ↓ | -0.6896 | 5.81E-01 | 9.92E-01 | 1.2685 ↑ | 0.3431 | 4.99E-01 | 7.76E-01 | 0.73636 ↓ | -0.44152 | 9.52E-01 | 9.98E-01 | 1.0012 ↑ | 0.0017887 |
| Heart | Positive | 204.1241_0.44 | Acetylcarnitine | 3.53E-01 | 9.37E-01 | 1.1591 ↑ | 0.21294 | 9.99E-01 | 1.00E+00 | 1.0387 ↑ | 0.054815 | 1.78E-01 | 6.17E-01 | 0.84473 ↓ | -0.24343 | 6.43E-01 | 9.97E-01 | 1.0002 ↑ | 0.00031755 |
| Heart | Positive | 793.5039_11.59 | PG(18:2) | 3.54E-01 | 9.37E-01 | 0.87509 ↓ | -0.1925 | 2.42E-01 | 8.06E-01 | 0.84751 ↓ | -0.23869 | 6.66E-01 | 8.50E-01 | 1.3006 ↑ | 0.3792 | 2.29E-01 | 9.97E-01 | 1.3558 ↑ | 0.43918 |
| Heart | Positive | 806.5694_9.78 | PC(16:0/22:6) | 3.56E-01 | 9.37E-01 | 1.0462 ↑ | 0.065137 | 9.41E-01 | 9.92E-01 | 1.0049 ↑ | 0.0070508 | 7.26E-01 | 8.78E-01 | 1.0292 ↑ | 0.041546 | 2.81E-01 | 9.97E-01 | 0.93958 ↓ | -0.089915 |
| Heart | Positive | 333.2791_8.5 | Adrenic Acid (22:4, n-6) | 3.94E-01 | 9.44E-01 | 1.2249 ↑ | 0.2927 | 6.45E-01 | 9.92E-01 | 1.176 ↑ | 0.2339 | 6.89E-02 | 6.07E-01 | 0.77551 ↓ | -0.36678 | 8.46E-01 | 9.98E-01 | 1.0489 ↑ | 0.068906 |
| Heart | Positive | 148.0049_0.43 | Glu | 4.10E-01 | 9.44E-01 | 1.0425 ↑ | 0.059994 | 7.91E-01 | 9.92E-01 | 1.0115 ↑ | 0.016534 | 9.87E-02 | 6.07E-01 | 0.93105 ↓ | -0.10307 | 6.15E-01 | 9.97E-01 | 0.98348 ↓ | -0.02403 |
| Heart | Positive | 307.2588_8.32 | γ-Homolinolenic acid | 4.69E-01 | 9.54E-01 | 0.88004 ↓ | -0.18436 | 8.47E-01 | 9.92E-01 | 0.97687 ↓ | -0.033755 | 4.51E-02 | 6.07E-01 | 0.8078 ↓ | -0.30792 | 9.60E-01 | 9.98E-01 | 1.0428 ↑ | 0.060452 |
| Heart | Positive | 118.0874_0.43 | DL-Norvaline | 5.05E-01 | 9.54E-01 | 1.0537 ↑ | 0.07548 | 8.22E-01 | 9.92E-01 | 0.93975 ↓ | -0.089652 | 2.51E-01 | 6.22E-01 | 1.1254 ↑ | 0.17049 | 1.50E-01 | 9.97E-01 | 1.1501 ↑ | 0.2017 |
| Heart | Positive | 287.2357_8.02 | N-Arachidonoyl-L-serine | 5.27E-01 | 9.54E-01 | 0.94674 ↓ | -0.078955 | 8.03E-01 | 9.92E-01 | 0.99491 ↓ | -0.0073657 | 2.15E-01 | 6.22E-01 | 0.85299 ↓ | -0.2294 | 4.70E-01 | 9.97E-01 | 0.91992 ↓ | -0.12041 |
| Heart | Positive | 379.2825_8.14 | 2-AG | 5.50E-01 | 9.55E-01 | 1.2014 ↑ | 0.26473 | 8.77E-01 | 9.92E-01 | 0.98246 ↓ | -0.02553 | 9.56E-01 | 9.82E-01 | 1.0585 ↑ | 0.081988 | 2.39E-01 | 9.97E-01 | 1.3179 ↑ | 0.39827 |
| Heart | Positive | 291.2665_8.27 | Eicosadienoic acid | 5.92E-01 | 9.55E-01 | 0.83194 ↓ | -0.26545 | 8.24E-01 | 9.92E-01 | 0.94069 ↓ | -0.088213 | 5.53E-01 | 7.95E-01 | 0.92196 ↓ | -0.11722 | 8.86E-01 | 9.98E-01 | 1.1031 ↑ | 0.14152 |
| Heart | Positive | 454.3896_6.9 | LysoPE(16:0) | 6.02E-01 | 9.55E-01 | 1.3115 ↑ | 0.39126 | 8.99E-01 | 9.92E-01 | 1.1871 ↑ | 0.24748 | 5.93E-01 | 8.15E-01 | 1.2646 ↑ | 0.33864 | 4.60E-01 | 9.97E-01 | 1.2728 ↑ | 0.34803 |
| Heart | Positive | 147.0776_0.44 | Gln | 6.18E-01 | 9.55E-01 | 1.0568 ↑ | 0.079707 | 9.90E-01 | 9.99E-01 | 0.97741 ↓ | -0.032958 | 1.54E-01 | 6.12E-01 | 0.83081 ↓ | -0.2674 | 6.52E-01 | 9.97E-01 | 1.0521 ↑ | 0.073315 |
| Heart | Positive | 162.1136_0.41 | L-Carnitine | 6.86E-01 | 9.57E-01 | 0.96072 ↓ | -0.057816 | 4.87E-01 | 9.84E-01 | 1.0913 ↑ | 0.12609 | 5.50E-01 | 7.95E-01 | 1.066 ↑ | 0.092172 | 6.28E-02 | 9.97E-01 | 1.2444 ↑ | 0.31542 |
| Heart | Positive | 524.3713_7.46 | LysoPC(18:0) | 6.97E-01 | 9.57E-01 | 1.0648 ↑ | 0.090599 | 1.93E-01 | 7.56E-01 | 1.1767 ↑ | 0.23472 | 4.81E-02 | 6.07E-01 | 0.72867 ↓ | -0.45666 | 2.83E-01 | 9.97E-01 | 0.88169 ↓ | -0.18166 |
| Heart | Positive | 400.2676_7.69 | 7α,12α-Dihydroxycholest-4-en-3-one | 7.09E-01 | 9.57E-01 | 0.99648 ↓ | -0.005088 | 3.36E-01 | 9.06E-01 | 0.77863 ↓ | -0.361 | 8.95E-01 | 9.50E-01 | 1.0957 ↑ | 0.13187 | 2.38E-01 | 9.97E-01 | 1.4677 ↑ | 0.55358 |
| Heart | Positive | 305.2484_8.02 | Arachidonic acid | 7.13E-01 | 9.57E-01 | 0.96396 ↓ | -0.052951 | 7.42E-01 | 9.92E-01 | 0.9745 ↓ | -0.037267 | 1.67E-01 | 6.17E-01 | 0.86114 ↓ | -0.21568 | 3.98E-01 | 9.97E-01 | 0.91735 ↓ | -0.12446 |
| Heart | Positive | 132.0778_0.42 | Creatine | 7.26E-01 | 9.57E-01 | 1.0272 ↑ | 0.038706 | 8.12E-01 | 9.92E-01 | 1.0234 ↑ | 0.033331 | 1.74E-01 | 6.17E-01 | 0.8643 ↓ | -0.21039 | 8.24E-01 | 9.98E-01 | 1.03 ↑ | 0.042586 |
| Heart | Positive | 313.2739_8.01 | Methyl ricinoleate | 7.56E-01 | 9.61E-01 | 0.91811 ↓ | -0.12326 | 8.02E-01 | 9.92E-01 | 0.9081 ↓ | -0.13908 | 2.96E-01 | 6.42E-01 | 1.134 ↑ | 0.18136 | 9.13E-01 | 9.98E-01 | 1.1004 ↑ | 0.13808 |
| Heart | Positive | 344.2587_8.67 | Methyl DPA | 7.82E-01 | 9.62E-01 | 0.87658 ↓ | -0.19003 | 5.80E-01 | 9.92E-01 | 0.82852 ↓ | -0.2714 | 6.26E-01 | 8.29E-01 | 0.93951 ↓ | -0.090015 | 2.92E-01 | 9.97E-01 | 1.2519 ↑ | 0.32414 |
| Heart | Positive | 338.1829_8.61 | Erucamide | 7.88E-01 | 9.62E-01 | 1.0194 ↑ | 0.027651 | 6.43E-01 | 9.92E-01 | 0.92871 ↓ | -0.10671 | 2.16E-02 | 6.07E-01 | 1.176 ↑ | 0.23391 | 6.92E-02 | 9.97E-01 | 1.186 ↑ | 0.24608 |
| Heart | Positive | 279.2321_7.69 | 9(10)-EpOME | 8.02E-01 | 9.65E-01 | 0.9603 ↓ | -0.058443 | 8.46E-01 | 9.92E-01 | 0.95757 ↓ | -0.062557 | 5.26E-01 | 7.87E-01 | 0.94477 ↓ | -0.081961 | 6.56E-01 | 9.98E-01 | 1.0568 ↑ | 0.079752 |
| Heart | Positive | 375.2612_7.63 | N-Arachidonoyl-L-alanine | 8.36E-01 | 9.70E-01 | 1.1093 ↑ | 0.1496 | 6.67E-01 | 9.92E-01 | 0.94526 ↓ | -0.081211 | 8.42E-01 | 9.23E-01 | 1.0487 ↑ | 0.068621 | 5.67E-01 | 9.97E-01 | 1.1642 ↑ | 0.21937 |
| Heart | Positive | 810.5073_9.69 | PC(18:0/18:1) | 9.17E-01 | 9.94E-01 | 0.97265 ↓ | -0.040008 | 3.13E-01 | 8.84E-01 | 0.83683 ↓ | -0.257 | 3.54E-02 | 6.07E-01 | 1.9092 ↑ | 0.93298 | 7.87E-01 | 9.98E-01 | 1.0427 ↑ | 0.060337 |
| Heart | Positive | 355.2837_7.63 | 2-Linoleoylglycerol | 9.88E-01 | 9.98E-01 | 1.044 ↑ | 0.062064 | 7.37E-01 | 9.92E-01 | 1.0242 ↑ | 0.03453 | 4.19E-01 | 7.40E-01 | 1.1393 ↑ | 0.18809 | 3.67E-01 | 9.97E-01 | 1.2483 ↑ | 0.32 |
| Heart | Positive | 353.2668_8.12 | 15,19-Hhydroxy PGE1 | 9.98E-01 | 9.99E-01 | 0.99532 ↓ | -0.0067726 | 4.73E-01 | 9.77E-01 | 0.81961 ↓ | -0.28698 | 1.64E-01 | 6.16E-01 | 1.171 ↑ | 0.22771 | 6.76E-02 | 9.97E-01 | 1.241 ↑ | 0.31147 |
| Spleen | Negative | 826.5609_10.2 | PC(16:0/20:4) | 4.00E-02 | 8.19E-01 | 1.1496 ↑ | 0.20108 | 3.12E-01 | 1.00E+00 | 1.0601 ↑ | 0.084258 | 6.02E-01 | 9.96E-01 | 0.96829 ↓ | -0.046485 | 4.61E-01 | 7.91E-01 | 1.0451 ↑ | 0.063577 |
| Spleen | Negative | 760.5127_11.91 | PS(16:0/18:1) | 5.69E-02 | 8.19E-01 | 1.1077 ↑ | 0.14763 | 1.23E-01 | 1.00E+00 | 1.086 ↑ | 0.11902 | 7.31E-02 | 9.95E-01 | 1.0924 ↑ | 0.12748 | 5.79E-02 | 5.97E-01 | 1.1237 ↑ | 0.16828 |
| Spleen | Negative | 802.5608_10.24 | PC(16:0/18:2) | 8.61E-02 | 8.19E-01 | 1.1899 ↑ | 0.25082 | 6.17E-01 | 1.00E+00 | 1.0429 ↑ | 0.060614 | 3.67E-01 | 9.96E-01 | 0.93715 ↓ | -0.093643 | 7.69E-01 | 9.19E-01 | 1.0185 ↑ | 0.026506 |
| Spleen | Negative | 810.5295_11.91 | PS(18:0/20:4) | 2.53E-01 | 8.19E-01 | 1.0591 ↑ | 0.08279 | 5.45E-01 | 1.00E+00 | 1.0361 ↑ | 0.0512 | 2.78E-01 | 9.96E-01 | 1.0523 ↑ | 0.07353 | 2.79E-01 | 6.93E-01 | 1.0505 ↑ | 0.07108 |
| Spleen | Negative | 788.5428_11.9 | PS(18:0/18:1) | 2.58E-01 | 8.19E-01 | 1.111 ↑ | 0.15186 | 9.56E-01 | 1.00E+00 | 0.9971 ↓ | -0.0041913 | 1.85E-02 | 9.68E-01 | 1.1983 ↑ | 0.26101 | 2.44E-03 | 4.34E-01 | 1.2932 ↑ | 0.37091 |
| Spleen | Negative | 714.5079_10.31 | PE(16:0/18:2) | 3.53E-01 | 8.19E-01 | 0.91791 ↓ | -0.12357 | 1.53E-01 | 1.00E+00 | 0.87601 ↓ | -0.19097 | 2.08E-01 | 9.96E-01 | 0.89293 ↓ | -0.16338 | 2.38E-01 | 6.80E-01 | 0.90439 ↓ | -0.14498 |
| Spleen | Negative | 327.2322_8.65 | Cervonic acid | 4.25E-01 | 8.19E-01 | 0.80024 ↓ | -0.32149 | 7.95E-01 | 1.00E+00 | 0.84705 ↓ | -0.23948 | 6.52E-01 | 9.96E-01 | 1.0931 ↑ | 0.12841 | 3.63E-01 | 7.33E-01 | 0.82357 ↓ | -0.28004 |
| Spleen | Negative | 520.2662_7.46 | PE(18:0/20:4) | 4.43E-01 | 8.20E-01 | 0.90123 ↓ | -0.15003 | 8.86E-01 | 1.00E+00 | 0.97777 ↓ | -0.032427 | 9.22E-01 | 9.96E-01 | 1.0102 ↑ | 0.014658 | 2.60E-01 | 6.88E-01 | 0.89999 ↓ | -0.15202 |
| Spleen | Negative | 303.2321_8.8 | ω-3 Arachidonic acid | 4.67E-01 | 8.28E-01 | 0.78929 ↓ | -0.34136 | 7.89E-01 | 1.00E+00 | 1.0294 ↑ | 0.04177 | 8.28E-01 | 9.96E-01 | 0.97082 ↓ | -0.042722 | 5.21E-01 | 8.16E-01 | 0.90363 ↓ | -0.14619 |
| Spleen | Negative | 786.5289_11.91 | PS(18:0/18:2) | 5.15E-01 | 8.36E-01 | 1.0331 ↑ | 0.046974 | 4.61E-01 | 1.00E+00 | 1.0498 ↑ | 0.070053 | 9.41E-02 | 9.96E-01 | 1.0987 ↑ | 0.13576 | 1.56E-02 | 4.72E-01 | 1.1701 ↑ | 0.2266 |
| Spleen | Negative | 464.3138_8.52 | PE(P-18:0/22:6) | 6.30E-01 | 8.62E-01 | 0.96038 ↓ | -0.058318 | 3.62E-01 | 1.00E+00 | 0.88221 ↓ | -0.1808 | 3.31E-01 | 9.96E-01 | 1.1065 ↑ | 0.14605 | 9.27E-01 | 9.75E-01 | 0.98209 ↓ | -0.026074 |
| Spleen | Negative | 295.227_7.22 | 12(13)-EpOME | 6.79E-01 | 8.74E-01 | 0.83184 ↓ | -0.26562 | 3.76E-01 | 1.00E+00 | 0.81363 ↓ | -0.29755 | 1.47E-01 | 9.96E-01 | 1.4861 ↑ | 0.57148 | 9.92E-01 | 9.96E-01 | 0.7683 ↓ | -0.38026 |
| Spleen | Negative | 782.4981_11.91 | PS(16:0/20:4) | 6.83E-01 | 8.74E-01 | 0.9795 ↓ | -0.029885 | 4.02E-01 | 1.00E+00 | 1.0534 ↑ | 0.075103 | 3.53E-02 | 9.68E-01 | 0.88142 ↓ | -0.18211 | 1.22E-01 | 6.67E-01 | 0.92376 ↓ | -0.11442 |
| Spleen | Negative | 146.0449_0.62 | Glu | 6.91E-01 | 8.78E-01 | 1.0319 ↑ | 0.045353 | 8.60E-01 | 1.00E+00 | 0.98822 ↓ | -0.017098 | 4.49E-01 | 9.96E-01 | 0.96647 ↓ | -0.049206 | 9.44E-01 | 9.82E-01 | 0.99635 ↓ | -0.0052703 |
| Spleen | Negative | 834.6139_10.43 | PS(18:0/22:6) | 7.15E-01 | 8.90E-01 | 0.98547 ↓ | -0.02111 | 1.33E-01 | 1.00E+00 | 0.92876 ↓ | -0.10663 | 2.91E-01 | 9.96E-01 | 1.0774 ↑ | 0.1076 | 2.83E-01 | 6.93E-01 | 1.0979 ↑ | 0.13479 |
| Spleen | Negative | 738.5085_10.27 | PE(16:0/20:4) | 7.52E-01 | 8.98E-01 | 0.9865 ↓ | -0.019614 | 8.80E-01 | 1.00E+00 | 1.0059 ↑ | 0.0084357 | 2.33E-02 | 9.68E-01 | 0.89713 ↓ | -0.15661 | 2.42E-02 | 5.26E-01 | 0.90067 ↓ | -0.15093 |
| Spleen | Negative | 132.0293_0.62 | Asp | 7.63E-01 | 9.01E-01 | 1.0261 ↑ | 0.037221 | 6.98E-01 | 1.00E+00 | 0.96656 ↓ | -0.049062 | 1.84E-01 | 9.96E-01 | 0.92845 ↓ | -0.10711 | 1.01E-01 | 6.66E-01 | 0.93377 ↓ | -0.098861 |

| | | | | | | | | | | | | | | | | | | | |
|---|---|---|---|---|---|---|---|---|---|---|---|---|---|---|---|---|---|---|---|
| Spleen | Negative | 464.312_7.87 | PE(P-18:0/22:6) | 7.92E-01 | 9.15E-01 | 1.007 ↑ | 0.010034 | 7.22E-01 | 1.00E+00 | 1.0974 ↑ | 0.13403 | 7.46E-01 | 9.96E-01 | 1.0638 ↑ | 0.089205 | 8.98E-01 | 9.65E-01 | 0.96833 ↓ | -0.046423 |
| Spleen | Negative | 259.0216_0.66 | Mannose 6-Phosphate | 8.81E-01 | 9.52E-01 | 0.99162 ↓ | -0.012141 | 5.19E-01 | 1.00E+00 | 0.95889 ↓ | -0.06056 | 4.22E-01 | 9.96E-01 | 0.96006 ↓ | -0.058808 | 1.59E-01 | 6.67E-01 | 1.0613 ↑ | 0.085895 |
| Spleen | Positive | 173.0806_0.85 | GLR | 1.03E-01 | 8.05E-01 | 1.3186 ↑ | 0.39902 | 2.03E-01 | 1.00E+00 | 1.1173 ↑ | 0.16003 | 6.45E-01 | 9.19E-01 | 1.0289 ↑ | 0.041114 | 3.78E-01 | 6.43E-01 | 1.0544 ↑ | 0.076426 |
| Spleen | Positive | 760.5845_10.36 | PC(16:0/18:1) | 1.63E-01 | 8.05E-01 | 0.95131 ↓ | -0.072007 | 2.16E-01 | 1.00E+00 | 0.96703 ↓ | -0.048365 | 5.62E-01 | 9.10E-01 | 0.98314 ↓ | -0.024529 | 1.94E-02 | 2.36E-01 | 0.93569 ↓ | -0.095905 |
| Spleen | Positive | 834.5996_10.25 | PC(18:0/22:6) | 1.63E-01 | 8.05E-01 | 0.94283 ↓ | -0.084926 | 4.29E-01 | 1.00E+00 | 0.97061 ↓ | -0.043035 | 2.93E-01 | 7.76E-01 | 0.95519 ↓ | -0.066146 | 2.59E-01 | 5.57E-01 | 0.95832 ↓ | -0.061414 |
| Spleen | Positive | 137.0466_0.7 | Hypoxanthine | 1.65E-01 | 8.05E-01 | 0.8846 ↓ | -0.17691 | 6.76E-01 | 1.00E+00 | 0.9749 ↓ | -0.036678 | 6.94E-01 | 9.24E-01 | 1.0312 ↑ | 0.044357 | 3.05E-01 | 5.99E-01 | 0.93249 ↓ | -0.10084 |
| Spleen | Positive | 730.544_10.2 | PC(16:1/16:1) | 1.67E-01 | 8.05E-01 | 0.93346 ↓ | -0.099333 | 2.06E-01 | 1.00E+00 | 0.9512 ↓ | -0.07218 | 5.98E-01 | 9.10E-01 | 1.0193 ↑ | 0.027559 | 1.89E-01 | 4.95E-01 | 0.94131 ↓ | -0.087259 |
| Spleen | Positive | 510.355_7.9 | LysoPC(17:0) | 1.71E-01 | 8.05E-01 | 0.83158 ↓ | -0.26608 | 8.49E-01 | 1.00E+00 | 1.045 ↑ | 0.06354 | 9.38E-01 | 9.83E-01 | 1.005 ↑ | 0.0072156 | 1.38E-01 | 4.45E-01 | 0.84877 ↓ | -0.23656 |
| Spleen | Positive | 165.0544_0.71 | N-Ac-Phe | 2.42E-01 | 8.05E-01 | 0.86745 ↓ | -0.20515 | 2.27E-01 | 1.00E+00 | 0.86717 ↓ | -0.20562 | 5.17E-01 | 8.97E-01 | 0.92663 ↓ | -0.10993 | 5.57E-03 | 1.79E-01 | 0.74393 ↓ | -0.42675 |
| Spleen | Positive | 476.2791_7.46 | LysoPE(16:0) | 3.09E-01 | 8.05E-01 | 0.89345 ↓ | -0.16254 | 2.65E-01 | 1.00E+00 | 0.88799 ↓ | -0.17138 | 3.65E-01 | 8.09E-01 | 1.0696 ↑ | 0.097115 | 5.88E-01 | 7.92E-01 | 0.96557 ↓ | -0.050545 |
| Spleen | Positive | 706.5434_10.3 | PC(14:0/16:0) | 3.66E-01 | 8.05E-01 | 1.1005 ↑ | 0.13822 | 3.49E-01 | 1.00E+00 | 0.9182 ↓ | -0.12312 | 3.17E-02 | 6.16E-01 | 1.1618 ↑ | 0.21639 | 1.55E-01 | 4.60E-01 | 1.0951 ↑ | 0.13102 |
| Spleen | Positive | 307.2567_9.03 | 11-HEDE | 3.70E-01 | 8.05E-01 | 0.79869 ↓ | -0.3243 | 8.63E-01 | 1.00E+00 | 1.0377 ↑ | 0.053411 | 5.82E-01 | 9.10E-01 | 0.92227 ↓ | -0.11674 | 1.43E-01 | 4.49E-01 | 0.7888 ↓ | -0.34226 |
| Spleen | Positive | 810.6004_10.33 | PC(18:0/18:1)? | 3.83E-01 | 8.05E-01 | 0.96396 ↓ | -0.052951 | 6.43E-01 | 1.00E+00 | 1.016 ↑ | 0.022857 | 1.18E-01 | 6.57E-01 | 0.92521 ↓ | -0.11215 | 6.37E-02 | 3.41E-01 | 0.92758 ↓ | -0.10846 |
| Spleen | Positive | 118.0867_0.67 | L-Valine | 4.39E-01 | 8.05E-01 | 0.94248 ↓ | -0.085473 | 1.62E-01 | 1.00E+00 | 0.91497 ↓ | -0.1282 | 8.26E-01 | 9.63E-01 | 0.9848 ↓ | -0.0221 | 2.30E-02 | 2.54E-01 | 0.88045 ↓ | -0.18369 |
| Spleen | Positive | 305.2477_8.8 | Arachidonic acid | 4.84E-01 | 8.05E-01 | 0.85912 ↓ | -0.21907 | 7.66E-01 | 1.00E+00 | 1.051 ↑ | 0.071748 | 6.01E-01 | 9.10E-01 | 0.93903 ↓ | -0.090763 | 3.13E-01 | 6.05E-01 | 0.85615 ↓ | -0.22407 |
| Spleen | Positive | 482.324_8.33 | Taurolite | 4.93E-01 | 8.09E-01 | 0.94309 ↓ | -0.084539 | 2.18E-01 | 1.00E+00 | 0.85035 ↓ | -0.23387 | 8.17E-02 | 6.46E-01 | 1.1687 ↑ | 0.2249 | 4.39E-01 | 6.92E-01 | 0.93822 ↓ | -0.092003 |
| Spleen | Positive | 792.5738_10.28 | DCHA-PAF | 5.51E-01 | 8.31E-01 | 0.97663 ↓ | -0.034116 | 3.20E-01 | 1.00E+00 | 0.96288 ↓ | -0.054577 | 1.26E-01 | 6.68E-01 | 1.0581 ↑ | 0.081468 | 6.45E-02 | 3.43E-01 | 1.0457 ↑ | 0.064516 |
| Spleen | Positive | 580.3577_9.64 | LysoPC(22:0) | 5.62E-01 | 8.31E-01 | 0.96384 ↓ | -0.05313 | 4.93E-01 | 1.00E+00 | 1.0751 ↑ | 0.10443 | 2.43E-02 | 6.16E-01 | 1.0872 ↑ | 0.12057 | 4.85E-02 | 3.17E-01 | 1.0959 ↑ | 0.13212 |
| Spleen | Positive | 732.5571_10.3 | PC(18:1/14:0) | 5.83E-01 | 8.43E-01 | 0.97668 ↓ | -0.034039 | 6.46E-01 | 1.00E+00 | 0.98262 ↓ | -0.025287 | 2.30E-01 | 7.49E-01 | 0.95258 ↓ | -0.070092 | 1.01E-01 | 4.04E-01 | 0.94025 ↓ | -0.088883 |
| Spleen | Positive | 729.5727_10.38 | SM(d18:1/18:1) | 5.87E-01 | 8.43E-01 | 0.97548 ↓ | -0.035822 | 7.84E-01 | 1.00E+00 | 0.989 ↓ | -0.015953 | 1.02E-01 | 6.48E-01 | 1.0775 ↑ | 0.10772 | 5.19E-02 | 3.27E-01 | 1.0916 ↑ | 0.1265 |
| Spleen | Positive | 613.1586_0.69 | GSSG | 6.01E-01 | 8.43E-01 | 0.92028 ↓ | -0.11985 | 7.83E-01 | 1.00E+00 | 1.0279 ↑ | 0.039682 | 7.63E-01 | 9.48E-01 | 0.96174 ↓ | -0.056278 | 5.35E-01 | 7.57E-01 | 0.91626 ↓ | -0.12618 |
| Spleen | Positive | 731.5945_10.43 | SM(d18:1/18:0) | 6.35E-01 | 8.57E-01 | 1.0451 ↑ | 0.063611 | 7.65E-01 | 1.00E+00 | 0.95383 ↓ | -0.068201 | 2.87E-01 | 7.73E-01 | 1.1122 ↑ | 0.15337 | 2.46E-02 | 2.55E-01 | 1.2121 ↑ | 0.2775 |
| Spleen | Positive | 508.372_7.89 | PC(P-18:0/0:0) | 6.65E-01 | 8.69E-01 | 0.97003 ↓ | -0.043899 | 8.68E-01 | 1.00E+00 | 1.1099 ↑ | 0.15045 | 8.77E-01 | 9.72E-01 | 1.0332 ↑ | 0.047172 | 5.51E-01 | 7.69E-01 | 0.92433 ↓ | -0.11352 |
| Spleen | Positive | 175.0747_0.63 | Arg | 7.74E-01 | 9.17E-01 | 0.99096 ↓ | -0.013101 | 1.72E-01 | 1.00E+00 | 0.8922 ↓ | -0.16456 | 8.06E-02 | 6.46E-01 | 0.88755 ↓ | -0.1721 | 7.72E-04 | 1.42E-01 | 0.78418 ↓ | -0.35074 |
| Spleen | Positive | 206.0677_0.64 | 3-Indolyllactic acid | 8.33E-01 | 9.35E-01 | 0.98905 ↓ | -0.015885 | 6.91E-01 | 1.00E+00 | 0.96955 ↓ | -0.04461 | 4.89E-01 | 8.85E-01 | 1.0493 ↑ | 0.069399 | 1.83E-01 | 4.91E-01 | 0.93719 ↓ | -0.093594 |
| Kidney | Negative | 480.309_8.31 | LysoPE(18:0) | 9.80E-04 | 9.60E-02 | 0.75206 ↓ | -0.41108 | 1.03E-03 | 2.45E-01 | 0.82492 ↓ | -0.27767 | 3.48E-02 | 2.51E-01 | 0.85927 ↓ | -0.21882 | 8.58E-02 | 7.47E-01 | 0.87348 ↓ | -0.19516 |
| Kidney | Negative | 146.045_0.62 | Glu | 3.64E-03 | 1.45E-01 | 1.1143 ↑ | 0.15609 | 3.28E-02 | 9.66E-01 | 1.1272 ↑ | 0.17274 | 5.35E-01 | 7.66E-01 | 0.97287 ↓ | -0.039683 | 6.34E-01 | 9.98E-01 | 0.98583 ↓ | -0.020594 |
| Kidney | Negative | 271.2269_8.59 | 2-Hydroxypalmitic acid | 4.13E-03 | 1.49E-01 | 1.4594 ↑ | 0.54533 | 2.07E-01 | 9.66E-01 | 1.1066 ↑ | 0.14612 | 8.24E-01 | 9.30E-01 | 0.98154 ↓ | -0.026879 | 3.84E-01 | 9.98E-01 | 1.0736 ↑ | 0.10241 |
| Kidney | Negative | 191.0187_0.7 | Citric Acid | 5.11E-03 | 1.74E-01 | 3.4518 ↑ | 1.7873 | 6.73E-02 | 9.66E-01 | 2.3504 ↑ | 1.2329 | 5.80E-02 | 3.07E-01 | 1.4567 ↑ | 0.54274 | 2.05E-02 | 4.77E-01 | 1.7349 ↑ | 0.79481 |
| Kidney | Negative | 826.5608_10.2 | PC(16:0/20:4) | 8.73E-03 | 2.15E-01 | 1.5163 ↑ | 0.60052 | 9.09E-02 | 9.66E-01 | 1.2127 ↑ | 0.27821 | 2.38E-01 | 5.20E-01 | 1.0954 ↑ | 0.1315 | 6.82E-01 | 9.98E-01 | 1.0746 ↑ | 0.10382 |
| Kidney | Negative | 802.5607_10.25 | PC(16:0/18:2) | 1.31E-02 | 2.44E-01 | 1.4881 ↑ | 0.57346 | 2.92E-01 | 9.66E-01 | 1.1622 ↑ | 0.21686 | 1.59E-01 | 4.41E-01 | 1.1409 ↑ | 0.19022 | 7.42E-01 | 9.98E-01 | 1.101 ↑ | 0.13885 |
| Kidney | Negative | 327.232_8.65 | Cervonic acid | 2.44E-02 | 2.90E-01 | 0.77093 ↓ | -0.37532 | 3.40E-01 | 9.66E-01 | 0.90954 ↓ | -0.13679 | 7.86E-02 | 3.33E-01 | 0.8488 ↓ | -0.2365 | 6.16E-01 | 9.98E-01 | 1.0583 ↑ | 0.081769 |
| Kidney | Negative | 850.5611_10.15 | PC(16:0/22:6) | 2.91E-02 | 2.99E-01 | 1.2456 ↑ | 0.3168 | 3.62E-01 | 9.66E-01 | 1.0922 ↑ | 0.12719 | 1.40E-01 | 4.15E-01 | 1.0939 ↑ | 0.12949 | 7.39E-01 | 9.98E-01 | 1.0409 ↑ | 0.05787 |
| Kidney | Negative | 886.6084_10.37 | PI(18:0/20:4) | 3.63E-02 | 3.03E-01 | 1.8556 ↑ | 0.8919 | 3.08E-01 | 9.66E-01 | 1.27 ↑ | 0.34486 | 8.07E-01 | 9.21E-01 | 0.97019 ↓ | -0.043657 | 4.59E-01 | 9.98E-01 | 1.0532 ↑ | 0.074831 |
| Kidney | Negative | 804.5756_10.35 | PC(18:1/16:0) | 4.10E-02 | 3.07E-01 | 1.1882 ↑ | 0.24875 | 1.38E-01 | 9.66E-01 | 1.1061 ↑ | 0.14549 | 1.18E-02 | 1.96E-01 | 1.1101 ↑ | 0.15064 | 2.50E-01 | 9.98E-01 | 1.0689 ↑ | 0.096158 |
| Kidney | Negative | 452.2776_7.48 | LysoPE(16:0) | 4.31E-02 | 3.13E-01 | 0.86704 ↓ | -0.20584 | 8.33E-02 | 9.66E-01 | 0.91126 ↓ | -0.13407 | 1.71E-01 | 4.56E-01 | 0.92278 ↓ | -0.11595 | 7.52E-01 | 9.98E-01 | 0.98397 ↓ | -0.023318 |
| Kidney | Negative | 303.2321_8.79 | 14(15)-EpEDE | 4.91E-02 | 3.19E-01 | 0.78772 ↓ | -0.34424 | 7.19E-01 | 9.77E-01 | 0.96238 ↓ | -0.055323 | 1.74E-02 | 2.19E-01 | 0.80136 ↓ | -0.31947 | 5.59E-01 | 9.98E-01 | 0.96071 ↓ | -0.05782 |
| Kidney | Negative | 878.5919_10.26 | PC(18:0/22:6) | 4.94E-02 | 3.19E-01 | 1.3458 ↑ | 0.42849 | 5.11E-01 | 9.66E-01 | 1.0941 ↑ | 0.1298 | 1.26E-01 | 4.03E-01 | 1.1689 ↑ | 0.22519 | 8.06E-01 | 9.98E-01 | 1.0673 ↑ | 0.093964 |
| Kidney | Negative | 781.4888_11.91 | PS(16:0/20:4) | 9.32E-02 | 3.87E-01 | 0.86459 ↓ | -0.20992 | 2.18E-02 | 9.66E-01 | 0.84246 ↓ | -0.24732 | 1.27E-02 | 1.99E-01 | 1.1808 ↑ | 0.23982 | 3.15E-01 | 9.98E-01 | 1.0794 ↑ | 0.11027 |
| Kidney | Negative | 464.3138_8.52 | PE(P-18:0/22:6) | 1.96E-01 | 4.85E-01 | 0.86057 ↓ | -0.21664 | 2.00E-01 | 9.66E-01 | 0.90063 ↓ | -0.15099 | 9.67E-01 | 9.91E-01 | 0.98366 ↓ | -0.02377 | 6.20E-01 | 9.98E-01 | 1.0539 ↑ | 0.075753 |
| Kidney | Negative | 790.5385_10.3 | PE(18:0/22:6) | 2.64E-01 | 5.35E-01 | 0.86625 ↓ | -0.20715 | 4.66E-01 | 9.66E-01 | 0.93951 ↓ | -0.090027 | 3.32E-01 | 6.08E-01 | 1.0881 ↑ | 0.12176 | 8.62E-01 | 9.98E-01 | 1.0394 ↑ | 0.055701 |
| Kidney | Negative | 806.5851_10.39 | PC(16:0/18:0) | 4.51E-01 | 6.70E-01 | 1.0709 ↑ | 0.098806 | 6.26E-01 | 9.67E-01 | 1.0326 ↑ | 0.046301 | 4.91E-02 | 2.85E-01 | 1.1042 ↑ | 0.143 | 6.07E-01 | 9.98E-01 | 1.0277 ↑ | 0.039425 |
| Kidney | Negative | 747.5197_10.26 | PG(16:0/18:1) | 4.90E-01 | 7.05E-01 | 0.94616 ↓ | -0.07985 | 9.07E-01 | 9.90E-01 | 0.99417 ↓ | -0.008438 | 1.41E-01 | 4.15E-01 | 1.0872 ↑ | 0.12067 | 4.69E-01 | 9.98E-01 | 1.0485 ↑ | 0.068392 |
| Kidney | Negative | 834.5288_11.91 | PS(18:0/22:6) | 4.94E-01 | 7.08E-01 | 0.94944 ↓ | -0.074847 | 6.10E-01 | 9.67E-01 | 0.96447 ↓ | -0.052194 | 1.34E-02 | 2.01E-01 | 1.1979 ↑ | 0.26055 | 4.89E-01 | 9.98E-01 | 1.0451 ↑ | 0.063578 |
| Kidney | Negative | 192.0654_3.15 | Phenaceturic acid | 5.46E-01 | 7.49E-01 | 1.1016 ↑ | 0.13965 | 4.91E-01 | 9.66E-01 | 1.2544 ↑ | 0.32697 | 3.02E-01 | 5.82E-01 | 1.4638 ↑ | 0.54969 | 9.70E-01 | 9.98E-01 | 0.86383 ↓ | -0.21118 |
| Kidney | Negative | 132.0292_0.62 | Asp | 6.67E-01 | 8.25E-01 | 0.97955 ↓ | -0.029803 | 9.56E-02 | 9.66E-01 | 0.92226 ↓ | -0.11676 | 3.58E-03 | 1.73E-01 | 0.81293 ↓ | -0.2988 | 3.14E-01 | 9.98E-01 | 0.95788 ↓ | -0.062076 |
| Kidney | Negative | 810.5296_11.92 | PS(18:0/20:4) | 6.97E-01 | 8.43E-01 | 0.98844 ↓ | -0.01677 | 8.19E-01 | 9.87E-01 | 0.98962 ↓ | -0.015055 | 6.14E-02 | 3.09E-01 | 1.1594 ↑ | 0.21332 | 4.57E-01 | 9.98E-01 | 1.0612 ↑ | 0.085754 |
| Kidney | Negative | 329.2477_9.03 | Carbocyclic thromboxane A2 | 8.19E-01 | 9.14E-01 | 0.9339 ↓ | -0.098664 | 2.82E-01 | 9.66E-01 | 1.2425 ↑ | 0.31326 | 2.14E-02 | 2.30E-01 | 0.73809 ↓ | -0.43814 | 9.46E-01 | 9.98E-01 | 1.0718 ↑ | 0.10007 |
| Kidney | Negative | 524.2986_12.01 | PS(18:0/0:0) | 9.20E-01 | 9.62E-01 | 0.98215 ↓ | -0.025982 | 6.59E-01 | 9.69E-01 | 0.95194 ↓ | -0.071059 | 2.79E-01 | 5.61E-01 | 0.89602 ↓ | -0.15839 | 9.22E-01 | 9.98E-01 | 1.0033 ↑ | 0.0048172 |
| Kidney | Negative | 568.2675_7.42 | LysoPC(18:0) | 9.72E-01 | 9.88E-01 | 0.99276 ↓ | -0.010481 | 6.64E-01 | 9.72E-01 | 1.058 ↑ | 0.08139 | 8.47E-01 | 9.37E-01 | 0.99837 ↓ | -0.0023603 | 1.84E-01 | 9.65E-01 | 1.1924 ↑ | 0.25389 |
| Kidney | Positive | 782.5704_10.25 | PC(18:0/18:1) | 8.95E-02 | 3.85E-01 | 1.1456 ↑ | 0.19611 | 5.52E-02 | 9.98E-01 | 1.1623 ↑ | 0.21704 | 2.76E-01 | 8.23E-01 | 1.0426 ↑ | 0.060253 | 6.17E-01 | 9.10E-01 | 1.0322 ↑ | 0.045722 |
| Kidney | Positive | 303.2307_8.38 | Eicosapentaenoic acid | 1.17E-01 | 4.08E-01 | 0.77409 ↓ | -0.36943 | 2.46E-01 | 9.98E-01 | 0.84297 ↓ | -0.24644 | 6.46E-01 | 9.43E-01 | 0.97238 ↓ | -0.040409 | 9.94E-01 | 9.98E-01 | 1.0239 ↑ | 0.034107 |
| Kidney | Positive | 730.545_10.19 | PC(16:1/16:1) | 1.46E-01 | 4.24E-01 | 1.1641 ↑ | 0.21924 | 1.18E-01 | 9.98E-01 | 1.0852 ↑ | 0.11801 | 7.90E-02 | 5.60E-01 | 1.0883 ↑ | 0.12202 | 7.01E-01 | 9.20E-01 | 1.0169 ↑ | 0.024152 |
| Kidney | Positive | 305.2473_8.78 | Arachidonic acid | 1.48E-01 | 4.26E-01 | 0.77661 ↓ | -0.36473 | 9.51E-01 | 9.99E-01 | 0.99592 ↓ | -0.0059039 | 1.07E-02 | 4.04E-01 | 0.78308 ↓ | -0.35276 | 3.38E-01 | 8.61E-01 | 0.94236 ↓ | -0.085644 |
| Kidney | Positive | 794.5986_10.27 | PC(P-18:0/20:4) | 1.77E-01 | 4.44E-01 | 0.86766 ↓ | -0.2048 | 4.55E-01 | 9.98E-01 | 0.93697 ↓ | -0.093927 | 1.66E-01 | 7.31E-01 | 0.93657 ↓ | -0.094536 | 9.57E-05 | 3.76E-02 | 0.78413 ↓ | -0.35084 |
| Kidney | Positive | 732.5564_10.31 | PC(18:1/14:0) | 1.82E-01 | 4.52E-01 | 1.2011 ↑ | 0.26431 | 7.18E-02 | 9.98E-01 | 1.17 ↑ | 0.22651 | 8.50E-02 | 5.78E-01 | 1.1036 ↑ | 0.1422 | 3.54E-02 | 8.49E-01 | 1.1501 ↑ | 0.20177 |
| Kidney | Positive | 784.5822_10.28 | Arachidonoyl Thio-PC | 2.43E-01 | 5.06E-01 | 1.1155 ↑ | 0.15764 | 7.82E-02 | 9.98E-01 | 1.1302 ↑ | 0.17663 | 1.40E-02 | 4.04E-01 | 1.0897 ↑ | 0.12395 | 5.11E-01 | 8.83E-01 | 1.0416 ↑ | 0.058805 |
| Kidney | Positive | 792.59_10.26 | PC(O-16:0/22:6) | 2.71E-01 | 5.29E-01 | 0.90059 ↓ | -0.15107 | 4.86E-01 | 9.98E-01 | 0.94557 ↓ | -0.080738 | 7.06E-01 | 9.43E-01 | 0.98607 ↓ | -0.020245 | 8.92E-04 | 7.25E-02 | 0.8362 ↓ | -0.25808 |
| Kidney | Positive | 510.3542_7.88 | LysoPC(O-18:0) | 3.46E-01 | 5.83E-01 | 0.80998 ↓ | -0.30403 | 9.84E-01 | 9.99E-01 | 1.0274 ↑ | 0.039053 | 3.55E-01 | 8.36E-01 | 0.90799 ↓ | -0.13925 | 5.94E-01 | 9.09E-01 | 1.0835 ↑ | 0.11569 |
| Kidney | Positive | 780.5565_10.15 | PC(16:0/20:5) | 4.72E-01 | 6.64E-01 | 1.0496 ↑ | 0.069774 | 9.85E-01 | 9.99E-01 | 1.0005 ↑ | 0.00078005 | 2.41E-02 | 4.61E-01 | 1.0965 ↑ | 0.13285 | 1.56E-01 | 8.49E-01 | 1.0547 ↑ | 0.07685 |
| Kidney | Positive | 166.0862_1.11 | Phe | 5.90E-01 | 7.50E-01 | 0.92122 ↓ | -0.11837 | 9.88E-01 | 9.99E-01 | 1.0878 ↑ | 0.12136 | 2.95E-02 | 4.78E-01 | 0.68777 ↓ | -0.53999 | 9.25E-01 | 9.75E-01 | 0.98175 ↓ | -0.026569 |
| Kidney | Positive | 731.5898_10.4 | Sphingomyelin | 7.16E-01 | 8.32E-01 | 0.96784 ↓ | -0.047152 | 8.36E-01 | 9.99E-01 | 1.0016 ↑ | 0.0022645 | 6.51E-01 | 9.43E-01 | 1.0348 ↑ | 0.049348 | 7.91E-01 | 9.46E-01 | 1.0231 ↑ | 0.032973 |
| Kidney | Positive | 137.0462_0.72 | Hypoxanthine | 7.41E-01 | 8.39E-01 | 0.97365 ↓ | -0.038532 | 4.41E-01 | 9.98E-01 | 1.0854 ↑ | 0.11823 | 3.48E-01 | 8.36E-01 | 0.95647 ↓ | -0.064208 | 5.05E-01 | 8.83E-01 | 1.0267 ↑ | 0.037967 |
| Kidney | Positive | 123.0415_0.69 | Niacinamide | 8.51E-01 | 9.01E-01 | 0.97975 ↓ | -0.029519 | 5.12E-01 | 9.98E-01 | 1.0901 ↑ | 0.12451 | 4.81E-01 | 8.78E-01 | 0.96316 ↓ | -0.054146 | 1.71E-01 | 8.49E-01 | 1.0861 ↑ | 0.1191 |
| Kidney | Positive | 518.3202_7.49 | LysoPC(16:0) | 9.15E-01 | 9.44E-01 | 1.0229 ↑ | 0.032597 | 3.89E-01 | 9.98E-01 | 1.1371 ↑ | 0.18538 | 6.01E-01 | 9.36E-01 | 0.96775 ↓ | -0.047297 | 1.95E-01 | 8.49E-01 | 1.0968 ↑ | 0.13331 |

**TABLE 5**

**Amifostine protection effect in SD4 and SD9 for validated metabolites across all tissues**

| Name | Matrix | Mode | Recovery Effect Indicator SD 4 50mg | Recovery Effect Indicator SD 4 200mg | Recovery Effect Indicator SD 9 50mg | Recovery Effect Indicator SD 9 200mg | Recovery Effect Indicator II SD 4 50mg | Recovery Effect Indicator II SD 4 200mg | Recovery Effect Indicator II SD 9 50mg | Recovery Effect Indicator II SD 9 200mg | SD4 Radiation p-value | SD4 Radiation FDR | SD4 Radiation Fold Change | SD4 Radiation Log2(FC) | SD4 Amifostine 50mg p-value | SD4 Amifostine 50mg FDR | SD4 Amifostine 50mg Fold Change | SD4 Amifostine 50mg Log2(FC) | SD4 Amifostine 200mg p-value | SD4 Amifostine 200mg FDR | SD4 Amifostine 200mg Fold Change | SD4 Amifostine 200mg Log2(FC) |
|---|---|---|---|---|---|---|---|---|---|---|---|---|---|---|---|---|---|---|---|---|---|---|
| PC(16:0/18:2) | Heart | Positive | * | - | - | * | x | x | - | - | 2.97E-04 | 5.20E-03 | 0.7808 | -0.3570 | 1.49E-01 | 8.09E-01 | 1.1346 | 0.1821 | 6.79E-01 | 9.85E-01 | 0.9783 | -0.0317 |
| PC(16:0/20:5) | Heart | Positive | - | - | * | * | - | - | - | - | 1.17E-02 | 7.52E-02 | 0.8609 | -0.2161 | 5.58E-01 | 9.96E-01 | 0.9721 | -0.0408 | 3.07E-01 | 9.85E-01 | 0.9466 | -0.0791 |
| Arachidonoyl Thio-PC | Heart | Positive | * | - | - | * | - | - | - | - | 1.77E-01 | 4.61E-01 | 0.9235 | -0.1148 | 3.35E-01 | 9.19E-01 | 1.0842 | 0.1167 | 1.20E-02 | 9.85E-01 | 0.8672 | -0.2057 |
| PC(16:0/18:1) | Heart | Positive | * | * | - | - | - | - | - | - | 3.48E-01 | 6.40E-01 | 0.9128 | -0.1316 | 1.01E-01 | 7.87E-01 | 1.1058 | 0.1451 | 9.89E-01 | 9.97E-01 | 1.0068 | 0.0098 |
| 5'-Adenylic acid | Heart | Positive | * | * | * | * | - | - | - | - | 1.10E-01 | 3.48E-01 | 1.3881 | 0.4731 | 1.20E-01 | 7.91E-01 | 0.7360 | -0.4423 | 4.27E-01 | 9.85E-01 | 0.9070 | -0.1408 |
| PC(16:1/16:1) | Heart | Positive | * | * | * | - | x | x | - | - | 1.34E-03 | 1.46E-02 | 0.7520 | -0.4113 | 4.10E-01 | 9.83E-01 | 1.1319 | 0.1788 | 1.42E-01 | 9.85E-01 | 1.1297 | 0.1760 |
| PE(18:0/22:6) | Heart | Positive | * | - | * | * | x | x | - | - | 5.45E-04 | 7.79E-03 | 1.4382 | 0.5243 | 2.59E-01 | 8.90E-01 | 0.9326 | -0.1006 | 9.17E-01 | 9.91E-01 | 1.0036 | 0.0052 |
| PC(16:0/18:0) | Heart | Positive | * | * | - | * | x | x | - | - | 4.71E-03 | 4.02E-02 | 1.4114 | 0.4971 | 1.34E-01 | 7.91E-01 | 0.9255 | -0.1117 | 7.47E-02 | 9.85E-01 | 0.9087 | -0.1382 |
| Cervonic acid | Heart | Positive | * | * | - | * | x | x | - | - | 7.94E-05 | 2.58E-03 | 0.3276 | -1.6102 | 2.95E-01 | 8.95E-01 | 1.2547 | 0.3273 | 7.90E-01 | 9.86E-01 | 1.0342 | 0.0485 |
| Methyl oleoylethanolamide | Heart | Positive | * | * | * | * | x | x | - | - | 3.89E-07 | 5.44E-04 | 0.3053 | -1.7118 | 9.23E-01 | 9.96E-01 | 1.0005 | 0.0006 | 3.97E-02 | 9.85E-01 | 1.7711 | 0.8246 |
| DELTA.17-U-46619 | Heart | Positive | - | * | - | * | - | - | - | - | 3.67E-02 | 1.64E-01 | 0.7180 | -0.4780 | 8.13E-01 | 9.96E-01 | 0.9465 | -0.0794 | 7.58E-01 | 9.85E-01 | 1.1641 | 0.2192 |
| Hypoxanthine | Heart | Positive | * | * | * | * | - | - | - | - | 3.27E-01 | 6.26E-01 | 1.0834 | 0.1155 | 3.81E-01 | 9.62E-01 | 0.9336 | -0.0992 | 1.31E-01 | 9.85E-01 | 0.8549 | -0.2261 |
| LysoPE(18:0) | Heart | Positive | * | * | * | * | - | - | - | - | 1.91E-01 | 4.83E-01 | 1.4209 | 0.5068 | 6.18E-01 | 9.96E-01 | 0.9771 | -0.0335 | 3.32E-01 | 9.85E-01 | 0.7852 | -0.3488 |
| Cer(d18:1/20:0) | Heart | Positive | - | - | * | * | - | - | - | - | 9.87E-01 | 9.94E-01 | 0.9336 | -0.0992 | 2.17E-02 | 7.87E-01 | 0.6261 | -0.6756 | 5.63E-01 | 9.85E-01 | 0.9299 | -0.1049 |
| PG(16:0/18:1) | Heart | Positive | * | * | - | * | - | - | - | - | 5.54E-01 | 7.83E-01 | 1.0782 | 0.1087 | 1.29E-02 | 7.87E-01 | 0.6449 | -0.6329 | 2.02E-01 | 9.85E-01 | 0.8335 | -0.2628 |
| PG(18:1/18:1) | Heart | Positive | * | * | - | * | - | - | - | - | 6.54E-01 | 8.46E-01 | 1.0541 | 0.0760 | 1.06E-01 | 7.87E-01 | 0.7883 | -0.3432 | 4.16E-01 | 9.85E-01 | 0.8972 | -0.1566 |
| PC(22:6/18:0) | Heart | Positive | * | - | * | * | x | x | - | - | 2.57E-03 | 2.45E-02 | 1.3541 | 0.4374 | 1.38E-01 | 7.91E-01 | 0.9273 | -0.1089 | 4.99E-01 | 9.85E-01 | 1.0310 | 0.0440 |
| Methyl DHA | Heart | Positive | * | - | * | - | - | - | - | - | 6.94E-01 | 8.63E-01 | 0.9757 | -0.0355 | 5.42E-01 | 9.96E-01 | 1.0918 | 0.1267 | 3.72E-01 | 9.85E-01 | 0.9039 | -0.1458 |
| LysoPC(16:0) | Heart | Positive | - | - | * | - | - | - | - | - | 5.36E-01 | 7.72E-01 | 0.8188 | -0.2884 | 5.66E-01 | 9.96E-01 | 0.8786 | -0.1867 | 4.49E-01 | 9.85E-01 | 0.8215 | -0.2837 |
| Monoolein | Heart | Positive | - | - | * | * | - | - | - | - | 8.14E-02 | 2.82E-01 | 0.4248 | -1.2351 | 2.65E-01 | 8.90E-01 | 0.7076 | -0.4991 | 4.85E-01 | 9.85E-01 | 0.8217 | -0.2834 |
| Acetylcarnitine | Heart | Positive | * | * | * | * | - | - | - | - | 1.36E-01 | 3.94E-01 | 1.3238 | 0.4046 | 5.34E-01 | 9.96E-01 | 0.8893 | -0.1692 | 2.51E-01 | 9.85E-01 | 0.8042 | -0.3144 |
| PG(18:2) | Heart | Positive | * | * | - | * | - | - | - | - | 1.32E-01 | 3.88E-01 | 1.2203 | 0.2872 | 9.88E-02 | 7.87E-01 | 0.7996 | -0.3227 | 2.68E-01 | 9.85E-01 | 0.8698 | -0.2012 |
| PC(16:0/22:6) | Heart | Positive | * | * | * | * | x | x | - | - | 5.41E-03 | 4.51E-02 | 1.1827 | 0.2421 | 6.09E-02 | 7.87E-01 | 0.9090 | -0.1376 | 5.80E-01 | 9.85E-01 | 0.9750 | -0.0365 |
| Adrenic Acid (22:4, n-6) | Heart | Positive | * | * | * | * | - | - | - | - | 2.32E-02 | 1.20E-01 | 0.5632 | -0.8283 | 8.53E-01 | 9.96E-01 | 1.0071 | 0.0102 | 6.10E-01 | 9.85E-01 | 1.2391 | 0.3093 |
| Glu | Heart | Positive | * | * | * | - | - | - | - | - | 3.39E-01 | 6.33E-01 | 1.0395 | 0.0558 | 6.34E-01 | 9.96E-01 | 0.9837 | -0.0237 | 2.02E-01 | 9.85E-01 | 0.9318 | -0.1020 |
| γ-Homolinolenic acid | Heart | Positive | - | * | * | * | x | x | - | - | 3.87E-04 | 6.27E-03 | 0.5248 | -0.9300 | 5.62E-01 | 9.96E-01 | 0.9460 | -0.0801 | 7.76E-02 | 9.85E-01 | 1.5055 | 0.5902 |
| DL-Norvaline | Heart | Positive | * | * | * | - | - | - | - | - | 2.02E-02 | 1.10E-01 | 0.6838 | -0.5485 | 2.46E-01 | 8.90E-01 | 1.1285 | 0.1744 | 2.43E-02 | 9.85E-01 | 1.3294 | 0.4108 |
| N-Arachidonoyl-L-serine | Heart | Positive | * | * | - | - | - | - | - | - | 9.35E-01 | 9.74E-01 | 1.0052 | 0.0075 | 3.30E-01 | 9.14E-01 | 0.8854 | -0.1756 | 2.17E-01 | 9.85E-01 | 0.8268 | -0.2745 |
| 2-AG | Heart | Positive | * | * | - | * | x | x | - | - | 1.85E-03 | 1.89E-02 | 0.3498 | -1.5154 | 2.17E-01 | 8.83E-01 | 1.6575 | 0.7290 | 2.24E-01 | 9.85E-01 | 1.5356 | 0.6188 |
| Eicosadienoic acid | Heart | Positive | * | * | - | * | x | x | - | - | 2.47E-04 | 4.61E-03 | 0.3641 | -1.4577 | 8.09E-01 | 9.96E-01 | 1.1897 | 0.2506 | 2.86E-01 | 9.85E-01 | 1.1410 | 0.1903 |
| LysoPE(16:0) | Heart | Positive | * | * | * | - | x | x | - | - | 5.53E-03 | 4.53E-02 | 0.4320 | -1.2109 | 1.06E-01 | 7.87E-01 | 1.5037 | 0.5885 | 6.35E-01 | 9.85E-01 | 1.6316 | 0.7063 |
| Gln | Heart | Positive | * | * | - | * | - | - | - | - | 2.52E-02 | 1.27E-01 | 1.5181 | 0.6022 | 2.23E-01 | 8.83E-01 | 0.8280 | -0.2723 | 3.50E-02 | 9.85E-01 | 0.6852 | -0.5455 |
| L-Carnitine | Heart | Positive | - | - | * | - | - | - | - | - | 1.97E-01 | 4.89E-01 | 0.8969 | -0.1570 | 1.33E-01 | 7.91E-01 | 0.8805 | -0.1836 | 6.34E-01 | 9.85E-01 | 0.9750 | -0.0366 |
| LysoPC(18:0) | Heart | Positive | * | * | - | - | - | - | - | - | 2.57E-01 | 5.56E-01 | 1.1849 | 0.2448 | 6.87E-01 | 9.96E-01 | 0.9479 | -0.0773 | 1.60E-01 | 9.85E-01 | 0.8006 | -0.3208 |
| 7α,12α-Dihydroxycholest-4-en-3-one | Heart | Positive | * | * | - | * | x | x | - | - | 8.37E-05 | 2.60E-03 | 0.2342 | -2.0941 | 2.11E-01 | 8.79E-01 | 1.9744 | 0.9815 | 9.07E-02 | 9.85E-01 | 1.4633 | 0.5492 |
| Arachidonic acid | Heart | Positive | - | - | - | - | - | - | - | - | 8.78E-01 | 9.46E-01 | 0.9930 | -0.0102 | 6.75E-01 | 9.96E-01 | 0.9470 | -0.0786 | 2.49E-01 | 9.85E-01 | 0.8434 | -0.2458 |
| Creatine | Heart | Positive | * | * | - | * | - | - | - | - | 3.24E-01 | 6.25E-01 | 1.1094 | 0.1498 | 6.53E-02 | 7.87E-01 | 0.8132 | -0.2983 | 7.59E-02 | 9.85E-01 | 0.8136 | -0.2976 |
| Methyl ricinoleate | Heart | Positive | * | * | * | - | x | x | - | - | 5.91E-04 | 8.20E-03 | 0.3962 | -1.3357 | 6.02E-02 | 7.87E-01 | 1.5193 | 0.6034 | 2.64E-01 | 9.85E-01 | 1.2354 | 0.3050 |
| Methyl DPA | Heart | Positive | * | * | - | * | x | x | - | - | 2.11E-03 | 2.09E-02 | 0.3701 | -1.4342 | 3.54E-01 | 9.39E-01 | 1.1649 | 0.2203 | 7.09E-01 | 9.85E-01 | 1.1461 | 0.1967 |
| Erucamide | Heart | Positive | * | * | - | - | - | - | - | - | 1.56E-02 | 9.13E-02 | 0.6584 | -0.6031 | 5.14E-02 | 7.87E-01 | 1.2383 | 0.3084 | 7.45E-01 | 9.85E-01 | 1.1205 | 0.1641 |
| 9(10)-EpOME | Heart | Positive | * | * | * | * | x | x | - | - | 1.70E-03 | 1.76E-02 | 0.5273 | -0.9233 | 1.50E-01 | 8.09E-01 | 1.1791 | 0.2377 | 1.17E-01 | 9.85E-01 | 1.3878 | 0.4728 |
| N-Arachidonoyl-L-alanine | Heart | Positive | * | * | * | * | x | x | - | - | 1.07E-04 | 3.02E-03 | 0.2425 | -2.0438 | 1.23E-01 | 7.91E-01 | 2.1184 | 1.0830 | 8.39E-02 | 9.85E-01 | 1.9303 | 0.9488 |
| PC(18:0/18:1) | Heart | Positive | * | - | - | * | - | - | - | - | 6.53E-01 | 8.45E-01 | 1.1733 | 0.2305 | 6.54E-01 | 9.96E-01 | 0.9936 | -0.0093 | 6.72E-01 | 9.85E-01 | 1.0136 | 0.0195 |
| 2-Linoleoylglycerol | Heart | Positive | * | * | * | * | x | x | - | - | 5.98E-05 | 2.28E-03 | 0.4460 | -1.1650 | 3.72E-02 | 7.87E-01 | 1.4199 | 0.5058 | 2.91E-01 | 9.85E-01 | 1.3676 | 0.4516 |
| 15,19-Hhydroxy PGE1 | Heart | Positive | - | * | * | - | - | - | - | - | 2.46E-01 | 5.47E-01 | 0.7283 | -0.4574 | 6.60E-01 | 9.96E-01 | 0.9734 | -0.0389 | 9.69E-01 | 9.93E-01 | 1.0219 | 0.0313 |
| PC(16:0/20:4) | Spleen | Negative | * | - | - | * | x | x | x | - | 1.68E-04 | 1.74E-03 | 1.2985 | 0.3769 | 8.63E-01 | 9.99E-01 | 0.9901 | -0.0144 | 6.84E-01 | 9.07E-01 | 1.0510 | 0.0717 |
| PS(16:0/18:1) | Spleen | Negative | * | * | - | * | x | x | x | - | 2.01E-06 | 4.94E-05 | 0.6788 | -0.5591 | 3.32E-02 | 9.73E-01 | 1.1735 | 0.2308 | 4.10E-01 | 8.36E-01 | 1.0620 | 0.0869 |
| PC(16:0/18:2) | Spleen | Negative | * | - | - | * | x | x | x | - | 3.29E-03 | 1.85E-02 | 1.2725 | 0.3477 | 8.77E-01 | 9.99E-01 | 0.9857 | -0.0207 | 4.37E-01 | 8.42E-01 | 1.1434 | 0.1933 |
| PS(18:0/20:4) | Spleen | Negative | - | * | * | * | x | x | - | - | 5.27E-03 | 2.63E-02 | 1.1937 | 0.2554 | 1.40E-01 | 9.73E-01 | 1.0905 | 0.1250 | 1.81E-01 | 6.66E-01 | 0.9092 | -0.1373 |
| PS(18:0/18:1) | Spleen | Negative | - | - | * | * | - | - | - | - | 5.51E-01 | 6.55E-01 | 1.0434 | 0.0613 | 3.90E-02 | 9.73E-01 | 1.1632 | 0.2182 | 9.12E-01 | 9.79E-01 | 1.0180 | 0.0258 |
| PE(16:0/18:2) | Spleen | Negative | * | - | - | - | x | x | - | - | 2.01E-05 | 3.18E-04 | 0.6136 | -0.7047 | 1.25E-01 | 9.73E-01 | 1.1080 | 0.1480 | 5.72E-01 | 8.71E-01 | 0.9688 | -0.0458 |
| Cervonic acid | Spleen | Negative | * | - | - | - | - | - | - | - | 8.82E-01 | 9.16E-01 | 0.9401 | -0.0891 | 5.20E-01 | 9.99E-01 | 0.8615 | -0.2151 | 3.39E-01 | 8.01E-01 | 0.8100 | -0.3041 |
| PE(18:0/20:4) | Spleen | Negative | * | * | * | * | x | x | - | - | 3.71E-03 | 2.00E-02 | 0.6432 | -0.6366 | 9.50E-01 | 9.99E-01 | 1.0299 | 0.0425 | 2.60E-02 | 4.87E-01 | 1.2867 | 0.3637 |
| ω-3 Arachidonic acid | Spleen | Negative | * | * | - | - | - | - | x | - | 2.87E-01 | 4.28E-01 | 1.3311 | 0.4126 | 9.38E-01 | 9.99E-01 | 0.9555 | -0.0657 | 1.62E-01 | 6.38E-01 | 0.6655 | -0.5876 |
| PS(18:0/18:2) | Spleen | Negative | * | * | - | - | x | x | - | - | 2.07E-05 | 3.26E-04 | 0.7461 | -0.4225 | 2.36E-02 | 9.73E-01 | 1.1644 | 0.2197 | 3.69E-01 | 8.18E-01 | 1.0690 | 0.0963 |
| PE(P-18:0/22:6) | Spleen | Negative | - | - | * | - | - | - | x | - | 1.84E-01 | 3.32E-01 | 0.8307 | -0.2676 | 4.39E-01 | 9.99E-01 | 0.9081 | -0.1391 | 1.88E-01 | 6.74E-01 | 0.8065 | -0.3103 |
| 12(13)-EpOME | Spleen | Negative | * | * | * | * | - | - | - | - | 3.59E-01 | 4.94E-01 | 0.8709 | -0.1995 | 3.04E-01 | 9.73E-01 | 1.0545 | 0.0766 | 9.42E-01 | 9.86E-01 | 1.7053 | 0.7701 |
| PS(16:0/20:4) | Spleen | Negative | * | - | - | - | x | x | x | - | 8.89E-05 | 1.06E-03 | 0.7522 | -0.4107 | 9.76E-02 | 9.73E-01 | 1.1133 | 0.1549 | 7.67E-01 | 9.32E-01 | 0.9834 | -0.0242 |
| Glu | Spleen | Negative | - | * | * | * | x | x | x | - | 8.42E-05 | 1.02E-03 | 0.7413 | -0.4318 | 1.84E-02 | 9.73E-01 | 0.8527 | -0.2300 | 9.81E-01 | 9.92E-01 | 1.0040 | 0.0058 |
| PS(18:0/22:6) | Spleen | Negative | - | * | * | * | - | - | - | - | 4.12E-01 | 5.46E-01 | 1.0810 | 0.1123 | 5.19E-01 | 9.99E-01 | 1.0510 | 0.0717 | 9.43E-01 | 9.86E-01 | 0.9934 | -0.0095 |
| PE(16:0/20:4) | Spleen | Negative | * | - | - | - | x | x | xxx | - | 2.70E-05 | 4.01E-04 | 0.7299 | -0.4542 | 3.49E-01 | 9.84E-01 | 1.0517 | 0.0727 | 5.14E-01 | 8.59E-01 | 0.9590 | -0.0604 |
| Asp | Spleen | Negative | - | * | * | * | - | - | x | - | 2.72E-02 | 8.88E-02 | 0.8336 | -0.2625 | 4.47E-02 | 9.73E-01 | 0.8568 | -0.2230 | 9.50E-01 | 9.86E-01 | 1.0029 | 0.0042 |
| PE(P-18:0/22:6) | Spleen | Negative | - | * | - | * | x | x | - | - | 7.08E-05 | 9.06E-04 | 0.4727 | -1.0810 | 6.29E-01 | 9.99E-01 | 0.9857 | -0.0208 | 9.71E-01 | 9.90E-01 | 1.0814 | 0.1129 |
| Mannose 6-Phosphate | Spleen | Negative | - | * | - | * | - | - | - | - | 1.26E-02 | 5.11E-02 | 0.7731 | -0.3714 | 8.07E-01 | 9.99E-01 | 0.9982 | -0.0027 | 8.93E-01 | 9.74E-01 | 1.0063 | 0.0090 |
| GLR | Spleen | Positive | * | * | * | * | x | x | x | - | 2.70E-03 | 1.90E-02 | 1.3355 | 0.4174 | 8.12E-01 | 9.66E-01 | 0.9794 | -0.0300 | 7.60E-01 | 9.25E-01 | 0.9844 | -0.0227 |
| PC(16:0/18:1) | Spleen | Positive | - | - | - | - | - | - | - | - | 5.09E-01 | 6.49E-01 | 0.9726 | -0.0400 | 8.08E-01 | 9.66E-01 | 0.9807 | -0.0281 | 2.01E-02 | 2.07E-01 | 0.8459 | -0.2415 |
| PC(18:0/22:6) | Spleen | Positive | * | - | * | * | - | - | - | - | 8.94E-01 | 9.37E-01 | 1.0089 | 0.0128 | 6.87E-01 | 9.50E-01 | 0.9792 | -0.0304 | 4.16E-01 | 7.97E-01 | 1.0398 | 0.0564 |
| Hypoxanthine | Spleen | Positive | * | * | * | * | x | x | x | - | 1.15E-04 | 1.88E-03 | 0.5822 | -0.7805 | 7.97E-01 | 9.65E-01 | 1.0053 | 0.0076 | 6.15E-01 | 8.86E-01 | 1.0652 | 0.0911 |
| PC(16:1/16:1) | Spleen | Positive | * | - | - | * | x | x | x | - | 1.75E-09 | 6.75E-07 | 0.6393 | -0.6455 | 6.79E-01 | 9.42E-01 | 1.0159 | 0.0227 | 7.64E-01 | 9.25E-01 | 0.9888 | -0.0163 |
| LysoPC(17:0) | Spleen | Positive | * | * | * | * | - | - | - | - | 4.28E-02 | 1.29E-01 | 0.7504 | -0.4142 | 8.95E-01 | 9.81E-01 | 1.0275 | 0.0392 | 7.20E-01 | 9.14E-01 | 1.0938 | 0.1294 |
| N-Ac-Phe | Spleen | Positive | * | * | - | - | x | x | - | - | 5.69E-03 | 3.18E-02 | 1.3291 | 0.4104 | 1.78E-01 | 6.79E-01 | 0.8673 | -0.2055 | 6.34E-01 | 8.89E-01 | 0.9634 | -0.0538 |
| LysoPE(16:0) | Spleen | Positive | * | - | * | * | x | x | - | - | 6.75E-04 | 7.46E-03 | 0.6866 | -0.5424 | 6.10E-01 | 9.25E-01 | 1.0326 | 0.0463 | 5.71E-01 | 8.65E-01 | 0.9637 | -0.0533 |
| PC(14:0/16:0) | Spleen | Positive | * | - | - | * | x | x | x | - | 7.57E-05 | 1.34E-03 | 0.6270 | -0.6736 | 7.87E-01 | 9.65E-01 | 1.0281 | 0.0399 | 3.51E-01 | 7.50E-01 | 0.9179 | -0.1237 |
| 11-HEDE | Spleen | Positive | * | * | * | - | - | - | - | - | 2.26E-01 | 3.85E-01 | 1.3256 | 0.4066 | 3.88E-01 | 8.30E-01 | 0.8099 | -0.3042 | 1.30E-01 | 4.90E-01 | 0.6809 | -0.5545 |
| PC(18:0/18:1) | Spleen | Positive | * | * | * | * | - | - | - | - | 1.57E-02 | 6.43E-02 | 1.1520 | 0.2042 | 4.43E-01 | 8.60E-01 | 0.9596 | -0.0594 | 8.97E-02 | 3.96E-01 | 0.9074 | -0.1402 |
| L-Valine | Spleen | Positive | - | * | * | * | x | x | x | - | 9.29E-07 | 4.93E-05 | 0.5822 | -0.7805 | 2.75E-01 | 7.57E-01 | 0.9354 | -0.0964 | 3.37E-01 | 7.39E-01 | 1.0606 | 0.0849 |
| Arachidonic acid | Spleen | Positive | * | * | - | - | - | - | x | - | 2.07E-01 | 3.66E-01 | 1.4092 | 0.4949 | 8.91E-01 | 9.81E-01 | 0.9530 | -0.0695 | 2.06E-01 | 6.28E-01 | 0.7145 | -0.4850 |
| Taurolite | Spleen | Positive | - | - | * | * | x | x | - | - | 3.84E-03 | 2.42E-02 | 0.6329 | -0.6599 | 4.78E-01 | 8.78E-01 | 0.9007 | -0.1509 | 3.30E-01 | 7.36E-01 | 0.8693 | -0.2021 |
| DCHA-PAF | Spleen | Positive | - | * | * | * | x | x | x | - | 1.48E-05 | 3.95E-04 | 0.7942 | -0.3324 | 6.19E-01 | 9.27E-01 | 0.9846 | -0.0225 | 8.87E-01 | 9.63E-01 | 1.0081 | 0.0116 |
| LysoPC(22:0) | Spleen | Positive | * | * | * | * | - | - | - | - | 2.22E-01 | 3.82E-01 | 0.9267 | -0.1098 | 5.02E-02 | 5.44E-01 | 1.1339 | 0.1813 | 5.43E-03 | 9.66E-02 | 1.1768 | 0.2349 |
| PC(18:1/14:0) | Spleen | Positive | - | - | - | * | x | x | x | - | 2.46E-03 | 1.81E-02 | 0.8513 | -0.2323 | 2.03E-01 | 6.93E-01 | 0.9391 | -0.0906 | 5.46E-03 | 9.66E-02 | 0.8524 | -0.2304 |
| SM(d18:1/18:1) | Spleen | Positive | - | * | * | * | x | x | x | - | 4.78E-05 | 9.38E-04 | 1.3619 | 0.4457 | 6.56E-01 | 9.40E-01 | 1.0302 | 0.0430 | 4.10E-02 | 2.69E-01 | 0.8382 | -0.2546 |
| GSSG | Spleen | Positive | * | * | * | * | - | - | x | - | 2.38E-02 | 8.48E-02 | 0.6096 | -0.7140 | 7.18E-01 | 9.51E-01 | 1.0134 | 0.0193 | 2.71E-01 | 6.79E-01 | 1.3137 | 0.3936 |
| SM(d18:1/18:0) | Spleen | Positive | - | * | * | * | - | - | - | - | 2.70E-01 | 4.37E-01 | 1.1540 | 0.2066 | 3.54E-01 | 8.08E-01 | 1.0816 | 0.1132 | 5.54E-01 | 8.56E-01 | 0.9284 | -0.1071 |
| PC(P-18:0/0:0) | Spleen | Positive | - | * | - | * | x | x | x | - | 2.54E-06 | 1.04E-04 | 0.3706 | -1.4320 | 5.91E-01 | 9.19E-01 | 0.9748 | -0.0368 | 5.19E-01 | 8.46E-01 | 1.2651 | 0.3393 |
| Arg | Spleen | Positive | * | * | * | * | x | x | x | - | 1.01E-06 | 5.10E-05 | 1.8313 | 0.8729 | 1.72E-01 | 6.79E-01 | 0.8638 | -0.2113 | 2.38E-01 | 6.64E-01 | 0.8905 | -0.1673 |
| 3-Indolyllactic acid | Spleen | Positive | - | * | * | * | x | x | x | - | 1.44E-05 | 3.89E-04 | 0.6111 | -0.7104 | 7.97E-01 | 9.65E-01 | 0.9797 | -0.0295 | 9.57E-01 | 9.85E-01 | 1.0200 | 0.0285 |
| LysoPE(18:0) | Kidney | Negative | * | * | * | * | - | - | - | - | 1.03E-01 | 6.21E-01 | 0.8810 | -0.1828 | 2.77E-01 | 4.39E-01 | 1.0800 | 0.1110 | 1.01E-01 | 3.94E-01 | 1.1458 | 0.1964 |
| Glu | Kidney | Negative | * | * | * | * | - | - | x | - | 4.26E-01 | 7.74E-01 | 0.9706 | -0.0431 | 2.74E-01 | 4.37E-01 | 1.0557 | 0.0783 | 3.20E-02 | 2.98E-01 | 1.1654 | 0.2209 |
| 2-Hydroxypalmitic acid | Kidney | Negative | * | * | - | * | - | - | - | - | 2.75E-02 | 4.02E-01 | 0.7884 | -0.3430 | 2.23E-01 | 3.78E-01 | 1.0561 | 0.0788 | 2.23E-03 | 1.39E-01 | 1.1811 | 0.2402 |
| Citric Acid | Kidney | Negative | * | * | * | * | - | - | - | - | 3.49E-01 | 7.56E-01 | 0.5742 | -0.8004 | 4.12E-01 | 5.57E-01 | 1.1230 | 0.1673 | 1.39E-01 | 4.53E-01 | 1.2137 | 0.2794 |
| PC(16:0/20:4) | Kidney | Negative | - | * | * | * | - | - | - | - | 6.61E-02 | 5.57E-01 | 1.1759 | 0.2337 | 8.16E-01 | 8.69E-01 | 1.0273 | 0.0388 | 1.39E-01 | 4.53E-01 | 0.8678 | -0.2046 |
| PC(16:0/18:2) | Kidney | Negative | - | * | * | * | - | - | - | - | 1.50E-01 | 6.79E-01 | 1.1659 | 0.2214 | 9.81E-01 | 9.87E-01 | 1.0184 | 0.0264 | 2.09E-01 | 5.24E-01 | 0.8735 | -0.1951 |
| Cervonic acid | Kidney | Negative | * | * | - | - | - | - | - | - | 4.43E-01 | 7.80E-01 | 0.9341 | -0.0984 | 2.80E-02 | 1.36E-01 | 1.2387 | 0.3088 | 1.64E-01 | 4.77E-01 | 1.1354 | 0.1832 |
| PC(16:0/22:6) | Kidney | Negative | * | * | - | - | - | - | - | - | 3.90E-01 | 7.61E-01 | 1.0549 | 0.0771 | 1.34E-01 | 2.82E-01 | 0.9132 | -0.1310 | 1.62E-01 | 4.77E-01 | 0.8984 | -0.1545 |
| PI(18:0/20:4) | Kidney | Negative | - | - | - | * | - | - | - | - | 6.87E-01 | 8.76E-01 | 1.0845 | 0.1170 | 1.93E-01 | 3.46E-01 | 1.2592 | 0.3325 | 4.71E-01 | 7.36E-01 | 1.1715 | 0.2284 |
| PC(18:1/16:0) | Kidney | Negative | * | * | * | * | - | - | - | - | 1.75E-01 | 6.94E-01 | 1.0717 | 0.0998 | 2.69E-02 | 1.35E-01 | 0.9101 | -0.1359 | 5.16E-02 | 3.29E-01 | 0.9156 | -0.1272 |
| LysoPE(16:0) | Kidney | Negative | * | * | * | * | - | - | x | - | 1.53E-01 | 6.80E-01 | 0.9060 | -0.1424 | 9.67E-02 | 2.38E-01 | 1.1162 | 0.1586 | 7.94E-02 | 3.64E-01 | 1.1618 | 0.2164 |
| 14(15)-EpEDE | Kidney | Negative | * | * | - | * | - | - | - | - | 9.66E-01 | 9.92E-01 | 0.9922 | -0.0113 | 8.50E-03 | 8.91E-02 | 1.2792 | 0.3553 | 2.01E-01 | 5.18E-01 | 1.1639 | 0.2190 |

| Name | SD9 Radiation p-value | SD9 Radiation FDR | SD9 Radiation Fold Change | SD9 Radiation Log2(FC) | SD9 Amifostine 50mg p-value | SD9 Amifostine 50mg FDR | SD9 Amifostine 50mg Fold Change | SD9 Amifostine 50mg Log2(FC) | SD9 Amifostine 200mg p-value | SD9 Amifostine 200mg FDR | SD9 Amifostine 200mg Fold Change | SD9 Amifostine 200mg Log2(FC) |
|---|---|---|---|---|---|---|---|---|---|---|---|---|
| PC(16:0/18:2) | 9.80E-01 | 1.00E+00 | 1.0147 | 0.0210 | 2.85E-02 | 1.00E+00 | 1.2006 | 0.2637 | 9.37E-01 | 9.66E-01 | 0.9834 | -0.0241 |
| PC(16:0/20:5) | 5.03E-02 | 9.20E-01 | 0.8411 | -0.2497 | 4.24E-02 | 1.00E+00 | 1.1641 | 0.2193 | 2.88E-01 | 6.80E-01 | 1.0636 | 0.0889 |
| Arachidonoyl Thio-PC | 4.12E-03 | 3.49E-01 | 1.2167 | 0.2830 | 3.82E-01 | 1.00E+00 | 1.0544 | 0.0764 | 3.54E-02 | 6.33E-01 | 0.8668 | -0.2062 |
| PC(16:0/18:1) | 6.77E-01 | 9.76E-01 | 1.0251 | 0.0358 | 3.91E-02 | 1.00E+00 | 1.1558 | 0.2089 | 3.11E-01 | 6.89E-01 | 1.0584 | 0.0818 |
| 5'-Adenylic acid | 3.76E-01 | 9.40E-01 | 1.1272 | 0.1727 | 5.96E-02 | 1.00E+00 | 0.7223 | -0.4694 | 4.11E-01 | 7.02E-01 | 0.8974 | -0.1562 |
| PC(16:1/16:1) | 1.58E-01 | 9.40E-01 | 0.8958 | -0.1588 | 7.97E-02 | 1.00E+00 | 1.2241 | 0.2918 | 5.91E-01 | 7.82E-01 | 0.9407 | -0.0882 |
| PE(18:0/22:6) | 6.52E-01 | 9.70E-01 | 1.0307 | 0.0436 | 5.59E-01 | 1.00E+00 | 0.9589 | -0.0606 | 4.92E-01 | 7.27E-01 | 0.9586 | -0.0610 |
| PC(16:0/18:0) | 9.17E-01 | 1.00E+00 | 1.0014 | 0.0020 | 4.96E-01 | 1.00E+00 | 1.0660 | 0.0922 | 8.58E-01 | 9.30E-01 | 0.9916 | -0.0121 |
| Cervonic acid | 5.74E-01 | 9.64E-01 | 0.9346 | -0.0975 | 9.09E-01 | 1.00E+00 | 0.9765 | -0.0343 | 1.00E-01 | 6.38E-01 | 1.7024 | 0.7676 |
| Methyl oleoylethanolamide | 2.15E-04 | 7.01E-02 | 0.5944 | -0.7506 | 3.97E-01 | 1.00E+00 | 1.1622 | 0.2168 | 7.65E-02 | 6.33E-01 | 1.3178 | 0.3981 |
| DELTA.17-U-46619 | 4.61E-01 | 9.49E-01 | 0.9388 | -0.0912 | 9.00E-01 | 1.00E+00 | 0.9970 | -0.0043 | 3.02E-01 | 6.88E-01 | 1.1133 | 0.1548 |
| Hypoxanthine | 1.04E-01 | 9.40E-01 | 0.9110 | -0.1345 | 2.33E-01 | 1.00E+00 | 1.0888 | 0.1227 | 3.10E-01 | 6.89E-01 | 1.0675 | 0.0942 |
| LysoPE(18:0) | 4.69E-01 | 9.50E-01 | 0.9012 | -0.1501 | 5.02E-01 | 1.00E+00 | 1.1686 | 0.2247 | 1.25E-01 | 6.47E-01 | 1.2733 | 0.3485 |
| Cer(d18:1/20:0) | 8.53E-02 | 9.40E-01 | 1.9741 | 0.9812 | 6.90E-01 | 1.00E+00 | 0.8913 | -0.1661 | 2.76E-01 | 6.80E-01 | 0.6584 | -0.6029 |
| PG(16:0/18:1) | 1.43E-01 | 9.40E-01 | 1.6495 | 0.7221 | 8.06E-01 | 1.00E+00 | 1.1048 | 0.1438 | 4.95E-01 | 7.27E-01 | 0.7817 | -0.3553 |
| PG(18:1/18:1) | 1.01E-01 | 9.40E-01 | 1.5751 | 0.6555 | 9.49E-01 | 1.00E+00 | 1.0536 | 0.0754 | 5.63E-01 | 7.62E-01 | 0.8491 | -0.2360 |
| PC(22:6/18:0) | 2.77E-01 | 9.40E-01 | 0.9376 | -0.0930 | 5.10E-01 | 1.00E+00 | 1.0454 | 0.0640 | 1.89E-01 | 6.56E-01 | 1.0765 | 0.1063 |
| Methyl DHA | 7.53E-01 | 9.86E-01 | 0.9776 | -0.0327 | 7.34E-01 | 1.00E+00 | 1.0191 | 0.0273 | 7.23E-01 | 8.57E-01 | 0.9345 | -0.0977 |
| LysoPC(16:0) | 4.77E-01 | 9.50E-01 | 1.0040 | 0.0057 | 7.86E-01 | 1.00E+00 | 0.9763 | -0.0346 | 1.69E-01 | 6.56E-01 | 1.5590 | 0.6407 |
| Monoolein | 1.26E-01 | 9.40E-01 | 0.4967 | -1.0096 | 1.17E-01 | 1.00E+00 | 1.6979 | 0.7638 | 1.16E-01 | 6.41E-01 | 2.5384 | 1.3439 |
| Acetylcarnitine | 3.51E-02 | 8.35E-01 | 0.7238 | -0.4663 | 7.16E-01 | 1.00E+00 | 1.1181 | 0.1610 | 1.32E-01 | 6.47E-01 | 1.3078 | 0.3871 |
| PG(18:2) | 5.98E-02 | 9.40E-01 | 1.5486 | 0.6310 | 8.40E-01 | 1.00E+00 | 1.0210 | 0.0300 | 7.69E-02 | 6.33E-01 | 0.7588 | -0.3982 |
| PC(16:0/22:6) | 1.17E-01 | 9.40E-01 | 0.9366 | -0.0945 | 4.47E-01 | 1.00E+00 | 1.0404 | 0.0572 | 3.84E-01 | 6.95E-01 | 1.0330 | 0.0468 |
| Adrenic Acid (22:4, n-6) | 4.34E-01 | 9.46E-01 | 0.9205 | -0.1196 | 8.39E-01 | 1.00E+00 | 1.0111 | 0.0160 | 2.82E-01 | 6.80E-01 | 1.1831 | 0.2426 |
| Glu | 9.26E-01 | 1.00E+00 | 0.9996 | -0.0006 | 8.67E-01 | 1.00E+00 | 1.0098 | 0.0141 | 3.32E-01 | 6.95E-01 | 0.9517 | -0.0715 |
| γ-Homolinolenic acid | 2.06E-01 | 9.40E-01 | 0.8620 | -0.2142 | 8.15E-01 | 1.00E+00 | 1.0382 | 0.0541 | 8.92E-02 | 6.38E-01 | 1.2488 | 0.3206 |
| DL-Norvaline | 5.63E-03 | 3.98E-01 | 0.7143 | -0.4853 | 7.52E-02 | 1.00E+00 | 1.2743 | 0.3497 | 2.06E-01 | 6.56E-01 | 1.1829 | 0.2424 |
| N-Arachidonoyl-L-serine | 7.80E-01 | 9.87E-01 | 0.9708 | -0.0428 | 4.19E-01 | 1.00E+00 | 0.8867 | -0.1735 | 7.22E-01 | 8.57E-01 | 0.9433 | -0.0842 |
| 2-AG | 3.28E-01 | 9.40E-01 | 0.8772 | -0.1891 | 9.60E-01 | 1.00E+00 | 0.9499 | -0.0742 | 9.58E-02 | 6.38E-01 | 1.4437 | 0.5298 |
| Eicosadienoic acid | 2.24E-01 | 9.40E-01 | 0.8135 | -0.2978 | 3.56E-01 | 1.00E+00 | 0.8508 | -0.2331 | 1.36E-01 | 6.51E-01 | 1.5374 | 0.6205 |
| LysoPE(16:0) | 1.08E-01 | 9.40E-01 | 0.6895 | -0.5364 | 4.46E-01 | 1.00E+00 | 1.5574 | 0.6391 | 7.00E-01 | 8.45E-01 | 0.7681 | -0.3807 |
| Gln | 3.22E-01 | 9.40E-01 | 0.8880 | -0.1713 | 4.57E-01 | 1.00E+00 | 0.8577 | -0.2215 | 2.67E-01 | 6.69E-01 | 1.1960 | 0.2583 |
| L-Carnitine | 3.56E-01 | 9.40E-01 | 1.1041 | 0.1429 | 1.77E-01 | 1.00E+00 | 0.8597 | -0.2181 | 9.63E-01 | 9.81E-01 | 1.0176 | 0.0252 |
| LysoPC(18:0) | 9.25E-01 | 1.00E+00 | 1.0186 | 0.0265 | 7.79E-01 | 1.00E+00 | 1.0213 | 0.0304 | 2.53E-01 | 6.59E-01 | 1.1415 | 0.1909 |
| 7α,12α-Dihydroxycholest-4-en-3-one | 3.80E-01 | 9.40E-01 | 0.8676 | -0.2050 | 6.97E-01 | 1.00E+00 | 0.9870 | -0.0189 | 5.61E-02 | 6.33E-01 | 2.2039 | 1.1401 |
| Arachidonic acid | 7.63E-01 | 9.86E-01 | 0.9833 | -0.0243 | 3.86E-01 | 1.00E+00 | 0.8759 | -0.1911 | 3.34E-01 | 6.95E-01 | 0.8661 | -0.2074 |
| Creatine | 5.16E-01 | 9.55E-01 | 0.9398 | -0.0896 | 5.77E-01 | 1.00E+00 | 0.9126 | -0.1319 | 6.24E-01 | 7.99E-01 | 1.0585 | 0.0821 |
| Methyl ricinoleate | 5.16E-01 | 9.55E-01 | 1.1194 | 0.1628 | 1.61E-01 | 1.00E+00 | 0.7895 | -0.3410 | 3.23E-01 | 6.95E-01 | 1.3384 | 0.4205 |
| Methyl DPA | 6.14E-01 | 9.70E-01 | 0.9106 | -0.1351 | 9.00E-01 | 1.00E+00 | 0.9867 | -0.0194 | 6.13E-02 | 6.33E-01 | 1.5715 | 0.6522 |
| Erucamide | 7.43E-01 | 9.85E-01 | 0.9859 | -0.0206 | 3.88E-01 | 1.00E+00 | 0.8824 | -0.1805 | 6.93E-01 | 8.39E-01 | 0.9494 | -0.0749 |
| 9(10)-EpOME | 4.89E-04 | 9.79E-02 | 0.6171 | -0.6965 | 1.14E-01 | 1.00E+00 | 1.1485 | 0.1997 | 2.64E-02 | 6.33E-01 | 1.3164 | 0.3966 |
| N-Arachidonoyl-L-alanine | 3.18E-02 | 7.96E-01 | 0.6320 | -0.6620 | 3.34E-01 | 1.00E+00 | 1.1267 | 0.1721 | 2.20E-02 | 6.33E-01 | 1.7613 | 0.8167 |
| PC(18:0/18:1) | 2.07E-01 | 9.40E-01 | 1.2997 | 0.3782 | 8.56E-01 | 1.00E+00 | 1.0213 | 0.0304 | 3.52E-02 | 6.33E-01 | 0.6478 | -0.6264 |
| 2-Linoleoylglycerol | 1.26E-01 | 9.40E-01 | 0.7935 | -0.3337 | 2.26E-01 | 1.00E+00 | 1.1487 | 0.2000 | 9.50E-02 | 6.38E-01 | 1.4238 | 0.5098 |
| 15,19-Hhydroxy PGE1 | 6.37E-01 | 9.70E-01 | 1.1061 | 0.1455 | 5.92E-01 | 1.00E+00 | 0.9013 | -0.1500 | 1.59E-01 | 6.51E-01 | 1.3873 | 0.4723 |
| PC(16:0/20:4) | 1.70E-03 | 1.08E-02 | 1.2121 | 0.2775 | 5.36E-01 | 9.99E-01 | 1.0469 | 0.0662 | 4.68E-04 | 1.39E-02 | 0.8047 | -0.3135 |
| PS(16:0/18:1) | 1.86E-04 | 1.83E-03 | 0.7433 | -0.4280 | 3.60E-03 | 2.66E-01 | 0.8104 | -0.3034 | 8.70E-01 | 9.42E-01 | 1.0322 | 0.0457 |
| PC(16:0/18:2) | 2.52E-03 | 1.47E-02 | 1.2789 | 0.3549 | 4.09E-01 | 9.99E-01 | 1.0952 | 0.1312 | 4.38E-03 | 4.61E-02 | 0.7792 | -0.3600 |
| PS(18:0/20:4) | 5.36E-02 | 1.61E-01 | 1.1608 | 0.2151 | 1.15E-01 | 9.99E-01 | 0.8770 | -0.1893 | 1.16E-01 | 3.21E-01 | 0.8572 | -0.2223 |
| PS(18:0/18:1) | 8.18E-02 | 2.17E-01 | 1.1578 | 0.2114 | 3.66E-01 | 9.99E-01 | 0.9212 | -0.1184 | 5.00E-01 | 7.39E-01 | 0.9391 | -0.0907 |
| PE(16:0/18:2) | 4.74E-02 | 1.47E-01 | 0.8272 | -0.2737 | 5.89E-02 | 8.04E-01 | 0.8417 | -0.2486 | 3.72E-02 | 1.67E-01 | 0.8320 | -0.2653 |
| Cervonic acid | 1.41E-02 | 5.69E-02 | 1.7704 | 0.8241 | 3.17E-01 | 9.99E-01 | 1.2139 | 0.2796 | 5.12E-02 | 1.97E-01 | 1.4645 | 0.5504 |
| PE(18:0/20:4) | 8.02E-01 | 8.99E-01 | 0.9950 | -0.0072 | 7.49E-01 | 9.99E-01 | 1.0866 | 0.1198 | 1.03E-01 | 3.01E-01 | 1.2609 | 0.3344 |
| ω-3 Arachidonic acid | 1.18E-02 | 4.93E-02 | 1.8112 | 0.8569 | 6.29E-01 | 9.99E-01 | 1.0771 | 0.1072 | 3.67E-01 | 6.51E-01 | 1.2150 | 0.2810 |
| PS(18:0/18:2) | 1.13E-01 | 2.71E-01 | 0.8777 | -0.1882 | 8.46E-02 | 9.39E-01 | 0.8663 | -0.2071 | 8.54E-01 | 9.32E-01 | 0.9926 | -0.0107 |
| PE(P-18:0/22:6) | 5.76E-03 | 2.78E-02 | 1.6116 | 0.6885 | 1.33E-01 | 9.99E-01 | 0.7652 | -0.3861 | 4.36E-01 | 7.02E-01 | 1.1103 | 0.1510 |
| 12(13)-EpOME | 9.93E-01 | 9.96E-01 | 1.2155 | 0.2815 | 1.41E-01 | 9.99E-01 | 0.5318 | -0.9111 | 7.61E-01 | 8.83E-01 | 0.7191 | -0.4758 |
| PS(16:0/20:4) | 3.92E-05 | 5.03E-04 | 0.6725 | -0.5723 | 3.07E-03 | 2.56E-01 | 0.7740 | -0.3696 | 3.68E-01 | 6.51E-01 | 0.9275 | -0.1086 |
| Glu | 1.27E-05 | 2.15E-04 | 0.6347 | -0.6559 | 3.30E-01 | 9.99E-01 | 1.0980 | 0.1349 | 4.48E-02 | 1.83E-01 | 1.1886 | 0.2493 |
| PS(18:0/22:6) | 4.67E-02 | 1.46E-01 | 1.1478 | 0.1988 | 7.75E-01 | 9.99E-01 | 0.9821 | -0.0260 | 2.72E-01 | 5.54E-01 | 0.9333 | -0.0996 |
| PE(16:0/20:4) | 2.22E-04 | 2.12E-03 | 0.7911 | -0.3381 | 3.60E-05 | 2.48E-02 | 0.8041 | -0.3145 | 3.86E-04 | 1.34E-02 | 0.8296 | -0.2695 |
| Asp | 1.38E-05 | 2.25E-04 | 0.6026 | -0.7308 | 8.46E-01 | 9.99E-01 | 1.0297 | 0.0422 | 7.82E-01 | 8.93E-01 | 1.0285 | 0.0406 |
| PE(P-18:0/22:6) | 5.99E-02 | 1.74E-01 | 0.7237 | -0.4666 | 4.32E-01 | 9.99E-01 | 0.8741 | -0.1941 | 2.02E-01 | 4.57E-01 | 1.2653 | 0.3394 |
| Mannose 6-Phosphate | 1.67E-02 | 6.51E-02 | 0.7825 | -0.3539 | 3.89E-01 | 9.99E-01 | 0.8792 | -0.1857 | 3.12E-02 | 1.53E-01 | 1.2879 | 0.3651 |
| GLR | 1.68E-03 | 1.31E-02 | 1.4569 | 0.5429 | 8.03E-01 | 9.80E-01 | 0.9837 | -0.0237 | 1.06E-02 | 1.00E-01 | 0.7453 | -0.4241 |
| PC(16:0/18:1) | 9.64E-02 | 2.81E-01 | 0.9523 | -0.0706 | 2.02E-01 | 7.21E-01 | 0.9566 | -0.0641 | 6.00E-01 | 8.02E-01 | 0.9853 | -0.0214 |
| PC(18:0/22:6) | 2.04E-02 | 9.73E-02 | 0.8880 | -0.1715 | 6.10E-02 | 6.32E-01 | 1.1078 | 0.1477 | 4.02E-03 | 4.99E-02 | 1.1828 | 0.2422 |
| Hypoxanthine | 6.34E-07 | 1.62E-05 | 0.5018 | -0.9949 | 4.41E-01 | 8.46E-01 | 1.0889 | 0.1228 | 2.93E-04 | 7.51E-03 | 1.4745 | 0.5602 |
| PC(16:1/16:1) | 1.12E-08 | 6.46E-07 | 0.6209 | -0.6877 | 5.69E-01 | 9.24E-01 | 0.9822 | -0.0260 | 5.85E-06 | 2.55E-04 | 1.2271 | 0.2952 |
| LysoPC(17:0) | 8.25E-01 | 9.00E-01 | 0.9947 | -0.0077 | 7.18E-01 | 9.59E-01 | 1.0539 | 0.0757 | 4.77E-01 | 7.30E-01 | 1.0935 | 0.1290 |
| N-Ac-Phe | 4.64E-01 | 6.77E-01 | 1.1312 | 0.1779 | 7.93E-01 | 9.76E-01 | 1.0951 | 0.1310 | 1.01E-01 | 3.50E-01 | 1.3067 | 0.3860 |
| LysoPE(16:0) | 1.52E-01 | 3.70E-01 | 1.1791 | 0.2377 | 5.16E-01 | 8.92E-01 | 0.9330 | -0.1001 | 4.51E-01 | 7.16E-01 | 0.9150 | -0.1282 |
| PC(14:0/16:0) | 1.06E-04 | 1.23E-03 | 0.7469 | -0.4210 | 3.01E-02 | 6.15E-01 | 0.8172 | -0.2912 | 3.34E-05 | 1.21E-03 | 1.2352 | 0.3047 |
| 11-HEDE | 1.01E-02 | 5.62E-02 | 1.8189 | 0.8630 | 9.99E-01 | 1.00E+00 | 0.9581 | -0.0617 | 3.30E-01 | 6.08E-01 | 1.2136 | 0.2793 |
| PC(18:0/18:1) | 5.02E-02 | 1.81E-01 | 0.9147 | -0.1287 | 3.52E-02 | 6.15E-01 | 1.1041 | 0.1428 | 2.52E-01 | 5.25E-01 | 1.0387 | 0.0548 |
| L-Valine | 1.64E-09 | 1.21E-07 | 0.4706 | -1.0875 | 1.04E-01 | 6.56E-01 | 1.1617 | 0.2162 | 8.88E-09 | 6.57E-07 | 1.7597 | 0.8153 |
| Arachidonic acid | 3.81E-03 | 2.59E-02 | 1.7899 | 0.8398 | 6.91E-01 | 9.58E-01 | 1.0544 | 0.0765 | 4.65E-01 | 7.24E-01 | 1.1283 | 0.1741 |
| Taurolite | 6.59E-02 | 2.18E-01 | 1.3122 | 0.3919 | 2.66E-01 | 7.54E-01 | 0.8482 | -0.2375 | 7.12E-01 | 8.69E-01 | 0.9588 | -0.0606 |
| DCHA-PAF | 7.03E-09 | 4.51E-07 | 0.7149 | -0.4842 | 8.66E-01 | 9.85E-01 | 1.0087 | 0.0126 | 2.51E-10 | 4.32E-08 | 1.7174 | 0.7802 |
| LysoPC(22:0) | 4.02E-01 | 6.28E-01 | 1.0466 | 0.0657 | 4.74E-01 | 8.67E-01 | 0.9612 | -0.0571 | 2.20E-01 | 4.86E-01 | 0.9228 | -0.1160 |
| PC(18:1/14:0) | 5.38E-06 | 9.40E-05 | 0.7707 | -0.3758 | 3.90E-03 | 4.69E-01 | 0.8651 | -0.2090 | 9.36E-01 | 9.68E-01 | 1.0040 | 0.0058 |
| SM(d18:1/18:1) | 2.18E-05 | 3.18E-04 | 1.3200 | 0.4005 | 3.31E-01 | 8.00E-01 | 0.9470 | -0.0786 | 5.63E-01 | 7.77E-01 | 0.9836 | -0.0238 |
| GSSG | 1.59E-03 | 1.26E-02 | 0.4762 | -1.0705 | 9.55E-01 | 9.99E-01 | 1.0148 | 0.0213 | 4.45E-01 | 7.09E-01 | 1.2280 | 0.2963 |
| SM(d18:1/18:0) | 3.72E-02 | 1.48E-01 | 1.1948 | 0.2568 | 4.08E-01 | 8.31E-01 | 0.9435 | -0.0839 | 1.16E-03 | 1.96E-02 | 0.7137 | -0.4866 |
| PC(P-18:0/0:0) | 1.33E-04 | 1.46E-03 | 0.3465 | -1.5293 | 6.11E-01 | 9.36E-01 | 0.8609 | -0.2161 | 4.13E-03 | 5.03E-02 | 2.1083 | 1.0761 |
| Arg | 2.29E-05 | 3.26E-04 | 1.7485 | 0.8061 | 6.58E-01 | 9.46E-01 | 0.9633 | -0.0539 | 6.75E-01 | 8.46E-01 | 0.9646 | -0.0520 |
| 3-Indolyllactic acid | 1.90E-06 | 3.88E-05 | 0.6367 | -0.6513 | 5.74E-01 | 9.25E-01 | 1.0919 | 0.1269 | 2.71E-03 | 3.60E-02 | 1.2511 | 0.3232 |
| LysoPE(18:0) | 5.77E-02 | 2.13E-01 | 1.2388 | 0.3090 | 2.06E-01 | 7.13E-01 | 0.8672 | -0.2055 | 1.02E-01 | 4.70E-01 | 0.7791 | -0.3601 |
| Glu | 1.03E-06 | 1.65E-04 | 0.8040 | -0.3148 | 1.14E-03 | 1.33E-01 | 1.1797 | 0.2384 | 2.25E-03 | 7.44E-02 | 1.2506 | 0.3226 |
| 2-Hydroxypalmitic acid | 6.25E-01 | 7.48E-01 | 1.0588 | 0.0825 | 3.08E-01 | 7.92E-01 | 1.8416 | 0.8809 | 6.24E-01 | 8.37E-01 | 0.9436 | -0.0838 |
| Citric Acid | 1.78E-02 | 1.08E-01 | 2.6207 | 1.3899 | 5.88E-02 | 5.91E-01 | 0.4312 | -1.2135 | 2.75E-02 | 2.76E-01 | 0.3789 | -1.4003 |
| PC(16:0/20:4) | 4.59E-01 | 6.25E-01 | 1.1089 | 0.1492 | 4.02E-01 | 8.09E-01 | 0.8917 | -0.1654 | 3.13E-01 | 6.58E-01 | 0.8724 | -0.1970 |
| PC(16:0/18:2) | 2.94E-01 | 4.92E-01 | 0.8687 | -0.2031 | 3.73E-01 | 8.03E-01 | 1.1639 | 0.2190 | 4.05E-01 | 7.10E-01 | 1.2490 | 0.3207 |
| Cervonic acid | 1.32E-02 | 8.73E-02 | 1.3540 | 0.4373 | 2.01E-01 | 7.12E-01 | 1.1369 | 0.1851 | 3.02E-01 | 6.50E-01 | 1.1169 | 0.1596 |
| PC(16:0/22:6) | 4.96E-01 | 6.56E-01 | 0.9559 | -0.0650 | 1.00E+00 | 1.00E+00 | 0.9860 | -0.0203 | 8.42E-01 | 9.38E-01 | 0.9737 | -0.0384 |
| PI(18:0/20:4) | 2.11E-01 | 4.20E-01 | 0.6943 | -0.5263 | 9.43E-01 | 9.86E-01 | 0.8044 | -0.3139 | 3.68E-01 | 6.97E-01 | 1.1295 | 0.1757 |
| PC(18:1/16:0) | 8.81E-01 | 9.25E-01 | 1.0153 | 0.0219 | 7.88E-01 | 9.47E-01 | 0.9992 | -0.0012 | 3.96E-01 | 7.08E-01 | 0.8757 | -0.1915 |
| LysoPE(16:0) | 3.08E-04 | 6.50E-03 | 1.4798 | 0.5654 | 1.41E-01 | 6.65E-01 | 0.8601 | -0.2174 | 2.56E-02 | 2.61E-01 | 0.7454 | -0.4239 |
| 14(15)-EpEDE | 6.64E-02 | 2.28E-01 | 1.2083 | 0.2730 | 7.93E-01 | 9.48E-01 | 1.0291 | 0.0414 | 6.79E-01 | 8.64E-01 | 0.9546 | -0.0671 |

| | | | | | | | | | | | | | | | | | | | | | | | | | | | | | | |
|---|---|---|---|---|---|---|---|---|---|---|---|---|---|---|---|---|---|---|---|---|---|---|---|---|---|---|---|---|---|---|
| PC(18:0/22:6) | Kidney | Negative | * | * | * | * | - | - | - | - | 6.98E-01 | 8.77E-01 | 1.0209 | ↑ 0.0298 | 2.59E-01 | 4.20E-01 | 0.8960 | ↓ -0.1585 | 4.37E-01 | 7.15E-01 | 0.9367 | ↓ -0.0944 | 5.10E-01 | 6.66E-01 | 0.9287 | ↓ -0.1068 | 3.56E-01 | 8.03E-01 | 1.2637 | ↑ 0.3377 |
| PS(16:0/20:4) | Kidney | Negative | - | * | * | * | - | - | - | - | 2.40E-01 | 7.35E-01 | 0.9069 | ↓ -0.1410 | 3.91E-01 | 5.41E-01 | 0.9195 | ↓ -0.1211 | 3.79E-01 | 6.66E-01 | 1.0896 | ↑ 0.1238 | 6.83E-01 | 7.85E-01 | 1.0273 | ↑ 0.0388 | 6.21E-01 | 8.87E-01 | 0.9676 | ↓ -0.0476 |
| PE(P-18:0/22:6) | Kidney | Negative | - | - | * | * | - | - | x | - | 3.91E-01 | 7.61E-01 | 1.0761 | ↑ 0.1058 | 3.59E-01 | 5.13E-01 | 1.0879 | ↑ 0.1215 | 7.41E-01 | 8.88E-01 | 1.0355 | ↑ 0.0504 | 3.66E-03 | 3.45E-02 | 1.6061 | ↑ 0.6836 | 4.33E-02 | 5.60E-01 | 0.7634 | ↓ -0.3894 |
| PE(18:0/22:6) | Kidney | Negative | - | * | - | * | - | - | - | - | 1.77E-01 | 6.94E-01 | 0.8958 | ↓ -0.1587 | 3.06E-02 | 1.42E-01 | 0.8392 | ↓ -0.2530 | 9.58E-01 | 9.80E-01 | 1.0197 | ↑ 0.0282 | 6.78E-01 | 7.82E-01 | 0.9590 | ↓ -0.0605 | 8.34E-01 | 9.57E-01 | 0.9845 | ↓ -0.0225 |
| PC(16:0/18:0) | Kidney | Negative | * | * | - | - | - | - | - | - | 7.21E-01 | 8.83E-01 | 1.0110 | ↑ 0.0158 | 1.28E-02 | 1.01E-01 | 0.9079 | ↓ -0.1393 | 5.14E-02 | 3.29E-01 | 0.9043 | ↓ -0.1451 | 8.22E-01 | 8.86E-01 | 0.9924 | ↓ -0.0110 | 8.52E-01 | 9.61E-01 | 0.9894 | ↓ -0.0154 |
| PG(16:0/18:1) | Kidney | Negative | * | - | * | * | - | - | - | - | 7.63E-01 | 9.06E-01 | 1.0162 | ↑ 0.0232 | 3.13E-03 | 6.06E-02 | 0.8541 | ↓ -0.2276 | 9.35E-01 | 9.72E-01 | 1.0034 | ↑ 0.0049 | 6.72E-02 | 2.29E-01 | 0.8977 | ↓ -0.1557 | 3.27E-01 | 8.03E-01 | 1.0478 | ↑ 0.0674 |
| PS(18:0/22:6) | Kidney | Negative | - | * | - | - | - | - | - | - | 3.42E-02 | 4.40E-01 | 0.8723 | ↓ -0.1972 | 1.79E-03 | 5.03E-02 | 0.8045 | ↓ -0.3139 | 9.76E-01 | 9.86E-01 | 1.0189 | ↑ 0.0270 | 2.67E-01 | 4.66E-01 | 0.9197 | ↓ -0.1207 | 2.39E-01 | 7.44E-01 | 0.9065 | ↓ -0.1416 |
| Phenaceturic acid | Kidney | Negative | - | - | - | - | - | - | - | - | 3.56E-02 | 4.42E-01 | 1.6147 | ↑ 0.6912 | 7.93E-01 | 8.53E-01 | 1.3144 | ↑ 0.3944 | 1.39E-01 | 4.53E-01 | 1.8027 | ↑ 0.8502 | 9.40E-03 | 7.05E-02 | 4.0261 | ↑ 2.0094 | 3.08E-01 | 7.92E-01 | 1.0913 | ↑ 0.1260 |
| Asp | Kidney | Negative | * | * | * | * | - | - | - | - | 9.18E-02 | 6.03E-01 | 0.9230 | ↓ -0.1156 | 1.42E-02 | 1.05E-01 | 1.1405 | ↑ 0.1896 | 4.41E-02 | 3.15E-01 | 1.1469 | ↑ 0.1978 | 2.19E-02 | 1.20E-01 | 0.8859 | ↓ -0.1748 | 5.35E-01 | 8.64E-01 | 1.0310 | ↑ 0.0440 |
| PS(18:0/20:4) | Kidney | Negative | * | * | * | * | - | - | - | - | 1.88E-01 | 6.97E-01 | 1.0779 | ↑ 0.1082 | 4.96E-02 | 1.74E-01 | 0.8773 | ↓ -0.1888 | 2.71E-01 | 5.77E-01 | 0.9278 | ↓ -0.1081 | 2.51E-01 | 4.55E-01 | 1.1141 | ↑ 0.1559 | 2.15E-02 | 4.96E-01 | 0.7804 | ↓ -0.3577 |
| Carbocyclic thromboxane A2 | Kidney | Negative | * | * | * | * | - | - | x | - | 1.99E-03 | 1.11E-01 | 0.5516 | ↓ -0.8582 | 1.50E-02 | 1.06E-01 | 1.4119 | ↑ 0.4976 | 3.27E-01 | 6.29E-01 | 1.1884 | ↑ 0.2490 | 1.86E-03 | 2.16E-02 | 1.6133 | ↑ 0.6900 | 5.39E-01 | 8.64E-01 | 0.9259 | ↓ -0.1110 |
| PS(18:0/0:0) | Kidney | Negative | - | * | * | * | - | - | x | - | 3.44E-01 | 7.51E-01 | 1.1147 | ↑ 0.1566 | 1.62E-01 | 3.15E-01 | 1.1913 | ↑ 0.2525 | 6.53E-01 | 8.41E-01 | 0.9514 | ↓ -0.0718 | 6.99E-07 | 1.49E-04 | 3.1595 | ↑ 1.6597 | 6.09E-02 | 5.91E-01 | 0.7247 | ↓ -0.4645 |
| LysoPC(18:0) | Kidney | Negative | * | * | * | * | - | - | - | - | 2.04E-02 | 3.46E-01 | 0.7449 | ↓ -0.4249 | 6.58E-02 | 1.99E-01 | 1.2387 | ↑ 0.3088 | 2.55E-03 | 1.48E-01 | 1.4852 | ↑ 0.5707 | 5.29E-01 | 6.79E-01 | 0.9554 | ↓ -0.0658 | 6.73E-01 | 9.06E-01 | 1.0166 | ↑ 0.0238 |
| PC(18:0/18:1) | Kidney | Positive | - | * | * | * | - | - | x | - | 1.83E-02 | 3.40E-01 | 1.1309 | ↑ 0.1775 | 2.35E-01 | 5.86E-01 | 1.0606 | ↑ 0.0848 | 4.19E-02 | 4.73E-01 | 0.9218 | ↓ -0.1174 | 1.09E-03 | 1.38E-02 | 1.2546 | ↑ 0.3272 | 1.86E-02 | 4.27E-01 | 0.8746 | ↓ -0.1933 |
| Eicosapentaenoic acid | Kidney | Positive | * | * | * | * | - | - | - | - | 1.31E-02 | 2.77E-01 | 0.6947 | ↓ -0.5255 | 1.27E-01 | 4.78E-01 | 1.2388 | ↑ 0.3089 | 5.69E-02 | 4.73E-01 | 1.4237 | ↑ 0.5097 | 5.38E-01 | 6.86E-01 | 0.9571 | ↓ -0.0633 | 4.19E-02 | 6.80E-01 | 1.3265 | ↑ 0.4076 |
| PC(16:1/16:1) | Kidney | Positive | - | * | - | - | - | - | - | - | 2.34E-01 | 5.71E-01 | 1.0702 | ↑ 0.0979 | 2.85E-01 | 6.01E-01 | 1.0528 | ↑ 0.0742 | 3.24E-01 | 5.89E-01 | 0.9478 | ↓ -0.0774 | 1.84E-02 | 1.20E-01 | 0.8681 | ↓ -0.2040 | 2.02E-01 | 8.59E-01 | 0.9401 | ↓ -0.0891 |
| Arachidonic acid | Kidney | Positive | * | * | - | * | - | - | - | - | 8.18E-01 | 9.08E-01 | 0.9934 | ↓ -0.0096 | 9.23E-03 | 1.38E-01 | 1.2847 | ↑ 0.3614 | 3.36E-01 | 5.93E-01 | 1.1752 | ↑ 0.2329 | 7.30E-02 | 2.44E-01 | 1.2047 | ↑ 0.2687 | 6.23E-01 | 9.13E-01 | 1.0591 | ↑ 0.0829 |
| PC(P-18:0/20:4) | Kidney | Positive | - | - | * | * | - | - | x | - | 6.75E-01 | 8.35E-01 | 0.9616 | ↓ -0.0565 | 1.87E-01 | 5.64E-01 | 0.9041 | ↓ -0.1454 | 7.83E-01 | 8.77E-01 | 0.9769 | ↓ -0.0338 | 2.01E-06 | 1.86E-04 | 0.6298 | ↓ -0.6670 | 7.02E-04 | 5.72E-02 | 1.3719 | ↑ 0.4561 |
| PC(18:1/14:0) | Kidney | Positive | * | * | * | * | - | - | - | - | 8.55E-01 | 9.25E-01 | 1.0092 | ↑ 0.0132 | 4.50E-01 | 7.23E-01 | 0.9421 | ↓ -0.0860 | 1.25E-01 | 4.82E-01 | 0.8834 | ↓ -0.1789 | 4.87E-01 | 6.41E-01 | 1.0481 | ↑ 0.0678 | 1.80E-02 | 4.23E-01 | 0.8516 | ↓ -0.2318 |
| Arachidonoyl Thio-PC | Kidney | Positive | - | * | * | * | - | - | - | - | 1.22E-01 | 5.52E-01 | 1.0747 | ↑ 0.1039 | 4.72E-02 | 2.93E-01 | 1.0826 | ↑ 0.1145 | 1.04E-01 | 4.73E-01 | 0.9363 | ↓ -0.0950 | 1.14E-02 | 8.70E-02 | 1.1696 | ↑ 0.2260 | 1.25E-01 | 8.59E-01 | 0.9239 | ↓ -0.1142 |
| PC(O-16:0/22:6) | Kidney | Positive | - | * | * | * | - | - | x | - | 8.45E-01 | 9.19E-01 | 0.9821 | ↓ -0.0261 | 1.90E-01 | 5.64E-01 | 0.9125 | ↓ -0.1321 | 8.88E-01 | 9.32E-01 | 1.0091 | ↑ 0.0131 | 2.24E-06 | 1.86E-04 | 0.6417 | ↓ -0.6401 | 2.28E-04 | 5.72E-02 | 1.3956 | ↑ 0.4809 |
| LysoPC(O-18:0) | Kidney | Positive | * | * | * | * | - | - | - | - | 1.02E-01 | 5.52E-01 | 0.6853 | ↓ -0.5451 | 1.37E-02 | 1.56E-01 | 1.3270 | ↑ 0.4082 | 8.60E-02 | 4.73E-01 | 1.3757 | ↑ 0.4602 | 1.81E-02 | 1.19E-01 | 1.4744 | ↑ 0.5601 | 5.57E-01 | 8.91E-01 | 0.9069 | ↓ -0.1410 |
| PC(16:0/20:5) | Kidney | Positive | * | * | * | * | - | - | - | - | 8.39E-02 | 5.52E-01 | 0.8902 | ↓ -0.1678 | 3.02E-02 | 2.29E-01 | 1.1078 | ↑ 0.1477 | 1.69E-01 | 5.07E-01 | 1.1016 | ↑ 0.1396 | 7.37E-01 | 8.33E-01 | 1.0246 | ↑ 0.0350 | 9.50E-01 | 9.90E-01 | 0.9967 | ↓ -0.0048 |
| Phe | Kidney | Positive | * | * | - | - | - | - | - | - | 9.42E-01 | 9.77E-01 | 0.9680 | ↓ -0.0469 | 7.17E-04 | 7.34E-02 | 1.6874 | ↑ 0.7548 | 1.04E-01 | 4.73E-01 | 1.5374 | ↑ 0.6205 | 2.38E-01 | 4.21E-01 | 1.2103 | ↑ 0.2754 | 1.80E-01 | 8.59E-01 | 1.2941 | ↑ 0.3720 |
| Sphingomyelin | Kidney | Positive | * | * | - | - | - | - | - | - | 7.91E-01 | 8.93E-01 | 1.0124 | ↑ 0.0177 | 4.43E-01 | 7.23E-01 | 0.9485 | ↓ -0.0762 | 2.73E-01 | 5.54E-01 | 0.9385 | ↓ -0.0916 | 7.71E-01 | 8.49E-01 | 0.9747 | ↓ -0.0370 | 3.81E-02 | 6.39E-01 | 0.8644 | ↓ -0.2102 |
| Hypoxanthine | Kidney | Positive | - | - | * | * | - | - | - | - | 5.04E-01 | 7.35E-01 | 1.0374 | ↑ 0.0530 | 7.87E-04 | 7.34E-02 | 1.1750 | ↑ 0.2327 | 1.91E-01 | 5.14E-01 | 1.1339 | ↑ 0.1813 | 8.04E-01 | 8.70E-01 | 0.9901 | ↓ -0.0144 | 1.54E-01 | 8.59E-01 | 1.0849 | ↑ 0.1176 |
| Niacinamide | Kidney | Positive | * | * | - | * | - | - | - | - | 8.01E-01 | 8.96E-01 | 0.9641 | ↓ -0.0527 | 3.25E-03 | 9.92E-02 | 1.1722 | ↑ 0.2292 | 1.75E-01 | 5.08E-01 | 1.1544 | ↑ 0.2071 | 8.26E-01 | 8.82E-01 | 1.0109 | ↑ 0.0156 | 6.94E-01 | 9.23E-01 | 1.0359 | ↑ 0.0508 |
| LysoPC(16:0) | Kidney | Positive | * | * | * | * | - | - | x | - | 8.28E-01 | 9.12E-01 | 0.9472 | ↓ -0.0783 | 4.51E-03 | 1.05E-01 | 1.2126 | ↑ 0.2781 | 5.65E-01 | 7.56E-01 | 1.1025 | ↑ 0.1407 | 1.96E-03 | 1.99E-02 | 1.2897 | ↑ 0.3671 | 6.58E-01 | 9.13E-01 | 0.9684 | ↓ -0.0463 |

| | | | | |
|---|---|---|---|---|
| PC(18:0/22:6) | 1.91E-01 | 5.90E-01 | 1.3902 | ↑ 0.4753 |
| PS(16:0/20:4) | 4.32E-01 | 7.28E-01 | 0.9358 | ↓ -0.0957 |
| PE(P-18:0/22:6) | 1.73E-02 | 2.35E-01 | 0.6003 | ↓ -0.7362 |
| PE(18:0/22:6) | 5.88E-01 | 8.20E-01 | 1.0052 | ↑ 0.0075 |
| PC(16:0/18:0) | 2.26E-01 | 6.07E-01 | 0.8767 | ↓ -0.1899 |
| PG(16:0/18:1) | 7.41E-01 | 8.89E-01 | 1.0288 | ↑ 0.0409 |
| PS(18:0/22:6) | 4.26E-01 | 7.25E-01 | 0.9313 | ↓ -0.1026 |
| Phenaceturic acid | 3.61E-01 | 6.94E-01 | 1.0333 | ↑ 0.0472 |
| Asp | 7.65E-03 | 1.57E-01 | 1.1328 | ↑ 0.1799 |
| PS(18:0/20:4) | 6.93E-02 | 4.11E-01 | 0.7630 | ↓ -0.3903 |
| Carbocyclic thromboxane A2 | 1.48E-03 | 6.65E-02 | 0.5963 | ↓ -0.7460 |
| PS(18:0/0:0) | 9.21E-05 | 1.60E-02 | 0.4518 | ↓ -1.1461 |
| LysoPC(18:0) | 1.50E-01 | 5.52E-01 | 1.2072 | ↑ 0.2716 |
| PC(18:0/18:1) | 3.12E-02 | 4.17E-01 | 0.7701 | ↓ -0.3769 |
| Eicosapentaenoic acid | 6.80E-01 | 8.01E-01 | 1.1376 | ↑ 0.1861 |
| PC(16:1/16:1) | 7.74E-02 | 4.38E-01 | 0.8112 | ↓ -0.3019 |
| Arachidonic acid | 1.35E-01 | 4.42E-01 | 0.8210 | ↓ -0.2846 |
| PC(P-18:0/20:4) | 1.04E-01 | 4.38E-01 | 1.5037 | ↑ 0.5885 |
| PC(18:1/14:0) | 2.25E-02 | 3.77E-01 | 0.7099 | ↓ -0.4944 |
| Arachidonoyl Thio-PC | 9.31E-02 | 4.38E-01 | 0.8025 | ↓ -0.3174 |
| PC(O-16:0/22:6) | 7.14E-02 | 4.38E-01 | 1.5572 | ↑ 0.6390 |
| LysoPC(O-18:0) | 1.23E-01 | 4.38E-01 | 0.7375 | ↓ -0.4393 |
| PC(16:0/20:5) | 2.57E-01 | 5.35E-01 | 0.9145 | ↓ -0.1290 |
| Phe | 3.07E-01 | 5.64E-01 | 1.4774 | ↑ 0.5631 |
| Sphingomyelin | 4.94E-02 | 4.31E-01 | 0.7382 | ↓ -0.4380 |
| Hypoxanthine | 7.01E-01 | 8.06E-01 | 1.0497 | ↑ 0.0700 |
| Niacinamide | 5.45E-01 | 7.04E-01 | 0.9632 | ↓ -0.0541 |
| LysoPC(16:0) | 9.39E-02 | 4.38E-01 | 0.8030 | ↓ -0.3165 |

**Table 6A. Pathways Analysis of Amifostine Protection**
**Amifostine 50 mg**

| Heart | SD 4 | | SD 9 | |
|---|---|---|---|---|
| **Pathway** | *overlap size* | *p-value* | *overlap size* | *p-value* |
| Limonene and pinene degradation | 5(10) | 3.36E-04 | - | - |
| Pentose and Glucuronate Interconversions | - | - | 1(1) | 1.85E-03 |
| Glycosylphosphatidylinositol(GPI)-anchor biosynthesis | 2(3) | 7.90E-03 | - | - |
| Selenoamino acid metabolism | - | - | 1(4) | 1.07E-02 |
| Vitamin B3 (nicotinate and nicotinamide) metabolism | - | - | 1(4) | 1.07E-02 |
| Pentose phosphate pathway | - | - | 1(5) | 1.46E-02 |
| Saturated fatty acids beta-oxidation | - | - | 1(5) | 1.46E-02 |
| Xenobiotics metabolism | - | - | 1(5) | 1.46E-02 |
| Phosphatidylinositol phosphate metabolism | - | - | 1(8) | 2.41E-02 |
| Fatty Acid Metabolism | - | - | 1(8) | 2.41E-02 |
| Pyrimidine metabolism | - | - | 1(9) | 2.69E-02 |
| Purine metabolism | - | - | 1(9) | 2.69E-02 |
| Glycerophospholipid metabolism | 6(33) | 2.97E-02 | - | - |
| Fructose and mannose metabolism | 1(1) | 3.26E-02 | - | - |
| Nucleotide Sugar Metabolism | 1(1) | 3.26E-02 | - | - |
| Fatty acid activation | - | - | 1(15) | 3.87E-02 |
| Glycosphingolipid metabolism | - | - | 1(16) | 4.07E-02 |
| De novo fatty acid biosynthesis | - | - | 1(23) | 4.82E-02 |
| Sialic acid metabolism | 2(7) | 4.96E-02 | - | - |

*Note.* Pathway analysis result with positive mode and negative mode combined.

[a] Numbers in parenthesis indicates the pathway size.

**Table 6B. Pathways Analysis of Amifostine Protection**
**Amifostine 50 mg**

| **Spleen** | **SD 4** | | **SD 9** | |
|---|---|---|---|---|
| **Pathway** | ***overlap size*** | ***p-value*** | ***overlap size*** | ***p-value*** |
| Aspartate and asparagine metabolism | 7(13) | 8.40E-05 | 2(7) | 2.97E-02 |
| Valine, leucine and isoleucine degradation | 4(7) | 4.20E-04 | 2(7) | 4.20E-02 |
| Glycosphingolipid metabolism | - | - | 3(6) | 1.51E-03 |
| Histidine metabolism | 3(5) | 2.02E-03 | - | - |
| Pyrimidine metabolism | 5(16) | 2.02E-03 | - | - |
| Nitrogen metabolism | 2(2) | 4.03E-03 | - | - |
| Beta-Alanine metabolism | 3(6) | 4.37E-03 | - | - |
| Glutathione Metabolism | 3(6) | 4.37E-03 | - | - |
| Vitamin B3 (nicotinate and nicotinamide) metabolism | 3(6) | 4.37E-03 | - | - |
| Squalene and cholesterol biosynthesis | - | - | 2(2) | 4.54E-03 |
| Methionine and cysteine metabolism | 4(12) | 5.46E-03 | - | - |
| Purine metabolism | 4(13) | 6.30E-03 | - | - |
| Glutamate metabolism | 3(7) | 6.72E-03 | 2(7) | 4.20E-02 |
| Alanine and Aspartate Metabolism | 3(7) | 6.72E-03 | - | - |
| Butanoate metabolism | 3(7) | 6.72E-03 | 3(7) | 7.39E-03 |
| Glycine, serine, alanine and threonine metabolism | 4(14) | 8.82E-03 | - | - |
| Arginine and Proline Metabolism | 3(8) | 9.24E-03 | - | - |
| Tryptophan metabolism | 3(9) | 1.32E-02 | - | - |
| Putative anti-Inflammatory metabolites formation from EPA | 2(4) | 1.49E-02 | - | - |
| Urea cycle/amino group metabolism | 3(10) | 1.61E-02 | - | - |
| Lysine metabolism | 2(5) | 2.23E-02 | - | - |
| Bile acid biosynthesis | 3(12) | 2.39E-02 | - | - |
| Leukotriene metabolism | 3(12) | 2.39E-02 | - | - |
| Aminosugars metabolism | 2(6) | 3.28E-02 | - | - |
| Lipoate metabolism | - | - | 1(1) | 3.48E-02 |
| N-Glycan Degradation | - | - | 1(1) | 3.48E-02 |
| C21-steroid hormone biosynthesis and metabolism | 2(7) | 3.67E-02 | - | - |
| Vitamin B2 (riboflavin) metabolism | 1(1) | 3.68E-02 | - | - |
| Alkaloid biosynthesis II | 1(1) | 3.68E-02 | - | - |
| Vitamin B1 (thiamin) metabolism | 1(1) | 3.68E-02 | - | - |
| Vitamin E metabolism | 1(1) | 3.68E-02 | - | - |
| Mono-unsaturated fatty acid beta-oxidation | 1(1) | 3.68E-02 | - | - |
| Ubiquinone Biosynthesis | 1(1) | 3.68E-02 | - | - |
| Arachidonic acid metabolism | 3(14) | 3.73E-02 | - | - |

*Note.* Pathway analysis result with positive mode and negative mode combined.

[a] Numbers in parenthesis indicates the pathway size.

**Table 6C. Pathways Analysis of Amifostine Protection**
**Amifostine 50 mg**

| **Kidney** | **SD 4** | | **SD 9** | |
|---|---|---|---|---|
| **Pathway** | ***overlap size*** | ***p-value*** | ***overlap size*** | ***p-value*** |
| Beta-Alanine metabolism | - | - | 5(6) | 6.72E-04 |
| Glutathione Metabolism | - | - | 3(3) | 3.87E-03 |
| Vitamin A (retinol) metabolism | 5(7) | 4.61E-02 | 3(9) | 6.97E-03 |
| Glycerophospholipid metabolism | 11(19) | 2.97E-02 | 3(10) | 9.16E-03 |
| Glycine, serine, alanine and threonine metabolism | - | - | 6(13) | 1.14E-02 |
| Vitamin D3 (cholecalciferol) metabolism | 4(4) | 1.21E-02 | - | - |
| Histidine metabolism | - | - | 3(4) | 1.31E-02 |
| Tryptophan metabolism | 3(4) | 1.49E-02 | - | - |
| Tyrosine metabolism | 6(11) | 1.58E-02 | - | - |
| Vitamin B9 (folate) metabolism | - | - | 1(1) | 1.86E-02 |
| Linoleate metabolism | 6(8) | 1.98E-02 | - | - |
| Methionine and cysteine metabolism | - | - | 5(11) | 2.10E-02 |
| Glutamate metabolism | - | - | 3(5) | 2.76E-02 |
| Omega-3 fatty acid metabolism | 3(3) | 3.15E-02 | - | - |
| Sialic acid metabolism | - | - | 1(2) | 4.22E-02 |
| Hexose phosphorylation | - | - | 1(2) | 4.22E-02 |
| Ubiquinone Biosynthesis | - | - | 1(2) | 4.22E-02 |
| Purine metabolism | 3(5) | 4.67E-02 | - | - |

*Note.* Pathway analysis result with positive mode and negative mode combined.

[a] Numbers in parenthesis indicates the pathway size.

**Table 6D. Pathways Analysis of Amifostine Protection**
**Amifostine 200 mg**

| Heart | SD 4 | | SD 9 | |
|---|---|---|---|---|
| **Pathway** | *overlap size* | *p-value* | *overlap size* | *p-value* |
| Fatty acid activation | - | - | 6(15) | 1.68E-04 |
| De novo fatty acid biosynthesis | - | - | 7(23) | 6.72E-04 |
| Xenobiotics metabolism | - | - | 3(5) | 2.27E-03 |
| Fructose and mannose metabolism | 1(1) | 5.80E-03 | - | - |
| Nucleotide Sugar Metabolism | 1(1) | 5.80E-03 | - | - |
| Linoleate metabolism | - | - | 4(12) | 5.88E-03 |
| Fatty Acid Metabolism | - | - | 3(8) | 8.65E-03 |
| Polyunsaturated fatty acid biosynthesis | 1(2) | 1.13E-02 | - | - |
| Glycolysis and Gluconeogenesis | 1(2) | 1.13E-02 | - | - |
| Hexose phosphorylation | 1(2) | 1.13E-02 | - | - |
| Galactose metabolism | 1(2) | 1.13E-02 | - | - |
| Arachidonic acid metabolism | - | - | 3(9) | 1.21E-02 |
| Omega-3 fatty acid metabolism | - | - | 2(4) | 1.27E-02 |
| Pyrimidine metabolism | 2(9) | 1.37E-02 | - | - |
| Glycosphingolipid metabolism | - | - | 4(16) | 1.51E-02 |
| Starch and Sucrose Metabolism | 1(3) | 2.03E-02 | - | - |
| Glycosylphosphatidylinositol(GPI)-anchor biosynthesis | 1(3) | 2.03E-02 | - | - |
| Saturated fatty acids beta-oxidation | - | - | 2(5) | 2.12E-02 |
| N-Glycan biosynthesis | 1(4) | 2.64E-02 | - | - |
| Nitrogen metabolism | 1(2) | 2.66E-02 | - | - |
| Valine, leucine and isoleucine degradation | 1(2) | 2.66E-02 | - | - |
| Fatty acid oxidation, peroxisome | - | - | 1(1) | 2.92E-02 |
| Aminosugars metabolism | 1(5) | 3.51E-02 | - | - |
| Pentose phosphate pathway | 1(5) | 3.51E-02 | - | - |
| Arginine and Proline Metabolism | 1(3) | 4.18E-02 | - | - |
| Butanoate metabolism | 1(3) | 4.18E-02 | - | - |
| Alanine and Aspartate Metabolism | 1(3) | 4.18E-02 | - | - |

*Note.* Pathway analysis result with positive mode and negative mode combined.

[a] Numbers in parenthesis indicates the pathway size.

**Table 6E. Pathways Analysis of Amifostine Protection**
**Amifostine 200 mg**

| Spleen | SD 4 | | SD 9 | |
|---|---|---|---|---|
| **Pathway** | ***overlap size*** | ***p-value*** | ***overlap size*** | ***p-value*** |
| Glycolysis and Gluconeogenesis | - | - | 7(7) | 5.88E-04 |
| Pentose phosphate pathway | - | - | 7(9) | 6.72E-04 |
| Glycine, serine, alanine and threonine metabolism | - | - | 8(10) | 7.56E-04 |
| Tryptophan metabolism | 4(9) | 1.26E-03 | 7(9) | 8.82E-03 |
| Aspartate and asparagine metabolism | 3(13) | 3.25E-02 | 6(7) | 1.43E-03 |
| Fructose and mannose metabolism | - | - | 5(6) | 3.53E-03 |
| Prostaglandin formation from arachidonate | 6(11) | 4.45E-03 | - | - |
| Vitamin B3 (nicotinate and nicotinamide) metabolism | 3(6) | 4.71E-03 | 5(6) | 1.56E-02 |
| Pyruvate Metabolism | - | - | 5(5) | 4.96E-03 |
| Butanoate metabolism | 1(2) | 1.97E-02 | 6(7) | 6.81E-03 |
| Ascorbate (Vitamin C) and Aldarate Metabolism | - | - | 6(7) | 6.81E-03 |
| Galactose metabolism | - | - | 6(7) | 6.81E-03 |
| Polyunsaturated fatty acid biosynthesis | 1(1) | 7.73E-03 | - | - |
| Valine, leucine and isoleucine degradation | 1(3) | 2.77E-02 | 3(3) | 8.57E-03 |
| Pyrimidine metabolism | 4(16) | 1.43E-02 | 3(3) | 8.57E-03 |
| Lysine metabolism | - | - | 3(3) | 8.57E-03 |
| Purine metabolism | - | - | 4(5) | 1.03E-02 |
| Fatty acid activation | 4(7) | 1.08E-02 | - | - |
| TCA cycle | - | - | 4(4) | 1.21E-02 |
| Propanoate metabolism | - | - | 4(4) | 1.21E-02 |
| Glycerophospholipid metabolism | - | - | 9(19) | 1.32E-02 |
| Urea cycle/amino group metabolism | 3(10) | 1.77E-02 | - | - |
| Fatty Acid Metabolism | 3(5) | 1.88E-02 | - | - |
| De novo fatty acid biosynthesis | 5(12) | 2.56E-02 | - | - |
| Drug metabolism - cytochrome P450 | - | - | 3(4) | 2.92E-02 |
| Glutamate metabolism | 2(7) | 4.89E-02 | 3(4) | 3.03E-02 |
| Glutathione Metabolism | - | - | 3(4) | 3.03E-02 |
| Putative anti-Inflammatory metabolites formation from EPA | - | - | 2(2) | 3.18E-02 |
| Histidine metabolism | - | - | 2(2) | 3.18E-02 |
| Arginine and Proline Metabolism | - | - | 2(2) | 3.18E-02 |
| Beta-Alanine metabolism | 2(6) | 3.53E-02 | 2(2) | 3.18E-02 |
| Trihydroxycoprostanoyl-CoA beta-oxidation | - | - | 2(2) | 3.18E-02 |
| Glycosphingolipid metabolism | 3(6) | 3.39E-02 | - | - |
| Aminosugars metabolism | 2(6) | 3.53E-02 | - | - |
| Fatty acid oxidation, peroxisome | - | - | 2(2) | 3.74E-02 |
| Parathio degradation | 1(1) | 3.92E-02 | - | - |

*Note.* Pathway analysis result with positive mode and negative mode combined.

[a] Numbers in parenthesis indicates the pathway size.

**Table 6F. Pathways Analysis of Amifostine Protection**
**Amifostine 200 mg**

| **Kidney** | **SD 4** | | **SD 9** | |
|---|---|---|---|---|
| **Pathway** | ***overlap size*** | ***p-value*** | ***overlap size*** | ***p-value*** |
| Beta-Alanine metabolism | 6(6) | 1.09E-03 | 6(6) | 5.88E-04 |
| Valine, leucine and isoleucine degradation | 2(3) | 7.56E-04 | - | - |
| Butanoate metabolism | 8(10) | 1.51E-03 | - | - |
| Tryptophan metabolism | 2(4) | 1.76E-03 | 3(10) | 2.34E-02 |
| Pyruvate Metabolism | 5(5) | 2.27E-03 | - | - |
| Aspartate and asparagine metabolism | 10(15) | 2.69E-03 | - | - |
| Ascorbate (Vitamin C) and Aldarate Metabolism | 4(8) | 2.86E-03 | 6(8) | 7.31E-03 |
| Alanine and Aspartate Metabolism | 6(7) | 3.19E-03 | - | - |
| Methionine and cysteine metabolism | 8(11) | 3.36E-03 | 1(3) | 2.20E-02 |
| Vitamin B9 (folate) metabolism | - | - | 1(1) | 5.55E-03 |
| Histidine metabolism | 4(4) | 5.97E-03 | 3(4) | 4.68E-02 |
| Vitamin B3 (nicotinate and nicotinamide) metabolism | - | - | 3(6) | 7.31E-03 |
| Glycerophospholipid metabolism | 4(19) | 3.65E-02 | 2(10) | 7.65E-03 |
| Pentose phosphate pathway | 3(7) | 1.14E-02 | - | - |
| Sialic acid metabolism | 3(8) | 1.60E-02 | 1(2) | 1.34E-02 |
| Chondroitin sulfate degradation | 2(3) | 1.47E-02 | - | - |
| Glycosphingolipid biosynthesis - globoseries | 2(3) | 1.47E-02 | - | - |
| Glyoxylate and Dicarboxylate Metabolism | 2(3) | 1.47E-02 | - | - |
| Glycine, serine, alanine and threonine metabolism | 8(13) | 1.54E-02 | 1(5) | 3.62E-02 |
| Biopterin metabolism | - | - | 2(4) | 1.87E-02 |
| Arginine and Proline Metabolism | 6(9) | 2.03E-02 | - | - |
| Aminosugars metabolism | 6(9) | 2.03E-02 | - | - |
| Phosphatidylinositol phosphate metabolism | 3(9) | 2.06E-02 | - | - |
| Glutathione Metabolism | 3(3) | 2.08E-02 | - | - |
| Vitamin B1 (thiamin) metabolism | 3(3) | 2.08E-02 | - | - |
| Glycosphingolipid metabolism | - | - | 1(3) | 2.20E-02 |
| Selenoamino acid metabolism | - | - | 1(3) | 2.20E-02 |
| Heparan sulfate degradation | 2(4) | 2.24E-02 | - | - |
| Glycosphingolipid biosynthesis - ganglioseries | 2(4) | 2.24E-02 | - | - |
| Glycolysis and Gluconeogenesis | 5(7) | 2.25E-02 | - | - |
| Glutamate metabolism | 4(5) | 2.39E-02 | - | - |
| Purine metabolism | 8(14) | 2.49E-02 | - | - |
| Vitamin B6 (pyridoxine) metabolism | 1(2) | 2.55E-02 | - | - |
| Propanoate metabolism | 1(2) | 2.57E-02 | - | - |
| Leukotriene metabolism | - | - | 1(4) | 2.98E-02 |
| Pentose and Glucuronate Interconversions | 2(5) | 3.34E-02 | - | - |
| Urea cycle/amino group metabolism | - | - | 3(12) | 3.74E-02 |
| Parathio degradation | - | - | 1(1) | 4.13E-02 |
| Ubiquinone Biosynthesis | - | - | 1(2) | 4.57E-02 |
| Hexose phosphorylation | - | - | 1(2) | 4.57E-02 |
| N-Glycan biosynthesis | 2(6) | 4.77E-02 | - | - |
| N-Glycan Degradation | 1(1) | 4.81E-02 | - | - |
| Proteoglycan biosynthesis | 1(1) | 4.81E-02 | - | - |
| Keratan sulfate degradation | 1(1) | 4.81E-02 | - | - |

*Note.* Pathway analysis result with positive mode and negative mode combined.

[a] Numbers in parenthesis indicates the pathway size.

SD 4

**Heart**

| pathway | overlap size | pathway size | p-value | overlap Empirical Compounds | overlap features | overlap features name |
|---|---|---|---|---|---|---|
| Limonene and pinene degradation | 5 | 10 | 3.36E-04 | E474,E14,E169,E347,E402 | C06306,C02759/C11409/C02452/C00964,C02759/C11409/C02452/C00964,C06099/C06078/C00521,C02576 | S/(+)-trans-Carveol; (4S,6R)-trans-Carveol/Perillyl alcohol; (-)-Perillyl alcohol; p-Mentha-1,8-dien-7-ol; (-)-Perillylalcohol/(-)-trans-Carveol$(+)-trans-Carveol; (4S,6R)-trans-Carveol/Perillyl alcohol; (-)-Perillyl alcohol; p-Mentha-1,8-dien-7-ol; (-)-Perillylalcohol/(-)-trans-Carveol$(+)-Limonene; (+)-(R)-Limonene; (+)-( |
| Pentose and Glucuronate Interconversions | - | - | - | - | - | - |
| Carbon fixation | - | - | - | - | - | - |
| Glycosylphosphatidylinositol(GPI)-anchor biosynthesis | 2 | 3 | 0.007898496 | E912,E222 | C00165,C00092/C02965/C05345/C00085/C00103/C02976/C01171/C01177/C03735/C00636/C01172/C03862/C04006/C01097/C01094/C00668/C03546/C00446/C00275/CE3075 | Diacylglycerol; Diglyceride$D-Glucose 6-phosphate; Glucose 6-phosphate; Robison ester/D-Hexose 6-phosphate/beta-D-Fructose 6-phosphate/D-Fructose 6-phosphate; D-Fructose 6-phosphoric acid; Neuberg ester/D-Glucose 1-phosphate; alpha-D-Glucose 1-phosphate; Cori ester; D-Glucose alpha-1-phosphate, |
| Selenoamino acid metabolism | - | - | - | - | - | - |
| Vitamin B3 (nicotinate and nicotinamide) metabolism | - | - | - | - | - | - |
| Pentose phosphate pathway | - | - | - | - | - | - |
| Saturated fatty acids beta-oxidation | - | - | - | - | - | - |
| Xenobiotics metabolism | - | - | - | - | - | - |
| Phosphatidylinositol phosphate metabolism | - | - | - | - | - | - |
| Fatty Acid Metabolism | - | - | - | - | - | - |
| Pyrimidine metabolism | - | - | - | - | - | - |
| Purine metabolism | - | - | - | - | - | - |
| Glycerophospholipid metabolism | 6 | 33 | 0.029661373 | E730,E912,E763,E143,E222,E150 | C00319/C02934,C00165,C01595,C00219,C00092/C02965/C05345/C00085/C00103/C02976/C01171/C01177/C03735/C00636/C01172/C03862/C04006/C01097/C01094/C00668/C03546/C00446/C00275/CE3075,C00307 | Sphingosine; Sphingenine; Sphingoid; Sphing-4-enine/3-Dehydrosphinganine; 3-Dehydro-D-sphinganine$Diacylglycerol; Diglyceride$Linoleate; Linoleic acid; (9Z,12Z)-Octadecadienoic acid; 9-cis,12-cis-Octadecadienoate; 9-cis,12-cis-Octadecadienoic acid$(5Z,8Z,11Z,14Z)-Icosatetraenoic acid; Arachidonate; Arach |
| Fructose and mannose metabolism | 1 | 1 | 0.032602302 | E222 | C00092/C02965/C05345/C00085/C00103/C02976/C01171/C01177/C03735/C00636/C01172/C03862/C04006/C01097/C01094/C00668/C03546/C00446/C00275/CE3075 | D-Glucose 6-phosphate; Glucose 6-phosphate; Robison ester/D-Hexose 6-phosphate/beta-D-Fructose 6-phosphate/D-Fructose 6-phosphate; D-Fructose 6-phosphoric acid; Neuberg ester/D-Glucose 1-phosphate; alpha-D-Glucose 1-phosphate; Cori ester; D-Glucose alpha-1-phosphate/Fructose 1-phosphate/alph |
| Nucleotide Sugar Metabolism | 1 | 1 | 0.032602302 | E222 | C00092/C02965/C05345/C00085/C00103/C02976/C01171/C01177/C03735/C00636/C01172/C03862/C04006/C01097/C01094/C00668/C03546/C00446/C00275/CE3075 | D-Glucose 6-phosphate; Glucose 6-phosphate; Robison ester/D-Hexose 6-phosphate/beta-D-Fructose 6-phosphate/D-Fructose 6-phosphate; D-Fructose 6-phosphoric acid; Neuberg ester/D-Glucose 1-phosphate; alpha-D-Glucose 1-phosphate; Cori ester; D-Glucose alpha-1-phosphate/Fructose 1-phosphate/alph |
| Fatty acid activation | - | - | - | - | - | - |
| Glycosphingolipid metabolism | - | - | - | - | - | - |
| De novo fatty acid biosynthesis | - | - | - | - | - | - |
| Sialic acid metabolism | 2 | 7 | 0.049575666 | E912,E222 | C00165,C00092/C02965/C05345/C00085/C00103/C02976/C01171/C01177/C03735/C00636/C01172/C03862/C04006/C01097/C01094/C00668/C03546/C00446/C00275/CE3075 | Diacylglycerol; Diglyceride$D-Glucose 6-phosphate; Glucose 6-phosphate; Robison ester/D-Hexose 6-phosphate/beta-D-Fructose 6-phosphate/D-Fructose 6-phosphate; D-Fructose 6-phosphoric acid; Neuberg ester/D-Glucose 1-phosphate; alpha-D-Glucose 1-phosphate; Cori ester; D-Glucose alpha-1-phosphate, |

SD 4

**Spleen**

| pathway | overlap size | pathway size | p-value | overlap Empirical Compounds | overlap features | overlap features name |
|---|---|---|---|---|---|---|
| Aspartate and asparagine metabolism | 7 | 13 | 8.40E-05 | E701,E540,E719,E358,E69,E351,E514 | C00217/C05938/C00025,C02238,C00402/C00049,C00362/C00020,C01026/C01205/C02356/C00334/C03284/C05145,C04281/C04282/C01879,C00051 | D-Glutamate; D-Glutamic acid; D-Glutaminic acid; D-2-Aminoglutaric acid/L-4-Hydroxyglutamate semialdehyde/L-Glutamate; L-Glutamic acid; L-Glutaminic acid; Glutamate$5-Oxo-L-proline$D-Aspartate; D-Aspartic acid/L-Aspartate; L-Aspartic acid; 2-Aminosuccinic acid$dGMP; 2'-Deoxyguanosine 5'-monophos |
| Valine, leucine and isoleucine degradation | 4 | 7 | 4.20E-04 | E69,E701,E358,E120 | C01026/C01205/C02356/C00334/C03284/C05145,C00217/C05938/C00025,C00362/C00020,C00407/C00123 | N,N-Dimethylglycine; Dimethylglycine/(R)-3-Amino-2-methylpropanoate; D-3-Amino-isobutanoate/(S)-2-Aminobutanoate; (S)-2-Aminobutanoic acid; (S)-2-Aminobutyric acid/4-Aminobutanoate; 4-Aminobutanoic acid; 4-Aminobutyrate; 4-Aminobutyric acid; gamma-Aminobutyric acid/L-3-Amino-isobutanoate |
| Glycosphingolipid metabolism | - | - | - | - | - | - |
| Histidine metabolism | 3 | 5 | 0.002016637 | E514,E701,E358 | C00051,C00217/C05938/C00025,C00362/C00020 | Glutathione; 5-L-Glutamyl-L-cysteinylglycine; N-(N-gamma-L-Glutamyl-L-cysteinyl)glycine; gamma-L-Glutamyl-L-cysteinyl-glycine; GSH; Reduced glutathione$D-Glutamate; D-Glutamic acid; D-Glutaminic acid; D-2-Aminoglutaric acid/L-4-Hydroxyglutamate semialdehyde/L-Glutamate; L-Glutamic acid; L-Glutamir |
| Pyrimidine metabolism | 5 | 16 | 0.002016637 | E719,E701,E69,E358,E49 | C00402/C00049,C00217/C05938/C00025,C01026/C01205/C02356/C00334/C03284/C05145,C00362/C00020,C00055 | D-Aspartate; D-Aspartic acid/L-Aspartate; L-Aspartic acid; 2-Aminosuccinic acid$D-Glutamate; D-Glutamic acid; D-Glutaminic acid; D-2-Aminoglutaric acid/L-4-Hydroxyglutamate semialdehyde/L-Glutamate; L-Glutamic acid; L-Glutaminic acid; Glutamate$N,N-Dimethylglycine; Dimethylglycine/(R)-3-Amino-2-met |
| Nitrogen metabolism | 2 | 2 | 0.004033275 | E719,E701 | C00402/C00049,C00217/C05938/C00025 | D-Aspartate; D-Aspartic acid/L-Aspartate; L-Aspartic acid; 2-Aminosuccinic acid$D-Glutamate; D-Glutamic acid; D-Glutaminic acid; D-2-Aminoglutaric acid/L-4-Hydroxyglutamate semialdehyde/L-Glutamate; L-Glutamic acid; L-Glutaminic acid; Glutamate |
| Beta-Alanine metabolism | 3 | 6 | 0.004369381 | E719,E701,E69 | C00402/C00049,C00217/C05938/C00025,C01026/C01205/C02356/C00334/C03284/C05145 | D-Aspartate; D-Aspartic acid/L-Aspartate; L-Aspartic acid; 2-Aminosuccinic acid$D-Glutamate; D-Glutamic acid; D-Glutaminic acid; D-2-Aminoglutaric acid/L-4-Hydroxyglutamate semialdehyde/L-Glutamate; L-Glutamic acid; L-Glutaminic acid; Glutamate$N,N-Dimethylglycine; Dimethylglycine/(R)-3-Amino-2-met |
| Glutathione Metabolism | 3 | 6 | 0.004369381 | E514,E701,E351 | C00051,C00217/C05938/C00025,C04281/C04282/C01879 | Glutathione; 5-L-Glutamyl-L-cysteinylglycine; N-(N-gamma-L-Glutamyl-L-cysteinyl)glycine; gamma-L-Glutamyl-L-cysteinyl-glycine; GSH; Reduced glutathione$D-Glutamate; D-Glutamic acid; D-Glutaminic acid; D-2-Aminoglutaric acid/L-4-Hydroxyglutamate semialdehyde/L-Glutamate; L-Glutamic acid; L-Glutamir |
| Vitamin B3 (nicotinate and nicotinamide) metabolism | 3 | 6 | 0.004369381 | E719,E701,E358 | C00402/C00049,C00217/C05938/C00025,C00362/C00020 | D-Aspartate; D-Aspartic acid/L-Aspartate; L-Aspartic acid; 2-Aminosuccinic acid$D-Glutamate; D-Glutamic acid; D-Glutaminic acid; D-2-Aminoglutaric acid/L-4-Hydroxyglutamate semialdehyde/L-Glutamate; L-Glutamic acid; L-Glutaminic acid; Glutamate$dGMP; 2'-Deoxyguanosine 5'-monophosphate; 2'-Deoxyg |
| Squalene and cholesterol biosynthesis | - | - | - | - | - | - |
| Methionine and cysteine metabolism | 4 | 12 | 0.005461726 | E514,E701,E358,E657 | C00051,C00217/C05938/C00025,C00362/C00020,C00302 | Glutathione; 5-L-Glutamyl-L-cysteinylglycine; N-(N-gamma-L-Glutamyl-L-cysteinyl)glycine; gamma-L-Glutamyl-L-cysteinyl-glycine; GSH; Reduced glutathione$D-Glutamate; D-Glutamic acid; D-Glutaminic acid; D-2-Aminoglutaric acid/L-4-Hydroxyglutamate semialdehyde/L-Glutamate; L-Glutamic acid; L-Glutamir |
| Purine metabolism | 4 | 13 | 0.006301991 | E719,E701,E358,E113 | C00402/C00049,C00217/C05938/C00025,C00362/C00020,C00366 | D-Aspartate; D-Aspartic acid/L-Aspartate; L-Aspartic acid; 2-Aminosuccinic acid$D-Glutamate; D-Glutamic acid; D-Glutaminic acid; D-2-Aminoglutaric acid/L-4-Hydroxyglutamate semialdehyde/L-Glutamate; L-Glutamic acid; L-Glutaminic acid; Glutamate$dGMP; 2'-Deoxyguanosine 5'-monophosphate; 2'-Deoxyg |
| Glutamate metabolism | 3 | 7 | 0.006722124 | E69,E701,E514 | C01026/C01205/C02356/C00334/C03284/C05145,C00217/C05938/C00025,C00051 | N,N-Dimethylglycine; Dimethylglycine/(R)-3-Amino-2-methylpropanoate; D-3-Amino-isobutanoate/(S)-2-Aminobutanoate; (S)-2-Aminobutanoic acid; (S)-2-Aminobutyric acid/4-Aminobutanoate; 4-Aminobutanoic acid; 4-Aminobutyrate; 4-Aminobutyric acid; gamma-Aminobutyric acid/L-3-Amino-isobutanoate |
| Alanine and Aspartate Metabolism | 3 | 7 | 0.006722124 | E719,E701,E358 | C00402/C00049,C00217/C05938/C00025,C00362/C00020 | D-Aspartate; D-Aspartic acid/L-Aspartate; L-Aspartic acid; 2-Aminosuccinic acid$D-Glutamate; D-Glutamic acid; D-Glutaminic acid; D-2-Aminoglutaric acid/L-4-Hydroxyglutamate semialdehyde/L-Glutamate; L-Glutamic acid; L-Glutaminic acid; Glutamate$dGMP; 2'-Deoxyguanosine 5'-monophosphate; 2'-Deoxyg |
| Butanoate metabolism | 3 | 7 | 0.006722124 | E69,E701,E358 | C01026/C01205/C02356/C00334/C03284/C05145,C00217/C05938/C00025,C00362/C00020 | N,N-Dimethylglycine; Dimethylglycine/(R)-3-Amino-2-methylpropanoate; D-3-Amino-isobutanoate/(S)-2-Aminobutanoate; (S)-2-Aminobutanoic acid; (S)-2-Aminobutyric acid/4-Aminobutanoate; 4-Aminobutanoic acid; 4-Aminobutyrate; 4-Aminobutyric acid; gamma-Aminobutyric acid/L-3-Amino-isobutanoate |
| Glycine, serine, alanine and threonine metabolism | 4 | 14 | 0.008822788 | E69,E701,E358,E514 | C01026/C01205/C02356/C00334/C03284/C05145,C00217/C05938/C00025,C00362/C00020,C00051 | N,N-Dimethylglycine; Dimethylglycine/(R)-3-Amino-2-methylpropanoate; D-3-Amino-isobutanoate/(S)-2-Aminobutanoate; (S)-2-Aminobutanoic acid; (S)-2-Aminobutyric acid/4-Aminobutanoate; 4-Aminobutanoic acid; 4-Aminobutyrate; 4-Aminobutyric acid; gamma-Aminobutyric acid/L-3-Amino-isobutanoate |
| Arginine and Proline Metabolism | 3 | 8 | 0.009242921 | E719,E701,E351 | C00402/C00049,C00217/C05938/C00025,C04281/C04282/C01879 | D-Aspartate; D-Aspartic acid/L-Aspartate; L-Aspartic acid; 2-Aminosuccinic acid$D-Glutamate; D-Glutamic acid; D-Glutaminic acid; D-2-Aminoglutaric acid/L-4-Hydroxyglutamate semialdehyde/L-Glutamate; L-Glutamic acid; L-Glutaminic acid; Glutamate$L-1-Pyrroline-3-hydroxy-5-carboxylate; 3-Hydroxy-L-1-pyr |
| Tryptophan metabolism | 3 | 9 | 0.013192169 | E514,E701,E358 | C00051,C00217/C05938/C00025,C00362/C00020 | Glutathione; 5-L-Glutamyl-L-cysteinylglycine; N-(N-gamma-L-Glutamyl-L-cysteinyl)glycine; gamma-L-Glutamyl-L-cysteinyl-glycine; GSH; Reduced glutathione$D-Glutamate; D-Glutamic acid; D-Glutaminic acid; D-2-Aminoglutaric acid/L-4-Hydroxyglutamate semialdehyde/L-Glutamate; L-Glutamic acid; L-Glutamir |
| Putative anti-Inflammatory metabolites formation from EPA | 2 | 4 | 0.0148727 | E514,E701 | C00051,C00217/C05938/C00025 | Glutathione; 5-L-Glutamyl-L-cysteinylglycine; N-(N-gamma-L-Glutamyl-L-cysteinyl)glycine; gamma-L-Glutamyl-L-cysteinyl-glycine; GSH; Reduced glutathione$D-Glutamate; D-Glutamic acid; D-Glutaminic acid; D-2-Aminoglutaric acid/L-4-Hydroxyglutamate semialdehyde/L-Glutamate; L-Glutamic acid; L-Glutamir |
| Urea cycle/amino group metabolism | 3 | 10 | 0.016133098 | E719,E701,E69 | C00402/C00049,C00217/C05938/C00025,C01026/C01205/C02356/C00334/C03284/C05145 | D-Aspartate; D-Aspartic acid/L-Aspartate; L-Aspartic acid; 2-Aminosuccinic acid$D-Glutamate; D-Glutamic acid; D-Glutaminic acid; D-2-Aminoglutaric acid/L-4-Hydroxyglutamate semialdehyde/L-Glutamate; L-Glutamic acid; L-Glutaminic acid; Glutamate$N,N-Dimethylglycine; Dimethylglycine/(R)-3-Amino-2-met |
| Lysine metabolism | 2 | 5 | 0.022267036 | E701,E358 | C00217/C05938/C00025,C00362/C00020 | D-Glutamate; D-Glutamic acid; D-Glutaminic acid; D-2-Aminoglutaric acid/L-4-Hydroxyglutamate semialdehyde/L-Glutamate; L-Glutamic acid; L-Glutaminic acid; Glutamate$dGMP; 2'-Deoxyguanosine 5'-monophosphate; 2'-Deoxyguanosine 5'-phosphate; Deoxyguanylic acid; Deoxyguanosine monophosphate/A |
| Bile acid biosynthesis | 3 | 12 | 0.023947567 | E424,E358,E581 | C04483/C02528,C00362/C00020,C15517 | 3alpha,12alpha-Dihydroxy-5beta-cholanate; Deoxycholic acid; Deoxycholate; 3alpha,12alpha-Dihydroxy-5beta-cholanic acid/Chenodeoxycholate; Chenodeoxycholic acid; 3alpha,7alpha-Dihydroxy-5beta-cholanic acid; Chenodiol$dGMP; 2'-Deoxyguanosine 5'-monophosphate; 2'-Deoxyguanosine 5'-phosphate; De |
| Leukotriene metabolism | 3 | 12 | 0.023947567 | E514,E358,E657 | C00051,C00362/C00020,C00302 | Glutathione; 5-L-Glutamyl-L-cysteinylglycine; N-(N-gamma-L-Glutamyl-L-cysteinyl)glycine; gamma-L-Glutamyl-L-cysteinyl-glycine; GSH; Reduced glutathione$dGMP; 2'-Deoxyguanosine 5'-monophosphate; 2'-Deoxyguanosine 5'-phosphate; Deoxyguanylic acid; Deoxyguanosine monophosphate/AMP; Adenosine |
| Aminosugars metabolism | 2 | 6 | 0.032770355 | E701,E49 | C00217/C05938/C00025,C00055 | D-Glutamate; D-Glutamic acid; D-Glutaminic acid; D-2-Aminoglutaric acid/L-4-Hydroxyglutamate semialdehyde/L-Glutamate; L-Glutamic acid; L-Glutaminic acid; Glutamate$CMP; Cytidine-5'-monophosphate; Cytidylic acid |
| Lipoate metabolism | - | - | - | - | - | - |
| N-Glycan Degradation | - | - | - | - | - | - |
| C21-steroid hormone biosynthesis and metabolism | 2 | 7 | 0.036719603 | E484,E407 | C05487/C05475/C05478,C05472 | 17alpha,21-Dihydroxypregnenolone/11beta,21-Dihydroxy-5beta-pregnane-3,20-dione; 5beta-Pregnane-11beta,21-diol-3,20-dione/3alpha,21-Dihydroxy-5beta-pregnane-11,20-dione; 5beta-Pregnane-3alpha,21-diol-11,20-dione$Urocortisol; 5beta-Pregnane-3alpha,11beta,17alpha,21-tetrol-20-one |
| Vitamin B2 (riboflavin) metabolism | 1 | 1 | 0.03680363 | E358 | C00362/C00020 | dGMP; 2'-Deoxyguanosine 5'-monophosphate; 2'-Deoxyguanosine 5'-phosphate; Deoxyguanylic acid; Deoxyguanosine monophosphate/AMP; Adenosine 5'-monophosphate; Adenylic acid; Adenylate; 5'-AMP; 5'-Adenylic acid; 5'-Adenosine monophosphate; Adenosine 5'-phosphate |
| Alkaloid biosynthesis II | 1 | 1 | 0.03680363 | E358 | C00362/C00020 | dGMP; 2'-Deoxyguanosine 5'-monophosphate; 2'-Deoxyguanosine 5'-phosphate; Deoxyguanylic acid; Deoxyguanosine monophosphate/AMP; Adenosine 5'-monophosphate; Adenylic acid; Adenylate; 5'-AMP; 5'-Adenylic acid; 5'-Adenosine monophosphate; Adenosine 5'-phosphate |
| Vitamin B1 (thiamin) metabolism | 1 | 1 | 0.03680363 | E358 | C00362/C00020 | dGMP; 2'-Deoxyguanosine 5'-monophosphate; 2'-Deoxyguanosine 5'-phosphate; Deoxyguanylic acid; Deoxyguanosine monophosphate/AMP; Adenosine 5'-monophosphate; Adenylic acid; Adenylate; 5'-AMP; 5'-Adenylic acid; 5'-Adenosine monophosphate; Adenosine 5'-phosphate |
| Vitamin E metabolism | 1 | 1 | 0.03680363 | E358 | C00362/C00020 | dGMP; 2'-Deoxyguanosine 5'-monophosphate; 2'-Deoxyguanosine 5'-phosphate; Deoxyguanylic acid; Deoxyguanosine monophosphate/AMP; Adenosine 5'-monophosphate; Adenylic acid; Adenylate; 5'-AMP; 5'-Adenylic acid; 5'-Adenosine monophosphate; Adenosine 5'-phosphate |
| Mono-unsaturated fatty acid beta-oxidation | 1 | 1 | 0.03680363 | E358 | C00362/C00020 | dGMP; 2'-Deoxyguanosine 5'-monophosphate; 2'-Deoxyguanosine 5'-phosphate; Deoxyguanylic acid; Deoxyguanosine monophosphate/AMP; Adenosine 5'-monophosphate; Adenylic acid; Adenylate; 5'-AMP; 5'-Adenylic acid; 5'-Adenosine monophosphate; Adenosine 5'-phosphate |
| Ubiquinone Biosynthesis | 1 | 1 | 0.03680363 | E358 | C00362/C00020 | dGMP; 2'-Deoxyguanosine 5'-monophosphate; 2'-Deoxyguanosine 5'-phosphate; Deoxyguanylic acid; Deoxyguanosine monophosphate/AMP; Adenosine 5'-monophosphate; Adenylic acid; Adenylate; 5'-AMP; 5'-Adenylic acid; 5'-Adenosine monophosphate; Adenosine 5'-phosphate |
| Arachidonic acid metabolism | 3 | 14 | 0.037307789 | E514,E701,E358 | C00051,C00217/C05938/C00025,C00362/C00020 | Glutathione; 5-L-Glutamyl-L-cysteinylglycine; N-(N-gamma-L-Glutamyl-L-cysteinyl)glycine; gamma-L-Glutamyl-L-cysteinyl-glycine; GSH; Reduced glutathione$D-Glutamate; D-Glutamic acid; D-Glutaminic acid; D-2-Aminoglutaric acid/L-4-Hydroxyglutamate semialdehyde/L-Glutamate; L-Glutamic acid; L-Glutamir |

SD 4

**Kidney**

| pathway | overlap size | pathway size | p-value | overlap Empirical Compounds | overlap features | overlap features name |
|---|---|---|---|---|---|---|
| Beta-Alanine metabolism | - | - | - | - | - | - |
| Glutathione Metabolism | - | - | - | - | - | - |
| Vitamin A (retinol) metabolism | 5 | 7 | 0.046130577 | E761,E228,E432,E170,E535 | C00362/C00020,C06428/C02075/CE5932/CE5013,C14768/C04742/C14749/C14748/C14769/CE5016/C14778/C14770/C14771/C04805,CE2962,CE5653 | dGMP; 2'-Deoxyguanosine 5'-monophosphate; 2'-Deoxyguanosine 5'-phosphate; Deoxyguanylic acid; Deoxyguanosine monophosphate/AMP; Adenosine 5'-monophosphate; Adenylic acid; Adenylate; 5'-AMP; 5'-Adenylic acid; 5'-Adenosine monophosphate; Adenosine 5'-phosphate$(5Z,8Z,11Z,14Z,17Z)-Icosape |
| Glycerophospholipid metabolism | 11 | 19 | 0.0297454 | E420,E62,E296,E55,E452,E432,E653,E449,E599,E496,E562 | C06427/C06426,C00577,C00184/C00256/C00186/C01013,C15647,C00795/C00221/C00267/C00031/C00137/C00738/C00095/C00962/C00247/C00984/C00936/C01582/C00159/C02336/C00293/C00124,C14768/C04742/C14749/C14748/C14769/CE5016/C14778/C14770/C14771/C04805,C01595,C027 | (9Z,12Z,15Z)-Octadecatrienoic acid; alpha-Linolenic acid; 9,12,15-Octadecatrienoic acid; Linolenate; alpha-Linolenate/(6Z,9Z,12Z)-Octadecatrienoic acid; 6,9,12-Octadecatrienoic acid; gamma-Linolenic acid; Gamolenic acid$D-Glyceraldehyde$Glycerone; Dihydroxyacetone; 1,3-Dihydroxyacetone; 1,3-Dihydroxy-2- |
| Glycine, serine, alanine and threonine metabolism | - | - | - | - | - | - |
| Vitamin D3 (cholecalciferol) metabolism | 4 | 4 | 0.012099824 | E153,E79,E216,E41 | CE2205,C17335/CE2202,CE2204,C17337/CE2206/CE2203 | 1alpha,24R,25-trihydroxyvitamin D3$/23S,25,26-trihydroxyvitamin D3$25-hydroxyvitamin D3-26,23-lactone$/24-oxo-1alpha,25-dihydroxyvitamin D3/25-hydroxyvitamin D3-26,23-lactol |
| Histidine metabolism | - | - | - | - | - | - |
| Tryptophan metabolism | 3 | 4 | 0.0148727 | E287,E141,E201 | C05660,C02470,C05645 | 5-Methoxyindoleacetate$Xanthurenic acid; Xanthurenate$4-(2-Amino-3-hydroxyphenyl)-2,4-dioxobutanoate |
| Tyrosine metabolism | 6 | 11 | 0.015796992 | E56,E215,E254,E168,E188,E375 | C05838/C00166/C02763/C00811,CE4888/C05578,C05583/C05582,C05589/C00788,C01183/C02735/C00483,C00079/CE2172 | cis-2-Hydroxycinnamate; 2-Coumarinate/Phenylpyruvate; Phenylpyruvic acid; alpha-Ketohydrocinnamic acid; keto-Phenylpyruvate; 3-Phenyl-2-oxopropanoate/enol-Phenylpyruvate; enol-Phenylpyruvic acid; enol-alpha-Ketohydrocinnamic acid; 2-Hydroxy-3-phenylpropenoate/4-Coumarate; p-Coumaric acid; tra |
| Vitamin B9 (folate) metabolism | - | - | - | - | - | - |
| Linoleate metabolism | 6 | 8 | 0.019830266 | E420,E283,E703,E653,E760,E556 | C06427/C06426,C14762/C14825/C14826,C14765/CE5526/CE5527,C01595,C14762/C14825/C14826,C14762/C14825/C14826 | (9Z,12Z,15Z)-Octadecatrienoic acid; alpha-Linolenic acid; 9,12,15-Octadecatrienoic acid; Linolenate; alpha-Linolenate/(6Z,9Z,12Z)-Octadecatrienoic acid; 6,9,12-Octadecatrienoic acid; gamma-Linolenic acid; Gamolenic acid$13(S)-HODE; (13S)-Hydroxyoctadecadienoic acid; (9Z,11E)-(13S)-13-Hydroxyoctadeca-9,1 |
| Methionine and cysteine metabolism | - | - | - | - | - | - |
| Glutamate metabolism | - | - | - | - | - | - |
| Omega-3 fatty acid metabolism | 3 | 3 | 0.031509957 | E70,E761,E420 | C16300,C00362/C00020,C06427/C06426 | Stearidonic acid; 6,9,12,15-Octadecatetraenoic acid; (6Z,9Z,12Z,15Z)-Octadecatetraenoic acid$dGMP; 2'-Deoxyguanosine 5'-monophosphate; 2'-Deoxyguanosine 5'-phosphate; Deoxyguanylic acid; Deoxyguanosine monophosphate/AMP; Adenosine 5'-monophosphate; Adenylic acid; Adenylate; 5'-AMP; 5'-Aden |
| Sialic acid metabolism | - | - | - | - | - | - |
| Hexose phosphorylation | - | - | - | - | - | - |
| Ubiquinone Biosynthesis | - | - | - | - | - | - |
| Purine metabolism | 3 | 5 | 0.046718763 | E322,E74,E117 | C00262,C00312/C00310/C00259/C00181/C00309/C00121,C00294 | Hypoxanthine; Purine-6-ol$L-Xylulose; L-threo-Pentulose; L-Lyxulose/D-Xylulose; D-threo-Pentulose; D-Lyxulose/L-Arabinose; L-Arabinopyranose/D-Xylose; Wood sugar/D-Ribulose; D-erythro-2-Pentulose; D-Arabinoketose; D-Arabinulose; D-Riboketose/D-Ribose$Inosine |

SD 9

Heart

| pathway | overlap size | pathway size | p-value | overlap Empirical Compounds | overlap features | overlap features name |
|---|---|---|---|---|---|---|
| Limonene and pinene degradation | - | - | - | - | - | - |
| Pentose and Glucuronate Interconversions | 1 | 1 | 0.0018486 | E127 | C01101/C03736/C00117/C00442/C00620/C00231/C00199 | L-Ribulose 5-phosphate/alpha-D-Ribose 5-phosphate/D-Ribose 5-phosphate; Ribose 5-phosphate/alpha-D-Ribose 1-phosphate/alpha-D-Ribose 1-phosphate; Ribose 1-phosphate; D-Ribose 1-phosphate/D-Xylulose 5-phosphate/D-Ribulose 5-phosphate |
| Carbon fixation | 1 | 2 | 0.0037812 | E127 | C01101/C03736/C00117/C00442/C00620/C00231/C00199 | L-Ribulose 5-phosphate/alpha-D-Ribose 5-phosphate/D-Ribose 5-phosphate; Ribose 5-phosphate/alpha-D-Ribose 1-phosphate/alpha-D-Ribose 1-phosphate; Ribose 1-phosphate; D-Ribose 1-phosphate/D-Xylulose 5-phosphate/D-Ribulose 5-phosphate |
| Glycosylphosphatidylinositol(GPI)-anchor biosynthesis | - | - | - | - | - | - |
| Selenoamino acid metabolism | 1 | 4 | 0.0106714 | E865 | C05699 | Selenocystathionine |
| Vitamin B3 (nicotinate and nicotinamide) metabolism | 1 | 4 | 0.0106714 | E127 | C01101/C03736/C00117/C00442/C00620/C00231/C00199 | L-Ribulose 5-phosphate/alpha-D-Ribose 5-phosphate/D-Ribose 5-phosphate; Ribose 5-phosphate/alpha-D-Ribose 1-phosphate/alpha-D-Ribose 1-phosphate; Ribose 1-phosphate; D-Ribose 1-phosphate/D-Xylulose 5-phosphate/D-Ribulose 5-phosphate |
| Pentose phosphate pathway | 1 | 5 | 0.0146206 | E127 | C01101/C03736/C00117/C00442/C00620/C00231/C00199 | L-Ribulose 5-phosphate/alpha-D-Ribose 5-phosphate/D-Ribose 5-phosphate; Ribose 5-phosphate/alpha-D-Ribose 1-phosphate/alpha-D-Ribose 1-phosphate; Ribose 1-phosphate; D-Ribose 1-phosphate/D-Xylulose 5-phosphate/D-Ribulose 5-phosphate |
| Saturated fatty acids beta-oxidation | 1 | 5 | 0.0146206 | E953 | C00249 | Hexadecanoic acid; Hexadecanoate; Hexadecylic acid; Palmitic acid; Palmitate; Cetylic acid |
| Xenobiotics metabolism | 1 | 5 | 0.0146206 | E953 | C00249 | Hexadecanoic acid; Hexadecanoate; Hexadecylic acid; Palmitic acid; Palmitate; Cetylic acid |
| Phosphatidylinositol phosphate metabolism | 1 | 8 | 0.0241156 | E953 | C00249 | Hexadecanoic acid; Hexadecanoate; Hexadecylic acid; Palmitic acid; Palmitate; Cetylic acid |
| Fatty Acid Metabolism | 1 | 8 | 0.0241156 | E953 | C00249 | Hexadecanoic acid; Hexadecanoate; Hexadecylic acid; Palmitic acid; Palmitate; Cetylic acid |
| Pyrimidine metabolism | 1 | 9 | 0.0268885 | E127 | C01101/C03736/C00117/C00442/C00620/C00231/C00199 | L-Ribulose 5-phosphate/alpha-D-Ribose 5-phosphate/D-Ribose 5-phosphate; Ribose 5-phosphate/alpha-D-Ribose 1-phosphate/alpha-D-Ribose 1-phosphate; Ribose 1-phosphate; D-Ribose 1-phosphate/D-Xylulose 5-phosphate/D-Ribulose 5-phosphate |
| Purine metabolism | 1 | 9 | 0.0268885 | E127 | C01101/C03736/C00117/C00442/C00620/C00231/C00199 | L-Ribulose 5-phosphate/alpha-D-Ribose 5-phosphate/D-Ribose 5-phosphate; Ribose 5-phosphate/alpha-D-Ribose 1-phosphate/alpha-D-Ribose 1-phosphate; Ribose 1-phosphate; D-Ribose 1-phosphate/D-Xylulose 5-phosphate/D-Ribulose 5-phosphate |
| Glycerophospholipid metabolism | - | - | - | - | - | - |
| Fructose and mannose metabolism | - | - | - | - | - | - |
| Nucleotide Sugar Metabolism | - | - | - | - | - | - |
| Fatty acid activation | 1 | 15 | 0.0387362 | E953 | C00249 | Hexadecanoic acid; Hexadecanoate; Hexadecylic acid; Palmitic acid; Palmitate; Cetylic acid |
| Glycosphingolipid metabolism | 1 | 16 | 0.0406689 | E953 | C00249 | Hexadecanoic acid; Hexadecanoate; Hexadecylic acid; Palmitic acid; Palmitate; Cetylic acid |
| De novo fatty acid biosynthesis | 1 | 23 | 0.0482312 | E953 | C00249 | Hexadecanoic acid; Hexadecanoate; Hexadecylic acid; Palmitic acid; Palmitate; Cetylic acid |
| Sialic acid metabolism | - | - | - | - | - | - |

SD 9

Spleen

| pathway | overlap size | pathway size | p-value | overlap Empirical Compounds | overlap features | overlap features name |
|---|---|---|---|---|---|---|
| Aspartate and asparagine metabolism | 2 | 7 | 0.0297454 | E196,E98 | C01419,C00169 | Cys-Gly; L-Cysteinylglycine$Carbamoyl phosphate |
| Valine, leucine and isoleucine degradation | 2 | 7 | 0.0420133 | E339,E257 | C00042/C02170,C00164/C06002/C00109/C00349/C00232 | Succinate; Succinic acid; Butanedionic acid; Ethylenesuccinic acid/Methylmalonate; Methylmalonic acid$Acetoacetate; 3-Oxobutanoic acid; beta-Ketobutyric acid; Acetoacetic acid/(S)-Methylmalonate semialdehyde/2-Oxobutanoate; 2-Ketobutyric acid; 2-Oxobutyric acid; 2-Oxobutyrate; 2-Oxobutanoic acid; alpha-Ketobutyric acid; alpha-Ketobutyrate/2-Methyl-3-oxopropanoate; 3-Oxo-2-methylpropanoate; Methylmalonate semialdehyde/Succinate semialdehyde; Succinic semialdehyde; 4-Oxobutanoate |
| Glycosphingolipid metabolism | 3 | 6 | 0.0015125 | E97,E70,E352 | C12144,C00319/C02934,C00319/C02934 | Phytosphingosine; 4-D-Hydroxysphinganine$Sphingosine; Sphingenine; Sphingoid; Sphing-4-enine/3-Dehydrosphinganine; 3-Dehydro-D-sphinganine$Sphingosine; Sphingenine; Sphingoid; Sphing-4-enine/3-Dehydrosphinganine; 3-Dehydro-D-sphinganine |
| Histidine metabolism | - | - | - | - | - | - |
| Pyrimidine metabolism | - | - | - | - | - | - |
| Nitrogen metabolism | - | - | - | - | - | - |
| Beta-Alanine metabolism | - | - | - | - | - | - |
| Glutathione Metabolism | - | - | - | - | - | - |
| Vitamin B3 (nicotinate and nicotinamide) metabolism | - | - | - | - | - | - |
| Squalene and cholesterol biosynthesis | 2 | 2 | 0.0045374 | E644,E257 | C01143,C00164/C06002/C00109/C00349/C00232 | (R)-5-Diphosphomevalonate$Acetoacetate; 3-Oxobutanoic acid; beta-Ketobutyric acid; Acetoacetic acid/(S)-Methylmalonate semialdehyde/2-Oxobutanoate; 2-Ketobutyric acid; 2-Oxobutyric acid; 2-Oxobutyrate; 2-Oxobutanoic acid; alpha-Ketobutyric acid; alpha-Ketobutyrate/2-Methyl-3-oxopropanoate; 3-Oxo-2-methylpropanoate; Methylmalonate semialdehyde/Succinate semialdehyde; Succinic semialdehyde; 4-Oxobutanoate |
| Methionine and cysteine metabolism | - | - | - | - | - | - |
| Purine metabolism | - | - | - | - | - | - |
| Glutamate metabolism | 2 | 7 | 0.0420133 | E339,E257 | C00042/C02170,C00164/C06002/C00109/C00349/C00232 | Succinate; Succinic acid; Butanedionic acid; Ethylenesuccinic acid/Methylmalonate; Methylmalonic acid$Acetoacetate; 3-Oxobutanoic acid; beta-Ketobutyric acid; Acetoacetic acid/(S)-Methylmalonate semialdehyde/2-Oxobutanoate; 2-Ketobutyric acid; 2-Oxobutyric acid; 2-Oxobutyrate; 2-Oxobutanoic acid; alpha-Ketobutyric acid; alpha-Ketobutyrate/2-Methyl-3-oxopropanoate; 3-Oxo-2-methylpropanoate; Methylmalonate semialdehyde/Succinate semialdehyde; Succinic semialdehyde; 4-Oxobutanoate |
| Alanine and Aspartate Metabolism | - | - | - | - | - | - |
| Butanoate metabolism | 3 | 7 | 0.0073943 | E339,E257,E189 | C00042/C02170,C00164/C06002/C00109/C00349/C00232,C06144 | Succinate; Succinic acid; Butanedionic acid; Ethylenesuccinic acid/Methylmalonate; Methylmalonic acid$Acetoacetate; 3-Oxobutanoic acid; beta-Ketobutyric acid; Acetoacetic acid/(S)-Methylmalonate semialdehyde/2-Oxobutanoate; 2-Ketobutyric acid; 2-Oxobutyric acid; 2-Oxobutyrate; 2-Oxobutanoic acid; alpha-Ketobutyric acid; alpha-Ketobutyrate/2-Methyl-3-oxopropanoate; 3-Oxo-2-methylpropanoate; Methylmalonate semialdehyde/Succinate semialdehyde; Succinic semialdehyde; 4-Oxobutanoate$3-Butynoate |
| Glycine, serine, alanine and threonine metabolism | - | - | - | - | - | - |
| Arginine and Proline Metabolism | - | - | - | - | - | - |
| Tryptophan metabolism | - | - | - | - | - | - |
| Putative anti-Inflammatory metabolites formation from EPA | - | - | - | - | - | - |
| Urea cycle/amino group metabolism | - | - | - | - | - | - |
| Lysine metabolism | - | - | - | - | - | - |
| Bile acid biosynthesis | - | - | - | - | - | - |
| Leukotriene metabolism | - | - | - | - | - | - |
| Aminosugars metabolism | - | - | - | - | - | - |
| Lipoate metabolism | 1 | 1 | 0.034787 | E632 | CE2102 | lipoyllysine |
| N-Glycan Degradation | 1 | 1 | 0.034787 | E510 | C00795/C00221/C00095/C00267/C00031/C00137/C00738/C00962/C00247/C00984/C00936/C01582/C00159/C02336/C00293/C00124 | D-Tagatose; lyxo-Hexulose/beta-D-Glucose/D-Fructose; Levulose; Fruit sugar; D-arabino-Hexulose/alpha-D-Glucose/D-Glucose; Grape sugar; Dextrose/myo-Inositol; D-myo-Inositol; 1D-myo-Inositol; L-myo-Inositol; 1L-myo-Inositol; meso-Inositol; Inositol; Dambose; Cyclohexitol; Meat sugar; Bios I/D-Hexose/beta-D-Galactose/L-Sorbose; L-xylo-Hexulose/alpha-D-Galactose/(Galactose/D-Mannose; Mannose; Seminose; Carubinose/beta-D-Fructose; beta-Fruit sugar; beta-D-arabino-Hexulose; beta-Levulose; Fructose/Glucose/D-Galactose |
| C21-steroid hormone biosynthesis and metabolism | - | - | - | - | - | - |
| Vitamin B2 (riboflavin) metabolism | - | - | - | - | - | - |
| Alkaloid biosynthesis II | - | - | - | - | - | - |
| Vitamin B1 (thiamin) metabolism | - | - | - | - | - | - |
| Vitamin E metabolism | - | - | - | - | - | - |
| Mono-unsaturated fatty acid beta-oxidation | - | - | - | - | - | - |
| Ubiquinone Biosynthesis | - | - | - | - | - | - |
| Arachidonic acid metabolism | - | - | - | - | - | - |

SD 9

Kidney

| pathway | overlap size | pathway size | p-value | overlap Empirical Compounds | overlap features | overlap features name |
|---|---|---|---|---|---|---|
| Beta-Alanine metabolism | 5 | 6 | 6.72E-04 | E223,E673,E392,E228,E249 | C00222/C00022,C00135,C00256/C00186/C00184/C01013,C05938/C00217/C00025,C05145/C01026/C02356/C00334/C03284/C01205 | 3-Oxopropanoate; Malonate semialdehyde/Pyruvate; Pyruvic acid; 2-Oxopropanoate; 2-Oxopropanoic acid; Pyroracemic acid$L-Histidine; (S)-alpha-Amino-1H-imidazole-4-propionic acid$(R)-Lactate; D-Lactate; D-Lactic acid; D-2-Hydroxypropanoic acid; D-2-Hydroxypropionic acid/(S)-Lactate; L-Lactate; L-Lactic acid/Glycerone; Dihydroxyacetone; 1,3-Dihydroxyacetone; 1,3-Dihydroxy-2-propanone; 1,3-Dihydroxypropan-2-one/3-Hydroxypropanoate; 3-Hydroxypropanoic acid; 3-Hydroxypropionate; 3-Hydroxypropionic acid; Hydracrylic acid$L-4-Hydroxyglutamate semialdehyde/D-Glutamate; D-Glutamic acid; D-Glutaminic acid; D-2-Aminoglutaric acid/L-Glutamate; L-Glutamic acid; L-Glutaminic acid; Glutamate$3-Aminoisobutyric acid; 3-Aminoisobutanoate; 3-Amino-2-methylpropanoate/N,N-Dimethylglycine; Dimethylglycine/(S)-2-Aminobutanoate; (S)-2-Aminobutanoic acid; (S)-2-Aminobutyric acid/4-Aminobutanoate; 4-Aminobutanoic acid; 4-Aminobutyrate; 4-Aminobutyric acid; gamma-Aminobutyric acid/L-3-Amino-isobutanoate; (S)-3-Amino-isobutyrate; L-3-Ami |
| Glutathione Metabolism | 3 | 3 | 0.0038652 | E364,E620,E228 | C00097,C04281/C04282/C01879,C05938/C00217/C00025 | L-Cysteine; L-2-Amino-3-mercaptopropionic acid$L-1-Pyrroline-3-hydroxy-5-carboxylate; 3-Hydroxy-L-1-pyrroline-5-carboxylate; (3R,5S)-1-Pyrroline-3-hydroxy-5-carboxylate/1-Pyrroline-4-hydroxy-2-carboxylate/5-Oxoproline; Pyroglutamic acid; 5-Pyrrolidone-2-carboxylic acid; Pyroglutamate; 5-Oxo-L-proline; L-Pyroglutamic acid; L-5-Pyrrolidone-2-carboxylic acid$L-4-Hydroxyglutamate semialdehyde/D-Glutamate; D-Glutamic acid; D-Glutaminic acid; D-2-Aminoglutaric acid/L-Glutamate; L-Glutamic acid; L-Glutaminic acid; Glutamate |
| Vitamin A (retinol) metabolism | 3 | 9 | 0.0069742 | E44,E177,E159 | C06428/C02075/CE5932,CE5013,C14768/C04742/C14749/C14748/C14769/CE5016/C14778/C14770/C14771/C04805,CE5757 | (5Z,8Z,11Z,14Z,17Z)-Icosapentaenoic acid; (5Z,8Z,11Z,14Z,17Z)-Eicosapentaenoic acid; Eicosapentaenoic acid; 5,8,11,14,17-Icosapentaenoic acid; (5Z,8Z,11Z,14Z,17Z)-Eicosapentaenoate/Retinyl ester; all-trans-Retinyl ester/13,14-epoxy-retinol/$5,6-EET; (8Z,11Z,14Z)-5,6-Epoxyeicosa-8,11,14-trienoic acid; (8Z,11Z,14Z)-5,6-Epoxyicosa-8,11,14-trienoic acid/(15S)-15-Hydroxy-5,8,11-cis-13-trans-eicosatetraenoate; (15S)-15-Hydroxy-5,8,11-cis-13-trans-icosatetraenoate; (5Z,8Z,11Z,13E)-(15S)-15-Hydroxyicosa-5,8,11,13-tetraenoic acid; 15(S)-HETE/19(S)-HETE; (19S)-Hydroxyeicosatetraenoic acid; (19S)-Hydroxyicosatetraenoic acid; (19S)-Hydroxy arachidonic acid/20-HETE; (5Z,8Z,11Z,14Z)-20-Hydroxyicosa-5,8,11,14-tetraenoic acid; 20-Hydroxyeicosatetraenoic acid; 20-Hydroxyicosatetraenoic acid; 20-Hydroxy arachidonic acid/8,9-EET; (5Z,11Z,14Z)-8,9-Epoxyeicosa-5,11,14-trienoic acid; (5Z,11Z,14Z)-8,9-Epoxyicosa-5,11,14-trienoic acid/13,14-dihydroxy-retinol/16(R)-HETE; (5Z,8Z,11Z,14Z)-(16R)-16-Hydroxyeicosa-5,8,11,14-tetraenoic aci |
| Glycerophospholipid metabolism | 3 | 10 | 0.0091589 | E121,E177,E192 | C01595,C14768/C04742/C14749/C14748/C14769/CE5016/C14778/C14770/C14771/C04805,C00065/C00740 | Linoleate; Linoleic acid; (9Z,12Z)-Octadecadienoic acid; 9-cis,12-cis-Octadecadienoate; 9-cis,12-cis-Octadecadienoic acid$5,6-EET; (8Z,11Z,14Z)-5,6-Epoxyeicosa-8,11,14-trienoic acid; (8Z,11Z,14Z)-5,6-Epoxyicosa-8,11,14-trienoic acid/(15S)-15-Hydroxy-5,8,11-cis-13-trans-eicosatetraenoate; (15S)-15-Hydroxy-5,8,11-cis-13-trans-icosatetraenoate; (5Z,8Z,11Z,13E)-(15S)-15-Hydroxyicosa-5,8,11,13-tetraenoic acid; 15(S)-HETE/19(S)-HETE; (19S)-Hydroxyeicosatetraenoic acid; (19S)-Hydroxyicosatetraenoic acid; (19S)-Hydroxy arachidonic acid/20-HETE; (5Z,8Z,11Z,14Z)-20-Hydroxyicosa-5,8,11,14-tetraenoic acid; 20-Hydroxyeicosatetraenoic acid; 20-Hydroxyicosatetraenoic acid; 20-Hydroxy arachidonic acid/8,9-EET; (5Z,11Z,14Z)-8,9-Epoxyeicosa-5,11,14-trienoic acid; (5Z,11Z,14Z)-8,9-Epoxyicosa-5,11,14-trienoic acid/13,14-dihydroxy-retinol/16(R)-HETE; (5Z,8Z,11Z,14Z)-(16R)-16-Hydroxyeicosa-5,8,11,14-tetraenoic acid; (5Z,8Z,11Z,14Z)-(16R)-16-Hydroxyicosa-5,8,11,14-tetraenoic acid/11,12-EET; (5Z,8Z,14Z)-11,12-Epoxyeicosa-5,8,14-trie |
| Glycine, serine, alanine and threonine metabolism | 6 | 13 | 0.0114276 | E223,E249,E657,E392,E269,E228 | C00222/C00022,C05145/C01026/C02356/C00334/C03284/C01205,C05519/C00188,C00256/C00186/C00184/C01013,C00258,C05938/C00217/C00025 | 3-Oxopropanoate; Malonate semialdehyde/Pyruvate; Pyruvic acid; 2-Oxopropanoate; 2-Oxopropanoic acid; Pyroracemic acid$3-Aminoisobutyric acid; 3-Aminoisobutanoate; 3-Amino-2-methylpropanoate/N,N-Dimethylglycine; Dimethylglycine/(S)-2-Aminobutanoate; (S)-2-Aminobutanoic acid; (S)-2-Aminobutyric acid/4-Aminobutanoate; 4-Aminobutanoic acid; 4-Aminobutyrate; 4-Aminobutyric acid; gamma-Aminobutyric acid/L-3-Amino-isobutanoate; (S)-3-Amino-isobutyrate; L-3-Amino-isobutyrate; (S)-3-Amino-isobutanoate; (S)-3-Amino-2-methylpropanoate/(R)-3-Amino-2-methylpropanoate; D-3-Amino-isobutanoate$L-Allothreonine; L-allo-Threonine/L-Threonine; 2-Amino-3-hydroxybutyric acid$(R)-Lactate; D-Lactate; D-Lactic acid; D-2-Hydroxypropanoic acid; D-2-Hydroxypropionic acid/(S)-Lactate; L-Lactate; L-Lactic acid/Glycerone; Dihydroxyacetone; 1,3-Dihydroxyacetone; 1,3-Dihydroxy-2-propanone; 1,3-Dihydroxypropan-2-one/3-Hydroxypropanoate; 3-Hydroxypropanoic acid; 3-Hydroxypropionate; 3-Hydroxypropionic acid; Hydracrylic acid$D-Glycerate; Glycer |
| Vitamin D3 (cholecalciferol) metabolism | - | - | - | - | - | - |
| Histidine metabolism | 3 | 4 | 0.0131081 | E223,E673,E228 | C00222/C00022,C00135,C05938/C00217/C00025 | 3-Oxopropanoate; Malonate semialdehyde/Pyruvate; Pyruvic acid; 2-Oxopropanoate; 2-Oxopropanoic acid; Pyroracemic acid$L-Histidine; (S)-alpha-Amino-1H-imidazole-4-propionic acid$L-4-Hydroxyglutamate semialdehyde/D-Glutamate; D-Glutamic acid; D-Glutaminic acid; D-2-Aminoglutaric acid/L-Glutamate; L-Glutamic acid; L-Glutaminic acid; Glutamate |
| Tryptophan metabolism | - | - | - | - | - | - |
| Tyrosine metabolism | - | - | - | - | - | - |
| Vitamin B9 (folate) metabolism | 1 | 1 | 0.0185699 | E192 | C00065/C00740 | L-Serine; L-2-Amino-3-hydroxypropionic acid; L-3-Hydroxy-alanine; Serine/D-Serine |
| Linoleate metabolism | - | - | - | - | - | - |
| Methionine and cysteine metabolism | 5 | 11 | 0.0210066 | E190,E364,E228,E548,E223 | C00302,C00097,C05938/C00217/C00025,C05844,C00222/C00022 | DL-Glutamate; DL-Glutaminic acid; 2-Aminoglutaric acid; Glutamate$L-Cysteine; L-2-Amino-3-mercaptopropionic acid$L-4-Hydroxyglutamate semialdehyde/D-Glutamate; D-Glutamic acid; D-Glutaminic acid; D-2-Aminoglutaric acid/L-Glutamate; L-Glutamic acid; L-Glutaminic acid; Glutamate$5-L-Glutamyl-taurine; 5-Glutamyl-taurine$3-Oxopropanoate; Malonate semialdehyde/Pyruvate; Pyruvic acid; 2-Oxopropanoate; 2-Oxopropanoic acid; Pyroracemic acid |
| Glutamate metabolism | 3 | 5 | 0.0276447 | E223,E228,E249 | C00222/C00022,C05938/C00217/C00025,C05145/C01026/C02356/C00334/C03284/C01205 | 3-Oxopropanoate; Malonate semialdehyde/Pyruvate; Pyruvic acid; 2-Oxopropanoate; 2-Oxopropanoic acid; Pyroracemic acid$L-4-Hydroxyglutamate semialdehyde/D-Glutamate; D-Glutamic acid; D-Glutaminic acid; D-2-Aminoglutaric acid/L-Glutamate; L-Glutamic acid; L-Glutaminic acid; Glutamate$3-Aminoisobutyric acid; 3-Aminoisobutanoate; 3-Amino-2-methylpropanoate/N,N-Dimethylglycine; Dimethylglycine/(S)-2-Aminobutanoate; (S)-2-Aminobutanoic acid; (S)-2-Aminobutyric acid/4-Aminobutanoate; 4-Aminobutanoic acid; 4-Aminobutyrate; 4-Aminobutyric acid; gamma-Aminobutyric acid/L-3-Amino-isobutanoate; (S)-3-Amino-isobutyrate; L-3-Amino-isobutyrate; (S)-3-Amino-isobutanoate; (S)-3-Amino-2-methylpropanoate/(R)-3-Amino-2-methylpropanoate; D-3-Amino-isobutanoate |
| Omega-3 fatty acid metabolism | - | - | - | - | - | - |
| Sialic acid metabolism | 1 | 2 | 0.0421813 | E192 | C00065/C00740 | L-Serine; L-2-Amino-3-hydroxypropionic acid; L-3-Hydroxy-alanine; Serine/D-Serine |
| Hexose phosphorylation | 1 | 2 | 0.0421813 | E25 | C05838/C00166/C02763/C00811 | cis-2-Hydroxycinnamate; 2-Coumarinate/Phenylpyruvate; Phenylpyruvic acid; alpha-Ketohydrocinnamic acid; keto-Phenylpyruvate; 3-Phenyl-2-oxopropanoate/enol-Phenylpyruvate; enol-Phenylpyruvic acid; enol-alpha-Ketohydrocinnamic acid; 2-Hydroxy-3-phenylpropenoate/4-Coumarate; p-Coumaric acid; trans-4-Hydroxycinnamate; trans-p-Hydroxycinnamate; 4-Hydroxycinnamic acid; 4-Hydroxycinnamate |
| Ubiquinone Biosynthesis | 1 | 2 | 0.0421813 | E25 | C05838/C00166/C02763/C00811 | cis-2-Hydroxycinnamate; 2-Coumarinate/Phenylpyruvate; Phenylpyruvic acid; alpha-Ketohydrocinnamic acid; keto-Phenylpyruvate; 3-Phenyl-2-oxopropanoate/enol-Phenylpyruvate; enol-Phenylpyruvic acid; enol-alpha-Ketohydrocinnamic acid; 2-Hydroxy-3-phenylpropenoate/4-Coumarate; p-Coumaric acid; trans-4-Hydroxycinnamate; trans-p-Hydroxycinnamate; 4-Hydroxycinnamic acid; 4-Hydroxycinnamate |
| Purine metabolism | - | - | - | - | - | - |

SD 4

Heart

| pathway | overlap size | pathway size | p-value | overlap Empirical Compounds | overlap features | overlap features name |
|---|---|---|---|---|---|---|
| Fatty acid activation | - | - | - | - | - | - |
| De novo fatty acid biosynthesis | - | - | - | - | - | - |
| Xenobiotics metabolism | - | - | - | - | - | - |
| Fructose and mannose metabolism | 1 | 1 | 0.005797832 | E579 | C02965/C05345/C00092/C00085/C00103/C02976/C01171/C01172/C01177/C03735/C00636/C03862/C04006/C01097/C01094/C00668/C03546/C00446/C00275/CE3075 | D-Hexose 6-phosphate/beta-D-Fructose 6-phosphate/D-Glucose 6-phosphate; Glucose 6-phosphate; Robison ester/D-Fructose 6-phosphate; D-Fructose 6-phosphoric acid; Neuberg ester/D-Glucose 1-phosphate; alpha-D-Glucose 1-phosphate; Cori ester; D-Glucose alpha-1-phosphate/Fructose 1-phosphate/alph |
| Nucleotide Sugar Metabolism | 1 | 1 | 0.005797832 | E579 | C02965/C05345/C00092/C00085/C00103/C02976/C01171/C01172/C01177/C03735/C00636/C03862/C04006/C01097/C01094/C00668/C03546/C00446/C00275/CE3075 | D-Hexose 6-phosphate/beta-D-Fructose 6-phosphate/D-Glucose 6-phosphate; Glucose 6-phosphate; Robison ester/D-Fructose 6-phosphate; D-Fructose 6-phosphoric acid; Neuberg ester/D-Glucose 1-phosphate; alpha-D-Glucose 1-phosphate; Cori ester; D-Glucose alpha-1-phosphate/Fructose 1-phosphate/alph |
| Linoleate metabolism | - | - | - | - | - | - |
| Fatty Acid Metabolism | - | - | - | - | - | - |
| Polyunsaturated fatty acid biosynthesis | 1 | 2 | 0.011259558 | E493 | C01530 | Octadecanoic acid; Stearate; Stearic acid |
| Carbon fixation | 1 | 2 | 0.011259558 | E579 | C02965/C05345/C00092/C00085/C00103/C02976/C01171/C01172/C01177/C03735/C00636/C03862/C04006/C01097/C01094/C00668/C03546/C00446/C00275/CE3075 | D-Hexose 6-phosphate/beta-D-Fructose 6-phosphate/D-Glucose 6-phosphate; Glucose 6-phosphate; Robison ester/D-Fructose 6-phosphate; D-Fructose 6-phosphoric acid; Neuberg ester/D-Glucose 1-phosphate; alpha-D-Glucose 1-phosphate; Cori ester; D-Glucose alpha-1-phosphate/Fructose 1-phosphate/alph |
| Glycolysis and Gluconeogenesis | 1 | 2 | 0.011259558 | E579 | C02965/C05345/C00092/C00085/C00103/C02976/C01171/C01172/C01177/C03735/C00636/C03862/C04006/C01097/C01094/C00668/C03546/C00446/C00275/CE3075 | D-Hexose 6-phosphate/beta-D-Fructose 6-phosphate/D-Glucose 6-phosphate; Glucose 6-phosphate; Robison ester/D-Fructose 6-phosphate; D-Fructose 6-phosphoric acid; Neuberg ester/D-Glucose 1-phosphate; alpha-D-Glucose 1-phosphate; Cori ester; D-Glucose alpha-1-phosphate/Fructose 1-phosphate/alph |
| Hexose phosphorylation | 1 | 2 | 0.011259558 | E579 | C02965/C05345/C00092/C00085/C00103/C02976/C01171/C01172/C01177/C03735/C00636/C03862/C04006/C01097/C01094/C00668/C03546/C00446/C00275/CE3075 | D-Hexose 6-phosphate/beta-D-Fructose 6-phosphate/D-Glucose 6-phosphate; Glucose 6-phosphate; Robison ester/D-Fructose 6-phosphate; D-Fructose 6-phosphoric acid; Neuberg ester/D-Glucose 1-phosphate; alpha-D-Glucose 1-phosphate; Cori ester; D-Glucose alpha-1-phosphate/Fructose 1-phosphate/alph |
| Galactose metabolism | 1 | 2 | 0.011259558 | E579 | C02965/C05345/C00092/C00085/C00103/C02976/C01171/C01172/C01177/C03735/C00636/C03862/C04006/C01097/C01094/C00668/C03546/C00446/C00275/CE3075 | D-Hexose 6-phosphate/beta-D-Fructose 6-phosphate/D-Glucose 6-phosphate; Glucose 6-phosphate; Robison ester/D-Fructose 6-phosphate; D-Fructose 6-phosphoric acid; Neuberg ester/D-Glucose 1-phosphate; alpha-D-Glucose 1-phosphate; Cori ester; D-Glucose alpha-1-phosphate/Fructose 1-phosphate/alph |
| Arachidonic acid metabolism | - | - | - | - | - | - |
| Omega-3 fatty acid metabolism | - | - | - | - | - | - |
| Pyrimidine metabolism | 2 | 9 | 0.013696328 | E428,E101 | C00819/C00064/C05100,C00906 | D-Glutamine; D-2-Aminoglutaramic acid/L-Glutamine; L-2-Aminoglutaramic acid/3-Ureidoisobutyrate$5,6-Dihydrothymine; Dihydrothymine; 5,6-Dihydro-5-methyluracil |
| Glycosphingolipid metabolism | - | - | - | - | - | - |
| Starch and Sucrose Metabolism | 1 | 3 | 0.020334426 | E579 | C02965/C05345/C00092/C00085/C00103/C02976/C01171/C01172/C01177/C03735/C00636/C03862/C04006/C01097/C01094/C00668/C03546/C00446/C00275/CE3075 | D-Hexose 6-phosphate/beta-D-Fructose 6-phosphate/D-Glucose 6-phosphate; Glucose 6-phosphate; Robison ester/D-Fructose 6-phosphate; D-Fructose 6-phosphoric acid; Neuberg ester/D-Glucose 1-phosphate; alpha-D-Glucose 1-phosphate; Cori ester; D-Glucose alpha-1-phosphate/Fructose 1-phosphate/alph |
| Glycosylphosphatidylinositol(GPI)-anchor biosynthesis | 1 | 3 | 0.020334426 | E579 | C02965/C05345/C00092/C00085/C00103/C02976/C01171/C01172/C01177/C03735/C00636/C03862/C04006/C01097/C01094/C00668/C03546/C00446/C00275/CE3075 | D-Hexose 6-phosphate/beta-D-Fructose 6-phosphate/D-Glucose 6-phosphate; Glucose 6-phosphate; Robison ester/D-Fructose 6-phosphate; D-Fructose 6-phosphoric acid; Neuberg ester/D-Glucose 1-phosphate; alpha-D-Glucose 1-phosphate; Cori ester; D-Glucose alpha-1-phosphate/Fructose 1-phosphate/alph |
| Saturated fatty acids beta-oxidation | - | - | - | - | - | - |
| N-Glycan biosynthesis | 1 | 4 | 0.026384337 | E579 | C02965/C05345/C00092/C00085/C00103/C02976/C01171/C01172/C01177/C03735/C00636/C03862/C04006/C01097/C01094/C00668/C03546/C00446/C00275/CE3075 | D-Hexose 6-phosphate/beta-D-Fructose 6-phosphate/D-Glucose 6-phosphate; Glucose 6-phosphate; Robison ester/D-Fructose 6-phosphate; D-Fructose 6-phosphoric acid; Neuberg ester/D-Glucose 1-phosphate; alpha-D-Glucose 1-phosphate; Cori ester; D-Glucose alpha-1-phosphate/Fructose 1-phosphate/alph |
| Nitrogen metabolism | 1 | 2 | 0.026552391 | E428 | C00819/C00064/C05100 | D-Glutamine; D-2-Aminoglutaramic acid/L-Glutamine; L-2-Aminoglutaramic acid/3-Ureidoisobutyrate |
| Valine, leucine and isoleucine degradation | 1 | 2 | 0.026552391 | E786 | C00431/C00183/C00719 | 5-Aminopentanoate; 5-Aminopentanoic acid; 5-Aminovaleric acid/L-Valine; 2-Amino-3-methylbutyric acid/Betaine; Trimethylaminoacetate; Glycine betaine; N,N,N-Trimethylglycine; Trimethylammonioacetate |
| Fatty acid oxidation, peroxisome | - | - | - | - | - | - |
| Aminosugars metabolism | 1 | 5 | 0.035123099 | E579 | C02965/C05345/C00092/C00085/C00103/C02976/C01171/C01172/C01177/C03735/C00636/C03862/C04006/C01097/C01094/C00668/C03546/C00446/C00275/CE3075 | D-Hexose 6-phosphate/beta-D-Fructose 6-phosphate/D-Glucose 6-phosphate; Glucose 6-phosphate; Robison ester/D-Fructose 6-phosphate; D-Fructose 6-phosphoric acid; Neuberg ester/D-Glucose 1-phosphate; alpha-D-Glucose 1-phosphate; Cori ester; D-Glucose alpha-1-phosphate/Fructose 1-phosphate/alph |
| Pentose phosphate pathway | 1 | 5 | 0.035123099 | E579 | C02965/C05345/C00092/C00085/C00103/C02976/C01171/C01172/C01177/C03735/C00636/C03862/C04006/C01097/C01094/C00668/C03546/C00446/C00275/CE3075 | D-Hexose 6-phosphate/beta-D-Fructose 6-phosphate/D-Glucose 6-phosphate; Glucose 6-phosphate; Robison ester/D-Fructose 6-phosphate; D-Fructose 6-phosphoric acid; Neuberg ester/D-Glucose 1-phosphate; alpha-D-Glucose 1-phosphate; Cori ester; D-Glucose alpha-1-phosphate/Fructose 1-phosphate/alph |
| Arginine and Proline Metabolism | 1 | 3 | 0.041761197 | E428 | C00819/C00064/C05100 | D-Glutamine; D-2-Aminoglutaramic acid/L-Glutamine; L-2-Aminoglutaramic acid/3-Ureidoisobutyrate |
| Butanoate metabolism | 1 | 3 | 0.041761197 | E786 | C00431/C00183/C00719 | 5-Aminopentanoate; 5-Aminopentanoic acid; 5-Aminovaleric acid/L-Valine; 2-Amino-3-methylbutyric acid/Betaine; Trimethylaminoacetate; Glycine betaine; N,N,N-Trimethylglycine; Trimethylammonioacetate |
| Alanine and Aspartate Metabolism | 1 | 3 | 0.041761197 | E428 | C00819/C00064/C05100 | D-Glutamine; D-2-Aminoglutaramic acid/L-Glutamine; L-2-Aminoglutaramic acid/3-Ureidoisobutyrate |

SD 4

Spleen

| pathway | overlap size | pathway size | p-value | overlap Empirical Compounds | overlap features | overlap features name |
|---|---|---|---|---|---|---|
| Glycolysis and Gluconeogenesis | - | - | - | - | - | - |
| Pentose phosphate pathway | - | - | - | - | - | - |
| Glycine, serine, alanine and threonine metabolism | - | - | - | - | - | - |
| Tryptophan metabolism | 4 | 9 | 0.001260398 | E599,E218,E191,E506 | C10164/C00253,C02220,C00217/C05938/C00025,C03824 | /Nicotinate; Nicotinic acid; Niacin; 3-Pyridinecarboxylic acid$2-Aminomuconate; 2-Aminomuconic acid; o-Aminomuconate$D-Glutamate; D-Glutamic acid; D-Glutaminic acid; D-2-Aminoglutaric acid/L-4-Hydroxyglutamate semialdehyde/L-Glutamate; L-Glutamic acid; L-Glutaminic acid; Glutamate$2-Aminomuc |
| Aspartate and asparagine metabolism | 3 | 13 | 0.032518276 | E191,E288,E382 | C00217/C05938/C00025,C05145/C01026/C01205/C02356/C00334/C03284,C03287 | D-Glutamate; D-Glutamic acid; D-Glutaminic acid; D-2-Aminoglutaric acid/L-4-Hydroxyglutamate semialdehyde/L-Glutamate; L-Glutamic acid; L-Glutaminic acid; Glutamate$3-Aminoisobutyric acid; 3-Aminoisobutanoate; 3-Amino-2-methylpropanoate/N,N-Dimethylglycine; Dimethylglycine/(R)-3-Amino-2-meth |
| Fructose and mannose metabolism | - | - | - | - | - | - |
| Prostaglandin formation from arachidonate | 6 | 11 | 0.004453407 | E340,E34,E382,E90,E54,E512 | CE6234/CE5924/CE5929/CE4989/CE5931/CE7093/CE5663/CE5828,C00219,CE5730,C13856,CE5700,C13856 | prostaglandin H1///20-OH-10,11-dihydro-leukotriene B4//13,14-dihydro-15-oxo-PGE1/13,14-dihydro- lipoxin A4/$(5Z,8Z,11Z,14Z)-Icosatetraenoic acid; Arachidonate; Arachidonic acid; cis-5,8,11,14-Eicosatetraenoic acid$$2-Arachidonoylglycerol; 2-Arachidonoyl-sn-glycerol$12-hydroperoxyeicosatetraenoate |
| Vitamin B3 (nicotinate and nicotinamide) metabolism | 3 | 6 | 0.004705487 | E599,E191,E350 | C10164/C00253,C00217/C05938/C00025,C01185 | /Nicotinate; Nicotinic acid; Niacin; 3-Pyridinecarboxylic acid$D-Glutamate; D-Glutamic acid; D-Glutaminic acid; D-2-Aminoglutaric acid/L-4-Hydroxyglutamate semialdehyde/L-Glutamate; L-Glutamic acid; L-Glutaminic acid; Glutamate$Nicotinate D-ribonucleotide; beta-Nicotinate D-ribonucleotide; Nicotinate rib |
| Pyruvate Metabolism | - | - | - | - | - | - |
| Butanoate metabolism | 1 | 2 | 0.019662213 | E491 | C00431/C00183/C00719 | 5-Aminopentanoate; 5-Aminopentanoic acid; 5-Aminovaleric acid/L-Valine; 2-Amino-3-methylbutyric acid/Betaine; Trimethylaminoacetate; Glycine betaine; N,N,N-Trimethylglycine; Trimethylammonioacetate |
| Ascorbate (Vitamin C) and Aldarate Metabolism | - | - | - | - | - | - |
| Galactose metabolism | - | - | - | - | - | - |
| Polyunsaturated fatty acid biosynthesis | 1 | 1 | 0.007730443 | E201 | C01530 | Octadecanoic acid; Stearate; Stearic acid |
| Valine, leucine and isoleucine degradation | 1 | 3 | 0.027728762 | E491 | C00431/C00183/C00719 | 5-Aminopentanoate; 5-Aminopentanoic acid; 5-Aminovaleric acid/L-Valine; 2-Amino-3-methylbutyric acid/Betaine; Trimethylaminoacetate; Glycine betaine; N,N,N-Trimethylglycine; Trimethylammonioacetate |
| Pyrimidine metabolism | 4 | 16 | 0.014284514 | E309,E191,E287,E288 | C00299,C00217/C05938/C00025,C00380,C05145/C01026/C01205/C02356/C00334/C03284 | Uridine$D-Glutamate; D-Glutamic acid; D-Glutaminic acid; D-2-Aminoglutaric acid/L-4-Hydroxyglutamate semialdehyde/L-Glutamate; L-Glutamic acid; L-Glutaminic acid; Glutamate$Cytosine$3-Aminoisobutyric acid; 3-Aminoisobutanoate; 3-Amino-2-methylpropanoate/N,N-Dimethylglycine; Dimethylglycine/(I |
| Lysine metabolism | - | - | - | - | - | - |
| Carbon fixation | - | - | - | - | - | - |
| Purine metabolism | - | - | - | - | - | - |
| Fatty acid activation | 4 | 7 | 0.010839425 | E576,E192,E14,E502 | C00712/C01712,C00249,C01595,tettet6 | (9Z)-Octadecenoic acid; (Z)-Octadec-9-enoic acid; Oleate; Oleic acid/(9E)-Octadecenoic acid; 9-Octadecenoic acid, (E)-; (E)-Oleic acid; D9-trans-Octadecenoic acid; 9-trans-Octadecenoic acid; trans-D9-Octadecenoic acid; trans-9-Octadecenoic acid; trans-Elaidic acid; trans-Oleic acid; Elaidic acid; Acide elaidique; Elai |
| TCA cycle | - | - | - | - | - | - |
| Propanoate metabolism | - | - | - | - | - | - |
| Glycerophospholipid metabolism | - | - | - | - | - | - |
| Urea cycle/amino group metabolism | 3 | 10 | 0.017729603 | E191,E288,E382 | C00217/C05938/C00025,C05145/C01026/C01205/C02356/C00334/C03284,C03287 | D-Glutamate; D-Glutamic acid; D-Glutaminic acid; D-2-Aminoglutaric acid/L-4-Hydroxyglutamate semialdehyde/L-Glutamate; L-Glutamic acid; L-Glutaminic acid; Glutamate$3-Aminoisobutyric acid; 3-Aminoisobutanoate; 3-Amino-2-methylpropanoate/N,N-Dimethylglycine; Dimethylglycine/(R)-3-Amino-2-meth |
| Fatty Acid Metabolism | 3 | 5 | 0.018821948 | E288,E14,E192 | 3ohxccoa,C01595,C00249 | 3-Oxohexacosyl-CoA$Linoleate; Linoleic acid; (9Z,12Z)-Octadecadienoic acid; 9-cis,12-cis-Octadecadienoate; 9-cis,12-cis-Octadecadienoic acid$Hexadecanoic acid; Hexadecanoate; Hexadecylic acid; Palmitic acid; Palmitate; Cetylic acid |
| De novo fatty acid biosynthesis | 5 | 12 | 0.025628098 | E34,E576,E192,E14,E332 | C00219,C00712/C01712,C00249,C01595,C00712 | (5Z,8Z,11Z,14Z)-Icosatetraenoic acid; Arachidonate; Arachidonic acid; cis-5,8,11,14-Eicosatetraenoic acid$(9Z)-Octadecenoic acid; (Z)-Octadec-9-enoic acid; Oleate; Oleic acid/(9E)-Octadecenoic acid; 9-Octadecenoic acid, (E)-; (E)-Oleic acid; D9-trans-Octadecenoic acid; 9-trans-Octadecenoic acid; trans-D9-Octadec |
| Drug metabolism - cytochrome P450 | - | - | - | - | - | - |
| Glutamate metabolism | 2 | 7 | 0.048903453 | E191,E288 | C00217/C05938/C00025,C05145/C01026/C01205/C02356/C00334/C03284 | D-Glutamate; D-Glutamic acid; D-Glutaminic acid; D-2-Aminoglutaric acid/L-4-Hydroxyglutamate semialdehyde/L-Glutamate; L-Glutamic acid; L-Glutaminic acid; Glutamate$3-Aminoisobutyric acid; 3-Aminoisobutanoate; 3-Amino-2-methylpropanoate/N,N-Dimethylglycine; Dimethylglycine/(R)-3-Amino-2-meth |
| Glutathione Metabolism | - | - | - | - | - | - |
| Putative anti-Inflammatory metabolites formation from EPA | - | - | - | - | - | - |
| Histidine metabolism | - | - | - | - | - | - |
| Arginine and Proline Metabolism | - | - | - | - | - | - |
| Beta-Alanine metabolism | 2 | 6 | 0.035291152 | E191,E288 | C00217/C05938/C00025,C05145/C01026/C01205/C02356/C00334/C03284 | D-Glutamate; D-Glutamic acid; D-Glutaminic acid; D-2-Aminoglutaric acid/L-4-Hydroxyglutamate semialdehyde/L-Glutamate; L-Glutamic acid; L-Glutaminic acid; Glutamate$3-Aminoisobutyric acid; 3-Aminoisobutanoate; 3-Amino-2-methylpropanoate/N,N-Dimethylglycine; Dimethylglycine/(R)-3-Amino-2-meth |
| Trihydroxycoprostanoyl-CoA beta-oxidation | - | - | - | - | - | - |
| Glycosphingolipid metabolism | 3 | 6 | 0.033862701 | E192,E515,E250 | C00249,C01120,C12144 | Hexadecanoic acid; Hexadecanoate; Hexadecylic acid; Palmitic acid; Palmitate; Cetylic acid$Sphinganine 1-phosphate; Dihydrosphingosine 1-phosphate$Phytosphingosine; 4-D-Hydroxysphinganine |
| Aminosugars metabolism | 2 | 6 | 0.035291152 | E191,E181 | C00217/C05938/C00025,C00128 | D-Glutamate; D-Glutamic acid; D-Glutaminic acid; D-2-Aminoglutaric acid/L-4-Hydroxyglutamate semialdehyde/L-Glutamate; L-Glutamic acid; L-Glutaminic acid; Glutamate$CMP-N-acetylneuraminate |
| Fatty acid oxidation, peroxisome | - | - | - | - | - | - |
| Parathio degradation | 1 | 1 | 0.0392404 | E40 | C00870 | 4-Nitrophenol; p-Nitrophenol; PNP; Niphen; 4-Hydroxynitrobenzene |

SD 4

Kidney

| pathway | overlap size | pathway size | p-value | overlap Empirical Compounds | overlap features | overlap features name |
|---|---|---|---|---|---|---|
| Beta-Alanine metabolism | 6 | 6 | 0.001092345 | E322,E672,E402,E56,E422,E356 | C05145/C01026/C01205/C02356/C00334/C03284,C00025/C00217/C05938,C00222/C00022,C00402/C00049,C00184/C00256/C00186/C01013,C00135 | 3-Aminoisobutyric acid; 3-Aminoisobutanoate; 3-Amino-2-methylpropanoate/N,N-Dimethylglycine; Dimethylglycine/(R)-3-Amino-2-methylpropanoate; D-3-Amino-isobutanoate/(S)-2-Aminobutanoate; (S)-2-Aminobutanoic acid; (S)-2-Aminobutyric acid/4-Aminobutanoate; 4-Aminobutanoic acid; 4-Aminobuty |
| Valine, leucine and isoleucine degradation | 2 | 3 | 7.56E-04 | E184,E205 | C00431/C00183/C00719,C01188/C06001/C01089/C03197/C00989/C05984 | 5-Aminopentanoate; 5-Aminopentanoic acid; 5-Aminovaleric acid/L-Valine; 2-Amino-3-methylbutyric acid/Betaine; Trimethylaminoacetate; Glycine betaine; N,N,N-Trimethylglycine; Trimethylammonioacetate$3-Hydroxy-2-methylpropanoate; 3-Hydroxyisobutyrate; 3-Hydroxyisobutyric acid/(S)-3-Hydroxyisobu |
| Butanoate metabolism | 8 | 10 | 0.001512478 | E442,E286,E485,E672,E402,E322,E206,E745 | C00362/C00020,C00266/C00033,C00042/C02170,C00025/C00217/C05938,C00222/C00022,C05145/C01026/C01205/C02356/C00334/C03284,C03087,C06001/C01089/C03197/C00989/C05984 | dGMP; 2'-Deoxyguanosine 5'-monophosphate; 2'-Deoxyguanosine 5'-phosphate; Deoxyguanylic acid; Deoxyguanosine monophosphate/AMP; Adenosine 5'-monophosphate; Adenylic acid; Adenylate; 5'-AMP; 5'-Adenylic acid; 5'-Adenosine monophosphate; Adenosine 5'-phosphate$Glycolaldehyde; Hydroxyace |
| Tryptophan metabolism | 2 | 4 | 0.001764558 | E79,E202 | C02470,C05645 | Xanthurenic acid; Xanthurenate$4-(2-Amino-3-hydroxyphenyl)-2,4-dioxobutanoate |
| Pyruvate Metabolism | 5 | 5 | 0.002268717 | E402,E289,E128,E286,E422 | C00222/C00022,C00149,CE0737/C00546/C00511,C00266/C00033,C00184/C00256/C00186/C01013 | 3-Oxopropanoate; Malonate semialdehyde/Pyruvate; Pyruvic acid; 2-Oxopropanoate; 2-Oxopropanoic acid; Pyroracemic acid$(S)-Malate; L-Malate; L-Apple acid; L-Malic acid; L-2-Hydroxybutanedioic acid$malonic dialdehyde/Methylglyoxal; Pyruvaldehyde; Pyruvic aldehyde; 2-Ketopropionaldehyde; 2-Oxopron |
| Aspartate and asparagine metabolism | 10 | 15 | 0.00268885 | E442,E322,E465,E542,E672,E56,E286,E486,E745,E233 | C00362/C00020,C05145/C01026/C01205/C02356/C00334/C03284,C02266/C01087/C02630/C03196,C04281/C04282/C01879,C00025/C00217/C05938,C00402/C00049,C00266/C00033,C00097,C06001/C01089/C03197/C00989/C05984,C02238 | dGMP; 2'-Deoxyguanosine 5'-monophosphate; 2'-Deoxyguanosine 5'-phosphate; Deoxyguanylic acid; Deoxyguanosine monophosphate/AMP; Adenosine 5'-monophosphate; Adenylic acid; Adenylate; 5'-AMP; 5'-Adenylic acid; 5'-Adenosine monophosphate; Adenosine 5'-phosphate$3-Aminoisobutyric acid; 3-A |
| Ascorbate (Vitamin C) and Aldarate Metabolism | 4 | 8 | 0.002856903 | E636,E242,E203,E41 | C02045,C01620,C00257/C00800,CE5985 | $$D-Gluconic acid; D-Gluconate; D-gluco-Hexonic acid/L-Gulonate; L-Gulonic acid; Gulonate; Gulonic acid$ |
| Alanine and Aspartate Metabolism | 6 | 7 | 0.003193009 | E442,E286,E672,E402,E56,E695 | C00362/C00020,C00266/C00033,C00025/C00217/C05938,C00222/C00022,C00402/C00049,C00122 | dGMP; 2'-Deoxyguanosine 5'-monophosphate; 2'-Deoxyguanosine 5'-phosphate; Deoxyguanylic acid; Deoxyguanosine monophosphate/AMP; Adenosine 5'-monophosphate; Adenylic acid; Adenylate; 5'-AMP; 5'-Adenylic acid; 5'-Adenosine monophosphate; Adenosine 5'-phosphate$Glycolaldehyde; Hydroxyace |
| Methionine and cysteine metabolism | 8 | 11 | 0.003361062 | E442,E497,E286,E672,E402,E568,E486,E30 | C00362/C00020,C00491,C00266/C00033,C00025/C00217/C05938,C00222/C00022,C00245,C00097,C00302 | dGMP; 2'-Deoxyguanosine 5'-monophosphate; 2'-Deoxyguanosine 5'-phosphate; Deoxyguanylic acid; Deoxyguanosine monophosphate/AMP; Adenosine 5'-monophosphate; Adenylic acid; Adenylate; 5'-AMP; 5'-Adenylic acid; 5'-Adenosine monophosphate; Adenosine 5'-phosphate$L-Cystine; L-Dicysteine; L-al |
| Vitamin B9 (folate) metabolism | - | - | - | - | - | - |
| Histidine metabolism | 4 | 4 | 0.005965885 | E672,E442,E356,E402 | C00025/C00217/C05938,C00362/C00020,C00135,C00222/C00022 | L-Glutamate; L-Glutamic acid; L-Glutaminic acid; Glutamate/D-Glutamate; D-Glutamic acid; D-Glutaminic acid; D-2-Aminoglutaric acid/L-4-Hydroxyglutamate semialdehyde$dGMP; 2'-Deoxyguanosine 5'-monophosphate; 2'-Deoxyguanosine 5'-phosphate; Deoxyguanylic acid; Deoxyguanosine monophosphate/A |
| Vitamin B3 (nicotinate and nicotinamide) metabolism | - | - | - | - | - | - |
| Glycerophospholipid metabolism | 4 | 19 | 0.036467524 | E537,E538,E261,E168 | C00795/C00221/C00095/C00267/C00137/C00738/C00962/C00247/C00984/C00031/C00936/C01582/C00159/C02336/C00293/C00124,C00055,C00266/C00033,C02737 | D-Tagatose; lyxo-Hexulose/beta-D-Glucose/D-Fructose; Levulose; Fruit sugar; D-arabino-Hexulose/alpha-D-Glucose/myo-Inositol; D-myo-Inositol; 1D-myo-Inositol; L-myo-Inositol; 1L-myo-Inositol; meso-Inositol; Inositol; Dambose; Cyclohexitol; Meat sugar; Bios I/D-Hexose/beta-D-Galactose/L-Sorbose; L-xylo-H |
| Pentose phosphate pathway | 3 | 7 | 0.011427611 | E537,E203,E430 | C00795/C00221/C00095/C00267/C00137/C00738/C00962/C00247/C00984/C00031/C00936/C01582/C00159/C02336/C00293/C00124,C00257/C00800,C00312/C00310/C00508/C00259/C00181/C00309/C00121 | D-Tagatose; lyxo-Hexulose/beta-D-Glucose/D-Fructose; Levulose; Fruit sugar; D-arabino-Hexulose/alpha-D-Glucose/myo-Inositol; D-myo-Inositol; 1D-myo-Inositol; L-myo-Inositol; 1L-myo-Inositol; meso-Inositol; Inositol; Dambose; Cyclohexitol; Meat sugar; Bios I/D-Hexose/beta-D-Galactose/L-Sorbose; L-xylo-H |
| Sialic acid metabolism | 3 | 8 | 0.016049072 | E538,E537,E168 | C00055,C00795/C00221/C00095/C00267/C00137/C00738/C00962/C00247/C00984/C00031/C00936/C01582/C00159/C02336/C00293/C00124,C02737 | CMP; Cytidine-5'-monophosphate; Cytidylic acid$D-Tagatose; lyxo-Hexulose/beta-D-Glucose/D-Fructose; Levulose; Fruit sugar; D-arabino-Hexulose/alpha-D-Glucose/myo-Inositol; D-myo-Inositol; 1D-myo-Inositol; L-myo-Inositol; 1L-myo-Inositol; meso-Inositol; Inositol; Dambose; Cyclohexitol; Meat sugar; Bios |
| Chondroitin sulfate degradation | 2 | 3 | 0.014704647 | E537,E430 | C00795/C00221/C00095/C00267/C00137/C00738/C00962/C00247/C00984/C00031/C00936/C01582/C00159/C02336/C00293/C00124,C00312/C00310/C00508/C00259/C00181/C00309/C00121 | D-Tagatose; lyxo-Hexulose/beta-D-Glucose/D-Fructose; Levulose; Fruit sugar; D-arabino-Hexulose/alpha-D-Glucose/myo-Inositol; D-myo-Inositol; 1D-myo-Inositol; L-myo-Inositol; 1L-myo-Inositol; meso-Inositol; Inositol; Dambose; Cyclohexitol; Meat sugar; Bios I/D-Hexose/beta-D-Galactose/L-Sorbose; L-xylo-H |
| Glycosphingolipid biosynthesis - globoseries | 2 | 3 | 0.014704647 | E538,E537 | C00055,C00795/C00221/C00095/C00267/C00137/C00738/C00962/C00247/C00984/C00031/C00936/C01582/C00159/C02336/C00293/C00124 | CMP; Cytidine-5'-monophosphate; Cytidylic acid$D-Tagatose; lyxo-Hexulose/beta-D-Glucose/D-Fructose; Levulose; Fruit sugar; D-arabino-Hexulose/alpha-D-Glucose/myo-Inositol; D-myo-Inositol; 1D-myo-Inositol; L-myo-Inositol; 1L-myo-Inositol; meso-Inositol; Inositol; Dambose; Cyclohexitol; Meat sugar; Bios |
| Glyoxylate and Dicarboxylate Metabolism | 2 | 3 | 0.014704647 | E261,E430 | C00266/C00033,C00312/C00310/C00508/C00259/C00181/C00309/C00121 | Glycolaldehyde; Hydroxyacetaldehyde/Acetate; Acetic acid; Ethanoic acid; Glacial acetic acid$L-Xylulose; L-threo-Pentulose; L-Lyxulose/D-Xylulose; D-threo-Pentulose; D-Lyxulose/L-Ribulose; L-erythro-Pentulose; L-Arabinoketose; L-Arabinulose; L-Riboketose/L-Arabinose; L-Arabinopyranose/D-Xylose; Wood sug |
| Glycine, serine, alanine and threonine metabolism | 8 | 13 | 0.015376859 | E442,E322,E672,E402,E128,E286,E143,E422 | C00362/C00020,C05145/C01026/C01205/C02356/C00334/C03284,C00025/C00217/C05938,C00222/C00022,CE0737/C00546/C00511,C00266/C00033,C00258,C00184/C00256/C00186/C01013 | dGMP; 2'-Deoxyguanosine 5'-monophosphate; 2'-Deoxyguanosine 5'-phosphate; Deoxyguanylic acid; Deoxyguanosine monophosphate/AMP; Adenosine 5'-monophosphate; Adenylic acid; Adenylate; 5'-AMP; 5'-Adenylic acid; 5'-Adenosine monophosphate; Adenosine 5'-phosphate$3-Aminoisobutyric acid; 3-A |
| Biopterin metabolism | - | - | - | - | - | - |
| Arginine and Proline Metabolism | 6 | 9 | 0.020250399 | E485,E542,E672,E402,E56,E695 | C00042/C02170,C04281/C04282/C01879,C00025/C00217/C05938,C00222/C00022,C00402/C00049,C00122 | Succinate; Succinic acid; Butanedionic acid; Ethylenesuccinic acid/Methylmalonate; Methylmalonic acid$L-1-Pyrroline-3-hydroxy-5-carboxylate; 3-Hydroxy-L-1-pyrroline-5-carboxylate; (3R,5S)-1-Pyrroline-3-hydroxy-5-carboxylate/1-Pyrroline-4-hydroxy-2-carboxylate/5-Oxoproline; Pyroglutamic acid; 5-Pyrrolido |
| Aminosugars metabolism | 6 | 9 | 0.020250399 | E286,E672,E23,E402,E436,E706 | C00266/C00033,C00025/C00217/C05938,C03410,C00222/C00022,C04501/C04256/C04257/C00357/C06376,C00055 | Glycolaldehyde; Hydroxyacetaldehyde/Acetate; Acetic acid; Ethanoic acid; Glacial acetic acid$L-Glutamate; L-Glutamic acid; L-Glutaminic acid; Glutamate/D-Glutamate; D-Glutamic acid; D-Glutaminic acid; D-2-Aminoglutaric acid/L-4-Hydroxyglutamate semialdehyde$N-Glycoloyl-neuraminate; N-Glycolylneuram |
| Phosphatidylinositol phosphate metabolism | 3 | 9 | 0.020586505 | E538,E537,E261 | C00055,C00795/C00221/C00095/C00267/C00137/C00738/C00962/C00247/C00984/C00031/C00936/C01582/C00159/C02336/C00293/C00124,C00266/C00033 | CMP; Cytidine-5'-monophosphate; Cytidylic acid$D-Tagatose; lyxo-Hexulose/beta-D-Glucose/D-Fructose; Levulose; Fruit sugar; D-arabino-Hexulose/alpha-D-Glucose/myo-Inositol; D-myo-Inositol; 1D-myo-Inositol; L-myo-Inositol; 1L-myo-Inositol; meso-Inositol; Inositol; Dambose; Cyclohexitol; Meat sugar; Bios |
| Glutathione Metabolism | 3 | 3 | 0.020754558 | E672,E542,E486 | C00025/C00217/C05938,C04281/C04282/C01879,C00097 | L-Glutamate; L-Glutamic acid; L-Glutaminic acid; Glutamate/D-Glutamate; D-Glutamic acid; D-Glutaminic acid; D-2-Aminoglutaric acid/L-4-Hydroxyglutamate semialdehyde$L-1-Pyrroline-3-hydroxy-5-carboxylate; 3-Hydroxy-L-1-pyrroline-5-carboxylate; (3R,5S)-1-Pyrroline-3-hydroxy-5-carboxylate/1-Pyrroline-4- |
| Vitamin B1 (thiamin) metabolism | 3 | 3 | 0.020754558 | E442,E402,E424 | C00362/C00020,C00222/C00022,C01081 | dGMP; 2'-Deoxyguanosine 5'-monophosphate; 2'-Deoxyguanosine 5'-phosphate; Deoxyguanylic acid; Deoxyguanosine monophosphate/AMP; Adenosine 5'-monophosphate; Adenylic acid; Adenylate; 5'-AMP; 5'-Adenylic acid; 5'-Adenosine monophosphate; Adenosine 5'-phosphate$3-Oxopropanoate; Malonat |
| Glycosphingolipid metabolism | - | - | - | - | - | - |
| Selenoamino acid metabolism | - | - | - | - | - | - |
| Heparan sulfate degradation | 2 | 4 | 0.022351063 | E537,E430 | C00795/C00221/C00095/C00267/C00137/C00738/C00962/C00247/C00984/C00031/C00936/C01582/C00159/C02336/C00293/C00124,C00312/C00310/C00508/C00259/C00181/C00309/C00121 | D-Tagatose; lyxo-Hexulose/beta-D-Glucose/D-Fructose; Levulose; Fruit sugar; D-arabino-Hexulose/alpha-D-Glucose/myo-Inositol; D-myo-Inositol; 1D-myo-Inositol; L-myo-Inositol; 1L-myo-Inositol; meso-Inositol; Inositol; Dambose; Cyclohexitol; Meat sugar; Bios I/D-Hexose/beta-D-Galactose/L-Sorbose; L-xylo-H |
| Glycosphingolipid biosynthesis - ganglioseries | 2 | 4 | 0.022351063 | E537,E538 | C00795/C00221/C00095/C00267/C00137/C00738/C00962/C00247/C00984/C00031/C00936/C01582/C00159/C02336/C00293/C00124,C00055 | D-Tagatose; lyxo-Hexulose/beta-D-Glucose/D-Fructose; Levulose; Fruit sugar; D-arabino-Hexulose/alpha-D-Glucose/myo-Inositol; D-myo-Inositol; 1D-myo-Inositol; L-myo-Inositol; 1L-myo-Inositol; meso-Inositol; Inositol; Dambose; Cyclohexitol; Meat sugar; Bios I/D-Hexose/beta-D-Galactose/L-Sorbose; L-xylo-H |
| Glycolysis and Gluconeogenesis | 5 | 7 | 0.022519116 | E442,E402,E289,E286,E422 | C00362/C00020,C00222/C00022,C00149,C00266/C00033,C00184/C00256/C00186/C01013 | dGMP; 2'-Deoxyguanosine 5'-monophosphate; 2'-Deoxyguanosine 5'-phosphate; Deoxyguanylic acid; Deoxyguanosine monophosphate/AMP; Adenosine 5'-monophosphate; Adenylic acid; Adenylate; 5'-AMP; 5'-Adenylic acid; 5'-Adenosine monophosphate; Adenosine 5'-phosphate$3-Oxopropanoate; Malonat |
| Glutamate metabolism | 4 | 5 | 0.023863541 | E672,E402,E322,E485 | C00025/C00217/C05938,C00222/C00022,C05145/C01026/C01205/C02356/C00334/C03284,C00042/C02170 | L-Glutamate; L-Glutamic acid; L-Glutaminic acid; Glutamate/D-Glutamate; D-Glutamic acid; D-Glutaminic acid; D-2-Aminoglutaric acid/L-4-Hydroxyglutamate semialdehyde$3-Oxopropanoate; Malonate semialdehyde/Pyruvate; Pyruvic acid; 2-Oxopropanoate; 2-Oxopropanoic acid; Pyroracemic acid$3-Aminoisc |
| Purine metabolism | 8 | 14 | 0.02487186 | E442,E672,E402,E56,E707,E410,E345,E695 | C00362/C00020,C00025/C00217/C05938,C00222/C00022,C00402/C00049,C01762,C00385,C00294,C00122 | dGMP; 2'-Deoxyguanosine 5'-monophosphate; 2'-Deoxyguanosine 5'-phosphate; Deoxyguanylic acid; Deoxyguanosine monophosphate/AMP; Adenosine 5'-monophosphate; Adenylic acid; Adenylate; 5'-AMP; 5'-Adenylic acid; 5'-Adenosine monophosphate; Adenosine 5'-phosphate$L-Glutamate; L-Glutamic ac |
| Vitamin B6 (pyridoxine) metabolism | 1 | 2 | 0.025544072 | E278 | C00250 | Pyridoxal |
| Propanoate metabolism | 1 | 2 | 0.025712125 | E205 | C01188/C06001/C01089/C03197/C00989/C05984 | 3-Hydroxy-2-methylpropanoate; 3-Hydroxyisobutyrate; 3-Hydroxyisobutyric acid/(S)-3-Hydroxyisobutyrate/(R)-3-Hydroxybutanoate; (R)-3-Hydroxybutanoic acid; (R)-3-Hydroxybutyric acid/(S)-3-Hydroxybutanoate/4-Hydroxybutanoic acid; 4-Hydroxybutanoate; 4-Hydroxybutyric acid/2-Hydroxybutanoic acid; 2 |
| Leukotriene metabolism | - | - | - | - | - | - |
| Pentose and Glucuronate Interconversions | 2 | 5 | 0.033358541 | E203,E430 | C00257/C00800,C00312/C00310/C00508/C00259/C00181/C00309/C00121 | D-Gluconic acid; D-Gluconate; D-gluco-Hexonic acid/L-Gulonate; L-Gulonic acid; Gulonate; Gulonic acid$L-Xylulose; L-threo-Pentulose; L-Lyxulose/D-Xylulose; D-threo-Pentulose; D-Lyxulose/L-Ribulose; L-erythro-Pentulose; L-Arabinoketose; L-Arabinulose; L-Riboketose/L-Arabinose; L-Arabinopyranose/D-Xylose; |
| Urea cycle/amino group metabolism | - | - | - | - | - | - |
| Parathio degradation | - | - | - | - | - | - |
| Ubiquinone Biosynthesis | - | - | - | - | - | - |
| Hexose phosphorylation | - | - | - | - | - | - |
| N-Glycan biosynthesis | 2 | 6 | 0.047727082 | E537,E538 | C00795/C00221/C00095/C00267/C00137/C00738/C00962/C00247/C00984/C00031/C00936/C01582/C00159/C02336/C00293/C00124,C00055 | D-Tagatose; lyxo-Hexulose/beta-D-Glucose/D-Fructose; Levulose; Fruit sugar; D-arabino-Hexulose/alpha-D-Glucose/myo-Inositol; D-myo-Inositol; 1D-myo-Inositol; L-myo-Inositol; 1L-myo-Inositol; meso-Inositol; Inositol; Dambose; Cyclohexitol; Meat sugar; Bios I/D-Hexose/beta-D-Galactose/L-Sorbose; L-xylo-H |
| N-Glycan Degradation | 1 | 1 | 0.048147215 | E537 | C00795/C00221/C00095/C00267/C00137/C00738/C00962/C00247/C00984/C00031/C00936/C01582/C00159/C02336/C00293/C00124 | D-Tagatose; lyxo-Hexulose/beta-D-Glucose/D-Fructose; Levulose; Fruit sugar; D-arabino-Hexulose/alpha-D-Glucose/myo-Inositol; D-myo-Inositol; 1D-myo-Inositol; L-myo-Inositol; 1L-myo-Inositol; meso-Inositol; Inositol; Dambose; Cyclohexitol; Meat sugar; Bios I/D-Hexose/beta-D-Galactose/L-Sorbose; L-xylo-H |
| Proteoglycan biosynthesis | 1 | 1 | 0.048147215 | E261 | C00266/C00033 | Glycolaldehyde; Hydroxyacetaldehyde/Acetate; Acetic acid; Ethanoic acid; Glacial acetic acid |
| Keratan sulfate degradation | 1 | 1 | 0.048147215 | E537 | C00795/C00221/C00095/C00267/C00137/C00738/C00962/C00247/C00984/C00031/C00936/C01582/C00159/C02336/C00293/C00124 | D-Tagatose; lyxo-Hexulose/beta-D-Glucose/D-Fructose; Levulose; Fruit sugar; D-arabino-Hexulose/alpha-D-Glucose/myo-Inositol; D-myo-Inositol; 1D-myo-Inositol; L-myo-Inositol; 1L-myo-Inositol; meso-Inositol; Inositol; Dambose; Cyclohexitol; Meat sugar; Bios I/D-Hexose/beta-D-Galactose/L-Sorbose; L-xylo-H |

SD 9

**Heart**

| pathway | overlap size | pathway size | p-value | overlap Empirical Compounds | overlap features | overlap features name |
|---|---|---|---|---|---|---|
| Fatty acid activation | 6 | 15 | 1.68E-04 | E313,E19,E718,E557,E333,E764 | C06427/C06426,C03242,C00249,C06427/C06426,C00249,C01595 | (9Z,12Z,15Z)-Octadecatrienoic acid; alpha-Linolenic acid; 9,12,15-Octadecatrienoic acid; Linolenate; alpha-Linolenate/(6Z,9Z,12Z)-Octadecatrienoic acid; 6,9,12-Octadecatrienoic acid; gamma-Linolenic acid; Gamolenic acid$(8Z,11Z,14Z)-Icosatrienoic acid; Dihomo-gamma-linolenic acid; (Z,Z,Z)-8,11,14-Eicosatrienoic acid; (Z,Z,Z)-8,11,14-Icosatrienoic acid; [illegible] |
| De novo fatty acid biosynthesis | 7 | 23 | 6.72E-04 | E313,E19,E718,E165,E557,E333,E764 | C06427/C06426,C03242,C00249,C06429,C06427/C06426,C00249,C01595 | (9Z,12Z,15Z)-Octadecatrienoic acid; alpha-Linolenic acid; 9,12,15-Octadecatrienoic acid; Linolenate; alpha-Linolenate/(6Z,9Z,12Z)-Octadecatrienoic acid; 6,9,12-Octadecatrienoic acid; gamma-Linolenic acid; Gamolenic acid$(8Z,11Z,14Z)-Icosatrienoic acid; Dihomo-gamma-linolenic acid; (Z,Z,Z)-8,11,14-Eicosatrienoic acid; (Z,Z,Z)-8,11,14-Icosatrienoic acid; [illegible] |
| Xenobiotics metabolism | 3 | 5 | 0.0022687 | E333,E18,E718 | C00249,C00829,C00249 | Hexadecanoic acid; Hexadecanoate; Hexadecylic acid; Palmitic acid; Palmitate; Cetylic acid$Naphthalene$Hexadecanoic acid; Hexadecanoate; Hexadecylic acid; Palmitic acid; Palmitate; Cetylic acid |
| Fructose and mannose metabolism | - | - | - | - | - | - |
| Nucleotide Sugar Metabolism | - | - | - | - | - | - |
| Linoleate metabolism | 4 | 12 | 0.0058819 | E313,E764,E926,E557 | C06427/C06426,C01595,C14762/C14825/C14826,C06427/C06426 | (9Z,12Z,15Z)-Octadecatrienoic acid; alpha-Linolenic acid; 9,12,15-Octadecatrienoic acid; Linolenate; alpha-Linolenate/(6Z,9Z,12Z)-Octadecatrienoic acid; 6,9,12-Octadecatrienoic acid; gamma-Linolenic acid; Gamolenic acid$Linoleate; Linoleic acid; (9Z,12Z)-Octadecadienoic acid; 9-cis,12-cis-Octadecadienoate; 9-cis,12-cis-Octadecadienoic acid$13(S)-HODE; [illegible] |
| Fatty Acid Metabolism | 3 | 8 | 0.0086547 | E333,E764,E718 | C00249,C01595,C00249 | Hexadecanoic acid; Hexadecanoate; Hexadecylic acid; Palmitic acid; Palmitate; Cetylic acid$Linoleate; Linoleic acid; (9Z,12Z)-Octadecadienoic acid; 9-cis,12-cis-Octadecadienoate; 9-cis,12-cis-Octadecadienoic acid$Hexadecanoic acid; Hexadecanoate; Hexadecylic acid; Palmitic acid; Palmitate; Cetylic acid |
| Polyunsaturated fatty acid biosynthesis | - | - | - | - | - | - |
| Carbon fixation | - | - | - | - | - | - |
| Glycolysis and Gluconeogenesis | - | - | - | - | - | - |
| Hexose phosphorylation | - | - | - | - | - | - |
| Galactose metabolism | - | - | - | - | - | - |
| Arachidonic acid metabolism | 3 | 9 | 0.0120998 | E901,E313,E557 | leuktrE4,C06427/C06426,C06427/C06426 | leukotriene E4$(9Z,12Z,15Z)-Octadecatrienoic acid; alpha-Linolenic acid; 9,12,15-Octadecatrienoic acid; Linolenate; alpha-Linolenate/(6Z,9Z,12Z)-Octadecatrienoic acid; 6,9,12-Octadecatrienoic acid; gamma-Linolenic acid; Gamolenic acid$(9Z,12Z,15Z)-Octadecatrienoic acid; alpha-Linolenic acid; 9,12,15-Octadecatrienoic acid; Linolenate; alpha-Linolenate/(6Z,9Z,12 [illegible] |
| Omega-3 fatty acid metabolism | 2 | 4 | 0.012688 | E313,E557 | C06427/C06426,C06427/C06426 | [illegible] |
| Pyrimidine metabolism | - | - | - | - | - | - |
| Glycosphingolipid metabolism | 4 | 16 | 0.0151248 | E333,E277,E48,E718 | C00249,C00836,C01747,C00249 | Hexadecanoic acid; Hexadecanoate; Hexadecylic acid; Palmitic acid; Palmitate; Cetylic acid$Sphinganine; Dihydrosphingosine; 2-Amino-1,3-dihydroxyoctadecane$Psychosine; Galactosylsphingosine; O-Galactosylsphingosine$Hexadecanoic acid; Hexadecanoate; Hexadecylic acid; Palmitic acid; Palmitate; Cetylic acid |
| Starch and Sucrose Metabolism | - | - | - | - | - | - |
| Glycosylphosphatidylinositol(GPI)-anchor biosynthesis | - | - | - | - | - | - |
| Saturated fatty acids beta-oxidation | 2 | 5 | 0.0211747 | E333,E718 | C00249,C00249 | Hexadecanoic acid; Hexadecanoate; Hexadecylic acid; Palmitic acid; Palmitate; Cetylic acid$Hexadecanoic acid; Hexadecanoate; Hexadecylic acid; Palmitic acid; Palmitate; Cetylic acid |
| N-Glycan biosynthesis | - | - | - | - | - | - |
| Nitrogen metabolism | - | - | - | - | - | - |
| Valine, leucine and isoleucine degradation | - | - | - | - | - | - |
| Fatty acid oxidation, peroxisome | 1 | 1 | 0.0291572 | E509 | ttccoa/lgnccoa/CE2257 | tetracosanoyl-CoA (n-C24:0CoA)/lignocericyl coenzyme A/tetracosanoyl-CoA |
| Aminosugars metabolism | - | - | - | - | - | - |
| Pentose phosphate pathway | - | - | - | - | - | - |
| Arginine and Proline Metabolism | - | - | - | - | - | - |
| Butanoate metabolism | - | - | - | - | - | - |
| Alanine and Aspartate Metabolism | - | - | - | - | - | - |

SD 9

**Spleen**

| pathway | overlap size | pathway size | p-value | overlap Empirical Compounds | overlap features | overlap features name |
|---|---|---|---|---|---|---|
| Glycolysis and Gluconeogenesis | 7 | 7 | 5.88E-04 | E386,E241,E605,E649,E313,E439,E131 | [illegible] | AMP; Adenosine 5'-monophosphate; Adenylic acid; Adenylate; 5'-AMP; 5'-Adenylic acid; 5'-Adenosine monophosphate; Adenosine 5'-phosphate/dGMP; 2'-Deoxyguanosine 5'-monophosphate; 2'-Deoxyguanosine 5'-phosphate; Deoxyguanylic acid; Deoxyguanosine monophosphate$alpha-D-Glucose 1,6-bisphosphate; alpha-D-Glucose 1,6-biphosphate/beta-D- [illegible] |
| Pentose phosphate pathway | 7 | 9 | 6.72E-04 | E314,E496,E89,E600,E607,E501,E589 | [illegible] | L-Ribulose 5-phosphate/alpha-D-Ribose 5-phosphate/alpha-D-Ribose 1-phosphate/D-Ribulose 5-phosphate$DL-Glyceraldehyde 3-phosphate/Glycerone phosphate; Dihydroxyacetone phosphate/D-Glyceraldehyde 3-phosphate; (2R)-2-Hydroxy-3-(phosphonooxy)-propanal$D-Sedoheptulose 7-phosphate; D-altro-Heptulose 7-phosphate$D-Tagatose; lyxo-Hexu [illegible] |
| Glycine, serine, alanine and threonine metabolism | 8 | 10 | 7.56E-04 | E211,E462,E26,E399,E459,E129,E70,E19 | CE1554,C00025,C00431/C00183/C00719,C00248,C01419,C00256/C00186/C00184/C01013/C00577,C00051,CE0469 | N-acetyl-L-alanine$L-Glutamate; L-Glutamic acid; L-Glutaminic acid; Glutamate$5-Aminopentanoate; 5-Aminopentanoic acid; 5-Aminovaleric acid/L-Valine; 2-Amino-3-methylbutyric acid/Betaine; Trimethylaminoacetate; Glycine betaine; N,N,N-Trimethylglycine; Trimethylammonioacetate$Lipoamide; Thioctic acid amide$Cys-Gly; L-Cysteinylglycine$(R)-Lactate; D-Lact [illegible] |
| Tryptophan metabolism | 7 | 9 | 0.0088228 | E386,E203,E64,E672,E218,E303,E439 | C00020/C00362,C02220,C00051,C10164,C00253,C03824,C05938/C00217/C00025,C00795/C00221/C00267/C00031/C00137/C00738/C00095/C00962/C00247/C01582/C00984/C00936/C00159/C02336/C00293/C00124 | AMP; Adenosine 5'-monophosphate; Adenylic acid; Adenylate; 5'-AMP; 5'-Adenylic acid; 5'-Adenosine monophosphate; Adenosine 5'-phosphate/dGMP; 2'-Deoxyguanosine 5'-monophosphate; 2'-Deoxyguanosine 5'-phosphate; Deoxyguanylic acid; Deoxyguanosine monophosphate$2-Aminomuconate; 2-Aminomuconic acid; o-Aminomuconate$Glutathione; S-L- [illegible] |
| Aspartate and asparagine metabolism | 6 | 7 | 0.0014285 | E564,E462,E300,E384,E459,E70 | C01239,C00025,C00217/C05938/C00302,C00169,C01419,C00051 | N-Acetyl-beta-D-glucosaminylamine$L-Glutamate; L-Glutamic acid; L-Glutaminic acid; Glutamate$D-Glutamate; D-Glutamic acid; D-Glutaminic acid; D-2-Aminoglutaric acid/L-4-Hydroxyglutamate semialdehyde/DL-Glutamate; DL-Glutaminic acid; 2-Aminoglutaric acid; Glutamate$Carbamoyl phosphate$Cys-Gly; L-Cysteinylglycine$Glutathione; S-L-Glutamyl-L-cystei [illegible] |
| Fructose and mannose metabolism | 5 | 6 | 0.0035291 | E600,E333,E501,E496,E285 | [illegible] | D-Tagatose; lyxo-Hexulose/alpha-D-Glucose/D-Glucose; Grape sugar; Dextrose/myo-Inositol; D-myo-Inositol; 1D-myo-Inositol; L-myo-Inositol; 1L-myo-Inositol; meso-Inositol; Inositol; Dambose; Cyclohexitol; Meat sugar; Bios I/D-Hexose/beta-D-Glucose/D-Fructose; Levulose; Fruit sugar; D-arabino-Hexulose/beta-D-Galactose/L-Sorbose; L-xylo-Hexulose/(Galacto [illegible] |
| Prostaglandin formation from arachidonate | - | - | - | - | - | - |
| Vitamin B3 (nicotinate and nicotinamide) metabolism | 5 | 6 | 0.0156289 | E386,E506,E303,E581,E672 | C00020/C00362,C01185,C05938/C00217/C00025,C01101/C03736/C00442/C00199,C10164/C00253 | AMP; Adenosine 5'-monophosphate; Adenylic acid; Adenylate; 5'-AMP; 5'-Adenylic acid; 5'-Adenosine monophosphate; Adenosine 5'-phosphate/dGMP; 2'-Deoxyguanosine 5'-monophosphate; 2'-Deoxyguanosine 5'-phosphate; Deoxyguanylic acid; Deoxyguanosine monophosphate$Nicotinate D-ribonucleotide; beta-Nicotinate D-ribonucleotide; Nicotinate ribo [illegible] |
| Pyruvate Metabolism | 5 | 5 | 0.0049576 | E64,E313,E606,E605,E131 | C00051,C00661/C00111/C00118,C00546/CE0737/C00511,C00149,C00256/C00186/C00184/C01013 | Glutathione; S-L-Glutamyl-L-cysteinylglycine; N-(N-gamma-L-Glutamyl-L-cysteinyl)glycine; gamma-L-Glutamyl-L-cysteinyl-glycine; GSH; Reduced glutathione$DL-Glyceraldehyde 3-phosphate/Glycerone phosphate; Dihydroxyacetone phosphate/D-Glyceraldehyde 3-phosphate; (2R)-2-Hydroxy-3-(phosphonooxy)-propanal$Methylglyoxal; Pyruvaldehyde; Pyruvic ald [illegible] |
| Butanoate metabolism | 6 | 7 | 0.0068062 | E386,E405,E674,E731,E141,E303 | C00020/C00362,C00042/C02170,C00164/C00109/C00349/C06002/C00232,C06001/C01089/C03197/C00989/C05984,C06144,C05938/C00217/C00025 | AMP; Adenosine 5'-monophosphate; Adenylic acid; Adenylate; 5'-AMP; 5'-Adenylic acid; 5'-Adenosine monophosphate; Adenosine 5'-phosphate/dGMP; 2'-Deoxyguanosine 5'-monophosphate; 2'-Deoxyguanosine 5'-phosphate; Deoxyguanylic acid; Deoxyguanosine monophosphate$Succinate; Succinic acid; Butanedioic acid; Ethylenesuccinic acid/Methylmalona [illegible] |
| Ascorbate (Vitamin C) and Aldarate Metabolism | 6 | 7 | 0.0068062 | E253,E64,E639,E172,E442,E607 | C00311/C00158/C04575,C00051,C02045,C02147,C00818,C01620 | Isocitrate; Isocitric acid; 1-Hydroxytricarballylic acid; 1-Hydroxypropane-1,2,3-tricarboxylic acid/Citrate; Citric acid; 2-Hydroxy-1,2,3-propanetricarboxylic acid; 2-Hydroxytricarballylic acid/$Glutathione; S-L-Glutamyl-L-cysteinylglycine; N-(N-gamma-L-Glutamyl-L-cysteinyl)glycine; gamma-L-Glutamyl-L-cysteinyl-glycine; GSH; Reduced glutathione$Dihydrolipoate; Di [illegible] |
| Galactose metabolism | 6 | 7 | 0.0068062 | E229,E241,E649,E222,E313,E439 | [illegible] | D-Glyceraldehyde$alpha-D-Glucose 1,6-bisphosphate; alpha-D-Glucose 1,6-biphosphate/beta-D-Fructose 1,6-bisphosphate/D-Mannose 1,6-bisphosphate/beta-D-Fructose 2,6-bisphosphate; D-Fructose 2,6-bisphosphate/1D-myo-Inositol 1,4-bisphosphate; D-myo-Inositol 1,4-bisphosphate; myo-Inositol 1,4-bisphosphate; Inositol 1,4-bisphosphate/D-Tagato [illegible] |
| Polyunsaturated fatty acid biosynthesis | - | - | - | - | - | - |
| Valine, leucine and isoleucine degradation | 3 | 3 | 0.0085707 | E26,E399,E462 | C00431/C00183/C00719,C00248,C00025 | 5-Aminopentanoate; 5-Aminopentanoic acid; 5-Aminovaleric acid/L-Valine; 2-Amino-3-methylbutyric acid/Betaine; Trimethylaminoacetate; Glycine betaine; N,N,N-Trimethylglycine; Trimethylammonioacetate$Lipoamide; Thioctic acid amide$L-Glutamate; L-Glutamic acid; L-Glutaminic acid; Glutamate |
| Pyrimidine metabolism | 3 | 3 | 0.0085707 | E518,E462,E384 | C05345/C00092/C00085/C00103/C01172/C01177/C00636/C03862/C04006/C01097/C01094/C03546/C00446/C00275/CE3075,C00025,C00169 | beta-D-Fructose 6-phosphate/D-Glucose 6-phosphate; Glucose 6-phosphate; Robison ester/D-Fructose 6-phosphate; D-Fructose 6-phosphoric acid; Neuberg ester/D-Glucose 1-phosphate; alpha-D-Glucose 1-phosphate; Cori ester; D-Glucose alpha-1-phosphate/beta-D-Glucose 6-phosphate/Inositol 1-phosphate; myo-Inositol 1-phosphate; 1D-myo-Inositol 1- [illegible] |
| Lysine metabolism | 3 | 3 | 0.0085707 | E399,E323,E462 | C00248,C00318,C00025 | Lipoamide; Thioctic acid amide$L-Carnitine; L-gamma-Trimethyl-beta-hydroxybutyrobetaine; Vitamin BT; 3-Carboxy-2-hydroxy-N,N,N-trimethyl-1-propanaminium hydroxide, inner salt; Levocarnitine; (R)-Carnitine$L-Glutamate; L-Glutamic acid; L-Glutaminic acid; Glutamate |
| Carbon fixation | 4 | 5 | 0.0097471 | E333,E607,E501,E496 | C01231/C05378/C03693/C00665/C01220/C00354/C04063/C04062/C03785,C00117/C00620/C00231,C00092/C02965/C05345/C00085/C00103/C02976/C01171/C01172/C01177/C00636/C03862/C04006/C01097/C01094/C00668/C03546/C00446/C03735/C00275/CE3075,C00661/C00111/C0011 [illegible] | alpha-D-Glucose 1,6-bisphosphate; alpha-D-Glucose 1,6-biphosphate/beta-D-Fructose 1,6-bisphosphate/D-Mannose 1,6-bisphosphate/beta-D-Fructose 2,6-bisphosphate; D-Fructose 2,6-bisphosphate/1D-myo-Inositol 1,4-bisphosphate; D-myo-Inositol 1,4-bisphosphate; myo-Inositol 1,4-bisphosphate; Inositol 1,4-bisphosphate/D-Fructose 1,6-bisphosphat [illegible] |
| Purine metabolism | 4 | 5 | 0.0102512 | E538,E364,E130,E462 | C03373,C00262,C00385,C00025 | Aminoimidazole ribotide; AIR; 1-(5'-Phosphoribosyl)-5-aminoimidazole; 5'-Phosphoribosyl-5-aminoimidazole; 1-(5-Phospho-D-ribosyl)-5-aminoimidazole; 5-Amino-1-(5-phospho-D-ribosyl)imidazole$Hypoxanthine; Purine-6-ol$Xanthine$L-Glutamate; L-Glutamic acid; L-Glutaminic acid; Glutamate |
| Fatty acid activation | - | - | - | - | - | - |
| TCA cycle | 4 | 4 | 0.0120998 | E405,E605,E328,E253 | C00042/C02170,C00149,C00122,C00311/C00158/C04575 | Succinate; Succinic acid; Butanedioic acid; Ethylenesuccinic acid/Methylmalonate; Methylmalonic acid$(S)-Malate; L-Malate; L-Apple acid; L-Malic acid; L-2-Hydroxybutanedioic acid$Fumarate; Fumaric acid; trans-Butenedioic acid$Isocitrate; Isocitric acid; 1-Hydroxytricarballylic acid; 1-Hydroxypropane-1,2,3-tricarboxylic acid/Citrate; Citric acid; 2-Hydroxy-1,2,3-p [illegible] |
| Propanoate metabolism | 4 | 4 | 0.0120998 | E386,E731,E674,E131 | C00020/C00362,C06001/C01089/C03197/C00989/C05984,C00164/C00109/C00349/C06002/C00232,C00256/C00186/C00184/C01013 | AMP; Adenosine 5'-monophosphate; Adenylic acid; Adenylate; 5'-AMP; 5'-Adenylic acid; 5'-Adenosine monophosphate; Adenosine 5'-phosphate/dGMP; 2'-Deoxyguanosine 5'-monophosphate; 2'-Deoxyguanosine 5'-phosphate; Deoxyguanylic acid; Deoxyguanosine monophosphate$(S)-3-Hydroxyisobutyrate/(R)-3-Hydroxybutanoate; (R)-3-Hydroxybutanoic acid [illegible] |
| Glycerophospholipid metabolism | 9 | 19 | 0.0131922 | E45,E496,E590,E299,E241,E112,E600,E285,E501 | [illegible] | sn-glycero-3-Phosphoethanolamine; Glycerophosphoethanolamine$DL-Glyceraldehyde 3-phosphate/Glycerone phosphate; Dihydroxyacetone phosphate/D-Glyceraldehyde 3-phosphate; (2R)-2-Hydroxy-3-(phosphonooxy)-propanal$CDP-ethanolamine$Glycerone; Dihydroxyacetone; 1,3-Dihydroxyacetone; 1,3-Dihydroxy-2-propanone; 1,3-Dihydroxypropan-2- [illegible] |
| Urea cycle/amino group metabolism | - | - | - | - | - | - |
| Fatty Acid Metabolism | - | - | - | - | - | - |
| De novo fatty acid biosynthesis | - | - | - | - | - | - |
| Drug metabolism - cytochrome P450 | 3 | 4 | 0.0292412 | E576,E461,E244 | CE2173/C16586,C16662,C07501 | /2-Phenyl-1,3-propanediol monocarbamate$L-alpha-Acetyl-N,N-dinormethadol; dinor-LAAM$Felbamate |
| Glutamate metabolism | 3 | 4 | 0.0303336 | E70,E462,E384 | C00051,C00025,C00169 | Glutathione; S-L-Glutamyl-L-cysteinylglycine; N-(N-gamma-L-Glutamyl-L-cysteinyl)glycine; gamma-L-Glutamyl-L-cysteinyl-glycine; GSH; Reduced glutathione$L-Glutamate; L-Glutamic acid; L-Glutaminic acid; Glutamate$Carbamoyl phosphate |
| Glutathione Metabolism | 3 | 4 | 0.0303336 | E70,E459,E462 | C00051,C01419,C00025 | Glutathione; S-L-Glutamyl-L-cysteinylglycine; N-(N-gamma-L-Glutamyl-L-cysteinyl)glycine; gamma-L-Glutamyl-L-cysteinyl-glycine; GSH; Reduced glutathione$Cys-Gly; L-Cysteinylglycine$L-Glutamate; L-Glutamic acid; L-Glutaminic acid; Glutamate |
| Putative anti-Inflammatory metabolites formation from EPA | 2 | 2 | 0.031762 | E70,E462 | C00051,C00025 | Glutathione; S-L-Glutamyl-L-cysteinylglycine; N-(N-gamma-L-Glutamyl-L-cysteinyl)glycine; gamma-L-Glutamyl-L-cysteinyl-glycine; GSH; Reduced glutathione$L-Glutamate; L-Glutamic acid; L-Glutaminic acid; Glutamate |
| Histidine metabolism | 2 | 2 | 0.031762 | E70,E462 | C00051,C00025 | Glutathione; S-L-Glutamyl-L-cysteinylglycine; N-(N-gamma-L-Glutamyl-L-cysteinyl)glycine; gamma-L-Glutamyl-L-cysteinyl-glycine; GSH; Reduced glutathione$L-Glutamate; L-Glutamic acid; L-Glutaminic acid; Glutamate |
| Arginine and Proline Metabolism | 2 | 2 | 0.031762 | E300,E462 | C00217/C05938/C00302,C00025 | D-Glutamate; D-Glutamic acid; D-Glutaminic acid; D-2-Aminoglutaric acid/L-4-Hydroxyglutamate semialdehyde/DL-Glutamate; DL-Glutaminic acid; 2-Aminoglutaric acid; Glutamate$L-Glutamate; L-Glutamic acid; L-Glutaminic acid; Glutamate |
| Beta-Alanine metabolism | 2 | 2 | 0.031762 | E129,E462 | C00256/C00186/C00184/C01013/C00577,C00025 | (R)-Lactate; D-Lactate; D-Lactic acid; D-2-Hydroxypropanoic acid; D-2-Hydroxypropionic acid/(S)-Lactate; L-Lactate; L-Lactic acid/Glycerone; Dihydroxyacetone; 1,3-Dihydroxyacetone; 1,3-Dihydroxy-2-propanone; 1,3-Dihydroxypropan-2-one/3-Hydroxypropanoate; 3-Hydroxypropanoic acid; 3-Hydroxypropionate; 3-Hydroxypropionic acid; Hydracrylic acid/D-Gly [illegible] |
| Trihydroxycoprostanoyl-CoA beta-oxidation | 2 | 2 | 0.031762 | E215,E100 | CE5169,CE5168 | 24-oxo-25(R)-trihydroxycoprostanoyl-CoA$24(R),25(R)-varanoyl-CoA |
| Glycosphingolipid metabolism | - | - | - | - | - | - |
| Aminosugars metabolism | - | - | - | - | - | - |
| Fatty acid oxidation, peroxisome | 2 | 2 | 0.0373918 | E492,E474 | CE2245/CE5159/nrvnccoa,C00042/C02170 | trans-tetracos-2-enoyl-CoA/cis-15-tetracosenoyl-CoA/nervonyl coenzyme A$Succinate; Succinic acid; Butanedioic acid; Ethylenesuccinic acid/Methylmalonate; Methylmalonic acid |
| Parathio degradation | - | - | - | - | - | - |

SD 9

**Kidney**

| pathway | overlap size | pathway size | p-value | overlap Empirical Compounds | overlap features | overlap features name |
|---|---|---|---|---|---|---|
| Beta-Alanine metabolism | 6 | 6 | 5.88E-04 | E713,E472,E581,E71,E539,E648 | C00217/C05938/C00025,C00135,C00186/C00184/C01013/C00256,C00222/C00022,C00402/C00049,C01026/C01205/C02356/C00334/C03284/C05145 | D-Glutamate; D-Glutamic acid; D-Glutaminic acid; D-2-Aminoglutaric acid/L-4-Hydroxyglutamate semialdehyde/L-Glutamate; L-Glutamic acid; L-Glutaminic acid; Glutamate$L-Histidine; (S)-alpha-Amino-1H-imidazole-4-propionic acid$(S)-Lactate; L-Lactate; L-Lactic acid/Glycerone; Dihydroxyacetone; 1,3-Dihydroxyacetone; 1,3-Dihydroxy-2-propanone; 1,3-Dihydro [illegible] |
| Valine, leucine and isoleucine degradation | - | - | - | - | - | - |
| Butanoate metabolism | - | - | - | - | - | - |
| Tryptophan metabolism | 3 | 10 | 0.0233594 | E498,E461,E524 | C03824,C02220,C10164/C00253 | 2-Aminomuconate semialdehyde; 2-Aminomuconate 6-semialdehyde$2-Aminomuconate; 2-Aminomuconic acid; o-Aminomuconate$/Nicotinate; Nicotinic acid; Niacin; 3-Pyridinecarboxylic acid |
| Pyruvate Metabolism | - | - | - | - | - | - |
| Aspartate and asparagine metabolism | - | - | - | - | - | - |
| Ascorbate (Vitamin C) and Aldarate Metabolism | 6 | 8 | 0.0073103 | E716,E281,E505,E627,E13,E43 | glac/C00072/C02670,CE5985,C00311/C00158/C04575,C01620,C02045,C00818 | D-glucurono-6,3-lactone/Ascorbate; Ascorbic acid; L-Ascorbate; L-Ascorbic acid; Vitamin C/D-Glucuronolactone; Glucurone; Glucofuranurono-6,3-lactone; D-Glucurono-3,6-lactone; D-Glucurone$$Isocitrate; Isocitric acid; 1-Hydroxytricarballylic acid; 1-Hydroxypropane-1,2,3-tricarboxylic acid/Citrate; Citric acid; 2-Hydroxy-1,2,3-propanetricarboxylic acid; 2-Hydr [illegible] |
| Alanine and Aspartate Metabolism | - | - | - | - | - | - |
| Methionine and cysteine metabolism | 1 | 3 | 0.022015 | E146 | C00065/C00740 | L-Serine; L-2-Amino-3-hydroxypropionic acid; L-3-Hydroxy-alanine; Serine/D-Serine |
| Vitamin B9 (folate) metabolism | 1 | 1 | 0.0055458 | E146 | C00065/C00740 | L-Serine; L-2-Amino-3-hydroxypropionic acid; L-3-Hydroxy-alanine; Serine/D-Serine |
| Histidine metabolism | 3 | 4 | 0.0468028 | E713,E71,E472 | C00217/C05938/C00025,C00222/C00022,C00135 | D-Glutamate; D-Glutamic acid; D-Glutaminic acid; D-2-Aminoglutaric acid/L-4-Hydroxyglutamate semialdehyde/L-Glutamate; L-Glutamic acid; L-Glutaminic acid; Glutamate$3-Oxopropanoate; Malonate semialdehyde/Pyruvate; Pyruvic acid; 2-Oxopropanoate; 2-Oxopropanoic acid; Pyroracemic acid$L-Histidine; (S)-alpha-Amino-1H-imidazole-4-propionic acid |
| Vitamin B3 (nicotinate and nicotinamide) metabolism | 3 | 6 | 0.0073103 | E457,E524,E547 | C00049/C00402,C10164/C00253,C01185 | L-Aspartate; L-Aspartic acid; 2-Aminosuccinic acid/D-Aspartate; D-Aspartic acid$/Nicotinate; Nicotinic acid; Niacin; 3-Pyridinecarboxylic acid$Nicotinate D-ribonucleotide; beta-Nicotinate D-ribonucleotide; Nicotinate ribonucleotide; Nicotinic acid ribonucleotide |
| Glycerophospholipid metabolism | 2 | 10 | 0.0076464 | E146,E155 | C00065/C00740,C04598 | L-Serine; L-2-Amino-3-hydroxypropionic acid; L-3-Hydroxy-alanine; Serine/D-Serine$2-Acetyl-1-alkyl-sn-glycero-3-phosphocholine |
| Pentose phosphate pathway | - | - | - | - | - | - |
| Sialic acid metabolism | 1 | 2 | 0.0133602 | E146 | C00065/C00740 | L-Serine; L-2-Amino-3-hydroxypropionic acid; L-3-Hydroxy-alanine; Serine/D-Serine |
| Chondroitin sulfate degradation | - | - | - | - | - | - |
| Glycosphingolipid biosynthesis - globoseries | - | - | - | - | - | - |
| Glyoxylate and Dicarboxylate Metabolism | - | - | - | - | - | - |
| Glycine, serine, alanine and threonine metabolism | 1 | 5 | 0.0362154 | E146 | C00065/C00740 | L-Serine; L-2-Amino-3-hydroxypropionic acid; L-3-Hydroxy-alanine; Serine/D-Serine |
| Biopterin metabolism | 2 | 4 | 0.0186539 | E714,E460 | C00079/CE2172,C00082/CE2174 | L-Phenylalanine; (S)-alpha-Amino-beta-phenylpropionic acid/$L-Tyrosine; (S)-3-(p-Hydroxyphenyl)alanine; (S)-2-Amino-3-(p-hydroxyphenyl)propionic acid; Tyrosine/ |
| Arginine and Proline Metabolism | - | - | - | - | - | - |
| Aminosugars metabolism | - | - | - | - | - | - |
| Phosphatidylinositol phosphate metabolism | - | - | - | - | - | - |
| Glutathione Metabolism | - | - | - | - | - | - |
| Vitamin B1 (thiamin) metabolism | - | - | - | - | - | - |
| Glycosphingolipid metabolism | 1 | 3 | 0.022015 | E146 | C00065/C00740 | L-Serine; L-2-Amino-3-hydroxypropionic acid; L-3-Hydroxy-alanine; Serine/D-Serine |
| Selenoamino acid metabolism | 1 | 3 | 0.022015 | E146 | C00065/C00740 | L-Serine; L-2-Amino-3-hydroxypropionic acid; L-3-Hydroxy-alanine; Serine/D-Serine |
| Heparan sulfate degradation | - | - | - | - | - | - |
| Glycosphingolipid biosynthesis - ganglioseries | - | - | - | - | - | - |
| Glycolysis and Gluconeogenesis | - | - | - | - | - | - |
| Glutamate metabolism | - | - | - | - | - | - |
| Purine metabolism | - | - | - | - | - | - |
| Vitamin B6 (pyridoxine) metabolism | - | - | - | - | - | - |
| Propanoate metabolism | - | - | - | - | - | - |
| Leukotriene metabolism | 1 | 4 | 0.0298294 | E366 | CE4995 | 10,11-dihydro-12R-hydroxy-leukotriene E4 |
| Pentose and Glucuronate Interconversions | - | - | - | - | - | - |
| Urea cycle/amino group metabolism | 3 | 12 | 0.0373918 | E26,E457,E268 | C05589/C00788,C00049/C00402,C01026/C01205/C02356/C00334/C03284/C05145 | L-Normetanephrine/L-Adrenaline; (R)-(-)-Adrenaline; (R)-(-)-Epinephrine; (R)-(-)-Epirenamine; (R)-(-)-Adnephrine; 4-[(1R)-1-Hydroxy-2-(methylamino)ethyl]-1,2-benzenediol$L-Aspartate; L-Aspartic acid; 2-Aminosuccinic acid/D-Aspartate; D-Aspartic acid$N,N-Dimethylglycine; Dimethylglycine/(R)-3-Amino-2-methylpropanoate; D-3-Amino-isobutanoate/(S)-2-Ami [illegible] |
| Parathio degradation | 1 | 1 | 0.041257 | E238 | C00870 | 4-Nitrophenol; p-Nitrophenol; PNP; Niphen; 4-Hydroxynitrobenzene |
| Ubiquinone Biosynthesis | 1 | 2 | 0.0457104 | E334 | C05838/C00166/C02763/C00811 | cis-2-Hydroxycinnamate; 2-Coumarinate/Phenylpyruvate; Phenylpyruvic acid; alpha-Ketohydrocinnamic acid; keto-Phenylpyruvate; 3-Phenyl-2-oxopropanoate/enol-Phenylpyruvate; enol-Phenylpyruvic acid; enol-alpha-Ketohydrocinnamic acid; 2-Hydroxy-3-phenylpropenoate/4-Coumarate; p-Coumaric acid; trans-4-Hydroxycinnamate; trans-p-Hydroxycinnamate; [illegible] |
| Hexose phosphorylation | 1 | 2 | 0.0457104 | E334 | C05838/C00166/C02763/C00811 | cis-2-Hydroxycinnamate; 2-Coumarinate/Phenylpyruvate; Phenylpyruvic acid; alpha-Ketohydrocinnamic acid; keto-Phenylpyruvate; 3-Phenyl-2-oxopropanoate/enol-Phenylpyruvate; enol-Phenylpyruvic acid; enol-alpha-Ketohydrocinnamic acid; 2-Hydroxy-3-phenylpropenoate/4-Coumarate; p-Coumaric acid; trans-4-Hydroxycinnamate; trans-p-Hydroxycinnamate; [illegible] |
| N-Glycan biosynthesis | - | - | - | - | - | - |
| N-Glycan Degradation | - | - | - | - | - | - |
| Proteoglycan biosynthesis | - | - | - | - | - | - |
| Keratan sulfate degradation | - | - | - | - | - | - |

-Hexose 6-phosphate/beta-D-Fructose 6-phosp
r; Glucose 6-phosphate; Robison ester/D-Hexos

Hexose/D-Fructose; Levulose; Fr

itol 1,3-bisphosphate; myo-In

ityric acid$3-Butynoate$L-4-Hydroxygl

-inositol 1-phosphate; D-myo-Inositol 1-phospha

nositol 4-phosphate/alpha-D-Galactose 1-phosphate; alpha-D

isphate/Fructose 1-phosphate/alpha-D-Hexose 1-p

(S)-Lactate; L-Lactate; L-Lactic ac
Hydroxyeicosatetraenoic aci

id; gamma-Aminobutyri
